# Supplementary material for: A review of rate control in atrial fibrillation, and the rationale and protocol for the RATE-AF trial
Source: BMJ Open. 2017 Jul 20;7(7):e015099. doi: 10.1136/bmjopen-2016-015099 (PMC5588987; doi:10.1136/bmjopen-2016-015099)
Supplement: Supplementary material 1 [file bmjopen-2016-015099supp001.pdf]

Evaluating different rate control therapies in permanent atrial fibrillation: A prospective, randomised, open-label, blinded endpoint trial comparing digoxin and beta-blockers as initial rate control therapy

RAte control Therapy Evaluation in Atrial Fibrillation:

RATE-AF

RATE-AF TRIAL PROTOCOL

Version 1.0, 23rd March 2016

**Sponsor:**	**University of Birmingham**
**Chief Investigator:**	**Dr Dipak Kotecha**
**Coordinating Unit:**	**Birmingham Clinical Trials Unit**
**Funder:**	**National Institute for Health Research (NIHR) Career Development Fellowship**

**ISRCTN:**	**TBC**
**EudraCT No.:**	**2015-005043-13**
**REC Ref. No.:**	**TBC**

UNIVERSITY OF BIRMINGHAM

BIRMINGHAM CLINICAL TRIALS UNIT

NHS National Institute for Health Research

TRIAL COMMITTEES AND CONTACT DETAILS

Trial Management Group	
**Chief Investigator**<br>**Dr Dipak Kotecha**	**NIHR Career Development Fellow & Clinician Scientist**<br><br>Institute of Cardiovascular Sciences, University of Birmingham, The Medical School, Vincent Drive, Birmingham, B15 2TT, UK<br>Email: d.kotecha@bham.ac.uk<br>Telephone: 07974 115676
**Prof Paulus Kirchhof**	**Professor of Cardiovascular Medicine**<br><br>Institute of Cardiovascular Sciences, University of Birmingham, Institute of Biomedical Research, Vincent Drive, Birmingham B15 2TT, UK<br>Email: p.kirchhof@bham.ac.uk<br>Telephone: 0121 414 7042
**Dr Michael Griffith**	**Consultant Electrophysiologist**<br><br>University Hospitals Birmingham NHS Trust, Nuffield House, Queen Elizabeth Hospital, Birmingham, B15 2TH, UK<br>Email: michael.griffith@uhb.nhs.uk<br>Telephone: 0121 371 4038
**Prof Gregory Y H Lip**	**Professor of Cardiovascular Medicine & Director, Haemostasis Thrombosis & Vascular Biology Unit**<br><br>Institute of Cardiovascular Sciences, University of Birmingham, City Hospital, Birmingham, B18 7QH, UK<br>Email g.y.h.lip@bham.ac.uk<br>Telephone: 0121 5075080
**Prof Jonathan Townend**	**Professor of Cardiology**<br><br>University Hospitals Birmingham NHS Trust, Nuffield House, Queen Elizabeth Hospital, Birmingham, B15 2TH, UK<br>Email: john.townend@uhb.nhs.uk<br>Telephone: 0121 371 4623
**Dr Rick Steeds**	**Consultant Cardiologist and Head of Cardiac Imaging**<br><br>University Hospitals Birmingham NHS Trust, Nuffield House, Queen Elizabeth Hospital, Birmingham, B15 2TH, UK<br>Email: rick.steeds@uhb.nhs.uk<br>Telephone: 0121 371 6130
**Prof Melanie Calvert**	**Professor of Outcomes Methodology**<br><br>Institute of Applied Health Research, University of Birmingham, Birmingham, B15 2TT, UK<br>Email: m.calvert@bham.ac.uk<br>Telephone: 0121 414 8595
**Dr Susan Jowett**	**Senior Lecturer, Health Economics**<br><br>Institute of Applied Health Research, University of Birmingham, Birmingham, B15 2TT, UK<br>Email s.jowett@bham.ac.uk<br>Telephone: 0121 414 7898
**Dr Jonathan Mathers**	**Senior Lecturer, Qualitative and Mixed Methods Applied Health Research**<br><br>Institute of Applied Health Research, University of Birmingham, Birmingham, B15 2TT, UK<br>Email j.m.mathers@bham.ac.uk<br>Telephone: 0121 414 6024

Birmingham Clinical Trials Unit	
**Prof Jon Deeks**	**Professor of Biostatistics and Director, BCTU**<br><br>Birmingham Clinical Trials Unit, University of Birmingham, Birmingham, B15 2TT, UK<br>Email j.deeks@bham.ac.uk<br>Telephone: 0121 414 5328
**Dr Margaret Grant**	**Operations Manager**<br><br>Birmingham Clinical Trials Unit, University of Birmingham, Birmingham, B15 2TT, UK<br>Email m.r.grant@bham.ac.uk<br>Telephone: 0121 415 9106
**Gemma Slinn**	**Senior Trial Coordinator**<br><br>Birmingham Clinical Trials Unit, University of Birmingham, Birmingham, B15 2TT, UK<br>Email g.slinn@bham.ac.uk<br>Telephone: 0121 415 8445
**Samir Mehta**	**Statistician**<br><br>Birmingham Clinical Trials Unit, University of Birmingham, Birmingham, B15 2TT, UK<br>Email s.mehta.1@bham.ac.uk<br>Telephone: 0121 415 9117

Trial Oversight Committee

**Co-Chairs**	
**Dr Kazem Rahimi**	**Associate Professor of Cardiovascular Medicine, University of Oxford**<br>**Deputy Director, The George Institute for Global Health**<br><br>The George Institute for Global Health, University of Oxford, 34 Broad Street, Oxford OX1 3BD, UK<br>Email: kazem.rahimi@georgeinstitute.ox.ac.uk<br>Telephone: 01865 617 201
**Prof. John Camm**	**BHF Professor of Clinical Cardiology**<br><br>St George's University of London, Cranmer Terrace, London SW17 0RE, UK<br>Email: jcamm@sgul.ac.uk<br>Telephone: 0208 725 3414
**Patient Representative**	
**Mary Stanbury**	Lead PPI Representative<br>Email: dms27@btinternet.com
**On behalf of the Trial Management Group**	
**Dr Dipak Kotecha**	
**Prof. Jon Deeks**	For contact details, see Trial Management Group
**Prof. Paulus Kirchhof**	

RATE-AF Trial Office

For general protocol related queries and supply of trial materials:

Birmingham Clinical Trials Unit (BCTU), Institute of Applied Health Research, College of Medical & Dental Sciences, Public Health Building, University of Birmingham, Edgbaston, Birmingham B15 2TT

Telephone: 0121 415 8445
Fax: 0121 415 9135
Email: RATE-AF@trials.bham.ac.uk
Website: www.birmingham.ac.uk/RATE-AF

Randomisation

Telephone: 0800 953 0274

Website: https://www.birmingham.ac.uk/RATEAF

Safety Reporting

Fax SAE Forms to: 0121 415 9135 or 0121 415 9136

Protocol Development and Sign Off

Protocol Amendments				
The following amendments and/ or administrative changes have been made to this protocol since the implementation of the first approved version				
Amendment number	Date of amendment	Protocol version number	Type of amendment	Summary of amendment

Chief Investigator Signature Page

Trial Name: **RATE-AF**

Protocol Version Number: Version: __ __

Protocol Version Date: __ __ / __ __ __ / __ __ __ __

This protocol has been approved by:

CI Name: Dr Dipak Kotecha

Trial Role: Chief Investigator

Signature and date: _________________________ __ __ / __ __ __ / __ __ __ __

Sponsor Statement

Where the University of Birmingham takes on the sponsor role for protocol development oversight, the signing of the IRAS form by the Sponsor will serve as confirmation of the approval of this protocol.

Principal Investigator Signature Page

Principal Investigator:

I have read and agree to the protocol, as detailed in this document. I agree to adhere to the protocol as outlined and agree that any suggested changes to the protocol must be approved by the Trial Oversight Committee prior to seeking approval from the Research Ethics Committee and Regulatory Authority.

I am aware of my responsibilities as an Investigator under the guidelines of Good Clinical Practice (GCP), the Declaration of Helsinki, local regulations (as applicable) and the trial protocol and I agree to conduct the trial according to these guidelines and to appropriately direct and assist the staff under my control, who will be involved in the trial.

Trial Name: **RATE-AF**

Protocol Version Number: Version: __ __

Protocol Version Date: __ __ / __ __ __ / __ __ __ __

PI Name: <Enter>

Trial Role: Principal Investigator

Signature and date: ____________________________ __ __ / __ __ __ / __ __ __ __

The Principal Investigator should sign this page and return a copy to the RATE-AF Trial Office

Table of Contents

1 Trial Summary 13

1.1 Trial Schema 15

2 Introduction 16

2.1 Background 16

2.2 Epidemiology and Consequences of AF 16

2.3 Rhythm-Control in AF 17

2.4 Lack of Evidence to Guide Rate-Control Therapy 17

2.5 Patient Wellbeing 19

2.6 Rationale for the RATE-AF Trial 19

3 Trial Design and Objectives 20

3.1 Hypothesis 20

3.2 Primary objective 21

3.3 Secondary objectives 21

3.4 Feasibility objectives 21

3.5 Exploratory objectives 21

4 Selection of Participants 22

4.1 Inclusion Criteria 22

4.2 Exclusion Criteria 22

5 Informed Consent Process 23

6 Enrolment and Randomisation 24

6.1 Randomisation Procedures 25

7 Trial Treatment 26

7.1 Treatment 26

7.2 Treatment Supply and Storage 26

7.3 Dosing Schedule 27

7.4 Drug Interactions and Contraindications 27

7.5 Accountability Procedures and Labelling 29

7.6 Treatment Modification 29

7.7 Assessment of Compliance 30

8 Trial Procedures and Schedule of Assessments 30

8.1 Baseline Visit 30

8.2 Up-Titration Visits 31
8.3 Visit 2, Month 6 31
8.4 Visit 3, Month 12 (Final Trial Assessment) 32
8.5 Investigator-blinded Endpoints 32
8.6 Long Term Follow-Up 32
8.7 Withdrawal 33
8.8 Trial Duration 33
9 Trial Procedures 35
9.1 Procedures Defined as Standard Clinical Care 35
9.2 Medical History 35
9.3 Medication History 35
9.4 Physical Examination 36
9.5 Patient Reported Outcomes 36
9.5.1 Choice of Outcomes and Qualitative Research 36
9.5.2 Data Collection for PROMs 37
9.5.3 Outcome Appraisal 38
9.6 Transthoracic Echocardiography 38
9.6.1 Reproducibility and Validity of Measurements 38
9.6.2 Systolic LV Function 38
9.6.3 Diastolic LV Function 39
9.6.4 Left Atrial Size and Function 40
9.6.5 Additional Echocardiography Parameters 40
9.7 Laboratory Evaluations 40
9.7.1 Laboratory Assays 41
9.7.2 Cellular Response to Rate Control 41
9.7.3 Stored Blood Samples 41
9.7.4 Specimen Preparation, Handling, Storage and Shipment 41
9.8 Economic Evaluation 41
10 Pharmacovigilance 43
10.1 Recording and Assessment of Adverse Events 43
10.2 Non-Serious Adverse Events/ Adverse Reactions 45
10.3 Serious Adverse Events 45

10.3.1 Expected SAEs NOT to be Reported on a SAE Form 45
10.4 SUSARs 45
10.5 Development Safety Update Reports 46
10.6 Annual Progress Reports 46
10.7 Pregnancy 46
10.8 Reporting Urgent Safety Measures 46
11 Quality Control and Quality Assurance 47
11.1 Site Set-Up and Initiation 47
11.2 Central Monitoring 47
11.3 Audit and Inspection 47
11.4 Notification of Serious Breaches 48
11.5 Data Handling and Analysis 48
11.6 End of Trial 49
11.7 Archiving 49
12 Statistical Considerations 50
12.1 Outcome measures 50
12.1.1 Primary Outcome 50
12.1.2 Secondary Outcomes 50
12.1.3 Feasibility Outcomes 50
12.2 Power Calculations 51
12.3 Statistical analysis 51
12.3.1 Primary outcome analysis 52
12.3.2 Feasibility and Secondary outcomes analysis 52
12.3.3 Missing data and sensitivity analyses 52
12.3.4 Interim analyses and Stopping rules 52
12.4 Final analysis 53
13 Ethics and Regulatory Requirements 53
14 Oversight Committees 53
14.1 Trial Management Group 53
14.2 Trial Oversight Committee 54
14.3 Protocol amendments 54
15 Finance 54

16 Confidentiality and Data Protection 54

17 Insurance and Indemnity 55

18 Dissemination and Publication 55

19 Statement of Compliance 56

20 References 57

Appendix A: Randomised treatment arm - Digoxin

Appendix B: Randomised treatment arm - Bisoprolol

List of Abbreviations

**ABPI**	Association of the British Pharmaceutical Industry
**AE**	Adverse Event
**AF**	Atrial Fibrillation
**BCTU**	Birmingham Clinical Trials Unit
**BNP**	B-type Natriuretic Peptide
**BPM**	Beats per Minute
**CCB**	Calcium Channel Blocker
**CI**	Chief Investigator
**CMR**	Cardiac Magnetic Resonance
**CRF**	Case Report Form
**CTA**	Clinical Trial Authorisation
**CTIMP**	Clinical Trial of Medicinal Product
**DIBD**	Developmental International Birth Date
**DMC**	Data Monitoring Committee
**DSUR**	Developmental Safety Update Report
**DT**	Deceleration Time
**ECG**	Electrocardiogram
**EHRA**	European Heart Rhythm Association
**EU**	European Union
**EudraCT No.**	European Union Drug Regulating Authorities Clinical Trials Number
**GCP**	Good Clinical Practice
**GFR**	Glomerular Filtration Rate
**GP**	General Practitioner
**HF**	Heart Failure
**HR**	Hazard Ratio
**IMP**	Investigational Medicinal Product
**INR**	International Normalised Ratio
**ISRCTN**	International Standard Randomised Controlled Trial Number
**IVRT**	Isovolumic Relaxation Time
**LA**	Left-Atrial
**LV**	Left-Ventricular
**LVEDD**	Left-Ventricle End-Diastolic Dimension
**LVEDV**	Left-Ventricle End-Diastolic Volume
**LVEF**	Left-Ventricular Ejection Fraction

**LVESD**	Left-Ventricle End-Systolic Dimension
**LVESV**	Left-Ventricle End-Systolic Volume
**LVSD**	Left-Ventricular Systolic Dysfunction
**MHRA**	Medicines and Healthcare Products Regulatory Agency
**MREC**	Main Research Ethics Committee
**NHS**	National Health Service
**NICE**	National Institute of Clinical Excellence
**NOAC**	Novel Oral Anticoagulants
**NYHA**	New York Health Association
**PEF**	Preserved Ejection Fraction
**PI**	Principal Investigator
**PIC**	Patient Identification Centre
**PIL**	Participant Information Leaflet
**PROBE**	Prospective Randomised Open Blinded End-point
**QALY**	Quality-Adjusted Life Year
**QoL**	Quality of Life
**R&D**	Research and Development
**RCT**	Randomised Controlled Trial
**REC**	Research Ethics Committee
**SAE**	Serious Adverse Event
**SAR**	Serious Adverse Reaction
**SD**	Standard Deviation
**SmPC**	Summary of Product Characteristics
**SUSAR**	Suspected Unexpected Serious Adverse Reaction
**TAPSE**	Tricuspid Annular Plane Systolic Excursion
**TDI**	Tissue Doppler Imaging
**TMF**	Trial Master File
**TMG**	Trial Management Group
**TSC**	Trial Steering Committee
**UHB**	University Hospitals Birmingham
**WTCRF**	NIHR Wellcome Trust Clinical Research Facility at Queen Elizabeth Hospital, Birmingham

1 Trial Summary

**Title**	**Evaluating different rate control therapies in permanent atrial fibrillation: A prospective, randomised, open-label, blinded endpoint trial comparing digoxin and beta-blockers as initial rate control therapy**<br><br>**RAte control Therapy Evaluation in Atrial Fibrillation: RATE-AF**
**Acronym**	**RATE-AF**
**Trial Design and Methods**	A prospective, randomised, open-label, blinded-endpoint (PROBE) trial design. The RATE-AF trial combines hypothesis testing (quality of life, cardiac function, exercise capacity and biomarkers), evaluation of measures (validity, reproducibility and correlation of outcomes) and a feasibility study for a future clinical event trial (assessing recruitment, retention and sample size).
**Trial Medications**	Digoxin 62.5 – 250 µg od<br>Bisoprolol 1.25 – 15 mg od
**Trial Outcomes**	**Primary Outcome:**
	Patient-reported quality of life (QoL): SF-36 physical component summary score at six months
	**Secondary Outcomes:**
	Patient-reported QoL:<br>• SF-36 global and domain-specific scores at 6 and 12 months<br>• EQ-5D-5L summary index and visual analogue scale at six and twelve months<br>• AFEQT overall score at six and twelve months
	Cardiac function:<br>• Echocardiographic LVEF at 12 months<br>• Diastolic function (E/e' and composite of diastolic indices) at 12 months
	Functional assessment:<br>• Six-minute walking distance at 6 and 12 months<br>• Change in European Heart Rhythm Association (EHRA) class at 6 and 12 months
	Biomarkers:<br>• Change in B-type natriuretic peptide (BNP) levels at 6 months
	Change in heart rate using 24-hour ambulatory ECG
	**Feasibility Outcomes:**
	Recruitment target of 3 patients per week across all participating centres.
	Compliance and reasons for non-compliance
	Number of withdrawals and losses to follow-up (with reasons)
	Drug discontinuation rate and adverse reactions requiring drug discontinuation.
	Number of patients needing therapy-induced requirement for additional treatment

	Population-specific standard deviations (SD) and proportions:<br>• SD of SF36 physical functioning score at 6 and 12 months<br>• SD of SF36 overall score at 6 and 12 months<br>• SD of AFEQT overall score at 6 and 12 months<br>• SD of LVEF and E/e' scores at 6 and 12 months<br>• Unplanned hospitalisation admissions rates
	Cardiovascular Events (particularly mortality, thromboembolic events, myocardial infarction and cardiovascular interventions)
**Trial Duration per Participant**	12 months of trial therapy
**Planned Trial Sites**	Multiple screening sites with single site recruitment
**Total Number of Participants**	160
**Main Inclusion/ Exclusion Criteria**	**Inclusion Criteria**
	Adult patients, aged 60 years or older
	Permanent AF, characterised (at time of randomisation) as a physician decision for rate-control with no plans for cardioversion, anti-arrhythmic medication, or ablation therapy
	Symptoms of breathlessness (New York Heart Association Class II or more)
	Able to provide written, informed consent
	**Exclusion Criteria**
	Established indication for beta-blocker therapy, e.g. myocardial infarction in the last 6 months
	Known contraindications for therapy with beta-blockers or digoxin, e.g. a history of severe bronchospasm that would preclude use of beta-blockers, or known intolerance to these medications
	Baseline heart rate <60 bpm
	History of second or third-degree heart block
	Supraventricular arrhythmias associated with accessory conducting pathways (e.g. Wolff-Parkinson-White syndrome) or a history of ventricular tachycardia or fibrillation
	Planned pacemaker implantation (including cardiac resynchronisation therapy), pacemaker-dependent rhythm or history of atrioventricular node ablation
	Decompensated heart failure (evidenced by need for intravenous inotropes, vasodilators or diuretics) within 14 days prior to randomisation
	A current diagnosis of obstructive hypertrophic cardiomyopathy, myocarditis or constrictive pericarditis
	Received or on waiting list for heart transplantation
	Receiving renal replacement therapy
	Major surgery, including thoracic or cardiac surgery, within 3 months of randomisation
	Severe, concomitant non-cardiovascular disease (including malignancy) that is expected to reduce life expectancy

1.1 Trial Schema

Figure 1

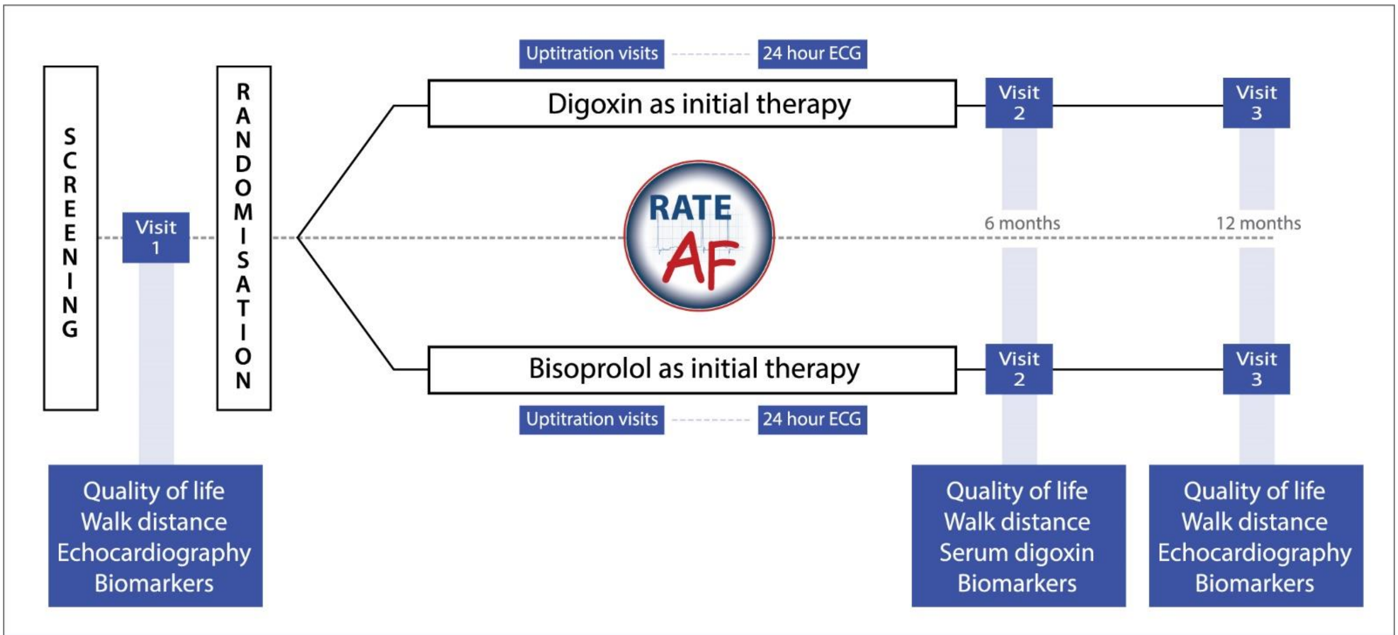

Inclusion Criteria:

- Age 60 years or older
- Permanent AF, characterised as a physician decision for rate control only
- Breathless; New York Heart Association Class II or above
- Able to provide written informed consent

Exclusion criteria:

- Established indication for beta-blocker therapy (e.g. myocardial infarction in the last 6 months)
- Known contraindication for beta-blockers or digoxin (e.g. history of severe bronchospasm or known intolerance)
- Baseline heart rate <60 bpm, 2nd/3rd degree block, accessory pathway or history of ventricular arrhythmia
- Planned pacemaker implantation, pacemaker-dependent rhythm or previous atrioventricular node ablation
- Decompensated heart failure within 14 days requiring intravenous inotropes, vasodilators or diuretics
- Hypertrophic cardiomyopathy, myo/pericarditis or previous/planned heart transplant
- Receiving renal replacement therapy
- Major surgery within 3 months or severe concomitant disease expected to reduce life expectancy

This protocol describes the **RATE-AF** trial only. The trial will be conducted in accordance with the protocol, Good Clinical Practice (GCP) and applicable regulatory requirements. Every care has been taken in the drafting of this protocol, but future amendments may be necessary, which will receive the required approvals prior to implementation.

2 Introduction

2.1 Background

Atrial fibrillation (AF) is an increasingly common cardiac condition that leads to a substantial burden on quality-of-life (QoL), an increased risk of cardiovascular events, hospitalisation and death, and significant healthcare costs for the NHS. In addition to anticoagulation and considerations for rhythm control therapy, most patients with AF are in need of pharmacological control of heart rate. This aspect of care has not received stringent investigation, with treatment guidelines based on small crossover studies and observational data rather than robust controlled trials.[1-3] Beta-blocker monotherapy remains the first-line option in the current NICE AF guidelines consultation document, with digoxin only for sedentary patients, although this recommendation is based on 'very low-quality evidence'.[4] The benefit of different rate-control therapies on symptoms and other intermediate outcomes (such as left-ventricular ejection fraction [LVEF] and diastolic function) are unknown, as are their effects on clinical events such as hospitalisation. This situation is unacceptable in light of the potential benefits and risk of different rate-control options in AF. It also limits our ability to personalise treatment according to patient characteristics.

The RAte control Therapy Evaluation in Atrial Fibrillation (**RATE-AF**) trial is informed by a number of in-depth systematic reviews of management and clinical outcomes in AF patients.[5-11] Taken together, this information provides a sound basis to plan a major randomised controlled trial (RCT).[12, 13] However as trials of rate-control in AF have typically been small or uncontrolled, further information is needed before designing a trial that can assess clinical outcomes. The **RATE-AF** trial will allow us to define appropriate primary and secondary outcome measures and their standard deviation in a contemporary population of patients with permanent AF. This information will allow us to estimate sample size, determination of recruitment, retention and adherence policies, and to ascertain the best methods of obtaining adverse event data and reliable economic costs for a larger trial assessing cardiovascular outcomes and hospitalisation. The **RATE-AF** trial will also be the largest RCT of its kind, allowing us to compare the effect of beta-blockers and digoxin on QoL as initial rate-control therapy in patients with permanent AF. The long-term aim of the research is to answer key questions about how to initiate therapy, stratified by relevant patient characteristics such as systolic and diastolic cardiac function, baseline symptoms and concurrent medication. The research will also define the patho-physiological mechanisms underlying AF-related symptoms, left-ventricular function and their association with adverse clinical outcomes, and to identify clinical markers for the response to different rate control therapy.

2.2 Epidemiology and Consequences of AF

AF is a common condition that is associated with increased rates of mortality and serious morbidity, including stroke, worsening of heart failure, sudden death, and reduced QoL.[1] The prevalence of AF increases with age, ranging from 0.7% in those aged 55–59 years to 17.8% in those aged above 85.[14] A doubling of both incidence and prevalence of AF is predicted in the next 20 years.[15]

Patients with AF are twice as likely to be hospitalised as propensity score-matched controls, with direct medical costs estimated to be 73% higher.[16] Further, AF is an independent predictor of all-cause mortality, with a two-fold adjusted increase in death.[17, 18] While most strokes in AF can be prevented by oral anticoagulation, AF patients still have high cardiovascular death rates due to sudden death or progressive heart failure.[19, 20] Patients with AF also have significantly poorer QoL[21], experiencing a variety of symptoms including lethargy, palpitations, dyspnoea, sleeping difficulties and psychosocial distress.[22, 23] In the context of patients diagnosed with heart failure, the presence of AF leads to higher rates of death and hospitalisation, independent of other risk variables or which condition comes first.[24, 25] From observational data, 40% of AF patients will be diagnosed with heart failure and vice-versa[16], representing a large and growing unmet clinical need for healthcare improvement.

2.3 Rhythm-Control in AF

Numerous large RCTs comparing rhythm-control (using arrhythmic drugs and/or cardioversion) versus rate-control have identified no significant difference in clinical outcomes in patients with persistent AF.[26-30] In a number of studies, hospitalisation rates were actually higher in those randomised to rhythm-control.[26, 29, 30] Similar findings have been shown in AF patients with heart failure[31, 32], both in those with impaired and preserved ejection fraction.[33-35] Although AF ablation is becoming increasingly popular to restore sinus rhythm, it remains a highly invasive method to improve AF-related symptoms.[36, 37] At present, European and NICE treatment guidelines recommend ablation only in symptomatic paroxysmal AF, or as a treatment option in symptomatic persistent AF that is refractory to other therapy.[3] Further trials are currently underway to determine the clinical value of prompt rhythm-control, including the Early treatment of Atrial fibrillation for Stroke prevention Trial (EAST).[38] In light of the high recurrence rate of AF (even in patients receiving intensive rhythm-control therapy), rate-control is an important part of AF management in almost all patients. Unfortunately, rate-control therapy has much less evidence underpinning its use.

2.4 Lack of Evidence to Guide Rate-Control Therapy

Rate-control in AF can be achieved with beta-blockers, non-dihydropyridine calcium-channel blockers (CCB), digoxin and their combinations. Unfortunately, little data exists to assist clinicians in choosing appropriate first-line and subsequent therapy. Current patterns of medication usage vary considerably (between and within countries). For example, in a worldwide registry, digoxin was prescribed in 2877 of 10,523 patients (27.3%), compared to 1599 of 3141 (50.9%) of patients in the German Competence NETwork on Atrial Fibrillation (AFNET).[39, 40]

Current European guidelines suggest “the choice of medication should be individualised and the dose modulated to avoid bradycardia”. This recommendation (Class 1, Level B) is based on a systematic review of trials addressing rate-control between 1983 and 1997.[41] Most of the studies included less than 50 participants (with several less than 10). The majority were low quality studies, as assessed by the risk of bias or confounding, and follow-up was typically in the order of

hours, days or weeks. Whilst this may be sufficient to assess an acute effect on heart-rate, it provides limited data on the longer-term effects of different treatments or the frequency of adverse reactions.

Beta-blockers are often preferred over other agents due to the prognostic benefit seen in patients with heart failure who are in sinus rhythm. However, in patients with heart failure, reduced LVEF and concomitant AF, we have shown that beta-blockers do not reduce mortality (hazard ratio 0·97, 95% CI 0.83-1.14; p=0.73) or cardiovascular hospital admissions (hazard ratio 0·91; 95% CI 0.79-1.04; p=0.15).[5] This distinctly contrasts with the significant benefit seen in patients with sinus rhythm and highlights the need for further comparative RCTs specifically in patients with AF.

The most highly cited trial comparing beta-blockers and digoxin for rate-control in chronic AF was an open-label two-week crossover study of 5 drug regimes in 12 patients.[42] Peak heart-rate after exercise was significantly higher in those taking digoxin compared to beta-blockers but there were no differences in exercise duration. In a trial of 42 patients, rate-control was improved with combination beta-blocker/digoxin therapy compared to digoxin alone, however there were similarly no differences in exercise capacity.[43] Systematic review of other small randomised studies identify no difference in exercise tolerance with beta-blockers, despite a lowering of heart-rate.[44] From observational data, such as the Atrial Fibrillation Follow-up Investigation of Rhythm Management (AFFIRM) study, more cardiac and non-cardiac adverse effects have been noted with beta-blockers than digoxin (n=67 vs. n=38).[28] In a 3-week crossover study of 60 participants, 10% withdrew during beta-blocker therapy due to adverse events.[45] Those in the beta-blocker group had a reduction in exercise capacity on cardio-pulmonary testing and a significant increase in B-type natriuretic peptide (BNP, a marker of ventricular strain) compared to patients treated with calcium-channel blockers.[46]

Only a single RCT has been published comparing digoxin and beta-blockers in patients with AF and heart failure (mean LVEF 24%, n=47).[47] Although there was a marginally-significant improvement in LVEF with carvedilol/digoxin versus placebo/digoxin, blinded withdrawal of digoxin then led to a deterioration in LVEF, accompanied by an increase in BNP. There was no difference in the number of heart-rate pauses >3 seconds or in daytime/exercise heart-rate comparing the two therapies alone.

Digoxin itself has been associated with an increased mortality in observational cohorts of AF patients[48], however careful adjustment of baseline differences reject a true excess in adverse outcomes.[49-51] In a detailed systematic review of all studies published on digoxin, we identified that confounding was the main reason that digoxin was associated with increased mortality in observational studies, and confirmed a neutral association in RCTs (risk ratio 0.99, 95% CI 0.93 to 1.05).[6] Lower rates of hospitalisation have been noted with digoxin therapy, independent of the type of heart failure[52], however the lack of randomised data versus placebo (despite widespread clinical use) makes true comparison difficult. Small RCTs comparing CCB with digoxin have been inconsistent; two have identified lower heart-rates with CCB but no significant difference in exercise capacity[42, 43], one demonstrated higher post-exercise cardiac output after

digoxin[53] and another showed improved exercise duration and QoL with CCB.[54] These results highlight the need for randomised data with appropriately-defined outcomes to accurately identify the benefits and risks of common therapies in patients with AF.

An example where RCT data have impacted on clinical practice is the Rate Control Efficacy in Permanent Atrial Fibrillation (RACE II) trial. This study challenged conventional wisdom that stricter control of heart-rate would allow time for diastolic filling and improve haemodynamics. In summary, 614 patients with permanent AF were randomised to strict or lenient rate-control and followed for 2-3 years.[55] There was no significant difference in the cumulative incidence of the composite primary outcome; 14.9% in the strict-control arm and 12.9% in the lenient-control group. There were also no differences in symptoms, New York Heart Association (NYHA) class or hospitalisations[55, 56], no interaction with baseline heart failure[57], and those participants achieving strict rate-control required more clinic visits and higher doses of medical therapy.[58] Current guidelines therefore suggest that lenient rate-control is acceptable, except for patients with adverse symptoms or clinical deterioration.[1] Whilst this study provides important data on the intensity of rate-control in AF, the more clinically-relevant questions of how to initiate therapy and the choice of optimal agents for individual patients remain unanswered.

2.5 Patient Wellbeing

Patient-reported outcomes are any report of a patient's health status (for example QoL) that is derived directly from the patient, without interpretation by a clinician.[59] There is limited data on the effect of pharmacological rate-control therapy on QoL and no comparative data assessing the benefit of different strategies.[22, 60] Rate-control has been associated with improved QoL scores in trials assessing rate versus rhythm-control.[61, 62] In the PIAF study, over 50% of participants randomised to calcium-channel blockers reported an improvement in health with significant benefits in the physical aspects of the SF-36.[63] A number of smaller studies have shown inconsistent effects on QoL in AF, although the data is limited by inclusion of patients with paroxysmal AF, a focus on heart rate and the use of a variety of QoL tools.

Current QoL questionnaires can be divided into disease-specific evaluations or generic health assessments (such as the Short Form Health Survey SF-36[64] or the EuroQol EQ-5D[65, 66]). However there is a distinct lack of knowledge regarding the mechanisms that underpin AF-related symptoms, the responsiveness of QoL questionnaires and their validity.[60] The Atrial Fibrillation Effect on QualiTy-of-life (AFEQT) questionnaire was designed to address these disparities by using more robust methods.[67] Although there is limited clinical application to-date, AFEQT has demonstrated sensitivity to clinical change.[68] An important objective of the research is to ascertain appropriate and responsive QoL tools for this population, as well as determine the acceptability and delivery of the questionnaires to patients.

2.6 Rationale for the RATE-AF Trial

Rate-control is an integral part of management in all AF patients but hardly any controlled trial evidence exists to guide the choice of agents. We have shown that neither beta-blockers nor

digoxin has an impact on mortality in AF patients, even with concomitant heart failure, which highlights the need to determine treatment effects on quality of life and cardiac function.

3 Trial Design and Objectives

RATE-AF is Prospective, Randomised Open-label Blinded Endpoint (PROBE) clinical trial comparing the use of digoxin and beta-blockers as initial rate control therapy.

In this section, we discuss the trial design and study objectives. Detailed outcome measures are listed in **Section 12**.

Justification for a PROBE rather than a Double Blind Trial Design

Although a double blind design would be the most robust trial design with respect to bias, it would not be ethical to do so in this scenario as clinicians would feel the need to add therapy according to heart rate. In addition, the RATE-AF Trial aims to test a strategy of initial care. PROBE trial design maintains the benefits associated with a strict randomisation procedure, while the blinded end points help to eliminate bias.

The trial design aims for a pragmatic 'all-comers' approach, applicable to those seen in clinical practice to allow transfer of the findings to routine clinical management of patients with permanent AF.

Assessment and Management of Risk

This trial is categorised by the Medicines and Healthcare products Regulatory Agency (MHRA) as:

Type A = No higher than the risk of standard medical care

The assessment and management of risk is detailed in the separate **RATE-AF** Risk Assessment document. An on-going evaluation of risk will continue throughout the recruitment period.

3.1 Hypothesis

Null Hypothesis for primary outcome:

No difference in patient-reported quality of life (measured using the physical functioning domain of the SF36 questionnaire) when comparing a strategy of digoxin versus beta-blocker therapy for initial rate control in patients with permanent AF.

Alternative Hypothesis:

Use of digoxin or beta-blocker therapy as initial rate control in patients with permanent AF is superior based on patient reported quality of life (measured using the physical functioning domain of the SF36 questionnaire).

3.2 Primary objective

- Patient-reported quality of life (QoL), with a predefined focus on physical well-being using the SF-36 physical component summary at six months.

3.3 Secondary objectives

- Generic and AF-specific patient-reported QoL using the SF-36 global and domain-specific scores, the AFEQT overall score and the EQ-5D-5L summary index and visual analogue scale at six and twelve months.
- Echocardiographic left-ventricular ejection fraction (LVEF) and diastolic function (E/e' and composite of diastolic indices) at twelve months.
- Functional assessment, including 6-minute walking distance achieved, change in European Heart Rhythm Association (EHRA) class and cognitive function at six and twelve months.
- Change in B-type natriuretic peptide (BNP) levels as a surrogate for total cardiac strain at six months.
- Change in heart rate from baseline and group comparison using 24-hour ambulatory ECG.

3.4 Feasibility objectives

- Successful methods for recruitment
- Key issues that affect retention of participants, such as convenience, compliance and cross-over (target of 85% study completion rate).
- Drug discontinuation rate and adverse reactions leading to drug discontinuation.
- Therapy-induced requirement for additional treatment (e.g. pacemaker implantation).
- Population-specific standard deviations and proportions to enable sample size calculation for a future trial.
- Assessment of cardiovascular outcomes including a composite of adverse clinical events (mortality, thromboembolic events, myocardial infarction and cardiovascular interventions).

3.5 Exploratory objectives

- Correlation of baseline measures, including QoL questionnaires and unblinded baseline investigations such as QoL, BNP, LVEF, E/e', EHRA, intracellular methods and heart rate.
- Impact of therapy on intracellular sodium and calcium concentration and cardiotonic steroid levels as biomarkers of cellular response at six and twelve months.

- Impact of combination therapy on outcomes, including comparison of bisoprolol/non-dihydropyridine calcium channel blocker (CCB) vs. bisoprolol/digoxin vs. digoxin/CCB vs. single therapies.
- Change in cognitive function at twelve months
- Qualitative research of patient-reported QoL using focus groups to explore patient acceptability, optimal delivery methods and responsiveness.
- Assessment of the validity and reproducibility of echocardiographic measures in patients with AF.
- Correlation of serum digoxin concentration with change in QoL and intracellular methods.
- Cost-consequence economic analysis from an NHS perspective.

4 Selection of Participants

Participants who potentially fulfil the inclusion criteria for this trial must have their eligibility confirmed by medically qualified personnel with access to and a full understanding of the potential participant's medical history. If eligibility has been assessed and documented by medically qualified personnel, then the process of obtaining informed consent may be delegated as appropriate and as documented on the **RATE-AF** Delegation and Signature Log.

4.1 Inclusion Criteria

- Adult patients aged 60 years or older
- Permanent AF, characterised (at time of randomisation) as a physician decision for rate-control with no plans for cardioversion, anti-arrhythmic medication, or ablation therapy
- Symptoms of breathlessness (New York Heart Association Class II or more)
- Able to provide written informed consent

4.2 Exclusion Criteria

- Established clinical indication for beta-blocker therapy, e.g. myocardial infarction in the last 6 months
- Known contraindications for therapy with beta-blockers or digoxin, e.g. a history of severe bronchospasm that would preclude use of beta-blockers, or known intolerance to these medications
- Baseline heart rate <60 bpm
- History of second or third-degree heart block
- Supraventricular arrhythmias associated with accessory conducting pathways (e.g. Wolff-Parkinson-White syndrome) or a history of ventricular tachycardia or fibrillation

- Planned pacemaker implantation (including cardiac resynchronisation therapy), pacemaker-dependent rhythm or history of atrioventricular node ablation
- Decompensated heart failure (evidenced by need for intravenous inotropes, vasodilators or diuretics) within 14 days prior to randomisation
- A current diagnosis of obstructive hypertrophic cardiomyopathy, myocarditis or constrictive pericarditis
- Received or on waiting list for heart transplantation
- Receiving renal replacement therapy
- Major surgery, including thoracic or cardiac surgery, within 3 months of randomisation
- Severe, concomitant non-cardiovascular disease (including malignancy) that is expected to reduce life expectancy

5 Informed Consent Process

It will be the responsibility of the Investigator to obtain written informed consent for each participant prior to performing any trial related procedure. If local practice allows, this responsibility may be delegated by the Principal Investigator, to a Research Nurse as captured on the Site Signature and Delegation Log. A Participant Information Leaflet (PIL) will be provided to facilitate this process. Investigators or delegate(s) will ensure that they adequately explain the aim, trial treatment, anticipated benefits and potential hazards of taking part in the trial to the participant. They will also stress that participation is voluntary and that the participant is free to refuse to take part and may withdraw from the trial at any time. The participant will be given adequate time to read the PIL and to discuss their participation with others outside of the site research team. The participant will be given the opportunity to ask questions.

If the participant expresses an interest in participating in the trial they will be asked to sign and date the latest version of the Informed Consent Form (ICF). The participant must give explicit consent for the regulatory authorities, members of the research team and representatives of the sponsor to be given direct access to the participant's medical records.

The Investigator or delegate(s) will then sign and date the form. A copy of the ICF will be given to the participant, a copy will be filed in the medical notes, and the original placed in the Investigator Site File (ISF). Once the participant is entered into the trial, the participant's unique trial identification number will be entered on the ICF maintained in the ISF. As part of the consent process, the participant will be asked to give explicit consent to their trial-related information being sent to the Trials Office at the University of Birmingham.

This trial will include **optional consent** to allow linkage to patient data available in NHS routine clinical datasets, including primary care data (e.g. Clinical Practice Research Datalink; CPRD, The Health Improvement Network; THIN, QResearch), secondary care data (Hospital Episode Statistics; HES) and mortality data from the Office of National Statistics (ONS) through The Health and Social Care Information Centre and other central UK NHS bodies. The consent will

also allow access to other new central UK NHS databases that will appear in the future. This will allow us to double check the main outcomes against routine data sources, and extend the follow-up of patients in the trial and collect long-term outcome and health resource usage data without needing further contact with the trial participants. This is important as it will link a trial of treatments that may become a clinical standard of care to long-term outcomes that are routinely collected in clinical data but which may be collected during the follow-up period of the trial.

Details of the informed consent discussions will be recorded in the participant's medical notes. This will include date of discussion, the name of the trial, summary of discussion, version number of the PIL given to participant and version number of ICF signed and date consent received. Where consent is obtained on the same day that the trial related assessments are due to start, a note will be made in the medical notes as to what time the consent was obtained and what time the procedures started.

At each visit the participant's willingness to continue in the trial will be ascertained and documented in the medical notes. Throughout the trial the participant will have the opportunity to ask questions about the trial. Any new information that may be relevant to the participant's continued participation will be provided. Where new information becomes available which may affect the participants' decision to continue, participants will be given time to consider and if happy to continue will be re-consented. Re-consent will be documented in the medical notes. The participant's right to withdraw from the trial will remain.

Electronic copies of the PIL and ICF will be available from the Trials Office and will be presented on the headed paper of the local institution. Details of all participants approached about the trial will be recorded on the Participant Screening/Enrolment Log and with the participant's prior consent, their General Practitioner (GP) will also be informed that they are taking part in the trial.

6 Enrolment and Randomisation

A flowchart of the recruitment process is shown in the Trial Schema (**Figure 1**) together with the schedule of investigation. **Section 9** gives more detailed information of trial procedures and assessments.

In the majority, potentially eligible participants will be identified by their Cardiologist, usually following referral from their General Practitioner (GP), and provided with an ethically-approved patient information leaflet (PIL). The patient will then be invited to attend a baseline visit at the NIHR Wellcome Trust Clinical Research Facility (WTCRF) at Queen Elizabeth Hospital, Birmingham. Potentially eligible participants may also be identified from inpatient referrals; these patients will be provided with a PIL and invited to attend a baseline visit following the same procedure.

GP Practices in the Birmingham area may be asked to refer patients that present with AF, but are not on medication, to the RATE-AF Research Team at University Hospitals Birmingham (UHB). These patients will be given a one-page, ethics committee-approved trial summary and asked to

sign a contact details form to confirm that they are happy to be contacted by a member of the Research Team to arrange an appointment.

Prior to patients undertaking any trial-related procedures, informed consent will be obtained.

Details of all patients approached about the trial should be recorded on the **RATE-AF** Screening & Enrolment Log. This Log should be maintained within the Investigator Site File.

6.1 Randomisation Procedures

After all eligibility criteria have been confirmed and informed consent has been received, the participants can be randomised into the **RATE-AF** trial.

Participants will be randomised in a 1:1 ratio to either **Digoxin 62.5 – 250 μg od or Bisoprolol 1.25 – 15 mg od**. The time between randomisation and commencement of trial therapy should be minimised (ideally <24 hours). Randomisation will be provided by a computer generated programme at the Birmingham Clinical Trials Unit (BCTU), using a minimisation algorithm to ensure balance between the arms with regard to important clinical variables, stratifying for baseline EHRA (class 1/2a and 2b/3/4) and gender.

Telephone and Online Randomisation

Participants can be randomised into the trial via a secure 24 hour internet based randomisation service (**https://www.trials.bham.ac.uk/RATEAF**) or by a telephone call to the BCTU (telephone number **0800 953 0274**). Telephone randomisations are available Monday-Friday, 09:00-17:00. For the secure internet randomisation, each site and each randomiser will be provided with a unique log-in username and password in order to access the online system. Online randomisation is available 24 hours a day, 7 days a week, apart from short periods of scheduled maintenance and occasional network problems.

Randomisation Forms will be provided to investigators and should be completed and used to collate the necessary information prior to randomisation. Once all eligibility criteria have been provided and confirmed, a Trial Number and treatment allocation be given and relevant parties notified, including the participant's GP.

Back-up Randomisation

If the internet based randomisation service is unavailable for an extended period of time, a back-up paper randomisation will also be available at the BCTU. The randomisation list will be produced using a random length block design. In this instance, investigators should ring the BCTU randomisation service (telephone number **0800 953 0274**).

7 Trial Treatment

7.1 Treatment

The Investigational Medicinal Products (IMPs) for this trial are Digoxin and Bisoprolol.

At randomisation, participants will be allocated to open-label treatment with either Digoxin 62.5 – 250 µg od or Bisoprolol 1.25 – 15 mg od.

Digoxin

Digoxin is a cardiac glycoside derived from the foxglove plant. The cardiac effects of digoxin therapy are summarised by:

- Positive inotropic effects: increased intracellular calcium due to direct inhibition of sodium-potassium adenosine triphosphatase (Na/K-ATPase)
- Negative chronotropic effects: decreased conduction velocity through the atrioventricular node, an increase in the effective refractory period and an increase in vagal activity leading to sinus node depression.

Clinically, digoxin is commonly prescribed in two conditions, heart failure and AF.

Bisoprolol

Bisoprolol fumarate is a highly beta-1 selective adrenoreceptor blocker first approved by the U.S. Food and Drug Administration in 1992. The cardiac effects of bisoprolol therapy are summarised by:

- Negative chronotropic effects: a reduction in resting and exercise heart rate due to prevention of norepinephrine and epinephrine from binding to the beta-receptor in cardiac conduction tissue.
- Negative (mild) inotropic effects: an initial fall in resting and exercise cardiac output with little observed change in stroke volume and only a small increase in right atrial pressure or pulmonary capillary wedge pressure.

Clinically, bisoprolol is commonly prescribed in a range of cardiology conditions, including post-myocardial infarction, heart failure and in patients with atrial tachyarrhythmia, including AF.

7.2 Treatment Supply and Storage

Due to the participant population and the fact that the trial closely aligns with standard care, trial medication may be dispensed from routine standard stock by both the pharmacy at the research site and community pharmacies local to the participant. Both treatments are used as per normal clinical practice therefore there is no additional requirement, above that of local policy, to monitor temperature during storage.

Digoxin

Digoxin is available as an oral tablet in doses of 62.5, 125 and 250 µg or as an elixir (50 µg/mL). It is packaged in 28 or 500 tablet packs under the generic title digoxin and trade label Lanoxin.[69] Digoxin should be stored according to local policy.

Bisoprolol

Bisoprolol is available as an oral tablet in doses of 1.25, 2.5, 3.75, 5.0, 7.5 and 10 mg. It is packaged as 28 tablets under the generic title bisoprolol fumarate and trade labels Cardicor and Emcor.[69] Bisoprolol should be stored according to local policy.

7.3 Dosing Schedule

Digoxin

An advice sheet for the investigator is presented in **Appendix A.**

Trial maintenance doses will initially be 62.5 or 125 µg orally (at the clinician's discretion, taking into account age and renal function), with planned up-titration to 125/250 µg. The maximum trial dose will be 250 µg daily.

A single loading dose of four tablets (250 or 500 µg according to target maintenance dose) will be prescribed in digoxin-naïve participants. The clinician is permitted to omit the loading dose or prescribe a second, where necessary.

Unblinded serum digoxin concentrations will be assessed at visits 2 and 3, with results reported back to the relevant clinician(s). This process will assist in monitoring compliance, adjusting dosage in cases of low serum levels and avoiding toxicity.

Bisoprolol

An advice sheet for the investigator is presented in **Appendix B**

Trial starting doses will be 1.25 or 2.5 or 5 mg (at the clinician's discretion), with planned up-titration to 10 mg in increments of 1.25 or 2.5 mg. The maximum trial dose will be 15 mg daily. No loading dose is required.

Plasma concentrations have not shown to be associated with toxicity and are not part of standard clinical practice.

7.4 Drug Interactions and Contraindications

Digoxin

Following oral administration of digoxin, approximately 60–85% of the dose is usually absorbed, mainly from the small intestine. The onset of action is 0.5-2 hours and maximal effects occur in

2-6 hours. Digoxin has a large volume of distribution and approximately 20-30% of digoxin in blood is bound to plasma proteins. Metabolism is minimal but variable, with the majority of drug excreted unchanged in the urine by glomerular filtration and tubular secretion. With normal renal function, the elimination half-life is 34-44 hours which is prolonged in patients with renal failure by two to threefold. Dose adjustment is unnecessary in patients with hepatic impairment. Therapeutic plasma concentrations of digoxin have been described as 0.5-2.0 ng/mL.[70] In digoxin-naïve patients with normal renal function, approximately seven days are required to reach steady-state therapeutic concentrations if a loading dose is omitted. As such, the majority of clinicians prescribe one or two loading doses, totalling 500 to 1000 μg over 24 hours.

Caution is recommended in patients with electrolyte disturbance (due to increased risk of toxicity) and reduced doses are recommended in patients with renal impairment. There are no concerns in pregnancy or with breast-feeding, although dose adjustment may be required.

Contraindications for digoxin therapy include heart block, accessory pathway supraventricular tachycardia and a current diagnosis of obstructive hypertrophic cardiomyopathy, myocarditis or constrictive pericarditis.

Digoxin has been associated with a number of adverse effects, although data from randomised trials show little difference in comparison to placebo, apart from cases of toxicity (2% versus 0.9% respectively in the DIG trial of patients with HF)[71]. The most common side effects are gastrointestinal upset, dizziness, blurred vision, headache and rash. In toxic states (serum levels >2 ng/mL), digoxin is pro-arrhythmic and can aggravate heart failure, particularly with co-existent hypokalaemia. In cases of overdose, repeated early doses of activated charcoal may be given to reduce absorption and in severe toxicity, digoxin-specific antibody fragments are available as an intravenous infusion.

In rigorous assessment, drug interactions with digoxin have proved inconsistent.[72] Serum digoxin concentrations are increased by amiodarone, dronedarone, propafenone and quinidine but increased bioavailability with CCB and certain antibiotics (such as erythromycin and tetracycline) only occur in selected patients. The risk of toxicity increases with drugs that cause electrolyte disturbances, such as thiazide and loop diuretics.

Bisoprolol

Following oral administration of digoxin, the absolute bioavailability is approximately 80%, first pass metabolism of 20% and 30% protein binding. Peak plasma concentrations occur within 2-4 hours, the elimination half-life is 9-12 hours and steady state is attained within 5 days. Elimination occurs equally by renal and non-renal pathways with about 50% of the dose remaining unchanged in the urine.

Caution is recommended in patients with first-degree heart block, portal hypertension, diabetes, a history of obstructive airways disease, myasthenia gravis, a history of hypersensitivity and psoriasis, although many cardiologists use beta-blockers frequently in these groups with appropriate supervision. In pregnancy, beta-blockers may cause intra-uterine growth restriction,

neonatal hypoglycaemia, and bradycardia (although as above, these agents are frequently used in pregnancy). There is a theoretical risk of toxicity in breast feeding, although the amount present in milk is likely too small to affect infants. Abrupt withdrawal should be avoided, especially in cases of ischaemic heart disease. Up-titration should be more cautious in patients with renal or hepatic impairment.

Contraindications for bisoprolol therapy include cardiogenic shock, overt cardiac failure, second or third degree heart block, marked sinus bradycardia and severe peripheral arterial disease.

Bisoprolol has been associated with a wide variety of adverse effects although data from RCTs suggest similar discontinuation rates compare to placebo.[5, 73] The most common adverse symptoms are lethargy, headache, peripheral oedema, upper respiratory tract symptoms, gastrointestinal upset and dizziness. In cases of overdose, bradycardia, hypotension, congestive heart failure, bronchospasm and hypoglycaemia may be expected, with treatment directed to supportive methods and atropine, fluids, glucagon or diuretics as required.

Pharmacokinetic interactions with beta-blockers have not shown to be clinically significant. Drugs that reduce absorption include aluminium salts and cholestyramine, whilst metabolism can be increased by barbiturates and rifampicin and decreased with cimetidine, erythromycin, fluvoxamine, and hydralazine.

7.5 Accountability Procedures and Labelling

Through the risk-adapted approach, a full risk assessment of the **RATE-AF** trial has been conducted including the drug accountability requirements. The IMPs will be used within their authorisations, prescribed on an NHS prescription and dispensed by pharmacy from standard stock. The risk assessment has determined that a normal dispensing label is appropriate and an additional clinical trial label is not necessary (as covered by Regulation 46 (2) of SI 2004/1031). Drug accountability will be according to standard practice for NHS prescriptions. Details of how compliance will be assessed can be found in **Section 7.7**.

7.6 Treatment Modification

Patients that withdraw from medication for any reason will do so under strict clinical supervision.

The trial is designed to assess the impact of **initial** impact of rate control therapy; it is expected that treatments will modify during the trial period (in particular, the addition of therapy to attain heart rate targets). Patients will not be withdrawn from the trial if they commence therapy from the other arm of the trial due to any absolute or relative clinical indications (for example, patients in the digoxin arm starting beta-blockers due to incident myocardial infarction, or heart failure with reduced LVEF).

7.7 Assessment of Compliance

We will ask participants about compliance with their trial medication at each follow-up visit and this will be documented in the CRFs. It may also be clinically evident from the heart rate check, performed as part of all visits, whether or not the patient has been compliant with their trial mediation.

In addition, patients that are randomised to the digoxin arm will have a serum digoxin sample taken as part of Visit 2 (month 6) and Visit 3 (month 12) follow-ups. The results will indicate whether the patient has been compliant with their trial medication.

8 Trial Procedures and Schedule of Assessments

8.1 Baseline Visit

The baseline visit will occur as soon as possible after screening and will involve the following procedures (see **Section 9** for procedure details):

- Verify inclusion/exclusion criteria.
- Obtain written informed consent from the potential participant.
- Randomise the patient via telephone or the secure web-based system as outlined in **Section 6**
- Administer QoL and functional capacity questionnaires.
- Review recent blood results (within 6 months of Baseline Visit)
    - Assessing renal function to aid in dose assignment and serum potassium level as part of standard clinical care.
- Document the use of oral anticoagulation and arrange appropriate prescription for patients not on therapy according to clinical guidelines. If the participant is already receiving vitamin-K antagonists (VKA), recent INR results will be documented.
- Record results of physical examinations.
- Collect blood samples for baseline blood tests and biomarker analysis.
- Complete case report form (CRF)
- Perform a 12-lead electrocardiogram.
- Supervise a 6-minute walk test, recording distance walked and peak heart rate.
- Arrange the baseline echocardiogram; images will be delivered to the echocardiographic core laboratory for blinded reporting.
- Discuss the randomised allocation with the participant including schedule for drug therapy and up-titration.

Participants will be followed up by telephone call two weeks after the Baseline Visit to ensure they have commenced trial medication.

8.2 Up-Titration Visits

For the majority of participants, two up-titration visits will be planned to supervise the appropriate use of medications as per the up-titration schedule (see **Appendices A and B**). Additional up-titration visits, as required, are acceptable in order to attain a heart rate at rest of ≤100bpm.

Up-titration visits will involve the following procedures:

- Record adverse events as reported by the participant or observed by the investigator.
- Review of medications and plan for trial drug up-titration
- Assessment of compliance
- Symptom-directed clinical examination
- Vital signs, including heart rate and blood pressure
- Administer QoL and functional capacity questionnaires (last uptitration visit only).
- Organise a 24-hour ambulatory ECG once up-titration completed (results to be forwarded to the clinician).

8.3 Visit 2, Month 6

Visit 2 will occur at an interval of six months (± four weeks) after the Baseline Visit and involve the following procedures:

- Administer QoL and functional capacity questionnaires.
- Record adverse events as reported by the participant or observed by the investigator.
- Confirm current rate control therapy (including dosage) and check concomitant medications.
- Assessment of compliance.
- Collect blood samples for biomarker analysis.
- Collect blood sample for serum digoxin concentration, potassium and creatinine as part of standard clinical care.
- Record time in therapeutic range for patients on anticoagulation with vitamin-K antagonists and compliance in patients receiving non-VKA oral anticoagulants.
- Obtain a twelve lead ECG.
- Record the results of physical examinations and vital signs.
- Supervise a 6-minute walk test, recording distance walked and peak heart rate.
- Complete other CRF requirements.
- If an echocardiogram has been performed for clinical reasons since the previous visit, images will be retrieved and sent to the core echocardiographic laboratory.

- Confirm appointment date for Visit 3.

8.4 Visit 3, Month 12 (Final Trial Assessment)

Visit 3 will occur at an interval of 12 months (± four weeks) after the Baseline Visit and involve the following procedures:

- Administer QoL and functional capacity questionnaires.
- Record adverse events as reported by the participant or observed by the investigator.
- Confirm current rate control therapy (including dosage) and check concomitant medications.
- Assessment of compliance.
- Transthoracic echocardiography (as per **Section 9.6**), with images delivered to the echocardiographic core laboratory for blinded reporting.
- Collect blood sample for serum digoxin concentration, potassium and creatinine as part of standard clinical care.
- Record time in therapeutic range for patients on anticoagulation with vitamin-K antagonists and compliance in patients receiving non-VKA oral anticoagulants.
- Obtain a twelve lead ECG.
- Record the results of physical examinations and vital signs.
- Supervise a 6-minute walk test, recording distance walked and peak heart rate.
- Complete other CRF requirements.
- If an echocardiogram has been performed for clinical reasons since the previous visit, images will be retrieved and sent to the core echocardiographic laboratory.
- Complete the end of trial standardised letter to the GP and clinician explaining that the participant has reached the end of the trial protocol and is no longer bound by their allocated medication strategy. Advise that all participants are invited for continued follow up and long term clinical outcome assessment.
- Provide final instructions to participant (e.g. follow-up of ongoing adverse events).

8.5 Investigator-blinded Endpoints

Investigator-blinded endpoints (PROMs, echocardiography and biomarkers) will be assessed by the core laboratory, identified only by the trial number. Ambulatory ECG and serum digoxin level will remain unblinded and results delivered to the responsible clinician.

8.6 Long Term Follow-Up

In patients who have agreed to NHS data linkage, a follow-up CRF will be completed. The CRF will capture items that include, but are not limited to death, hospital admissions and

cardiovascular events. The planned interval for outcome assessment is 2 and 5 years post-enrolment.

8.7 Withdrawal

Participants may withdraw at any time during the main **RATE-AF** trial if they choose not to continue or if their clinical team feel that continued participation in the trial is inappropriate.

An investigator may deem it necessary to withdraw a participant from the trial if:

1) Any clinical adverse event, laboratory abnormality, or other medical condition or situation occurs such that continued participation in the trial would not be in the best interest of the participant.
2) The participant meets an exclusion criterion (either newly developed or not previously recognised) that precludes further trial participation.

Full details of the reason(s) for withdrawal should be recorded on the Case Report Forms (CRFs) if healthcare professional-initiated, otherwise a simple statement reflecting patient preference will suffice.

Clear distinction will be made between withdrawals from trial treatments whilst allowing further follow-up, and any participants who refuse any follow-up. If a participant explicitly withdraws consent to any further data recording, then this decision will be respected. All communications surrounding the withdrawal will be noted in the participant's records and no further data will be collected for the participant.

In the case of missing echocardiographic outcome data due to withdrawal (but with consent for ongoing follow-up) or death, results of recent clinical echocardiography will be retrieved. The participant's permission to obtain such data will be obtained and documented during the consent process. As with all trial echocardiograms, the scan will be reported by the core echocardiographic laboratory. With respect to patient-reported outcomes, QoL questionnaires will be mailed to participants who withdraw from trial treatment but consent to ongoing follow up. Those patients where adverse symptoms were related to withdrawal will be invited to a focus group for further discussion.

8.8 Trial Duration

Patients will be on trial medication for 12 months and will be followed-up, during this period according to the protocol. At the end of the 12 months, the participants may, as determined by their clinician, continue on medication but it will not be considered part of the trial intervention. The trial will cease when the 12-month follow-up has been completed for the last participant recruited.

Table 1: Schedule of Assessments

Procedures		Baseline Visit	Up-titration Visits (Day 14 to 60)	Visit 2, Month 6 (± 4 weeks)	Visit 3, Month 12 (± 4 weeks)
Assessment of eligibility criteria		X			
Informed consent taken		X			
Review of medical history		X			
Review of medications		X	X	X	X
Physical exam	Complete	X			
	Symptom-directed		X	X	X
	Vital signs	X	X	X	X
Quality of life assessment		X	(X)	X	X
Functional and cognitive assessment		X		X	X
Transthoracic echocardiogram		X			X
12-lead electrocardiogram		X		X	X
6-minute walk test		X		X	X
24-hour ambulatory ECG			X	(X)	
Clinical labs	Chemistry	X		X	X
	Haematology	X		X	X
	Serum digoxin			(X)	(X)
Trial labs	BNP	X		X	
	Stored sample	X		X	
Assessment of compliance			X	X	X
Assessment of adverse events			X	X	X

Parentheses denote where a procedure is dependent on the stage of participants within the trial.

9 Trial Procedures

9.1 Procedures Defined as Standard Clinical Care

The following assessments are considered part of the standard clinical care of AF patients receiving heart rate control therapy and will occur at all trial visits:

- Blood tests for haemoglobin, serum creatinine, potassium and serum digoxin concentration; these will be obtained by the research nurse and submitted to the site-specific hospital laboratory as per local guidelines and SOPs, ensuring that all specimens are accurately labelled and handled appropriately. In the case of results requiring urgent action, local policies will be followed which may include the participant visiting their GP, local hospital or investigator. In all cases, appropriate trial documentation will be completed.
- A 12-lead ECG; these will be completed by appropriately trained local staff.

9.2 Medical History

Medical history will be obtained by interview and from medical records (physical and electronic) at the Baseline Visit comprising:

- Cardiovascular history, including prior ischaemic coronary disease, interventions and surgery, history of hypertension, heart failure or hyperlipidaemia, stroke or transient ischaemic attack, pulmonary embolus/deep vein thrombosis and peripheral vascular disease.
- AF history, including year of diagnosis, previous cardioversions, previous ablation therapy and anti-arrhythmic drug history.
- Pacemaker history, including date and reason for implantation, type of device (single-chamber, dual-chamber, biventricular, implanted defibrillator) and dependency.
- Non-cardiac history, including diabetes mellitus, airways disease (asthma/COPD), renal impairment, bleeding history and other major co-morbidities.
- Social and demographic history, including smoking status (current/ex/never), race (Caucasian/Indian subcontinent/Asian/African/other) and physical activity using the International Physical Activity Questionnaire (short form).

9.3 Medication History

Medications history will be assessed according to the categories below and include current dosage. Except for anticoagulation, antiarrhythmic and rate control therapies, only current medications will be included.

- Anticoagulation therapy (vitamin-K antagonists and novel agents), including past use, INR results and time in therapeutic range.
- Antiarrhythmic therapy, including past use.

- Rate control therapy (beta-blockers, digoxin, CCB), including past use.
- Antiplatelet therapy.
- Angiotensin-converting enzyme inhibitors or angiotensin receptor blockers.
- Aldosterone antagonists.
- Diuretics (loop, thiazide, potassium-sparing, others).
- Nitrates.
- Other anti-hypertensive/anti-anginal therapy.
- Statins.
- Other lipid-lowering medication.
- Diabetic medication and insulin.
- Asthma/COPD medication (including inhalers).
- Non-steroidal anti-inflammatory agents.

9.4 Physical Examination

Physical and vital signs will be assessed at all up-titration and trial visits. In most cases, a targeted physical examination will be performed, comprising of cardiovascular elements as summarised below:

- Heart rate (manual palpation at radial artery and apex).
- Heart sounds.
- Lung auscultation.
- Assessment of jugular venous pressure and/or peripheral oedema.
- Other focused examinations according to symptoms and complaints.
- Blood pressure (two measurements at the right brachial in a seated position preferred, using a validated oscillometric device).
- Height (Baseline Visit only), weight (all listed visits) and waist circumference (Baseline Visit; defined as the narrowest point between ribs and hips when viewed from the front after exhaling to the nearest centimetre).

9.5 Patient Reported Outcomes

9.5.1 Choice of Outcomes and Qualitative Research

A systematic review (according to and in collaboration with the COnsensus-based Standards for the selection of health Measurement Instruments, COSMIN[74]) is underway to evaluate PROMs in AF, with a focus on psychometric properties including internal consistency, reliability, and measurement error. Additional assessment and practical evaluation of PROMs will follow published guidance[75, 76], complementing qualitative research using patient focus groups, surveys

and directed interviews guided by the PROMs and qualitative research centres at the University of Birmingham.[77]

Instruments for assessment will be selected on the basis of overall validity, preferably in this patient population but including other groups where data are limited. Patient focus groups will allow exploration of patient perspectives on appropriate instruments that adequately reflect the experience of living with AF.[78] They will also allow comparison of QoL questionnaires that adequately summarise patient-prioritised components of their health and well-being. Additional focus groups and individual interviews will occur at interim and final follow-up during the trial. These aim to understand the patient experience of trial participation and processes, including the ease of completion of QoL questionnaires, relevance, reasons for non-completion and other feasibility issues that emerge during the trial e.g. non-compliance and recruitment, with reference to core outcome sets for this population.[79] A patient and public involvement (PPI) panel will contribute to all stages in the focus group process.[80]

This protocol was developed in accordance with the Standard Protocol Items for Randomized Trials [SPIRIT] statement[81], and the latest PROM-specific guidance from the International Society for Quality of Life Research (ISOQOL) Best Practice taskforce.[77, 82, 83]

9.5.2 Data Collection for PROMs

PROMs will be assessed at all main visits (Baseline, 2 and 3) and at the participants final up-titration visit (if applicable). The QoL tools used will be EQ-5D-5L, SF-36 and AFEQT. To avoid introducing co-intervention bias, all QoL data will be kept confidential and will not be used to inform clinical care.[84] Patients will be advised of this in the patient information sheet. PROMs will be collected at the start of each visit, before other trial procedures. In cases where the visit coincides with a clinician review, questionnaires should be completed in advance. The feasibility of using an online data collection tool will be explored, administered by trained research nurses and according to good-practice guidelines.[85] We will use this trial to perform an initial small-group assessment of electronic PROMs-equivalence to inform a future clinical event trial.

Qualitative research will be performed using a focus group of 10 volunteer patients enrolled at the start of the trial (5 in each randomised group). The focus group will meet after up-titration and then at 6 and 12 months. Detailed methods will be established before the first meeting, in collaboration with the University of Birmingham Qualitative Research Group.

All staff will receive training in QoL collection, with specific guidance on reducing introduced bias, minimising missing data and coaching participants to use the QoL software. Levels of missing PROMs data will be monitored. The site personnel responsible for collection of patient reported outcomes will be the Research Nurse under the supervision of the Principal Investigator.

9.5.3 Outcome Appraisal

Each QoL tool will be scored according to their published requirements (www.euroqol.org; www.sf-36.org; www.afeqt.org), using total and sub-category scores where appropriate.

To avoid dilution of effect over time, the primary analysis will be at six months (adjusting for baseline QOL and stratification variables). We have predefined a focus on physical well-being, which we hypothesize are where the greatest treatment effects will be observed, but will explore all aspects of QoL. Exploratory analysis of medication effects over the 12-month period will also be analysed and remain clinically important, as little data currently exists on the longer-term profile of QoL in AF.

Qualitative research outcomes will focus on the clinical responsiveness of the QoL instruments. The findings of the COSMIN systematic report will determine these outcomes and their relevant appraisal.

The RATE-AF trial will allow us to gain an initial understanding and framework of the patient experience of AF. We aim to begin the process of determining appropriate and responsive PROMs for AF patients and the optimum methods for delivery into a subsequent large-scale clinical trial.

9.6 Transthoracic Echocardiography

Echocardiography will be performed at Visits 1 and 3 and focus on systolic left-ventricular (LV) function, diastolic function and left-atrial assessment. Images will be obtained by an accredited echocardiographer. All trial echocardiograms will be labelled with the Trial Number and pseudoanonymised patient data, with specific instruction that the echocardiographer will remain blinded to the treatment assignment. All images will be archived to the core echocardiographic laboratory, with a copy retained in the site file.

9.6.1 Reproducibility and Validity of Measurements

Inter-observer and intra-observer variability in measurement will be assessed by comparing results of the stated methods discussed below across the cardiac cycle. To evaluate the minimum number of repeat measurements required that maintains clinical utility, reproducibility of single measurements will be compared to averages of 3/5/10 beats. This will also include the reliability of using an ‘index beat’ with a cycle length equivalent to a heart rate of 70-80 beats per minute, or with similar preceding and pre-preceding RR intervals.

9.6.2 Systolic LV Function

Systolic LV function will be determined by the following methods:

- Two-dimensional biplane Simpson’s method utilising the simultaneous multi-planar approach to obtain LVEF in a single heartbeat (four and two-chamber views). In each

view, LV end-diastolic and end-systolic volumes (LVEDV, LVESV) are computed, with LVEF calculated as (LVEDV – LVESV) / LVEDV. Two-dimensional echocardiography has excellent spatial resolution but is limited by potential foreshortening of the ventricular apex and drop-out of the endocardial border.

- Standard Simpson's biplane method with four and two-chamber volumes obtained from separate heartbeats. This is the conventional method in current clinical use but is limited by varying RR intervals in AF.
- Fractional shortening on M-mode along the minor-axis of the left-ventricle (parasternal long-axis), calculated by the formula: (LV internal dimension in diastole - LV internal dimension in systole) / LV internal dimension in diastole. M-mode measurements are reproducible and easy to perform with excellent temporal resolution, but are limited in cases of regional wall motion abnormalities and in patients where the true minor-axis is difficult to visualise.
- Both automated endocardial tracking and speckle-tracking analysis will be utilised (where available) by the echocardiographic core laboratory. Multiple planes will be obtained (four-chamber, two-chamber and short-axis mid-ventricle views). These methods have the advantage of reducing operator time and are angle-independent, but rely on good ultrasound windowing. Global longitudinal systolic strain using 2D speckle-tracking has recently been proposed as an important marker for adverse cardiovascular outcomes in AF.[86]
- Three-dimensional full-volume analyses of LV function, with single-beat analysis where feasible. This method has the advantage of not relying on geometric assumptions and allows the acquisition of full volume data within a single heartbeat. It correlates well with gold standard methods such as cardiac magnetic resonance imaging, but relies on adequate ultrasound windowing.
- Peak S-wave on tissue Doppler imaging (TDI) of the mitral valve annular sub-endocardium. This method has good correlation with LVEF across a wide range of function and is obtainable in patients with poor acoustic windows, but is limited in cases of regional wall motion abnormality.

Where poor quality acoustic windows limit accurate assessment of LV function, use of an intravenous contrast agent is recommended in participants without known allergy.

9.6.3 Diastolic LV Function

Diastolic LV function will be determined using the following methods (in all cases repeated over 3-5 cardiac cycles):

- Mitral inflow pulse-wave Doppler peak E velocity and deceleration time (DT).
- Mitral annular TDI to calculate septal E', lateral E' and the individual and averaged E/E' ratios.
- LV outflow tract pulse-wave Doppler to calculate isovolumic relaxation time (IVRT).

- Pulmonary vein pulse-wave Doppler to calculate peak systolic (where present) and diastolic velocities, ratio of peak velocities and DT of diastolic PV flow.
- Colour M-mode Doppler assessment of mitral flow propagation velocity (Vp) and ratio of E/Vp.

Overall diastolic function will be categorised according to the British Society of Echocardiography guidelines into normal function or mild/moderate/severe dysfunction based on a combination of the above variables. Individual parameters will also be categorised using cut-points identified from published studies.[87]

9.6.4 Left Atrial Size and Function

Left atrial (LA) size will be measured in the anteroposterior (parasternal long-axis), transverse and longitudinal dimensions (apical 4-chamber). LA volumes will be calculated using the biplane area-length method: (0.85 x 4-chamber LA area x 2-chamber LA area) / LA length. The length is measured from the middle of the plane of the mitral annulus to the superior aspect of the LA (shortest of 4- and 2-chamber measurements). LA volumes will be indexed for body surface area.

Where suitable datasets are obtained, 3D LA volumetric analysis and assessment of LA function and strain will also be performed.

9.6.5 Additional Echocardiography Parameters

The following parameters will be obtained in all participants:

- Tricuspid annular plane systolic excursion (TAPSE) for estimation of right ventricular function using pulse-wave Doppler.
- Where applicable, mitral regurgitation dP/dt.

9.7 Laboratory Evaluations

The use of biomarkers than can affect treatment decisions in AF is at an early stage of development.[88] The RATE-AF trial will allow us to collect and store blood samples on patients at baseline and follow-up, providing a unique biobank of AF patients receiving rate-control. In collaboration with the Human Biomaterials Resource Centre (HBRC) at the University of Birmingham, we will also isolate DNA for future work on predictors of response, including known polymorphisms of rate-responsiveness.[89]

Laboratories at each clinical site will process the standard laboratory investigations required as part of standard clinical care (see **Section 9.1**). Trial laboratory evaluations will be performed at the core laboratory and processed according to the guidelines in **Sections 9.7.1**, **9.7.2** and **9.7.3**.

9.7.1 Laboratory Assays

NT-pro B-type natriuretic peptide will be analysed using a Sandwich immunoassay using monoclonal ruthenium labelled antibody and Roche Cobas 8000 e602. The total coefficient of variation for repeatability with this assay is <2% with an estimated volume of 250 microlitres required for each test and measurement range of 5-35000 pg/mL (0.6-4130 pmol/L).

9.7.2 Cellular Response to Rate Control

The effect of baseline and follow-up serum on intracellular sodium/calcium, force of contraction and activation of ERK1/2-dependent cascades will be examined in human induced pluripotent stem cell-derived cardiomyocytes, using well-established integrated fluorescence/contractility photometry and western blotting techniques.[90, 91] DigiFAB[92], will be used to determine whether changes are dependent on endogenous cardiotonic steroids, which can modulate intracellular ion concentration in cardiomyocytes[93, 94], and potentially contribute to treatment discontinuation (or the development of toxicity).[95] The concentration of serum cardiotonic steroids will be determined using liquid chromatography–tandem mass spectrometry. Individual change in cardiotonic steroids and intracellular sodium/calcium will be correlated with the change in heart rate, LVEF, B-type natriuretic peptide and quality of life. In addition, we will identify patterns in patients withdrawing from treatment or experiencing adverse reactions.

9.7.3 Stored Blood Samples

Blood samples will be stored at HBRC for future biomarker and genetic analysis, with participants providing explicit consent for this process. Any future use of these samples will be undertaken with ethical approval.

9.7.4 Specimen Preparation, Handling, Storage and Shipment

Specimens will be handled according to local standard operating procedures consisting of the time requirements for processing, required temperatures, aliquots of specimens, where they will be stored, how they will be labelled, the process for remnant samples/disposal and appropriate instructions for transportation.

9.8 Economic Evaluation

The RATE-AF trial will allow determination of the most appropriate data collection methods and ease of acquiring resource use and cost data for a subsequent outcomes trial. Specifically, how data is obtained from secondary care records, patient-reported resource use and the feasibility of obtaining primary care records. A preliminary economic evaluation from an NHS perspective will be performed to estimate costs over the 12-month period. The patient-level cost-analysis will determine all AF-related costs, with respect to trial interventions and secondary-care resource use (including adverse events) in the two arms of the trial. We will collect both cost and outcome data and present them in a cost-consequence analysis. Costing for this trial suggests that simplifying medication alone could result in a saving of £5900 over each 12-month period.

Considering the high and increasing prevalence of AF, this could result in a substantial NHS cost savings, particularly if adverse reactions are also reduced. The aim of this objective within the trial is to prepare the groundwork for a future cost-per-quality adjusted life year (QALY) analysis of rate-control in AF.

Costs of care will be derived from patient level resource-use data, focusing on secondary care costs, and including adverse effects, such as pacemaker implantation. Other major drivers of cost are hospitalisation (including visits to Accident & Emergency), unplanned outpatient visits and outpatient tests such as echocardiography or ambulatory ECG. The cost analysis will also consider therapy costs, both trial drug and additional treatments. Unit costs will be obtained from standard sources including NHS Reference Costs, Unit Costs of Health and Social Care[96] and health care providers. Total per-patient health care costs will initially be calculated thus allowing the estimation of mean costs per trial arm over 12 months follow-up. Responses to the EQ-5D-5L questionnaire at baseline, visit 2 (6 months) and visit 3 (12 months) will be used to plan a future QALY analysis.

Key feasibility elements are:

- Determining the best methods for obtaining hospitalisation data, including where participants have been hospitalised outside of research site
- Whether robust primary care costs can be estimated and the method(s) for acquiring this type of data
- How key cost drivers can be incorporated into data collection for any future trial

10 Pharmacovigilance

Definitions of different types of AE are listed in **Table 2**. The Investigator should assess the seriousness and causality (relatedness) of AEs experienced by the participant (this should be documented in the source data). For further information please refer to **Section 10.1**.

Table 2: Standard AE Definitions

Term	Definition
**Adverse Event (AE)**	Any untoward medical occurrence in a patient or clinical trial participant administered a medicinal product and which does not necessarily have a causal relationship with this treatment
**Adverse Reaction (AR)**	Any untoward and unintended response in a participant to an investigational medicinal product which is related to any dose administered to that participant
**Serious adverse event (SAE), serious adverse reaction (SAR) or unexpected serious adverse reaction**	Any adverse event, adverse reaction or unexpected adverse reaction, respectively, that:<br>• results in death;<br>• is life-threatening;<br>• requires hospitalisation or prolongation of existing hospitalisation;<br>• results in persistent or significant disability or incapacity; or<br>• consists of a congenital anomaly or birth defect
**Unexpected Adverse Reaction**	An adverse reaction the nature and severity of which is not consistent with the information about the medicinal product in question set out:<br>(a) in the case of a product with a marketing authorisation, in the summary of product characteristics for that product;<br>(b) in the case of any other investigational medicinal product, in the investigator's brochure relating to the trial in question.
**SUSAR**	Suspected Unexpected Serious Adverse Reaction

10.1 Recording and Assessment of Adverse Events

All adverse events will be reportable to the **RATE-AF** Trial Office up to 30 days post last IMP administration. Any SUSAR related to the IMP should to be reported irrespective of how long after IMP administration the reaction has occurred.

Adverse events will be recorded in the medical records and CRFs. Most AE/ARs that occur in this trial, whether they are serious or not, will be 'expected' treatment-related toxicities due to the drugs used in this trial.

Refer to **Table 3** for definition of expectedness.

Table 3: Expectedness

Category	Definition
**Expected**	An adverse event that is classed in nature as serious and which is consistent with the information about the IMP listed in the Investigator Brochure (or SmPC if Licensed IMP) or clearly defined in this protocol
**Unexpected**	An adverse event that is classed in nature as serious and which is not consistent with the information about the IMP listed in the Investigator Brochure (or SmPC if Licensed IMP)

Adverse events will be recorded with clinical symptoms and accompanied with a simple, brief description of the event, including dates as appropriate.

The assessment of relationship of adverse events to the administration of IMP is a clinical decision based on all available information at the time. The following categories as outlined in **Table 4** will be used to define the causality of the adverse event.

Table 4: Categorisation of Causality

Category	Definition
**Definitely**	There is clear evidence to suggest a causal relationship, and other possible contributing factors can be ruled out
**Probably**	There is evidence to suggest a causal relationship, and the influence of other factors is unlikely
**Possibly**	There is some evidence to suggest a causal relationship (e.g. the event occurred within a reasonable time after administration of the trial medication). However, the influence of other factors may have contributed to the event (e.g. the patient's clinical condition, other concomitant events)
**Unlikely**	There is little evidence to suggest there is a causal relationship (e.g. the event did not occur within a reasonable time after administration of the trial medication). There is another reasonable explanation for the event (e.g. the patient's clinical condition, other concomitant treatments)
**Not related**	There is no evidence of any causal relationship

The relevant SmPC for Digoxin and Bisoprolol (which will be dependent on which generic is being used according to local practice at each site) should be filed in the site file by the local research team.

The **RATE-AF** Trial protocol and the reference safety information will be used to assess disease related and/or expected events related to the trial treatment.

10.2 Non-Serious Adverse Events/ Adverse Reactions

Refer to Table 2 for definitions

Common adverse reactions (see Section 7.4) will be recorded on the relevant CRF and sent to the RATE-AF Trial Office.

10.3 Serious Adverse Events

Refer to Table 2 for definitions

All Serious Adverse Events (SAEs), that are not excluded from expedited reporting will be recorded in the hospital notes and should be reported to the **RATE-AF** Trial Office on a SAE Form. The completed form should be **faxed to the RATE-AF Trial Office on 0121 415 9135 or 0121 415 9136**, as soon as possible and ideally within one working day of becoming aware of the event. The site Investigator should be able to respond to any related queries raised by the **RATE-AF** Trial Office as soon as possible.

10.3.1 Expected SAEs NOT to be Reported on a SAE Form

Expected SAEs are those listed in the current SmPC for the trial IMPs and can be excluded from the expedited reporting outlined in **Section 10.1**, for example if they are expected to occur on a regular basis and offer no further new information to the safety profile. These events should continue to be recorded in the source data and relevant CRFs.

In addition, events **NOT** considered to be SAEs are hospitalisations for:

- Routine treatment or monitoring of the studied indication, not associated with any deterioration in condition
- Treatment, which was elective or pre-planned, for a pre-existing condition that is unrelated to the indication under trial, and has not worsened

Note: Death from any cause should be reported on an SAE Form and returned to the **RATE-AF** Trial Office.

10.4 SUSARs

Refer to Table 2 for definitions

SAEs classed by as both suspected to be related to the trial IMP and unexpected are categorised as SUSARs, and are always subject to expedited reporting. An SAE Form should be completed,

and faxed to the RATE-AF Trial Office within 24 hours of the research staff at site becoming aware of the event. The local investigator will provide the causality assessment.

The Chief Investigator (or nominated individual) will undertake urgent review of all such SAEs and may request further information immediately from the clinical team at site. The Chief Investigator will not overrule the causality or seriousness assessment given by the local investigator but may add additional comment on these. The Chief Investigator will provide the determination of expectedness according to the reference safety information.

SUSARs will be notified to the MHRA and REC by the RATE-AF Trial Office. SUSARs that are fatal or life-threatening will be notified to the MHRA and REC within 7 days after the **RATE-AF** Trial Office has been notified. Other SUSARs will be reported to the REC and MHRA within 15 days after the Trial Office has been notified.

10.5 Development Safety Update Reports

The **RATE-AF** Trial Office will provide the MHRA with Development Safety Update Reports (DSURs). The reports will be submitted within 60 days of the Developmental International Birth Date (DIBD) of the trial each year until the trial is declared ended.

10.6 Annual Progress Reports

An annual progress report will be submitted to the REC within 30 days of the anniversary date on which the favourable opinion was given, and annually until the trial is declared ended.

10.7 Pregnancy

Due to the age of participants that will be randomised into the **RATE-AF** Trial (≥ 60 years), it is highly improbable that female participants will be pregnant at the time of randomisation, or become pregnant during the trial. Any pregnancies will be followed up for outcome; any outcome meeting the definition of an SAE will be reported to the **RATE-AF** Trial Office on the relevant CRF.

10.8 Reporting Urgent Safety Measures

If any urgent safety measures are taken the Principal Investigator/BCTU/Sponsor shall immediately and in any event no later than 3 days from the date the measures are taken, give written notice to the MHRA and the REC of the measures taken and the circumstances giving rise to those measures.

11 Quality Control and Quality Assurance

11.1 Site Set-Up and Initiation

All participating Principal Investigators will be asked to sign the necessary agreements and supply a current CV to the Trials Office. All members of the site research team will also be required to sign a site signature and delegation log. Prior to commencing recruitment all sites will undergo a process of initiation and will have completed GCP training. Key members of the site research team will be required to attend either a meeting or a teleconference covering aspects of the trial design, protocol procedures, Adverse Event reporting, collection and reporting of data and record keeping. Sites will be provided with an Investigator Site File containing essential documentation, instructions, and other documentation required for the conduct of the trial. The Trials Office must be informed immediately of any change in the site research team.

11.2 Central Monitoring

Monitoring of this trial will be to ensure compliance with Good Clinical Practice. A risk proportionate approach to the initiation, management and monitoring of the trial will be adopted (as per the MRC/DH/MHRA Joint Project: Risk-adapted Approaches to the Management of Clinical Trials of Investigational Medicinal Products) and outlined in the trial-specific risk assessment.

The Trials Office will be in regular contact with the site research team to check on progress and address any queries that they may have. The Trials Office will check incoming CRFs for compliance with the protocol, data consistency, missing data and timing. Sites will be asked for missing data or clarification of inconsistencies or discrepancies. Sites will be requested to send in copies of signed Informed Consent Forms and other documentation for in-house review for all participants providing explicit consent.

Additional on-site monitoring visits may be triggered, for example by poor CRF return, poor data quality, low SAE reporting rates, excessive number of participant withdrawals or deviations. This will be detailed in the monitoring plan. If a monitoring visit is required the Trials Office will contact the site to arrange a date for the proposed visit and will provide the site with written confirmation. Investigators will allow the **RATE-AF** trial staff access to source documents as requested.

11.3 Audit and Inspection

The Principal Investigator will permit trial-related monitoring, quality checks, audits, ethical reviews, and regulatory inspection(s) at their site, providing direct access to source data/documents. The Principal Investigator will comply with these visits and any required follow up. Sites are also requested to notify the Trials Office of any MHRA inspections.

11.4 Notification of Serious Breaches

In accordance with Regulation 29A of the Medicines for Human Use (Clinical Trials) Regulations 2004 and its amendments the Sponsor of the trial is responsible for notifying the licensing authority in writing of any serious breach of the conditions and principles of GCP in connection with that trial or the protocol relating to that trial, within 7 days of becoming aware of that breach.

For the purposes of this regulation, a "serious breach" is a breach which is likely to effect to a significant degree the safety or physical or mental integrity of the participants of the trial; or the scientific value of the trial. Sites are therefore requested to notify the Trials office of any suspected trial-related serious breach of GCP and/or the trial protocol. Where the Trials Office is investigating whether or not a serious breach has occurred sites are also requested to cooperate with the Trials Office in providing sufficient information to report the breach to the MHRA where required and in undertaking any corrective and/or preventive action. Sites may be suspended from further recruitment in the event of serious and persistent non-compliance with the protocol and/or GCP, and/or poor recruitment. Any major problems identified during monitoring may be reported to Trial Management Group and Trial Oversight Committee, the REC and the relevant regulatory bodies. This includes reporting serious breaches of GCP and/or the trial protocol to the REC and MHRA. A copy is sent to the University of Birmingham Clinical Research Compliance Team at the time of reporting to the REC and/or relevant regulatory bodies.

11.5 Data Handling and Analysis

Paper CRFs must be completed, signed/ dated and either entered directly online or returned to the **RATE-AF** Trial Office by the PI or an authorised member of the site research team (as delegated on the **RATE-AF** Trial Signature and Delegation Log) within the timeframe listed in **Table 5**. Copies of all completed CRFs should be filed in the site file. Entries on paper CRFs should be made in ballpoint pen, in black ink, and must be legible. Any errors should be crossed out with a single stroke, the correction inserted and the change initialled and dated. If it is not obvious why a change has been made, an explanation should be written next to the change.

CRFs can be entered online at http://www.bctu.bham.ac.uk/RATEAF. Authorised staff at sites will require an individual secure login username and password to access this online data entry system.

Data reported on each CRF should be consistent with the source data or the discrepancies should be explained. If information is not known, this must be clearly indicated on the CRF. All missing and ambiguous data will be queried. All sections are to be completed.

CRF versions may be updated by the **RATE-AF** Trial Office, as appropriate, throughout the duration of the trial. Whilst this will not constitute a protocol amendment, new versions of the CRFs must be implemented by participating sites immediately on receipt.

It will be the responsibility of the Principal Investigator to ensure the accuracy of all data entered in the CRFs. The **RATE-AF** Trial Signature & Delegation Log will identify all those personnel with responsibilities for data collection.

Access to data, including the final trial dataset, will be limited to members of the Research Team.

The investigator(s)/institution(s) will permit trial-related monitoring, audits, REC review, and regulatory inspection(s), providing direct access to source data/documents. Trial participants are informed of this during the informed consent discussion and will consent to provide access to their medical notes.

Table 5: Data Collection Forms

Form Name	Schedule for submission
**Randomisation Form**	Collected at randomisation
**Baseline and Follow-Up CRFs**	As soon as possible after each follow-up assessment time point
**Serious Adverse Event Form**	Faxed within 24hrs of research staff at site becoming aware of event

11.6 End of Trial

The end of trial will be 30 days after the last data capture. This will allow sufficient time for the completion of protocol procedures, data collection and data input. The Trials Office will notify the MHRA and REC that the trial has ended within 90 days of the end of trial. Where the trial has terminated early, the Trials Office will inform the MHRA and REC within 15 days of the end of trial. The Trials Office will provide them with a summary of the clinical trial report within 12 months of the end of trial.

A copy of the end of trial notification as well as the summary report is also sent to the University of Birmingham Research Governance Team at the time of sending these are sent to the MHRA and REC.

11.7 Archiving

Archiving will be authorised by the BCTU on behalf of the Sponsor following submission of the end of trial report.

Principal Investigators are responsible for the secure archiving of essential trial documents (for their site) as per their NHS Trust policy. All essential documents will be archived for a minimum of 25 years after completion of trial.

Destruction of essential documents will require authorisation from the BCTU on behalf of the Sponsor.

12 Statistical Considerations

12.1 Outcome measures

12.1.1 Primary Outcome

Patient-reported quality of life (QoL) - SF-36 physical component summary score at six months

12.1.2 Secondary Outcomes

Patient-reported QoL:

- SF-36 global and domain-specific scores at 6 and 12 months
- EQ-5D-5L summary index and visual analogue scale at six and twelve months
- AFEQT overall score at six and twelve months

Cardiac function:

- Echocardiographic LVEF at 12 months
- Diastolic function (E/e' and composite of diastolic indices) at 12 months
- Functional assessment:
- Six-minute walking distance at 6 and 12 months
- Change in European Heart Rhythm Association (EHRA) class at 6 and 12 months

Biomarkers:

- Change in B-type natriuretic peptide (BNP) levels at 6 months
- Change in heart rate using 24-hour ambulatory ECG

12.1.3 Feasibility Outcomes

- Recruitment target of 3 patients per week across all participating centres.
- Compliance and reasons for non-compliance
- Number of withdrawals and losses to follow-up (with reasons)
- Drug discontinuation rate and adverse reactions requiring drug discontinuation.
- Number of patients needing therapy-induced requirement for additional treatment
- Population-specific standard deviations (SD) and proportions

  - *SD of SF36 physical functioning score at 6 and 12 months*
  - *SD of SF36 overall score at 6 and 12 months*
  - *SD of AFEQT overall score at 6 and 12 months*
  - *SD of LVEF and E/e' score at 6 and 12 months*
  - *Unplanned hospitalisation admissions rates*
- Cardiovascular Events (particularly mortality, thromboembolic events, myocardial infarction and cardiovascular interventions)

The final analyses will follow a pre-specified analysis plan, drafted in conjunction with the Birmingham Clinical Trials Unit and submitted to the steering committee at the penultimate meeting. We intend to perform a primary intention-to-treat analysis, in addition to a per-protocol analysis.

Any additional (exploratory) analyses will be explicitly labelled as such in any subsequent manuscript.

12.2 Power Calculations

Randomising 144 patients we can assume an 85% power to detect an effect size of half a standard deviation in a continuous outcome measure of QoL (two-sided alpha of 0.05). A sample size of 160 patients would account for an estimated 10% loss to follow-up (including withdrawal and death prior to 12-month assessment). There is some evidence from existing research to support the notion that the treatment effect could be this large. The mean SF-36 role-physical score from the rate-control arm of the RACE study was 47, with a 17% improvement with rate-control over time.[62] In another study, CCB resulted in 22% improvement in a proprietary symptom-checklist, compared to a non-significant 8% change in those assigned to beta-blockers (SD 10-points in both groups). These values are also consistent with a 17% improvement in SF-36 scores in a third trial, PIAF.[63] Thus whilst it is possible that this trial may provide a clear indication of effect, it is accepted that the trial will be underpowered to detect smaller differences, reinforcing the requirement for a larger definitive trial which would also be powered to assess impact on clinical event rates.

12.3 Statistical analysis

A separate Statistical Analysis Plan for the RATE-AF trial provides a detailed description of the planned statistical analyses. A brief outline of these analyses is given below.

The primary comparison groups will be composed of those who are randomised to digoxin group and those randomised to the beta-blockers group. All analyses will be based on the intention to treat principle, with all patients analysed in the arms to which they were allocated irrespective of compliance with the randomised allocated treatment, and all patients will be included in the analyses. We will, as a sensitivity analysis, conduct per-protocol analyses, where appropriate.

For all analyses, a p-value <0.05 will be considered statistically significant.

12.3.1 Primary outcome analysis

The primary outcome for this trial is the continuous SF36 physical functioning domain score at 6 months. This outcome will be analysed using an ANCOVA model adjusting for treatment arm, baseline score and all minimisation variables. Results will be presented as difference in means and 95% confidence intervals.

12.3.2 Feasibility and Secondary outcomes analysis

The feasibility and secondary outcomes for the trial comprise of a combination of both continuous and categorical (dichotomous) data items.

Categorical endpoints:

For outcomes which are categorical (dichotomous) in nature, the proportion of participants and percentages will be analysed between arms.

Logistic/Log-binomial regression models will be fitted (where appropriate) to adjust for treatment arm, baseline scores and all minimisation variables.

Results will be presented as odds ratios/relative risks and 95% confidence intervals.

Continuous endpoints:

Any outcomes that are continuous in nature will be analysed in the same way as the primary outcome.

12.3.3 Missing data and sensitivity analyses

Primary analysis will concentrate on available data only, with no attempt made to impute missing data. Where appropriate, sensitivity analyses will be carried out to examine the possible impact of missing data on the results (full details will be discussed within the Statistical Analysis Plan).

12.3.4 Interim analyses and Stopping rules

Analysis of the data with respect to efficacy and safety will be performed at 12 months and sent to Data Monitoring Committee (DMC); see **Section 16**. The DMC will outline and agree the stopping rules for the trial which will be documented in the DMC charter. It is likely that the Haybittle-Peto boundary will be used. This states that if an interim analysis shows a probability of less than 0.001 that the treatments are different, then the trial should be stopped early. This will be used alongside data on important secondary endpoints and all other relevant evidence. A DMC report and charter outlining the terms of reference (including information on stopping rules) will be agreed with the DMEC.

12.4 Final analysis

The final analysis for the RATE-AF trial will occur once the last randomised participant completes their 12-month follow-up.

13 Ethics and Regulatory Requirements

The trial will be performed in accordance with the recommendations guiding physicians in biomedical research involving human participants, adopted by the 18th World Medical Association General Assembly, Helsinki, Finland, June 1964, amended at the 48th World Medical Association General Assembly, Somerset West, Republic of South Africa, October 1996 (website: http://www.wma.net/en/30publications/10policies/b3/index.html).

The trial will be conducted in accordance with the Research Governance Framework for Health and Social Care, the applicable UK Statutory Instruments, (which include the Medicines for Human Use Clinical Trials 2004 and subsequent amendments and the Data Protection Act 1998 and Human Tissue Act 2008, EU Clinical Trials Directive and amendment Regulations as appropriate) and Guidelines for Good Clinical Practice (GCP). This trial will be carried out under a Clinical Trial Authorisation in accordance with the Medicines for Human Use Clinical Trials regulations. The protocol will be submitted to and approved by the REC prior to circulation.

Before any participants are enrolled into the trial, the Principal Investigator at each site is required to obtain local R&D approval. Sites will not be permitted to enrol participants until written confirmation of R&D approval is received by the Principal Investigator.

It is the responsibility of the Principal Investigator to ensure that all subsequent amendments gain the necessary local approval. This does not affect the individual clinicians' responsibility to take immediate action if thought necessary to protect the health and interest of individual participants.

Within 90 days after the end of the trial, the Chief Investigator/Sponsor will ensure that the REC and the MHRA are notified that the trial has finished. If the trial is terminated prematurely, those reports will be made within 15 days after the end of the trial. The Chief Investigator will provide the Sponsor with a summary report of the clinical trial, which will then be submitted to the MHRA and REC within one year after the end of the trial.

14 Oversight Committees

14.1 Trial Management Group

The Trial Management Group (TMG) will comprise the CI, other lead investigators (clinical and non-clinical) and members of the BCTU. The TMG will be responsible for the day-to-day running and management of **RATE-AF**. The TMG will convene at regular intervals.

14.2 Trial Oversight Committee

A joint oversight committee comprising a Trial Steering Committee (TSC) and Data Monitoring Committee (DMC) will be engaged for this trial.

The role of the TSC is to provide the overall supervision of the trial. The TSC will monitor trial progress and conduct and advice on scientific credibility. The TSC will consider and act, as appropriate, upon the recommendations of the Data Monitoring Committee. Further details of the remit and role of the TSC are available in the TSC Charter.

An independent DMC will be established to oversee the safety of participants in the trial. The DMC will meet prior to the trial opening to enrolment, and then meet at least annually, or as per a timetable agreed by the DMC prior to trial commencement. Data analyses will be supplied in confidence to the DMC, which will be asked to give advice on whether the accumulated data from the trial, together with the results from other relevant research, justifies the continuing recruitment of further participants. The DMC will operate in accordance with the trial specific charter.

14.3 Protocol amendments

Where important protocol modifications are required as a result of oversight (for example, changes to eligibility criteria, outcomes or analyses), this information will be communicated to relevant parties, such as investigators, the REC, trial registries and regulators.

15 Finance

The **RATE-AF** Trial is funded through a Career Development Fellowship awarded to the Chief Investigator by the National Institute for Health Research (NIHR).

16 Confidentiality and Data Protection

Personal data recorded on all documents will be regarded as strictly confidential and will be handled and stored in accordance with the Data Protection Act 1998.

Participants will be identified using their unique trial identification number, date of birth and hospital number on the CRFs. and correspondence between the Trials Office and the participating site. Participants will give their explicit consent for the movement of their consent form, giving permission for the Trials Office to be sent a copy. This will be used to perform in-house monitoring of the consent process.

The Investigator must maintain documents not for submission to the Trials Office (e.g. Participant Identification Logs) in strict confidence. In the case of specific issues and/or queries from the

regulatory authorities, it will be necessary to have access to the complete trial records, provided that participant confidentiality is protected.

The Trials Office will maintain the confidentiality of all participants' data and will not disclose information by which participants may be identified to any third party other than those directly involved in the treatment of the participant and organisations for which the participant has given explicit consent for data transfer (e.g. competent authority, sponsor). Representatives of the **RATE-AF** Trials Office and sponsor may be required to have access to participant's notes for quality assurance purposes but participants should be reassured that their confidentiality will be respected at all times.

17 Insurance and Indemnity

The University of Birmingham has in place Clinical Trials indemnity coverage for this trial which provides cover to the University for harm which comes about through the University's, or its staff's, negligence in relation to the design or management of the trial and may alternatively, and at the University's discretion provide cover for non-negligent harm to participants.

With respect to the conduct of the trial at the Clinical Site and other clinical care of the patient, responsibility for the care of the patients remains with the NHS organisation responsible for the Clinical Site and is therefore indemnified through the NHS Litigation Authority.

The University of Birmingham is independent of any pharmaceutical company, and as such it is not covered by the Association of the British Pharmaceutical Industry (ABPI) guidelines for participant compensation.

18 Dissemination and Publication

Regular newsletters will keep collaborators informed of trial progress and regular meetings will be held to report the progress of the trial and to address any problems encountered in the conduct of the trial. The CI will coordinate dissemination of data from this trial. All publications and presentations, including abstracts, relating to the main trial will be authorised by the **RATE-AF** TMG. The results of the analysis will be published in the name of the **RATE-AF** Collaborative Group in a peer reviewed journal (provided that this does not conflict with the journal's policy).

Named authors must satisfy the International Committee of Medical Journal Editors (ICMJE) criteria for authorship (contribute to drafting of the article or revision for important intellectual content), provide timely approval of the final version to be published and supply detailed statements on any potential conflict of interest or financial relationship (http://www.icmje.org/). Members of the group who do not fulfil ICMJE criteria for authorship will be listed in the article appendix. Trial participants will be sent a lay summary of the final results of the trial, which will contain a reference to the full paper.

19 Statement of Compliance

The **RATE-AF** trial will be conducted in compliance with the approved protocol, EU GCP and the applicable regulatory requirements.

20 References

1. Camm AJ, Kirchhof P, Lip GY, Schotten U, Savelieva I, Ernst S, Van Gelder IC, Al-Attar N, Hindricks G, Prendergast B, Heidbuchel H, Alfieri O, Angelini A, Atar D, Colonna P, De Caterina R, De Sutter J, Goette A, Gorenek B, Heldal M, Hohloser SH, Kolh P, Le Heuzey JY, Ponikowski P, Rutten FH. Guidelines for the management of atrial fibrillation: the Task Force for the Management of Atrial Fibrillation of the European Society of Cardiology (ESC). *Eur Heart J*. 2010;31:2369-2429

2. Anderson JL, Halperin JL, Albert NM, Bozkurt B, Brindis RG, Curtis LH, DeMets D, Guyton RA, Hochman JS, Kovacs RJ, Ohman EM, Pressler SJ, Sellke FW, Shen W-K. Management of patients with atrial fibrillation (Compilation of 2006 ACCF/AHA/ESC and 2011 ACCF/AHA/HRS recommendations). *Circulation*. 2013;127:1916-1926

3. Camm AJ, Lip GY, De Caterina R, Savelieva I, Atar D, Hohnloser SH, Hindricks G, Kirchhof P, E. S. C. Committee for Practice Guidelines. 2012 focused update of the ESC Guidelines for the management of atrial fibrillation: an update of the 2010 ESC Guidelines for the management of atrial fibrillation. Developed with the special contribution of the European Heart Rhythm Association. *Eur Heart J*. 2012;33:2719-2747

4. National Institute for Health and Care Excellence. Atrial fibrillation: the management of atrial fibrillation. *NICE clinical guideline 180*. 2014; Accessed 15/09/2014; http://www.nice.org.uk/guidance/cg180/

5. Kotecha D, Holmes J, Krum H, Altman DG, Manzano L, Cleland JG, Lip GY, Coats AJ, Andersson B, Kirchhof P, von Lueder TG, Wedel H, Rosano G, Shibata MC, Rigby A, Flather MD, Beta-Blockers in Heart Failure Collaborative Group. Efficacy of beta blockers in patients with heart failure plus atrial fibrillation: an individual-patient data meta-analysis. *Lancet*. 2014;384:2235-2243

6. Ziff OJ, Lane DA, Samra M, Griffith M, Kirchhof P, Lip GY, Steeds RP, Townend J, Kotecha D. Safety and efficacy of digoxin: systematic review and meta-analysis of observational and controlled trial data. *BMJ*. 2015;351:h4451

7. Kotecha D, Banerjee A, Lip GY. Increased stroke risk in atrial fibrillation patients with heart failure: does ejection fraction matter? *Stroke*. 2015;46:608-609

8. Kotecha D, Kirchhof P. Rate and rhythm control have comparable effects on mortality and stroke in atrial fibrillation but better data are needed. *Evid Based Med*. 2014;19:222-223

9. Kotecha D, Piccini JP. Atrial fibrillation in heart failure: what should we do? *Eur Heart J*. 2015;36:3250-3257

10. Senoo K, Lip GY, Lane DA, Buller HR, Kotecha D. Residual risk of stroke and death in anticoagulated patients according to the type of atrial fibrillation: AMADEUS Trial. *Stroke*. 2015;46:2523-2528

11. Kotecha D, Chudasama R, Lane DA, Kirchhof P, Lip GY. Atrial fibrillation and heart failure due to reduced versus preserved ejection fraction: A systematic review and meta-analysis of death and adverse outcomes. *Int J Cardiol*. 2016;203:660-666

12. Arain M, Campbell MJ, Cooper CL, Lancaster GA. What is a pilot or feasibility study? A review of current practice and editorial policy. *BMC Med Res Methodol*. 2010;10:67

13. Thabane L, Ma J, Chu R, Cheng J, Ismaila A, Rios LP, Robson R, Thabane M, Giangregorio L, Goldsmith CH. A tutorial on pilot studies: the what, why and how. *BMC Med Res Methodol*. 2010;10:1

14. Heeringa J, van der Kuip DAM, Hofman A, Kors JA, van Herpen G, Stricker BHC, Stijnen T, Lip GYH, Witteman JCM. Prevalence, incidence and lifetime risk of atrial fibrillation: the Rotterdam study. *Eur Heart J*. 2006;27:949-953

15. Colilla S, Crow A, Petkun W, Singer DE, Simon T, Liu X. Estimates of Current and Future Incidence and Prevalence of Atrial Fibrillation in the U.S. Adult Population. *Am J Cardiol*. 2013;112:1142-1147

16. Kim MH, Johnston SS, Chu BC, Dalal MR, Schulman KL. Estimation of total incremental health care costs in patients with atrial fibrillation in the United States. *Circ Cardiovasc Qual Outcomes*. 2011;4:313-320

17. Benjamin EJ, Wolf PA, D'Agostino RB, Silbershatz H, Kannel WB, Levy D. Impact of Atrial Fibrillation on the Risk of Death: The Framingham Heart Study. *Circulation*. 1998;98:946-952

18. Stewart S, Hart CL, Hole DJ, McMurray JJV. A population-based study of the long-term risks associated with atrial fibrillation: 20-year follow-up of the Renfrew/Paisley study. *Am J Med*. 2002;113:359-364

19. Marijon E, Le Heuzey JY, Connolly S, Yang S, Pogue J, Brueckmann M, Eikelboom J, Themeles E, Ezekowitz M, Wallentin L, Yusuf S. Causes of death and influencing factors in patients with atrial fibrillation: a competing-risk analysis from the randomized evaluation of long-term anticoagulant therapy study. *Circulation*. 2013;128:2192-2201

20. Chen LY, Sotoodehnia N, Buzkova P, Lopez FL, Yee LM, Heckbert SR, Prineas R, Soliman EZ, Adabag S, Konety S, Folsom AR, Siscovick D, Alonso A. Atrial fibrillation and the risk of sudden cardiac death: the atherosclerosis risk in communities study and cardiovascular health study. *JAMA Intern Med*. 2013;173:29-35

21. Dorian P, Jung W, Newman D, Paquette M, Wood K, Ayers GM, Camm J, Akhtar M, Luderitz B. The impairment of health-related quality of life in patients with intermittent atrial fibrillation: implications for the assessment of investigational therapy. *J Am Coll Cardiol*. 2000;36:1303-1309

22. Thrall G, Lane D, Carroll D, Lip GYH. Quality of Life in Patients with Atrial Fibrillation: A Systematic Review. *Am J Med*. 2006;119:448.e441-419

23. Sears SF, Serber ER, Alvarez LG, Schwartzman DS, Hoyt RH, Ujhelyi MR. Understanding Atrial Symptom Reports: Objective versus Subjective Predictors. *Pacing Clin Electrophysiol*. 2005;28:801-807

24. Dries D, Exner D, Gersh B, Domanski M, Waclawiw M, Stevenson L. Atrial fibrillation is associated with an increased risk for mortality and heart failure progression in patients with asymptomatic and symptomatic left ventricular systolic dysfunction: a retrospective analysis of the SOLVD trials. *J Am Coll Cardiol*. 1998;32:695-703

25. Wang TJ, Larson MG, Levy D, Vasan RS, Leip EP, Wolf PA, D'Agostino RB, Murabito JM, Kannel WB, Benjamin EJ. Temporal Relations of Atrial Fibrillation and Congestive Heart Failure and Their Joint Influence on Mortality: The Framingham Heart Study. *Circulation*. 2003;107:2920-2925

26. The Atrial Fibrillation Follow-up Investigation of Rhythm Management (AFFIRM) Investigators. A Comparison of Rate Control and Rhythm Control in Patients with Atrial Fibrillation. *N Engl J Med*. 2002;347:1825-1833

27. Van Gelder IC, Hagens VE, Bosker HA, Kingma JH, Kamp O, Kingma T, Said SA, Darmanata JI, Timmermans AJM, Tijssen JGP, Crijns HJGM. A Comparison of Rate Control and Rhythm Control in Patients with Recurrent Persistent Atrial Fibrillation. *N Engl J Med*. 2002;347:1834-1840

28. Olshansky B, Rosenfeld LE, Warner AL, Solomon AJ, O'Neill G, Sharma A, Platia E, Feld GK, Akiyama T, Brodsky MA, Greene HL. The Atrial Fibrillation Follow-up Investigation of Rhythm Management (AFFIRM) study: approaches to control rate in atrial fibrillation. *J Am Coll Cardiol*. 2004;43:1201-1208

29. Hohnloser SH, Kuck KH, Lilienthal J. Rhythm or rate control in atrial fibrillation--Pharmacological Intervention in Atrial Fibrillation (PIAF): a randomised trial. *Lancet*. 2000;356:1789-1794

30. Carlsson J, Miketic S, Windeler J, Cuneo A, Haun S, Micus S, Walter S, Tebbe U, Investigators S. Randomized trial of rate-control versus rhythm-control in persistent atrial fibrillation: the Strategies of Treatment of Atrial Fibrillation (STAF) study. *J Am Coll Cardiol*. 2003;41:1690-1696

31. de Denus S, Sanoski CA, Carlsson J, Opolski G, Spinler SA. Rate vs rhythm control in patients with atrial fibrillation: a meta-analysis. *Arch Intern Med*. 2005;165:258-262

32. Chatterjee S, Sardar P, Lichstein E, Mukherjee D, Aikat S. Pharmacologic rate versus rhythm-control strategies in atrial fibrillation: an updated comprehensive review and meta-analysis. *PACE*. 2013;36:122-133

33. Shelton RJ, Clark AL, Goode K, Rigby AS, Houghton T, Kaye GC, Cleland JG. A randomised, controlled study of rate versus rhythm control in patients with chronic atrial fibrillation and heart failure: (CAFE-II Study). *Heart*. 2009;95:924-930

34. Roy D, Talajic M, Nattel S, Wyse DG, Dorian P, Lee KL, Bourassa MG, Arnold JMO, Buxton AE, Camm AJ, Connolly SJ, Dubuc M, Ducharme A, Guerra PG, Hohnloser SH, Lambert J, Le Heuzey J-Y, O'Hara G, Pedersen OD, Rouleau J-L, Singh BN, Stevenson LW, Stevenson WG, Thibault B, Waldo AL. Rhythm Control versus Rate Control for Atrial Fibrillation and Heart Failure. *N Engl J Med*. 2008;358:2667-2677

35. Kong MH, Shaw LK, O'Connor C, Califf RM, Blazing MA, Al-Khatib SM. Is rhythm-control superior to rate-control in patients with atrial fibrillation and diastolic heart failure? *Ann Noninvasive Electrocardiol*. 2010;15:209-217

36. Wazni O, Wilkoff B, Saliba W. Catheter Ablation for Atrial Fibrillation. *N Engl J Med*. 2011;365:2296-2304

37. Jones DG, Haldar SK, Hussain W, Sharma R, Francis DP, Rahman-Haley SL, McDonagh TA, Underwood SR, Markides V, Wong T. A randomized trial to assess catheter ablation versus rate control in the management of persistent atrial fibrillation in heart failure. *J Am Coll Cardiol*. 2013;61:1894-1903

38. Kirchhof P, Breithardt G, Camm AJ, Crijns HJ, Kuck KH, Vardas P, Wegscheider K. Improving outcomes in patients with atrial fibrillation: rationale and design of the Early treatment of Atrial fibrillation for Stroke prevention Trial. *Am Heart J*. 2013;166:442-448

39. Steg PG, Alam S, Chiang C-E, Gamra H, Goethals M, Inoue H, Krapf L, Lewalter T, Merioua I, Murin J, Naditch-Brûlé L, Ponikowski P, Rosenqvist M, Silva-Cardoso J, Zharinov O, Brette S, Neill JO. Symptoms, functional status and quality of life in patients with controlled and uncontrolled atrial fibrillation: data from the RealiseAF cross-sectional international registry. *Heart*. 2012;98:195-201

40. Nabauer M, Gerth A, Limbourg T, Schneider S, Oeff M, Kirchhof P, Goette A, Lewalter T, Ravens U, Meinertz T, Breithardt G, Steinbeck G. The Registry of the German Competence NETwork on Atrial Fibrillation: patient characteristics and initial management. *Europace*. 2009;11:423-434

41. Segal JB, McNamara RL, Miller MR, Kim N, Goodman SN, Powe NR, Robinson K, Yu D, Bass EB. The evidence regarding the drugs used for ventricular rate control. *J Fam Practice*. 2000;49:47-59

42. Farshi R, Kistner D, Sarma JSM, Longmate JA, Singh BN. Ventricular rate control in chronic atrial fibrillation during daily activity and programmed exercise: a crossover open-label study of five drug regimens. *J Am Coll Cardiol*. 1999;33:304-310

43. Koh KK, Kwon KS, Park HB, Baik SH, Park SJ, Lee KH, Kim EJ, Kim SH, Cho SK, Kim SS. Efficacy and safety of digoxin alone and in combination with low-dose diltiazem or betaxolol to control ventricular rate in chronic atrial fibrillation. *Am J Cardiol*. 1995;75:88-90

44. Nikolaidou T, Channer KS. Chronic atrial fibrillation: a systematic review of medical heart rate control management. *Postgrad Med J*. 2009;85:303-312

45. Ulimoen SR, Enger S, Carlson J, Platonov PG, Pripp AH, Abdelnoor M, Arnesen H, Gjesdal K, Tveit A. Comparison of four single-drug regimens on ventricular rate and arrhythmia-related symptoms in patients with permanent atrial fibrillation. *Am J Cardiol*. 2013;111:225-230

46. Ulimoen SR, Enger S, Pripp AH, Abdelnoor M, Arnesen H, Gjesdal K, Tveit A. Calcium channel blockers improve exercise capacity and reduce N-terminal Pro-B-type natriuretic peptide levels compared with beta-blockers in patients with permanent atrial fibrillation. *Eur Heart J*. 2014;35:517-524

47. Khand AU, Rankin AC, Martin W, Taylor J, Gemmell I, Cleland JG. Carvedilol alone or in combination with digoxin for the management of atrial fibrillation in patients with heart failure? *J Am Coll Cardiol*. 2003;42:1944-1951

48. Vamos M, Erath JW, Hohnloser SH. Digoxin-associated mortality: a systematic review and meta-analysis of the literature. *Eur Heart J*. 2015; In press: 10.1093/eurheartj/ehv143

49. Gheorghiade M, Fonarow GC, van Veldhuisen DJ, Cleland JG, Butler J, Epstein AE, Patel K, Aban IB, Aronow WS, Anker SD, Ahmed A. Lack of evidence of increased mortality among patients with atrial fibrillation taking digoxin: findings from post hoc propensity-matched analysis of the AFFIRM trial. *Eur Heart J*. 2013;34:1489-1497

50. Friberg L, Hammar N, Rosenqvist M. Digoxin in atrial fibrillation: report from the Stockholm Cohort study of Atrial Fibrillation (SCAF). *Heart*. 2010;96:275-280

51. Flory JH, Ky B, Haynes K, S MB, Munson J, Rowan C, Strom BL, Hennessy S. Observational cohort study of the safety of digoxin use in women with heart failure. *BMJ Open*. 2012;2:e000888

52. Andrey JL, Romero S, Garcia-Egido A, Escobar MA, Corzo R, Garcia-Dominguez G, Lechuga V, Gomez F. Mortality and morbidity of heart failure treated with digoxin. A propensity-matched study. *Int J Clin Pract*. 2011;65:1250-1258

53. Lewis RV, Irvine N, McDevitt DG. Relationships between heart rate, exercise tolerance and cardiac output in atrial fibrillation: the effects of treatment with digoxin, verapamil and diltiazem. *Eur Heart J*. 1988;9:777-781

54. Tsuneda T, Yamashita T, Fukunami M, Kumagai K, Niwano S, Okumura K, Inoue H. Rate control and quality of life in patients with permanent atrial fibrillation: the Quality of Life and Atrial Fibrillation (QOLAF) Study. *Circ J*. 2006;70:965-970

55. Van Gelder IC, Groenveld HF, Crijns HJGM, Tuininga YS, Tijssen JGP, Alings AM, Hillege HL, Bergsma-Kadijk JA, Cornel JH, Kamp O, Tukkie R, Bosker HA, Van Veldhuisen DJ, Van den Berg MP. Lenient versus Strict Rate Control in Patients with Atrial Fibrillation. *N Engl J Med*. 2010;362:1363-1373

56. Groenveld HF, Crijns HJGM, Van den Berg MP, Van Sonderen E, Alings AM, Tijssen JGP, Hillege HL, Tuininga YS, Van Veldhuisen DJ, Ranchor AV, Van Gelder IC. The Effect of Rate Control on Quality of Life in Patients With Permanent Atrial Fibrillation: Data From the RACE II (Rate Control Efficacy in Permanent Atrial Fibrillation II) Study. *J Am Coll Cardiol*. 2011;58:1795-1803

57. Mulder BA, Van Veldhuisen DJ, Crijns HJGM, Tijssen JGP, Hillege HL, Alings M, Rienstra M, Groenveld HF, Van den Berg MP, Van Gelder IC. Lenient vs. strict rate control in patients with atrial fibrillation and heart failure: a post-hoc analysis of the RACE II study. *Eur J Heart Fail*. 2013;15:1311-1318

58. Groenveld HF, Tijssen JG, Crijns HJ, Van den Berg MP, Hillege HL, Alings M, Van Veldhuisen DJ, Van Gelder IC. Rate control efficacy in permanent atrial fibrillation: successful and failed strict rate control against a background of lenient rate control: data from RACE II (Rate Control Efficacy in Permanent Atrial Fibrillation). *J Am Coll Cardiol*. 2013;61:741-748

59. US Department of Health and Human Services Food and Drug Administration. Guidance for industry: patient-reported outcome measures: use in medical product development to support

labeling claims. www.fda.gov/downloads/Drugs/GuidanceComplianceRegulatoryInformation/Guidances/UCM193282.pdf. 2009

60. Rienstra M, Lubitz SA, Mahida S, Magnani JW, Fontes JD, Sinner MF, Van Gelder IC, Ellinor PT, Benjamin EJ. Symptoms and Functional Status of Patients With Atrial Fibrillation: State of the Art and Future Research Opportunities. *Circulation*. 2012;125:2933-2943

61. Pepine CJ. Effects of pharmacologic therapy on health-related quality of life in elderly patients with atrial fibrillation: a systematic review of randomized and nonrandomized trials. *Clin Med Insights Cardiol*. 2013;7:1-20

62. Hagens VE, Ranchor AV, Van Sonderen E, Bosker HA, Kamp O, Tijssen JGP, Kingma JH, Crijns HJGM, Van Gelder IC. Effect of rate or rhythm control on quality of life in persistent atrial fibrillation: Results from the Rate Control Versus Electrical Cardioversion (RACE) study. *J Am Coll Cardiol*. 2004;43:241-247

63. Grönefeld GC, Lilienthal J, Kuck K-H, Hohnloser SH. Impact of rate versus rhythm control on quality of life in patients with persistent atrial fibrillation: Results from a prospective randomized study. *Eur Heart J*. 2003;24:1430-1436

64. Ware Jr JE, Gandek B. Overview of the SF-36 Health Survey and the International Quality of Life Assessment (IQOLA) Project. *J Clin Epidemiol*. 1998;51:903-912

65. Herdman M, Gudex C, Lloyd A, Janssen M, Kind P, Parkin D, Bonsel G, Badia X. Development and preliminary testing of the new five-level version of EQ-5D (EQ-5D-5L). *Qual Life Res*. 2011;20:1727-1736

66. Devlin NJ, Krabbe PF. The development of new research methods for the valuation of EQ-5D-5L. *Eur J Health Econ*. 2013;14 Suppl 1:S1-3

67. Spertus J, Dorian P, Bubien R, Lewis S, Godejohn D, Reynolds MR, Lakkireddy DR, Wimmer AP, Bhandari A, Burk C. Development and validation of the Atrial Fibrillation Effect on QualiTy-of-Life (AFEQT) Questionnaire in patients with atrial fibrillation. *Circ Arrhythm Electrophysiol*. 2011;4:15-25

68. Dorian P, Burk C, Mullin CM, Bubien R, Godejohn D, Reynolds MR, Lakkireddy DR, Wimmer AP, Bhandari A, Spertus J. Interpreting changes in quality of life in atrial fibrillation: How much change is meaningful? *Am Heart J*. 2013;166:381-387.e388

69. Joint Formulary Committee. *British National Formulary*. London: BMJ Group and Pharmaceutical Press; 2013.

70. American Hospital Formulary Service. *Drug Information*. Bethesda: American Society of Health-System Pharmacists; 2013.

71. The Digitalis Investigation Group. The effect of digoxin on mortality and morbidity in patients with heart failure. *N Engl J Med*. 1997;336:525-533

72. Magnani B, Malini PL. Cardiac glycosides. Drug interactions of clinical significance. *Drug Safety*. 1995;12:97-109

73. Ko DT, Hebert PR, Coffey CS, Sedrakyan A, Curtis JP, Krumholz HM. Beta-blocker therapy and symptoms of depression, fatigue, and sexual dysfunction. *JAMA*. 2002;288:351-357

74. Terwee C, Mokkink L, Knol D, Ostelo RJG, Bouter L, Vet HW. Rating the methodological quality in systematic reviews of studies on measurement properties: a scoring system for the COSMIN checklist. *Qual Life Res*. 2012;21:651-657

75. Streiner DL, Norman GR. *Health measurement scales : a practical guide to their development and use*. Oxford ; New York: Oxford University Press; 2008.

76. Staniszewska S, Haywood KL, Brett J, Tutton L. Patient and public involvement in patient-reported outcome measures: evolution not revolution. *Patient*. 2012;5:79-87

77. Calvert M, Kyte D, Duffy H, Gheorghe A, Mercieca-Bebber R, Ives J, Draper H, Brundage M, Blazeby J, King M. Patient-reported outcome (PRO) assessment in clinical trials: a systematic review of guidance for trial protocol writers. *PLoS One*. 2014;9:e110216

78. McCabe PJ, Schumacher K, Barnason SA. Living with atrial fibrillation: a qualitative study. *J Cardiovasc Nurs*. 2011;26:336-344

79. Kirchhof P, Auricchio A, Bax J, Crijns H, Camm J, Diener H-C, Goette A, Hindricks G, Hohnloser S, Kappenberger L, Kuck K-H, Lip GYH, Olsson B, Meinertz T, Priori S, Ravens U, Steinbeck G, Svernhage E, Tijssen J, Vincent A, Breithardt G. Outcome parameters for trials in atrial fibrillation: Recommendations from a consensus conference organized by the German Atrial Fibrillation Competence NETwork (AFNET) and the European Heart Rhythm Association (EHRA). *Eur Heart J*. 2007;28:2803-2817

80. Haywood K, Brett J, Salek S, Marlett N, Penman C, Shklarov S, Norris C, Santana MJ, Staniszewska S. Patient and public engagement in health-related quality of life and patient-reported outcomes research: what is important and why should we care? Findings from the first ISOQOL patient engagement symposium. *Qual Life Res*. 2014: Epub ahead of print; PMID 25194573

81. Chan AW, Tetzlaff JM, Gotzsche PC, Altman DG, Mann H, Berlin JA, Dickersin K, Hrobjartsson A, Schulz KF, Parulekar WR, Krleza-Jeric K, Laupacis A, Moher D. SPIRIT 2013 explanation and elaboration: guidance for protocols of clinical trials. *BMJ*. 2013;346:e7586

82. Calvert M, Kyte D, von Hildebrand M, King M, Moher D. Putting patients at the heart of health-care research. *Lancet*. 2015;385:1073-1074

83. Kyte D, Duffy H, Fletcher B, Gheorghe A, Mercieca-Bebber R, King M, Draper H, Ives J, Brundage M, Blazeby J, Calvert M. Systematic evaluation of the patient-reported outcome (PRO) content of clinical trial protocols. *PLoS One*. 2014;9:e110229

84. Kyte D, Draper H, Calvert M. Patient-reported outcome alerts: ethical and logistical considerations in clinical trials. *JAMA*. 2013;310:1229-1230

85. Zbrozek A, Hebert J, Gogates G, Thorell R, Dell C, Molsen E, Craig G, Grice K, Kern S, Hines S. Validation of electronic systems to collect patient-reported outcome (PRO) data-recommendations for clinical trial teams: report of the ISPOR ePRO systems validation good research practices task force. *Value Health*. 2013;16:480-489

86. Su H-M, Lin T-H, Hsu P-C, Lee W-H, Chu C-Y, Lee C-S, Voon W-C, Lai W-T, Sheu S-H. Global left ventricular longitudinal systolic strain as a major predictor of cardiovascular events in patients with atrial fibrillation. *Heart*. 2013;99:1588-1596

87. Al-Omari MA, Finstuen J, Appleton CP, Barnes ME, Tsang TSM. Echocardiographic assessment of left ventricular diastolic function and filling pressure in atrial fibrillation. *Am J Cardiol*. 2008;101:1759-1765

88. Hijazi Z, Oldgren J, Siegbahn A, Granger CB, Wallentin L. Biomarkers in atrial fibrillation: a clinical review. *Eur Heart J*. 2013;34:1475-1480

89. Parvez B, Chopra N, Rowan S, Vaglio JC, Muhammad R, Roden DM, Darbar D. A common beta1-adrenergic receptor polymorphism predicts favorable response to rate-control therapy in atrial fibrillation. *J Am Coll Cardiol*. 2012;59:49-56

90. Mahmmoud YA, Shattock M, Cornelius F, Pavlovic D. Inhibition of K+ transport through Na+, K+-ATPase by capsazepine: role of membrane span 10 of the alpha-subunit in the modulation of ion gating. *PLoS One*. 2014;9:e96909

91. Pavlovic D, Hall AR, Kennington EJ, Aughton K, Boguslavskyi A, Fuller W, Despa S, Bers DM, Shattock MJ. Nitric oxide regulates cardiac intracellular Na(+) and Ca(2)(+) by modulating Na/K ATPase via PKCepsilon and phospholemman-dependent mechanism. *J Mol Cell Cardiol*. 2013;61:164-171

92. Pullen MA, Brooks DP, Edwards RM. Characterization of the neutralizing activity of digoxin-specific Fab toward ouabain-like steroids. *J Pharmacol Exp Ther*. 2004;310:319-325

93. Manunta P, Messaggio E, Casamassima N, Gatti G, Carpini SD, Zagato L, Hamlyn JM. Endogenous ouabain in renal Na(+) handling and related diseases. *Biochim Biophys Acta*. 2010;1802:1214-1218

94. Pavlovic D. The role of cardiotonic steroids in the pathogenesis of cardiomyopathy in chronic kidney disease. *Nephron Clin Pract*. 2014;128:11-21

95. Song H, Karashima E, Hamlyn JM, Blaustein MP. Ouabain-digoxin antagonism in rat arteries and neurones. *J Physiol*. 2014;592:941-969

96. Curtis L. Unit Costs of Health & Social Care 2012. *Personal Social Services Research Unit*. 2012:http://www.pssru.ac.uk/archive/pdf/uc/uc2012/full-with-covers.pdf

97. Jerosch-Herold M. Quantification of myocardial perfusion by cardiovascular magnetic resonance. *Journal of Cardiovascular Magnetic Resonance*. 2010;12:57

APPENDIX A – Dosing Schedule (Digoxin)

Randomised treatment arm: Group A

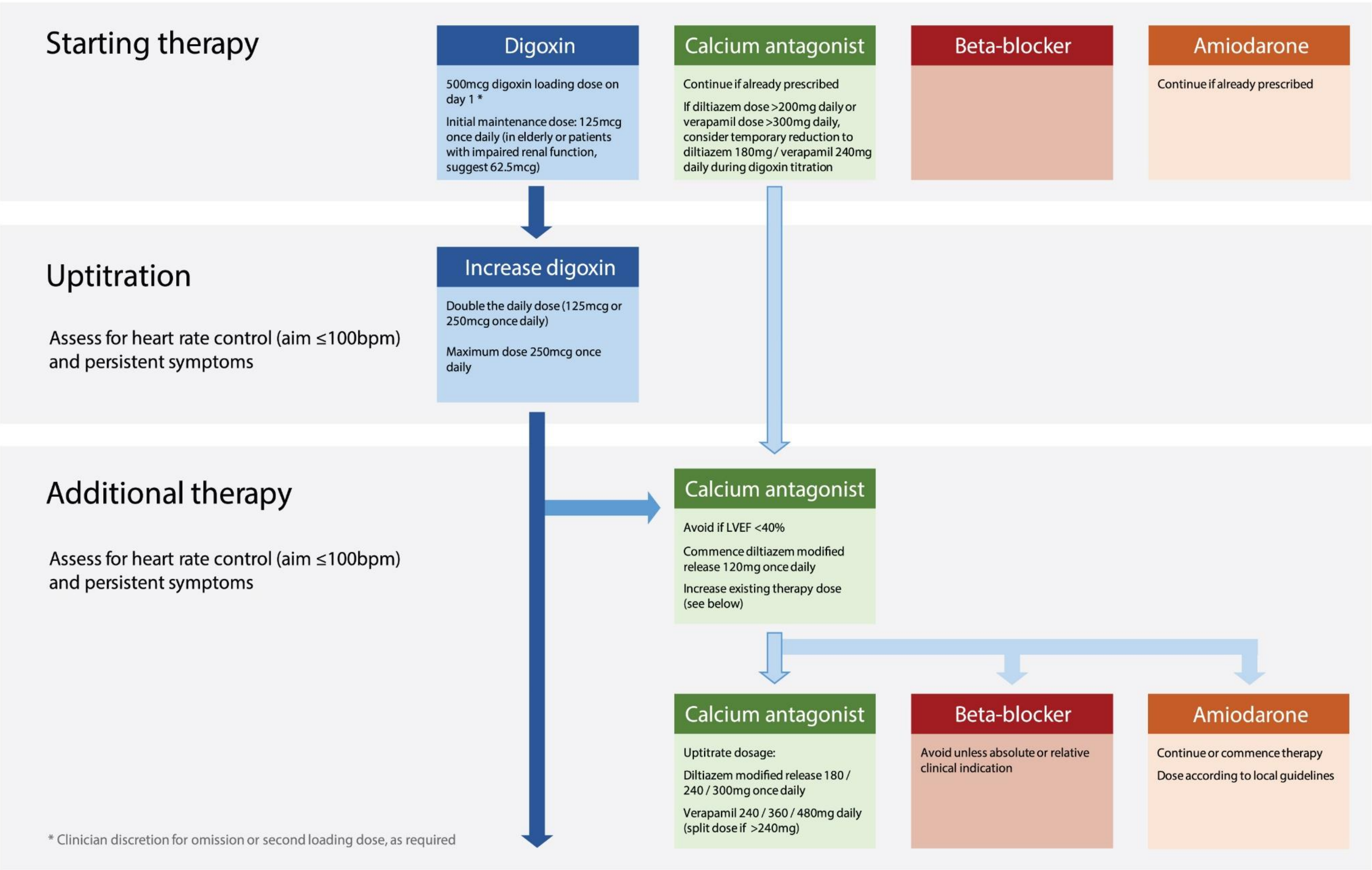

APPENDIX B – Dosing Schedule (Bisoprolol)

Randomised treatment arm: Group B

Starting therapy

Bisoprolol
Start at 1.25/2.5mg according to baseline heart rate

Calcium antagonist
Cease therapy with 24-hour withdrawal period

Digoxin

Amiodarone
Continue if already prescribed

Uptitration

Assess for persistent symptoms and heart rate control (aim ≤100 bpm)

Increase bisoprolol
Uptitrate according to response: 2.5 / 3.75 / 5 / 7.5 / 10 / 15mg

If intolerant of bisoprolol, use alternative beta-blocker (see dosage chart below)

Additional therapy

Assess for heart rate control (aim ≤100bpm) and persistent symptoms

Calcium antagonist
Avoid if LVEF <40%

Commence diltiazem modified release 120mg once daily

Calcium antagonist
Uptitrate dosage:

Diltiazem modified release 180 / 240 / 300mg once daily

Digoxin
Avoid unless absolute or relative clinical indication

Amiodarone
Continue or commence therapy

Dose according to local guidelines

Bisoprolol	Carvedilol	Metoprolol	Nebivolol	Atenolol
1.25 mg od	6.25 mg bd	12.5 mg bd	1.25 mg od	12.5 mg od
2.5 mg od	12.5 mg bd	25 mg bd	2.5 mg od	25 mg od
5 mg od	25 mg bd	50 mg bd	5 mg od	50 mg od
10 mg od	50 mg bd	50 mg tds	10 mg od	100 mg od
