# Supplementary material for: A review of rate control in atrial fibrillation, and the rationale and protocol for the RATE-AF trial
Source: BMJ Open. 2017 Jul 20;7(7):e015099. doi: 10.1136/bmjopen-2016-015099 (PMC5588987; doi:10.1136/bmjopen-2016-015099)
Supplement: Author's manuscript [file bmjopen-2016-015099.draft_revisions.pdf]

BMJ Open

A review of rate control in atrial fibrillation and rationale for the RATE-AF trial

Journal:	*BMJ Open*
Manuscript ID	bmjopen-2016-015099
Article Type:	Protocol
Date Submitted by the Author:	29-Nov-2016
Complete List of Authors:	Kotecha, Dipak; University of Birmingham, Institute of Cardiovascular Sciences; University Hospitals Birmingham NHS Trust, Cardiology<br>Calvert, Melanie; University of Birmingham, Centre for Patient Reported Outcomes Research; University of Birmingham, Institute of Applied Health Research<br>Deeks, Jon; University of Birmingham, Birmingham Clinical Trials Unit; University of Birmingham, Institute of Applied Health Research<br>Griffith, Mike; University Hospitals Birmingham NHS Trust, Cardiology<br>Kirchhof, Paulus; University of Birmingham, Institute of Cardiovascular Sciences; Sandwell & West Birmingham Hospitals NHS Trust, Cardiology<br>Lip, Gregory; University of Birmingham, Institute of Cardiovascular Sciences; Sandwell & West Birmingham Hospitals NHS Trust, Cardiology<br>Mehta, Samir; University of Birmingham, Birmingham Clinical Trials Unit<br>Slinn, Gemma; University of Birmingham, Birmingham Clinical Trials Unit<br>Stanbury, Mary; (Lead for the patient involvement panel)<br>Steeds, Richard; University Hospitals Birmingham NHS Trust, Cardiology; University of Birmingham, Institute of Cardiovascular Sciences<br>Townend, Jonathan; University Hospitals Birmingham NHS Trust, Cardiology; University of Birmingham, Institute of Cardiovascular Sciences
**Primary Subject Heading**:	Cardiovascular medicine
Secondary Subject Heading:	Evidence based practice, Medical management, Patient-centred medicine
Keywords:	Atrial fibrillation, heart rate, RATE-AF trial, quality of life, Echocardiography < CARDIOLOGY, Protocols & guidelines < HEALTH SERVICES ADMINISTRATION & MANAGEMENT

SCHOLARONE™
Manuscripts

Title: A review of rate control in atrial fibrillation and rationale for the RATE-AF trial

Brief Title: Kotecha *et al*, Rate control therapy evaluation in atrial fibrillation

Authors: Dr Dipak Kotecha MBChB PhD MRCP FESC FHEA[1,2,3,4]*, Prof Melanie Calvert BSc PhD FHEA[4,5], Prof Jonathan J Deeks BSc MSc PhD CStat[5,6], Dr Michael Griffith MD FRCP[2], Prof Paulus Kirchhof MD FRCP FESC[1,2,3,4], Prof Gregory YH Lip MD FRCP DFM FESC[1,3,4], Samir Mehta MSc BSc[6], Gemma Slinn BSc MPhil[6], Mary Stanbury RGN RDN RHV**, Dr Richard P Steeds MA MD FRCP FESC[1,2] and Prof Jonathan N Townend BSc MBChB MD FESC[1,2].

From the (1) Institute of Cardiovascular Sciences, University of Birmingham, Birmingham, UK; (2) University Hospitals Birmingham NHS Trust, Birmingham, UK; (3) Sandwell & West Birmingham Hospitals NHS Trust, Birmingham, UK; (4) Centre for Patient Reported Outcomes Research, University of Birmingham, Birmingham, UK; (5) Institute of Applied Health Research, University of Birmingham, Birmingham, UK; and (6) Birmingham Clinical Trials Unit, University of Birmingham, Birmingham, UK. * Authors after the Chief Investigator listed in alphabetical order. ** Lead for the Patient Involvement Panel.

Address for correspondence:

Dr Dipak Kotecha

University of Birmingham Institute of Cardiovascular Sciences, Institute of Translational Medicine, Heritage Building, Queen Elizabeth Hospital Birmingham, B15 2TH, UK.

Email: d.kotecha@bham.ac.uk Tel: +44 121 371 8122 Fax: +44 121 554 4083

Word count (text): 2740 Word count (abstract): 296

Key Words: Atrial fibrillation; heart rate; Protocols & guidelines<health services administration & management; RATE-AF trial; quality of life; echocardiography<cardiology.

For peer review only

Abstract

Background & Objective: Atrial fibrillation (AF) is common and causes impaired quality of life, an increased risk of stroke and death, as well as frequent hospital admissions. The majority of patients with AF require control of heart rate. In this article we summarise the limited evidence from clinical trials that guides prescription, and present the rationale and protocol for a new randomised trial. As rate control has not, as yet, been shown to reduce mortality, there is a clear need to compare the impact of therapy on quality of life, cardiac function and exercise capacity. Such a trial should concentrate on the longer-term effects of treatment in the largest proportion of AF patients, those with symptomatic permanent AF, with the aim of improving patient well-being.

Design & Intervention: The RAte control Therapy Evaluation in permanent Atrial Fibrillation (RATE-AF) trial will enrol 160 participants with a prospective, randomised, open-label, blinded end-point design comparing initial rate control with digoxin or bisoprolol. This will be the first head-to-head randomised trial of digoxin and beta-blockers in AF.

Participants: Recruited patients will be aged ≥60 years with permanent AF and symptoms of breathlessness (NYHA Class II or above), with few exclusion criteria to maximise generalisability to routine clinical practice.

Outcome measures: The primary outcome is patient-reported quality of life, with secondary outcomes including ventricular function using echocardiography, exercise capacity and surrogate biomarkers of cellular and clinical response. Follow-up will occur at 6 and 12 months, with feasibility components to inform the design of a future trial powered to detect a difference in hospital admission. The RATE-AF trial will underpin an integrated approach to management including biomarkers, function and symptoms that will guide future research into optimal, personalised rate control in patients with AF.

Trial registration: clinicaltrials.gov NCT02391337; EudraCT 2015-005043-13; ISRCTN 95259705.

For peer review only

Strengths and limitations of this study

- Control of heart rate is universally used in patients with atrial fibrillation, but evidence from good quality randomised trials is extremely limited.
- Despite common clinical use, there has never been a direct randomised comparison of beta-blockers and digoxin for heart rate control in AF patients (with or without heart failure).
- The RATE-AF trial will assess the effect of therapy on patient-reported quality of life, and improve methods to capture this information in patients with AF. The trial will also evaluate the longer-term impact on cardiac function, define reproducible methods to measure systolic and diastolic function in AF, and develop new biomarkers for personalisation of treatment.
- The trial will not have the power to identify differences in clinical events, but will allow us to plan a future trial designed to detect a difference in the need for admissions to hospital.

For peer review only

Introduction

Atrial fibrillation (AF) is a common cause of stroke and cardiovascular death, leads to poor quality of life and doubles the risk of hospital admission.[1] We are currently in the midst of an epidemic of AF, with both incidence and prevalence expected to double in the next 20 years.[2, 3] Although AF can affect any age-group, patients are typically elderly with significant comorbidities, including up to 50% suffering from heart failure.[4] AF is both a cause and consequence of heart failure, with complex interactions leading to impairment of systolic and diastolic function.[5, 6] The combination of these two conditions is expected to have a dramatic impact on the burden of healthcare worldwide.[7-10]

Management of AF involves anticoagulation to prevent strokes, selecting appropriate patients for restoration of sinus rhythm and almost universal need for control of heart rate. In contrast to other management strategies, the choice of rate control therapy has a very low-quality evidence-base (**Figure 1**).[11] Guidelines from the National Institute for Health and Care Excellence (NICE) and the European Society of Cardiology (ESC) have mandated further research specifically on rate control[1, 12], which is also reflected in the level of recommendations from the American Heart Association.[13] The small studies currently available are often uncontrolled or with short follow-up[14-18], providing few insights on the biological effects of treatment or the mechanisms underpinning the response to therapy. With no evidence for any impact of rate control on mortality[19, 20], and limited data for any difference in quality of life or functional outcomes, the choice of rate control agent is currently informed by expert consensus and physician experience.

In this paper, we review the current evidence-base for rate control in AF and the rationale for a new randomised controlled trial (RCT). The RAte control Therapy Evaluation in permanent Atrial Fibrillation (RATE-AF) trial will compare initial therapy with beta-blockers versus digoxin in older patients with symptomatic permanent AF, assessing quality of life, functional

capacity, left-ventricular ejection fraction (LVEF), diastolic function and biomarkers of treatment response.

Rationale for a new trial of rate control in AF

Why not choose a rhythm control strategy?

A number of RCTs have assessed the addition of rhythm control strategies to control of heart rate in AF patients, most often with anti-arrhythmic drugs (AAD) and direct current cardioversion. Neither of the two largest trials (AFFIRM or RACE) found any difference in mortality, incident stroke or thromboembolism between a rate or rhythm control strategy.[21, 22] A number of meta-analyses have pooled these and other smaller trials and confirmed that rhythm control is not superior to regulation of heart rate alone,[23-25] including heart failure patients with both impaired and preserved ejection fraction.[26, 27] It should be noted that these studies have analysed heterogeneous populations, including both paroxysmal and permanent AF that may differ with regards to mechanism, prognosis and the response to treatment.[14] However there is also evidence that a rhythm control strategy may increase hospital admissions. A meta-analysis of major published trials is presented in **Figure 2**, highlighting a 17% increase in the risk of hospitalisation in the rhythm control group (after exclusion of hospital visits related to cardioversion). Although limited by patient crossover and the association that exists between AAD and adverse events,[28] the results highlight the importance of trials comparing different rate control options and associated healthcare costs.

Although AF ablation is becoming increasingly popular it remains a highly invasive method to restore sinus rhythm.[29, 30] Current European and American guidelines recommend ablation to improve AF-related symptoms in patients with paroxysmal AF, or as a treatment option in symptomatic persistent AF that is refractory to other therapy.[1, 13] Long-term outcome

For peer review only

studies are still awaited and need to be balanced against procedural complications and AF recurrence. Even in patients receiving intensive rhythm control therapy, rate control is often necessary to reduce symptoms during AF paroxysms. Further, 40-50% of AF patients are deemed as unsuitable for rhythm control (permanent AF),[4, 31] and are maintained on rate control therapy to reduce potential symptoms and avoid tachycardia that may worsen ventricular function.[5] Patients with permanent AF have a higher residual risk of cardiovascular death, stroke or systemic embolism, despite anticoagulation.[32]

What is the optimal heart rate target in AF?

There is clinical uncertainty about how to control heart rate and the intensity of rate-reduction. In the RACE II trial of 614 randomised patients with permanent AF, there were no benefits of strict (<80 bpm at rest) compared to lenient rate control (resting heart rate <110 bpm) over 3 years of follow-up.[33] Lenient rate control was non-inferior with an adjusted hazard ratio of 0.80 (90% CI 0.55-1.17) and cumulative adverse clinical outcomes in 12.9%, compared to 14.9% in the strict control arm. In addition, there were no differences in symptoms or NYHA class,[33, 34] with patients who achieved strict rate control requiring more clinic visits.[35] These findings are consistent with other trial data,[36-38] registries,[31] and even observational cohorts in patients with concomitant heart failure,[39] suggesting that intensity of heart rate control *per se* is not the key determinant of outcomes in AF.

Do outcomes vary with different rate control therapies?

Medical therapy to achieve rate control in AF can be achieved with beta-blockers, digoxin and non-dihydropiridine calcium channel blockers (CCB; diltiazem or verapamil).[1] However only a limited evidence-base is available to assist clinicians in choosing appropriate first-line and subsequent therapy. This results in wide variations in local clinical practice,[40-42] and the frequent use of combination therapy. Current European and American guidelines suggest the

For peer review only

choice of medication should be individualised, with dose and use of combination therapy dependent on the presence of ongoing symptoms.[1, 13] However, these recommendations are based on low quality trials and observational data, often with small numbers of participants and follow-up over a few weeks.[15] There are no current randomised trials comparing long-term rate control options in AF.

Demonstrating any reduction in hard clinical outcomes with rate control has proved elusive. In patients with heart failure, reduced ejection fraction and concomitant AF, an individual patient level meta-analysis of all RCT data has shown that beta-blockers do not reduce all-cause mortality or hospital admissions.[19] Similarly, after accounting for the fact that sicker patients tend to receive digoxin more often, the use of digoxin was not associated with any increase, or reduction, in mortality in a comprehensive systematic review.[20] Although digoxin is known to reduce hospital admissions in patients with heart failure and reduced ejection fraction in sinus rhythm[43], the impact in patients with AF is unknown.

If rate control has limited effect on mortality, what about evidence for a differential effect on other outcomes, such as functional capacity, cardiac function or quality of life? Beta-blockers are the most commonly-used rate control agents and although they have a greater impact than digoxin on heart rate during exertion, there is no evidence that this results in better exercise capacity.[16, 17, 44-46] Beta-blockers did not improve arrhythmia-related symptoms in an RCT of 60 low-risk patients with permanent AF, compared to diltiazem and verapamil which reduced the frequency of symptoms.[47] Those in the beta-blocker group had a reduction in exercise capacity on cardio-pulmonary testing and a significant increase in B-type natriuretic peptide (BNP) compared to those treated with CCB.[48] Analysis of smaller trials comparing beta-blockers with CCB are inconsistent.[16] Similarly, compared to verapamil or diltiazem, digoxin has less effect on heart rate but there is no consistent evidence for any difference in functional outcomes.[16, 17, 44, 46, 49] Importantly, diltiazem and verapamil are usually avoided in patients

For peer review only

with reduced ejection fraction due to the risk of adverse outcomes,[50-54] leaving only beta-blockers or digoxin as suitable therapy. Only a single RCT has been published comparing beta-blockers with digoxin in patients with AF and heart failure (mean LVEF 24%, n=47).[55] Although there was a marginally-significant improvement in LVEF with combined carvedilol/digoxin versus placebo/digoxin, blinded withdrawal of digoxin then led to a deterioration in LVEF, accompanied by an increase in BNP. The direct effects of digoxin on LVEF and diastolic function have only been studied in patients with sinus rhythm; in these patients digoxin increased LVEF by 3-11% and improved E/A ratio and mitral deceleration time.[56-58] Magnesium has been shown to successfully complement digoxin therapy to achieve lower ventricular rates in AF patients[59], but is not in common use due to the availability of beta-blockers and CCB which are more potent agents for acute heart rate control.[1] Although data on patient-reported quality of life is limited,[60, 61] rate control has been associated with improved quality of life in trials assessing rate versus rhythm control.[62-64] The mechanism by which rate control therapy mediates an increase in physical functioning and quality of life is unknown but conceivably due to improvements in LVEF and/or diastolic function.

In summary, rate control is an important part of treatment in all AF patients but the evidence-base is poor, particularly in those with permanent AF who form the majority of patients in clinical practice. Rate control in AF is also subject to considerable, and poorly characterised individual variability in response, with limited information about the effects of therapy on cardiac function, quality of life and functional capacity.

For peer review only

The RATE-AF trial

The RATE-AF trial is the first head-to-head randomised assessment of beta-blockers versus digoxin as the initial rate control agent in patients with AF. The trial has a prospective, randomised, open-label, investigator-blinded endpoint (PROBE) design, and is planned as an inclusive study that reflects and will have an important impact on clinical practice (see Information for Patients in **Table 1**). The primary outcome is patient-reported quality of life using the SF-36 physical component summary score at 6 months' post-randomisation. The major secondary outcomes are change in LVEF and diastolic function on echocardiography, functional capacity, global and AF-specific quality of life, and cardiovascular biomarkers (see **Table 2**). A key objective of the trial is to improve the methods used for measuring quality of life in AF patients, as well as optimising the validity, reproducibility and acquisition of echocardiographic left-ventricular function. The RATE-AF trial will also act as a feasibility study to plan a future, event-driven clinical trial exploring the impact of different rate control strategies on cardiovascular events and unplanned hospital admissions. The study is sponsored by the University of Birmingham and funded by the National Institute for Health Research (NIHR), as part of a Career Development Fellowship awarded to the Chief Investigator (DK).

Patients

Inclusion criteria are patients aged 60 years or older with breathlessness (New York Heart Association Class II or more) and permanent AF, characterised as a physician decision for rate control with no plans for cardioversion, AAD or ablation therapy. Only limited exclusion criteria apply (**Figure 3**), reflecting clear requirements or contraindications for either beta-blockers or digoxin. As neither agent impacts on mortality in patients with heart failure[19, 20], reduced LVEF is not an exclusion criterion. All patients are expected to be anticoagulated if appropriate, according to their clinical risk of stroke and thromboembolism.

For peer review only

Study procedures and outcomes

One hundred and sixty eligible patients newly in need of rate control will be invited to participate in the study from primary and secondary care across two major NHS Trusts in Birmingham, UK. The RATE-AF trial is managed by the Birmingham Clinical Trials Unit (BCTU; University of Birmingham) and situated within the Birmingham NIHR/Wellcome Trust Clinical Research Facility.

Following written informed consent, participants will be randomised in a 1:1 ratio to either bisoprolol or digoxin therapy. Stratified randomisation will be provided by a computer-generated minimisation algorithm to ensure balance between the treatment arms for baseline European Heart Rhythm Association (EHRA) class and gender. Allocation will be concealed until the patient has been recruited and consented, thereafter the trial will be open-label. Baseline assessment procedures will include patient-reported quality of life questionnaires (**Table 3**), 6-minute walk distance, echocardiography and biomarker assessment. Participants will then receive study medication (bisoprolol 1.25-15 mg or digoxin 62.5-250 μg once daily), with scheduled uptitration visits to attain a heart rate at rest of ≤100 bpm. Ambulatory 24-hour ECG monitoring will be performed at the end of uptitration (unblinded). Investigator-blinded endpoints will be assessed at the interim (6 month) and final (12 month) visit, which include patient-reported quality of life, echocardiographic parameters of systolic and diastolic left-ventricular function and biomarker assessment (**see Figure 3**).

Exploratory work and clinical practice improvement

During the trial, qualitative research using focus groups and structured interviews will assess whether the quality of life questionnaires adequately and acceptably assess changes in symptom burden in a sample of patients from each treatment arm. The aim of this work is to identify the best processes for measuring patient-reported outcomes in AF, following on from a

For peer review only

systematic review of measurement properties that identified key evidence-gaps.[65]

Optimal acquisition of echocardiography in patients with AF will be determined by reproducibility studies, comparing repeated measures of systolic/diastolic function according to cardiac cycle length. The aim of this work is to produce a standardised protocol of echocardiography in patients with AF.

Blood samples from participants will analysed for the cellular effects of rate control (intracellular sodium, calcium and cardiotonic steroids) using integrated fluorescence/contractility photometry in human cardiomyocytes. This work will give mechanistic insight into the cellular response to beta-blockers and digoxin, and identify novel markers of treatment effect. Serum will also be stored for the development of new blood-based and genetic biomarkers that aid in personalisation of rate control therapy.

Statistical considerations

The null hypothesis is of no difference in the physical functioning domain of the SF-36 quality of life questionnaire when comparing a strategy of digoxin versus beta-blocker therapy for initial rate control in older patients with permanent AF. The alternative hypothesis is superiority of one over the other therapy as an initial strategy of care. Randomising 144 patients we can assume an 85% power to detect an effect size of half a standard deviation in a continuous outcome measure of quality of life (two-sided alpha of 0.05). Assuming that 10% of patients will be lost to follow-up, 160 patients are needed. There is some evidence from existing research to support the notion that the treatment effect could be this large. This includes a 17% improvement in SF-36 role-physical score in the rate control arm of the RACE study,[63] a 22% improvement in a proprietary symptom-checklist with CCB (compared to 8% change in those assigned beta-blockers),[18] and 17% improvement with rate control using SF-36 in the PIAF trial.[64] The RATE-AF trial will also us to explore surrogates for clinical outcomes, such as LVEF using echocardiography and B-type natriuretic peptide, and provide estimates

For peer review only

for a future definitive trial of rate control in AF, including reliable information on recruitment rates, study drug titration, cross-over, retention and healthcare costs.

Trial oversight, management and registration

The trial has ethical approval from the East Midlands - Derby Research Ethics Committee (16/EM/0178), and regulatory approval from the Medicines and Healthcare products Regulatory Agency (MHRA). RATE-AF will be conducted in accordance with guidelines for Good Clinical Practice (GCP) and the Declaration of Helsinki.

Oversight will be provided by a Trial Steering Committee, comprising an independent Data Monitoring Committee and members of the RATE-AF Trial Management Group. This includes representatives of the patient and public involvement panel, involved in both the design and management of the trial. A Clinical Events Committee will be formed to adjudicate on adverse events.

The RATE-AF trial is registered at clinicaltrials.gov (NCT02391337) and EudraCT (2015-005043-13). Further information can be obtained from the trial website, http://www.birmingham.ac.uk/rate-af, and the trial protocol (see **Appendix**). The protocol was developed in accordance with the Standard Protocol Items for Randomized Trials [SPIRIT] statement[66], and the latest guidance from the International Society for Quality of Life Research (ISOQOL) Best Practice taskforce.[67-69]

For peer review only

Conclusion

Defining appropriate rate control therapy is vital, particularly in the rapidly growing number of older patients with permanent AF where current evidence is extremely limited. Rate control is an integral part of management in almost all AF patients but hardly any controlled trial evidence exists to guide the choice of agents. This is unacceptable in light of the potential benefits and possible adverse effects of treatment. In addition, the complete lack of data on the impact of medical therapy on symptom burden and heart function necessitate a programme of reproducibility and validity of both patient-reported quality of life and cardiac imaging in AF. The RATE-AF trial will answer key clinical questions about how to initiate therapy in order to improve patient well-being, stratified by relevant patient characteristics such as baseline symptoms, systolic and diastolic cardiac function, and biomarkers of treatment effect.

For peer review only

Acknowledgements

We would like to acknowledge other members of the wider RATE-AF team, including Karina Bunting, Margaret Grant, Hannah Lack, Susan Jowett, Jonathan Mathers, and Davor Pavlovic (University of Birmingham). We are indebted to the independent members of the trial oversight committees, as well as the Patient and Public Involvement (PPI) Team.

Competing interests

None of the authors report a conflict of interest. All authors have completed the ICMJE uniform disclosure form (www.icmje.org/coi_disclosure.pdf) and declare:

DK reports grants from Menarini, during the conduct of the study; non-financial support from Daiichi Sankyo and personal fees from AtriCure, outside the submitted work. MC reports grants from the National Institute of Health Research, during the conduct of the study; and personal fees from Astella Pharma and Ferring Pharma, outside the submitted work. PK reports consulting fees and honoraria from Bayer Healthcare, Boehringer Ingelheim, Bristol-Myers Squibb, Daiichi-Sankyo, Medtronic, Pfizer and Servier, all outside the submitted work; research grants from Bristol-Myers Squibb, Pfizer, Cardiovascular Therapeutics, Daiichi Sankyo, Sanofi, St. Jude Medical, German Federal Ministry for Education and Research (BMBF), Fondation Leducq, German Research Foundation (DFG), European Union, British Heart Foundation and Medical Research Council UK, all outside the submitted work; and is listed on two patent applications on AF therapy and markers for AF, both outside the submitted work. GYHL has served as a consultant for Bayer, Astellas, Merck, AstraZeneca, Sanofi, BMS/Pfizer, Biotronik, Portola and Boehringer Ingelheim, and has been on the speaker's bureau for Bayer, BMS/Pfizer, Boehringer Ingelheim, and Sanofi-Aventis. RPS is the President of the British Society of Echocardiography. JJD, MG, MS. JNT, SM, GS report no competing interests.

For peer review only

Authors' contributions

The manuscript was drafted by DK who is the Chief Investigator for the RATE-AF trial. MG and GYHL are Principal Investigators. MC, PK, RPS and JNT are members of the Trial Management Group. JJD, SM and GS are representatives from the Clinical Trials Unit. MS is the Lead for the Patient Involvement Panel, and a member of the Steering Committee. All authors contributed to the writing of the RATE-AF protocol and patient information, and edited this manuscript for intellectual content.

Funding

DK and the RATE-AF trial are supported by the National Institute of Health Research (NIHR) as part of a Career Development Fellowship (CDF-2015-08-074). The opinions expressed in this paper are those of the authors and do not represent the NIHR or the UK Department of Health.

Data Sharing Statement

No additional data available.

For peer review only

References

1. **Kirchhof P, Benussi S, Kotecha D, *et al.*** 2016 ESC Guidelines for the management of atrial fibrillation developed in collaboration with EACTS: The Task Force for the management of atrial fibrillation of the European Society of Cardiology (ESC). Developed with the special contribution of the European Heart Rhythm Association (EHRA) of the ESC. Endorsed by the European Stroke Organisation (ESO). *Eur Heart J*. 2016;10.1093/eurheartj/ehw210

2. **Chugh SS, Havmoeller R, Narayanan K, *et al.*** Worldwide epidemiology of atrial fibrillation: a Global Burden of Disease 2010 Study. *Circulation*. 2014;**129**:837-847

3. **Krijthe BP, Kunst A, Benjamin EJ, *et al.*** Projections on the number of individuals with atrial fibrillation in the European Union, from 2000 to 2060. *Eur Heart J*. 2013;**34**:2746-2751

4. **Chiang CE, Naditch-Brule L, Murin J, *et al.*** Distribution and risk profile of paroxysmal, persistent, and permanent atrial fibrillation in routine clinical practice: insight from the real-life global survey evaluating patients with atrial fibrillation international registry. *Circ Arrhythm Electrophysiol*. 2012;**5**:632-639

5. **Kotecha D, Piccini JP**. Atrial fibrillation in heart failure: what should we do? *Eur Heart J*. 2015;**36**:3250-3257

6. **Kotecha D, Lam CS, Van Veldhuisen DJ, *et al.*** Heart failure with preserved ejection fraction and atrial fibrillation - Vicious twins. *J Am Coll Cardiol*. 2016;**68**:2217-2228

7. **Kotecha D, Banerjee A, Lip GY**. Increased stroke risk in atrial fibrillation patients with heart failure: does ejection fraction matter? *Stroke*. 2015;**46**:608-609

8. **Christiansen CB, Olesen JB, Gislason G, *et al.*** Cardiovascular and non-cardiovascular hospital admissions associated with atrial fibrillation: a Danish nationwide, retrospective cohort study. *BMJ Open*. 2013;**3**

9. **Ambrosy AP, Fonarow GC, Butler J, *et al.*** The global health and economic burden of hospitalizations for heart failure: lessons learned from hospitalized heart failure registries. *J Am Coll Cardiol*. 2014;**63**:1123-1133

For peer review only

10. **Kotecha D, Chudasama R, Lane DA, *et al.*** Atrial fibrillation and heart failure due to reduced versus preserved ejection fraction: A systematic review and meta-analysis of death and adverse outcomes. *Int J Cardiol*. 2016;**203**:660-666

11. **Kirchhof P, Breithardt G, Bax J, *et al.*** A roadmap to improve the quality of atrial fibrillation management: proceedings from the fifth Atrial Fibrillation Network/European Heart Rhythm Association consensus conference. *Europace*. 2016;**18**:37-50

12. **National Institute for Health and Care Excellence**. Atrial fibrillation: the management of atrial fibrillation. *NICE clinical guideline 180*. 2014; **Accessed 15/09/2014; http://www.nice.org.uk/guidance/cg180/**

13. **January CT, Wann LS, Alpert JS, *et al.*** 2014 AHA/ACC/HRS guideline for the management of patients with atrial fibrillation: executive summary: a report of the American College of Cardiology/American Heart Association Task Force on practice guidelines and the Heart Rhythm Society. *Circulation*. 2014;**130**:2071-2104

14. **Kotecha D, Kirchhof P**. Rate and rhythm control have comparable effects on mortality and stroke in atrial fibrillation but better data are needed. *Evid Based Med*. 2014;**19**:222-223

15. Segal JB, McNamara RL, Miller MR, *et al.* The evidence regarding the drugs used for ventricular rate control. *J Fam Practice*. 2000;49:47-59

16. **Nikolaidou T, Channer KS**. Chronic atrial fibrillation: a systematic review of medical heart rate control management. *Postgrad Med J*. 2009;**85**:303-312

17. **Farshi R, Kistner D, Sarma JSM, *et al.*** Ventricular rate control in chronic atrial fibrillation during daily activity and programmed exercise: a crossover open-label study of five drug regimens. *J Am Coll Cardiol*. 1999;**33**:304-310

18. **Ulimoen SR, Enger S, Carlson J, *et al.*** Comparison of four single-drug regimens on ventricular rate and arrhythmia-related symptoms in patients with permanent atrial fibrillation. *Am J Cardiol*. 2013;**111**:225-230

19. **Kotecha D, Holmes J, Krum H, *et al.*** Efficacy of beta blockers in patients with heart failure plus atrial fibrillation: an individual-patient data meta-analysis. *Lancet*. 2014;**384**:2235-2243

For peer review only

20. **Ziff OJ, Lane DA, Samra M, *et al.*** Safety and efficacy of digoxin: systematic review and meta-analysis of observational and controlled trial data. *BMJ*. 2015;**351**:h4451

21. **Wyse DG, Waldo AL, DiMarco JP, *et al.*** A comparison of rate control and rhythm control in patients with atrial fibrillation. *N Engl J Med*. 2002;**347**:1825-1833

22. **Van Gelder IC, Hagens VE, Bosker HA, *et al.*** A Comparison of Rate Control and Rhythm Control in Patients with Recurrent Persistent Atrial Fibrillation. *N Engl J Med*. 2002;**347**:1834-1840

23. **de Denus S, Sanoski CA, Carlsson J, *et al.*** Rate vs rhythm control in patients with atrial fibrillation: a meta-analysis. *Arch Intern Med*. 2005;**165**:258-262

24. **Chatterjee S, Sardar P, Lichstein E, *et al.*** Pharmacologic rate versus rhythm-control strategies in atrial fibrillation: an updated comprehensive review and meta-analysis. *PACE*. 2013;**36**:122-133

25. **Al-Khatib SM, Allen LaPointe NM, Chatterjee R, *et al.*** Rate- and rhythm-control therapies in patients with atrial fibrillation: a systematic review. *Ann Intern Med*. 2014;**160**:760-773

26. **Roy D, Talajic M, Nattel S, *et al.*** Rhythm Control versus Rate Control for Atrial Fibrillation and Heart Failure. *N Engl J Med*. 2008;**358**:2667-2677

27. **Kong MH, Shaw LK, O'Connor C, *et al.*** Is rhythm-control superior to rate-control in patients with atrial fibrillation and diastolic heart failure? *Ann Noninvasive Electrocardiol*. 2010;**15**:209-217

28. **Corley SD, Epstein AE, DiMarco JP, *et al.*** Relationships between sinus rhythm, treatment, and survival in the Atrial Fibrillation Follow-Up Investigation of Rhythm Management (AFFIRM) Study. *Circulation*. 2004;**109**:1509-1513

29. **Wazni O, Wilkoff B, Saliba W**. Catheter Ablation for Atrial Fibrillation. *N Engl J Med*. 2011;**365**:2296-2304

30. **Jones DG, Haldar SK, Hussain W, *et al.*** A randomized trial to assess catheter ablation versus rate control in the management of persistent atrial fibrillation in heart failure. *J Am Coll Cardiol*. 2013;**61**:1894-1903

31. **Kirchhof P, Ammentorp B, Darius H, *et al.*** Management of atrial fibrillation in seven

For peer review only

European countries after the publication of the 2010 ESC Guidelines on atrial fibrillation: primary results of the PREvention oF thromboemolic events--European Registry in Atrial Fibrillation (PREFER in AF). *Europace*. 2014;**16**:6-14

32. **Senoo K, Lip GY, Lane DA, *et al.*** Residual risk of stroke and death in anticoagulated patients according to the type of atrial fibrillation: AMADEUS Trial. *Stroke*. 2015;**46**:2523-2528

33. **Van Gelder IC, Groenveld HF, Crijns HJGM, *et al.*** Lenient versus Strict Rate Control in Patients with Atrial Fibrillation. *N Engl J Med*. 2010;**362**:1363-1373

34. **Groenveld HF, Crijns HJGM, Van den Berg MP, *et al.*** The Effect of Rate Control on Quality of Life in Patients With Permanent Atrial Fibrillation: Data From the RACE II (Rate Control Efficacy in Permanent Atrial Fibrillation II) Study. *J Am Coll Cardiol*. 2011;**58**:1795-1803

35. **Groenveld HF, Tijssen JG, Crijns HJ, *et al.*** Rate control efficacy in permanent atrial fibrillation: successful and failed strict rate control against a background of lenient rate control: data from RACE II (Rate Control Efficacy in Permanent Atrial Fibrillation). *J Am Coll Cardiol*. 2013;**61**:741-748

36. **Van Gelder IC, Wyse DG, Chandler ML, *et al.*** Does intensity of rate-control influence outcome in atrial fibrillation? An analysis of pooled data from the RACE and AFFIRM studies. *Europace*. 2006;**8**:935-942

37. **Cooper HA, Bloomfield DA, Bush DE, *et al.*** Relation between achieved heart rate and outcomes in patients with atrial fibrillation (from the Atrial Fibrillation Follow-up Investigation of Rhythm Management [AFFIRM] Study). *Am J Cardiol*. 2004;**93**:1247-1253

38. **Groenveld HF, Crijns HJ, Rienstra M, *et al.*** Does intensity of rate control influence outcome in persistent atrial fibrillation? Data of the RACE study. *Am Heart J*. 2009;**158**:785-791

39. **Cullington D, Goode KM, Zhang J, *et al.*** Is heart rate important for patients with heart failure in atrial fibrillation? *JACC Heart Fail*. 2014;**2**:213-220

40. **Steg PG, Alam S, Chiang C-E, *et al.*** Symptoms, functional status and quality of life in patients with controlled and uncontrolled atrial fibrillation: data from the RealiseAF

For peer review only

cross-sectional international registry. *Heart*. 2012;**98**:195-201

41. **Nabauer M, Gerth A, Limbourg T, *et al.*** The Registry of the German Competence NETwork on Atrial Fibrillation: patient characteristics and initial management. *Europace*. 2009;**11**:423-434

42. **Lip GY, Laroche C, Dan GA, *et al.*** A prospective survey in European Society of Cardiology member countries of atrial fibrillation management: baseline results of EURObservational Research Programme Atrial Fibrillation (EORP-AF) Pilot General Registry. *Europace*. 2014;**16**:308-319

43. **The Digitalis Investigation Group**. The effect of digoxin on mortality and morbidity in patients with heart failure. *N Engl J Med*. 1997;**336**:525-533

44. **Koh KK, Kwon KS, Park HB, *et al.*** Efficacy and safety of digoxin alone and in combination with low-dose diltiazem or betaxolol to control ventricular rate in chronic atrial fibrillation. *Am J Cardiol*. 1995;**75**:88-90

45. **Lewis RV, McMurray J, McDevitt DG**. Effects of atenolol, verapamil, and xamoterol on heart rate and exercise tolerance in digitalised patients with chronic atrial fibrillation. *J Cardiovasc Pharmacol*. 1989;**13**:1-6

46. **Tsuneda T, Yamashita T, Fukunami M, *et al.*** Rate control and quality of life in patients with permanent atrial fibrillation: the Quality of Life and Atrial Fibrillation (QOLAF) Study. *Circ J*. 2006;**70**:965-970

47. **Ulimoen SR, Enger S, Carlson J, *et al.*** Comparison of four single-drug regimens on ventricular rate and arrhythmia-related symptoms in patients with permanent atrial fibrillation. *Am J Cardiol*. 2013;**111**:225-230

48. **Ulimoen SR, Enger S, Pripp AH, *et al.*** Calcium channel blockers improve exercise capacity and reduce N-terminal Pro-B-type natriuretic peptide levels compared with beta-blockers in patients with permanent atrial fibrillation. *Eur Heart J*. 2014;**35**:517-524

49. **Lewis RV, Irvine N, McDevitt DG**. Relationships between heart rate, exercise tolerance and cardiac output in atrial fibrillation: the effects of treatment with digoxin, verapamil and diltiazem. *Eur Heart J*. 1988;**9**:777-781

For peer review only

50. **McMurray JJ, Adamopoulos S, Anker SD*, et al.*** ESC Guidelines for the diagnosis and treatment of acute and chronic heart failure 2012: The Task Force for the Diagnosis and Treatment of Acute and Chronic Heart Failure 2012 of the European Society of Cardiology. Developed in collaboration with the Heart Failure Association (HFA) of the ESC. *Eur Heart J*. 2012;**33**:1787-1847

51. **Elkayam U**. Calcium channel blockers in heart failure. *Cardiology*. 1998;**89 Suppl 1**:38-46

52. The effect of diltiazem on mortality and reinfarction after myocardial infarction. The Multicenter Diltiazem Postinfarction Trial Research Group. *N Engl J Med*. 1988;**319**:385-392

53. **Goldstein RE, Boccuzzi SJ, Cruess D*, et al.*** Diltiazem increases late-onset congestive heart failure in postinfarction patients with early reduction in ejection fraction. *Circulation*. 1991;**83**:52-60

54. **The Danish Study Group on Verapamil in Myocardial Infarction**. Secondary prevention with verapamil after myocardial infarction. *Am J Cardiol*. 1990;**66**:33-40

55. **Khand AU, Rankin AC, Martin W*, et al.*** Carvedilol alone or in combination with digoxin for the management of atrial fibrillation in patients with heart failure? *J Am Coll Cardiol*. 2003;**42**:1944-1951

56. **Partanen J, Heikkila J, Pellinen T*, et al.*** Effect of digoxin on the heart in normal subjects: influence of isometric exercise and autonomic blockade: a noninvasive study. *Br J Clin Pharmacol*. 1988;**25**:331-340

57. **Dernellis JM, Panaretou MP**. Effects of digoxin on left atrial function in heart failure. *Heart*. 2003;**89**:1308-1315

58. **Giunta A, Maione S, Arnese MR*, et al.*** Effects of intravenous digoxin on pulmonary venous and transmitral flows in patients with chronic heart failure of different degrees. *Clin Cardiol*. 1995;**18**:27-33

59. **Kotecha D**. Magnesium for Atrial Fibrillation, Myth or Magic? *Circ Arrhythm Electrophysiol*. 2016;10.1161/circep.116.004521

60. **Thrall G, Lane D, Carroll D*, et al.*** Quality of Life in Patients with Atrial Fibrillation:

For peer review only

A Systematic Review. *Am J Med*. 2006;**119**:448.e441-419

61. **Rienstra M, Lubitz SA, Mahida S, *et al.*** Symptoms and Functional Status of Patients With Atrial Fibrillation: State of the Art and Future Research Opportunities. *Circulation*. 2012;**125**:2933-2943

62. **Pepine CJ**. Effects of pharmacologic therapy on health-related quality of life in elderly patients with atrial fibrillation: a systematic review of randomized and nonrandomized trials. *Clin Med Insights Cardiol*. 2013;**7**:1-20

63. **Hagens VE, Ranchor AV, Van Sonderen E, *et al.*** Effect of rate or rhythm control on quality of life in persistent atrial fibrillation: Results from the Rate Control Versus Electrical Cardioversion (RACE) study. *J Am Coll Cardiol*. 2004;**43**:241-247

64. **Grönefeld GC, Lilienthal J, Kuck K-H, *et al.*** Impact of rate versus rhythm control on quality of life in patients with persistent atrial fibrillation: Results from a prospective randomized study. *Eur Heart J*. 2003;**24**:1430-1436

65. **Kotecha D, Ahmed A, Calvert M, *et al.*** Patient-Reported Outcomes for Quality of Life Assessment in Atrial Fibrillation: A Systematic Review of Measurement Properties. *PLoS ONE*. 2016;**11**:e0165790

66. **Chan AW, Tetzlaff JM, Gotzsche PC, *et al.*** SPIRIT 2013 explanation and elaboration: guidance for protocols of clinical trials. *BMJ*. 2013;**346**:e7586

67. **Calvert M, Kyte D, von Hildebrand M, *et al.*** Putting patients at the heart of health-care research. *Lancet*. 2015;**385**:1073-1074

68. **Calvert M, Kyte D, Duffy H, *et al.*** Patient-reported outcome (PRO) assessment in clinical trials: a systematic review of guidance for trial protocol writers. *PLoS One*. 2014;**9**:e110216

69. **Kyte D, Duffy H, Fletcher B, *et al.*** Systematic evaluation of the patient-reported outcome (PRO) content of clinical trial protocols. *PLoS One*. 2014;**9**:e110229

70. **Ware JE, Gandek B**. Overview of the SF-36 Health Survey and the International Quality of Life Assessment (IQOLA) Project. *J Clin Epidemiol*. 1998;**51**:903-912

71. **Gandek B, Sinclair SJ, Kosinski M, *et al.*** Psychometric evaluation of the SF-36 health survey in Medicare managed care. *Health Care Financ Rev*. 2004;**25**:5-25

For peer review only

72. **Herdman M, Gudex C, Lloyd A, *et al.*** Development and preliminary testing of the new five-level version of EQ-5D (EQ-5D-5L). *Qual Life Res*. 2011;**20**:1727-1736

73. **Devlin NJ, Krabbe PF**. The development of new research methods for the valuation of EQ-5D-5L. *Eur J Health Econ*. 2013;**14 Suppl 1**:S1-3

74. **Janssen MF, Pickard AS, Golicki D, *et al.*** Measurement properties of the EQ-5D-5L compared to the EQ-5D-3L across eight patient groups: a multi-country study. *Qual Life Res*. 2013;**22**:1717-1727

75. **Spertus J, Dorian P, Bubien R, *et al.*** Development and validation of the Atrial Fibrillation Effect on QualiTy-of-Life (AFEQT) Questionnaire in patients with atrial fibrillation. *Circ Arrhythm Electrophysiol*. 2011;**4**:15-25

76. **Dorian P, Burk C, Mullin CM, *et al.*** Interpreting changes in quality of life in atrial fibrillation: How much change is meaningful? *Am Heart J*. 2013;**166**:381-387.e388

77. **Wynn GJ, Todd DM, Webber M, *et al.*** The European Heart Rhythm Association symptom classification for atrial fibrillation: validation and improvement through a simple modification. *Europace*. 2014;**16**:965-972

78. **Carlsson J, Miketic S, Windeler J, *et al.*** Randomized trial of rate-control versus rhythm-control in persistent atrial fibrillation: the Strategies of Treatment of Atrial Fibrillation (STAF) study. *J Am Coll Cardiol*. 2003;**41**:1690-1696

79. **Hohnloser SH, Kuck KH, Lilienthal J**. Rhythm or rate control in atrial fibrillation--Pharmacological Intervention in Atrial Fibrillation (PIAF): a randomised trial. *Lancet*. 2000;**356**:1789-1794

80. **Opolski G, Torbicki A, Kosior DA, *et al.*** Rate control vs rhythm control in patients with nonvalvular persistent atrial fibrillation: the results of the Polish How to Treat Chronic Atrial Fibrillation (HOT CAFE) Study. *Chest*. 2004;**126**:476-486

81. **Vora A, Karnad D, Goyal V, *et al.*** Control of heart rate versus rhythm in rheumatic atrial fibrillation: a randomized study. *J Cardiovasc Pharmacol Ther*. 2004;**9**:65-73

For peer review only

Table 1: The RATE-AF trial – Information for Patients

About atrial fibrillation
Atrial fibrillation is a common heart condition that leads to an irregular and often rapid heart rate. Atrial fibrillation causes 1 in 4 strokes, and patients have frequent hospital admissions and a higher risk of dying. In addition, atrial fibrillation makes many patients feel unwell, with reduced quality of life.
**What is the purpose of the trial?**
Atrial fibrillation usually requires medication to control heart rate, but we currently don't know which medication is better for patients. The aim of this study is to find out which of two treatments improves quality of life and the function of the heart, digoxin or bisoprolol (a beta-blocker).
**What will happen in the trial?**
The RATE-AF trial is designed to compare two approaches for control of heart rate, based on initial treatment with either digoxin or beta-blockers, medications which are commonly used by doctors. The main objective of the trial is to research the effects of treatment on quality of life in patients with atrial fibrillation. We will also test whether quality of life questionnaires respond to changes in symptoms experienced by patients, how we use ultrasound to look at the function of the heart, and develop new markers in the blood to personalise treatment.
**More information**
RATE-AF trial video: https://www.youtube.com/watch?v=4oxe8AcVo0E<br>Patient information (British Heart Foundation): https://www.bhf.org.uk/heart-health/conditions/atrial-fibrillation

For peer review only

Table 2: Outcomes and objectives of the RATE-AF trial

**Primary outcome:**
Comparison of two strategies for rate control on patient-reported quality of life, based on initial use of digoxin versus beta-blocker therapy, with a predefined focus on physical well-being using the SF-36 physical component summary at six months.
**Secondary outcomes:**
Patient-reported quality of life at six and twelve months, including SF-36 global and domain-specific scores, EQ-5D-5L summary index and visual analogue scale, and AFEQT overall score.
Echocardiographic left-ventricular function at 12 months, including LVEF and diastolic function (E/e' and composite of diastolic indices).
Functional assessment at 6 and 12 months, including six-minute walking distance and change in EHRA class.
Change in BNP levels at 6 months.
Change in heart rate from baseline and group comparison using 24-hour ambulatory ECG at end of uptitration.
**Feasibility assessment:**
Successful methods for recruitment across primary and secondary care.
Key issues that affect retention of participants, such as convenience, compliance and cross-over.
Drug discontinuation rate and adverse reactions leading to drug discontinuation.
Therapy-induced requirement for additional treatment (e.g. pacemaker implantation).
Population-specific standard deviations and proportions to enable sample size calculation for a future trial.
Assessment of unplanned hospital admissions and cardiovascular outcomes.
**Exploratory objectives:**
Correlation of baseline measures, including quality of life questionnaires and unblinded baseline investigations such as quality of life, BNP, LVEF, E/e', EHRA class, intracellular biomarkers and heart rate.
Impact of therapy on intracellular sodium and calcium concentration and cardiotonic steroid levels as biomarkers of cellular response at six and twelve months.
Impact of combination therapy on outcomes.
Change in cognitive function at twelve months.
Qualitative research of patient-reported quality of life using focus groups to explore patient acceptability, optimal delivery methods and responsiveness.
Assessment of the validity and reproducibility of echocardiographic measures in patients with AF.
Correlation of serum digoxin concentration with change in quality of life and intracellular methods.
Cost-consequence economic analysis from an NHS healthcare perspective.

AF, Atrial Fibrillation; AFEQT, Atrial Fibrillation Effect on QualiTy-of-life questionnaire; BNP, B-type natriuretic peptide; ECG, electrocardiogram; EHRA, European Heart Rhythm Association functional class; EQ-5D-5L, EuroQol five dimensions five level questionnaire; LVEF, left ventricular ejection fraction; NHS, National Health Service; SF-36, Short Form (36) Health Survey.

For peer review only

Table 3: Patient-reported quality of life questionnaires used in RATE-AF

Questionnaire	Details	Advantages and disadvantages
SF-36<br>Short Form (36) Health Survey [70]	Generic instrument with 4-week recall period in eights domains (vitality, physical functioning, bodily pain, general health perceptions, physical role functioning, emotional role functioning, social role functioning and mental health).<br>11 subdivided questions, each scored with a Likert scale.	Extensively validated across a wide variety of conditions and the elderly.[71]<br>Not specific to AF and hence other comorbidities may dominate responses.<br>Requires a license fee.
EQ-5D-5L<br>EuroQol five dimensions five level questionnaire [72, 73]	Generic instrument about today's health with a five-answer scale in five domains (mobility, self-care, usual activities, pain/discomfort and anxiety/depression).<br>Also includes a visual analogue scale denoting current health perception on a 0 to 100 scale.	Simple questionnaire that is quick to complete and includes a visual scale.<br>Extensive utilisation, particularly for heath economic assessment, with improvement discrimination over prior versions.[74]<br>Not specific to AF and hence other comorbidities may dominate responses.
AFEQT<br>Atrial Fibrillation Effect on QualiTy-of-life questionnaire [75]	AF-specific quality of life instrument with 4-week recall period in domains relating to symptoms, daily activities and concerns/satisfaction with current treatment.<br>20 questions, each scored with a 7-point Likert scale.	Specific to the impact of AF on quality of life.<br>Better than other AF-specific tools using methodological/psychometric assessment.[65]<br>Limited validation as yet in comparison to generic tools[76, 77], particularly for clinical responsiveness.<br>License fee may apply.

For peer review only

Figure legends

Figure 1: Evidenced-based summary for management of AF

Summary of evidence for main components of clinical management, highlighting paucity of robust data for key issues regarding rate control therapy. RCT, randomised controlled trial; LV, left-ventricular; NOAC, novel oral anticoagulants.

Figure 2: Hospitalisation in rate versus rhythm control trials

Meta-analysis of hospitalisation in the six largest rate versus rhythm control trials, excluding hospital visits for cardioversion procedures, where applicable. Studies are pooled with a random-effects model. Significant heterogeneity was identified, with an I^2 value of 66.8% (p=0.01). Grey boxes represent the comparative weight of the study.

STAF, Strategies of Treatment of Atrial Fibrillation study (cardioversion/AAD versus rate control in persistent AF)[78]; PIAF, Pharmacological Intervention in Atrial Fibrillation trial (amiodarone/cardioversion versus diltiazem in persistent AF)[79]; HOT CAFE, How to Treat Chronic Atrial Fibrillation study (cardioversion/AAD versus rate control in persistent AF)[80]; AF-CHF, Atrial Fibrillation and Congestive Heart Failure trial (cardioversion/AAD versus rate control in paroxysmal/persistent AF with LVEF ≤35%)[26]; CRAAFT, Control of Rate versus Rhythm in rheumatic Atrial Fibrillation Trial (cardioversion/amiodarone versus diltiazem in persistent AF due to rheumatic heart disease)[81]; AFFIRM, Atrial Fibrillation Follow-up Investigation of Rhythm Management study (AAD/cardioversion versus rate control in paroxysmal/persistent AF).[21]

Figure 3: RATE-AF trial schema

Trial flowchart, including major endpoints and inclusion/exclusion criteria.

For peer review only

Figure 1: Evidenced-based summary for management of atrial fibrillation

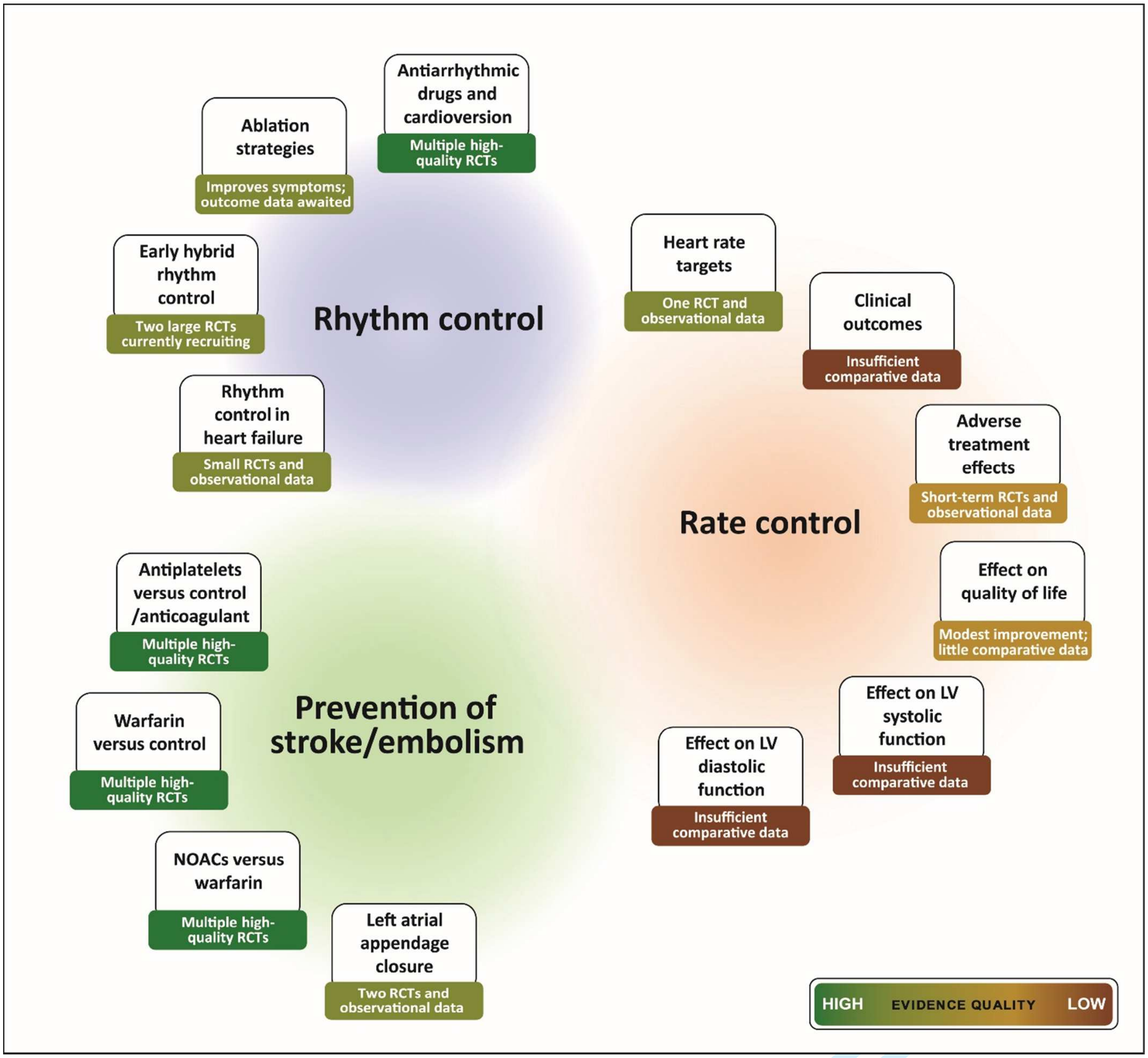

Figure 2: Hospitalisation in major trials of rate versus rhythm control

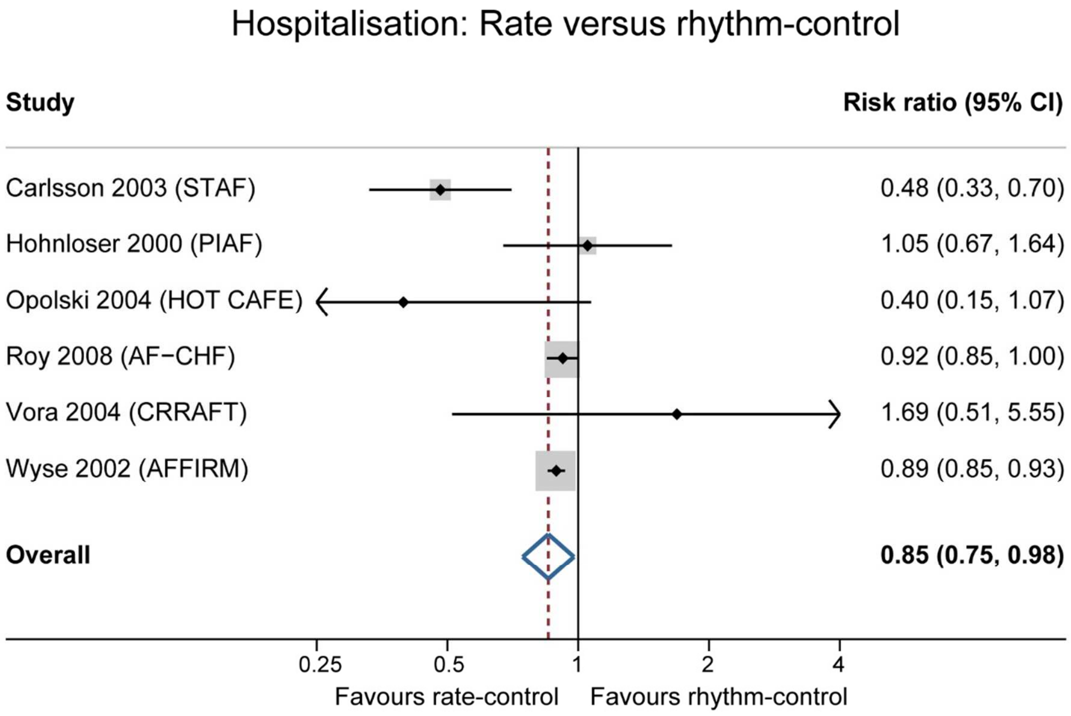

view only

Figure 3: RATE-AF trial schema

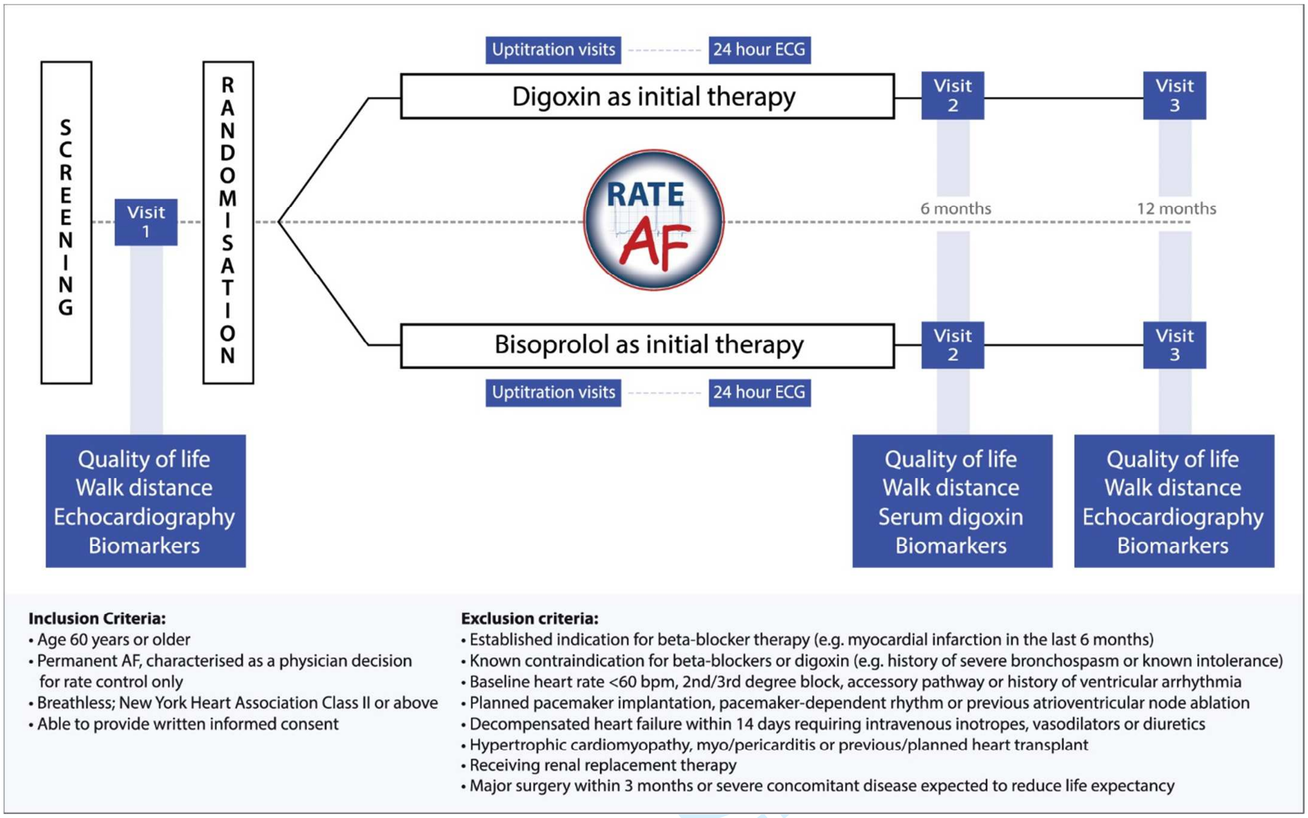

Appendix: RATE-AF trial protocol

Please see attached file.

For peer review only

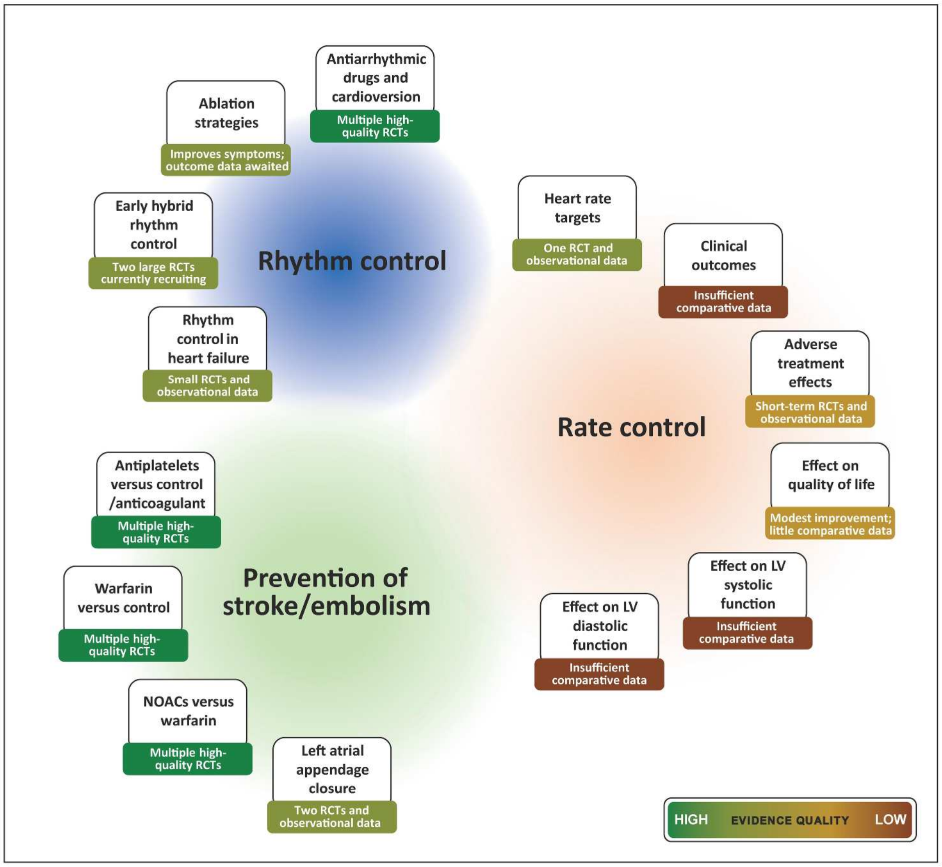

287x264mm (300 x 300 DPI)

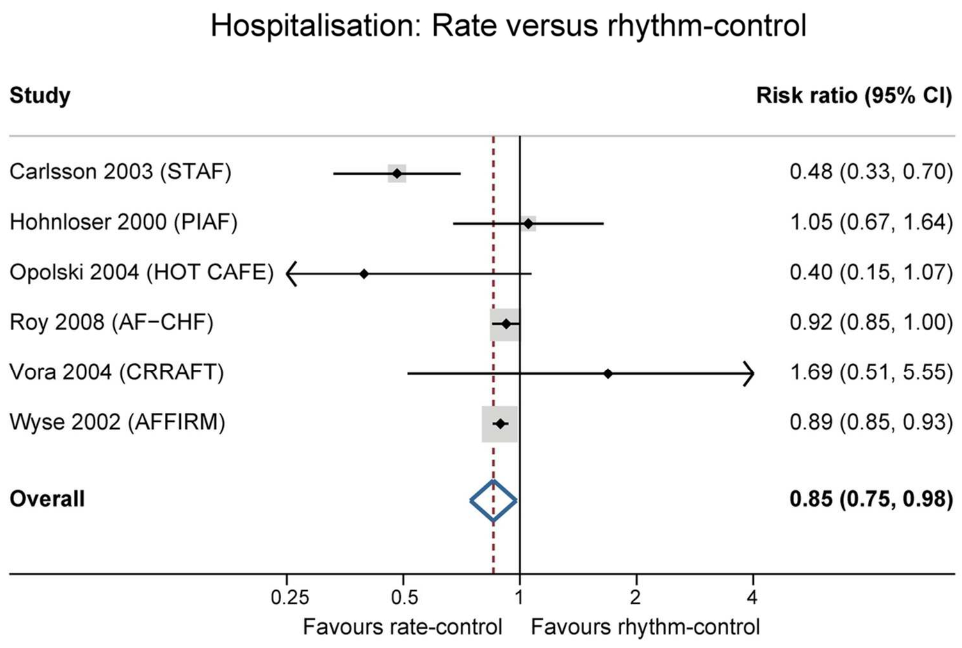

106x71mm (300 x 300 DPI)

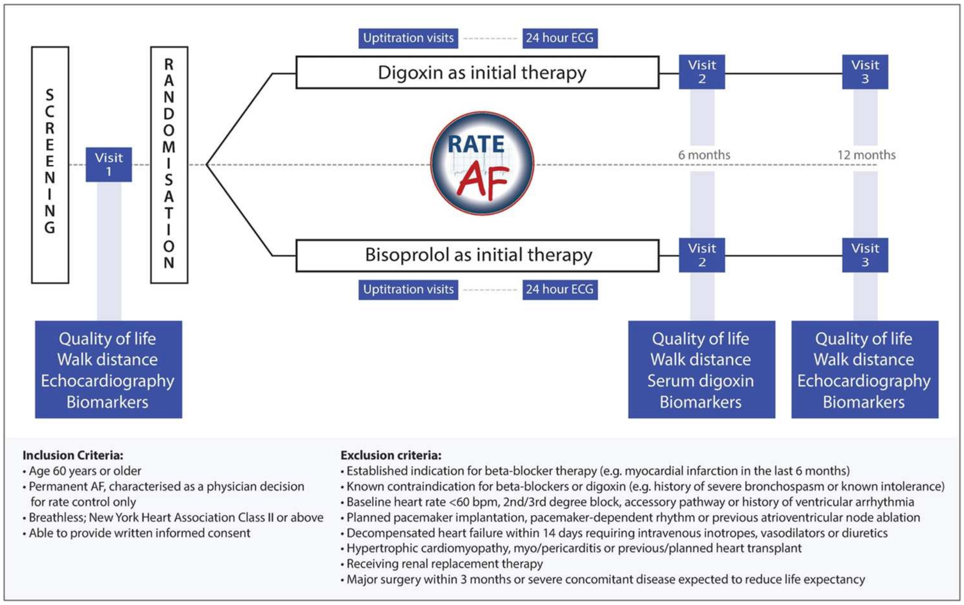

125x81mm (300 x 300 DPI)

For peer review only

Evaluating different rate control therapies in permanent atrial fibrillation: A prospective, randomised, open-label, blinded endpoint trial comparing digoxin and beta-blockers as initial rate control therapy

RAte control Therapy Evaluation in Atrial Fibrillation:

RATE-AF

RATE-AF TRIAL PROTOCOL

Version 1.0, 23rd March 2016

**Sponsor:**	**University of Birmingham**
**Chief Investigator:**	**Dr Dipak Kotecha**
**Coordinating Unit:**	**Birmingham Clinical Trials Unit**
**Funder:**	**National Institute for Health Research (NIHR) Career Development Fellowship**

ISRCTN: TBC
EudraCT No.: 2015-005043-13
REC Ref. No.: TBC

UNIVERSITY OF BIRMINGHAM

BIRMINGHAM CLINICAL TRIALS UNIT

NHS National Institute for Health Research

For peer review only

TRIAL COMMITTEES AND CONTACT DETAILS

Trial Management Group	
**Chief Investigator**<br>**Dr Dipak Kotecha**	**NIHR Career Development Fellow & Clinician Scientist**<br><br>Institute of Cardiovascular Sciences, University of Birmingham, The Medical School, Vincent Drive, Birmingham, B15 2TT, UK<br>Email: d.kotecha@bham.ac.uk<br>Telephone: 07974 115676
**Prof Paulus Kirchhof**	**Professor of Cardiovascular Medicine**<br><br>Institute of Cardiovascular Sciences, University of Birmingham, Institute of Biomedical Research, Vincent Drive, Birmingham B15 2TT, UK<br>Email: p.kirchhof@bham.ac.uk<br>Telephone: 0121 414 7042
**Dr Michael Griffith**	**Consultant Electrophysiologist**<br><br>University Hospitals Birmingham NHS Trust, Nuffield House, Queen Elizabeth Hospital, Birmingham, B15 2TH, UK<br>Email: michael.griffith@uhb.nhs.uk<br>Telephone: 0121 371 4038
**Prof Gregory Y H Lip**	**Professor of Cardiovascular Medicine & Director, Haemostasis Thrombosis & Vascular Biology Unit**<br><br>Institute of Cardiovascular Sciences, University of Birmingham, City Hospital, Birmingham, B18 7QH, UK<br>Email g.y.h.lip@bham.ac.uk<br>Telephone: 0121 5075080
**Prof Jonathan Townend**	**Professor of Cardiology**<br><br>University Hospitals Birmingham NHS Trust, Nuffield House, Queen Elizabeth Hospital, Birmingham, B15 2TH, UK<br>Email: john.townend@uhb.nhs.uk<br>Telephone: 0121 371 4623
**Dr Rick Steeds**	**Consultant Cardiologist and Head of Cardiac Imaging**<br><br>University Hospitals Birmingham NHS Trust, Nuffield House, Queen Elizabeth Hospital, Birmingham, B15 2TH, UK<br>Email: rick.steeds@uhb.nhs.uk<br>Telephone: 0121 371 6130
**Prof Melanie Calvert**	**Professor of Outcomes Methodology**<br><br>Institute of Applied Health Research, University of Birmingham, Birmingham, B15 2TT, UK<br>Email: m.calvert@bham.ac.uk<br>Telephone: 0121 414 8595
**Dr Susan Jowett**	**Senior Lecturer, Health Economics**<br><br>Institute of Applied Health Research, University of Birmingham, Birmingham, B15 2TT, UK<br>Email s.jowett@bham.ac.uk<br>Telephone: 0121 414 7898
**Dr Jonathan Mathers**	**Senior Lecturer, Qualitative and Mixed Methods Applied Health Research**<br><br>Institute of Applied Health Research, University of Birmingham, Birmingham, B15 2TT, UK<br>Email j.m.mathers@bham.ac.uk<br>Telephone: 0121 414 6024

For peer review only

Birmingham Clinical Trials Unit

**Prof Jon Deeks**	**Professor of Biostatistics and Director, BCTU**<br><br>Birmingham Clinical Trials Unit, University of Birmingham, Birmingham, B15 2TT, UK<br>Email j.deeks@bham.ac.uk<br>Telephone: 0121 414 5328
**Dr Margaret Grant**	**Operations Manager**<br><br>Birmingham Clinical Trials Unit, University of Birmingham, Birmingham, B15 2TT, UK<br>Email m.r.grant@bham.ac.uk<br>Telephone: 0121 415 9106
**Gemma Slinn**	**Senior Trial Coordinator**<br><br>Birmingham Clinical Trials Unit, University of Birmingham, Birmingham, B15 2TT, UK<br>Email g.slinn@bham.ac.uk<br>Telephone: 0121 415 8445
**Samir Mehta**	**Statistician**<br><br>Birmingham Clinical Trials Unit, University of Birmingham, Birmingham, B15 2TT, UK<br>Email s.mehta.1@bham.ac.uk<br>Telephone: 0121 415 9117

Trial Oversight Committee

Co-Chairs

**Dr Kazem Rahimi**	**Associate Professor of Cardiovascular Medicine, University of Oxford**<br>**Deputy Director, The George Institute for Global Health**<br><br>The George Institute for Global Health, University of Oxford, 34 Broad Street, Oxford OX1 3BD, UK<br>Email: kazem.rahimi@georgeinstitute.ox.ac.uk<br>Telephone: 01865 617 201
**Prof. John Camm**	**BHF Professor of Clinical Cardiology**<br><br>St George's University of London, Cranmer Terrace, London SW17 0RE, UK<br>Email: jcamm@sgul.ac.uk<br>Telephone: 0208 725 3414

Patient Representative

**Mary Stanbury**	Lead PPI Representative<br>Email: dms27@btinternet.com

On behalf of the Trial Management Group

**Dr Dipak Kotecha**	
**Prof. Jon Deeks**	For contact details, see Trial Management Group
**Prof. Paulus Kirchhof**	

For peer review only

For peer review only - http://bmjopen.bmj.com/site/about/guidelines.xhtml

RATE-AF Trial Office

For general protocol related queries and supply of trial materials:

Birmingham Clinical Trials Unit (BCTU), Institute of Applied Health Research, College of Medical & Dental Sciences, Public Health Building, University of Birmingham, Edgbaston, Birmingham B15 2TT

Telephone: 0121 415 8445
Fax: 0121 415 9135
Email: RATE-AF@trials.bham.ac.uk
Website: www.birmingham.ac.uk/RATE-AF

Randomisation

Telephone: 0800 953 0274

Website: https://www.birmingham.ac.uk/RATEAF

Safety Reporting

Fax SAE Forms to: 0121 415 9135 or 0121 415 9136

Protocol Development and Sign Off

Protocol Amendments

The following amendments and/ or administrative changes have been made to this protocol since the implementation of the first approved version

Amendment number	Date of amendment	Protocol version number	Type of amendment	Summary of amendment

For peer review only - http://bmjopen.bmj.com/site/about/guidelines.xhtml

Chief Investigator Signature Page

Trial Name: **RATE-AF**

Protocol Version Number: Version: __ __

Protocol Version Date: __ __ / __ __ __ / __ __ __ __

This protocol has been approved by:

CI Name: Dr Dipak Kotecha

Trial Role: Chief Investigator

Signature and date: ____________________ __ __ / __ __ __ / __ __ __ __

Sponsor Statement

Where the University of Birmingham takes on the sponsor role for protocol development oversight, the signing of the IRAS form by the Sponsor will serve as confirmation of the approval of this protocol.

For peer review only

For peer review only - http://bmjopen.bmj.com/site/about/guidelines.xhtml

Principal Investigator Signature Page

Principal Investigator:

I have read and agree to the protocol, as detailed in this document. I agree to adhere to the protocol as outlined and agree that any suggested changes to the protocol must be approved by the Trial Oversight Committee prior to seeking approval from the Research Ethics Committee and Regulatory Authority.

I am aware of my responsibilities as an Investigator under the guidelines of Good Clinical Practice (GCP), the Declaration of Helsinki, local regulations (as applicable) and the trial protocol and I agree to conduct the trial according to these guidelines and to appropriately direct and assist the staff under my control, who will be involved in the trial.

Trial Name: **RATE-AF**

Protocol Version Number: Version: __ __

Protocol Version Date: __ __ / __ __ __ / __ __ __ __

PI Name: <Enter>

Trial Role: Principal Investigator

Signature and date: ____________________________ __ __ / __ __ __ / __ __ __ __

The Principal Investigator should sign this page and return a copy to the RATE-AF Trial Office

For peer review only - http://bmjopen.bmj.com/site/about/guidelines.xhtml

Table of Contents

1 Trial Summary 13

1.1 Trial Schema 15

2 Introduction 16

2.1 Background 16

2.2 Epidemiology and Consequences of AF 16

2.3 Rhythm-Control in AF 17

2.4 Lack of Evidence to Guide Rate-Control Therapy 17

2.5 Patient Wellbeing 19

2.6 Rationale for the RATE-AF Trial 19

3 Trial Design and Objectives 20

3.1 Hypothesis 20

3.2 Primary objective 21

3.3 Secondary objectives 21

3.4 Feasibility objectives 21

3.5 Exploratory objectives 21

4 Selection of Participants 22

4.1 Inclusion Criteria 22

4.2 Exclusion Criteria 22

5 Informed Consent Process 23

6 Enrolment and Randomisation 24

6.1 Randomisation Procedures 25

7 Trial Treatment 26

7.1 Treatment 26

7.2 Treatment Supply and Storage 26

7.3 Dosing Schedule 27

7.4 Drug Interactions and Contraindications 27

7.5 Accountability Procedures and Labelling 29

7.6 Treatment Modification 29

7.7 Assessment of Compliance 30

8 Trial Procedures and Schedule of Assessments 30

8.1 Baseline Visit 30

For peer review only - http://bmjopen.bmj.com/site/about/guidelines.xhtml

8.2 Up-Titration Visits 31
8.3 Visit 2, Month 6..... 31
8.4 Visit 3, Month 12 (Final Trial Assessment)..... 32
8.5 Investigator-blinded Endpoints 32
8.6 Long Term Follow-Up..... 32
8.7 Withdrawal 33
8.8 Trial Duration..... 33
9 Trial Procedures..... 35
9.1 Procedures Defined as Standard Clinical Care..... 35
9.2 Medical History..... 35
9.3 Medication History..... 35
9.4 Physical Examination 36
9.5 Patient Reported Outcomes 36
9.5.1 Choice of Outcomes and Qualitative Research..... 36
9.5.2 Data Collection for PROMs..... 37
9.5.3 Outcome Appraisal..... 38
9.6 Transthoracic Echocardiography 38
9.6.1 Reproducibility and Validity of Measurements 38
9.6.2 Systolic LV Function..... 38
9.6.3 Diastolic LV Function..... 39
9.6.4 Left Atrial Size and Function..... 40
9.6.5 Additional Echocardiography Parameters..... 40
9.7 Laboratory Evaluations..... 40
9.7.1 Laboratory Assays..... 41
9.7.2 Cellular Response to Rate Control 41
9.7.3 Stored Blood Samples..... 41
9.7.4 Specimen Preparation, Handling, Storage and Shipment..... 41
9.8 Economic Evaluation..... 41
10 Pharmacovigilance 43
10.1 Recording and Assessment of Adverse Events 43
10.2 Non-Serious Adverse Events/ Adverse Reactions 45
10.3 Serious Adverse Events 45

For peer review only - http://bmjopen.bmj.com/site/about/guidelines.xhtml

10.3.1 Expected SAEs NOT to be Reported on a SAE Form 45
10.4 SUSARs 45
10.5 Development Safety Update Reports 46
10.6 Annual Progress Reports 46
10.7 Pregnancy 46
10.8 Reporting Urgent Safety Measures 46
11 Quality Control and Quality Assurance 47
11.1 Site Set-Up and Initiation 47
11.2 Central Monitoring 47
11.3 Audit and Inspection 47
11.4 Notification of Serious Breaches 48
11.5 Data Handling and Analysis 48
11.6 End of Trial 49
11.7 Archiving 49
12 Statistical Considerations 50
12.1 Outcome measures 50
12.1.1 Primary Outcome 50
12.1.2 Secondary Outcomes 50
12.1.3 Feasibility Outcomes 50
12.2 Power Calculations 51
12.3 Statistical analysis 51
12.3.1 Primary outcome analysis 52
12.3.2 Feasibility and Secondary outcomes analysis 52
12.3.3 Missing data and sensitivity analyses 52
12.3.4 Interim analyses and Stopping rules 52
12.4 Final analysis 53
13 Ethics and Regulatory Requirements 53
14 Oversight Committees 53
14.1 Trial Management Group 53
14.2 Trial Oversight Committee 54
14.3 Protocol amendments 54
15 Finance 54

For peer review only - http://bmjopen.bmj.com/site/about/guidelines.xhtml

16 Confidentiality and Data Protection ... 54

17 Insurance and Indemnity ... 55

18 Dissemination and Publication ... 55

19 Statement of Compliance ... 56

20 References ... 57

Appendix A: Randomised treatment arm - Digoxin

Appendix B: Randomised treatment arm - Bisoprolol

For peer review only

For peer review only - http://bmjopen.bmj.com/site/about/guidelines.xhtml

List of Abbreviations

**ABPI**	Association of the British Pharmaceutical Industry
**AE**	Adverse Event
**AF**	Atrial Fibrillation
**BCTU**	Birmingham Clinical Trials Unit
**BNP**	B-type Natriuretic Peptide
**BPM**	Beats per Minute
**CCB**	Calcium Channel Blocker
**CI**	Chief Investigator
**CMR**	Cardiac Magnetic Resonance
**CRF**	Case Report Form
**CTA**	Clinical Trial Authorisation
**CTIMP**	Clinical Trial of Medicinal Product
**DIBD**	Developmental International Birth Date
**DMC**	Data Monitoring Committee
**DSUR**	Developmental Safety Update Report
**DT**	Deceleration Time
**ECG**	Electrocardiogram
**EHRA**	European Heart Rhythm Association
**EU**	European Union
**EudraCT No.**	European Union Drug Regulating Authorities Clinical Trials Number
**GCP**	Good Clinical Practice
**GFR**	Glomerular Filtration Rate
**GP**	General Practitioner
**HF**	Heart Failure
**HR**	Hazard Ratio
**IMP**	Investigational Medicinal Product
**INR**	International Normalised Ratio
**ISRCTN**	International Standard Randomised Controlled Trial Number
**IVRT**	Isovolumic Relaxation Time
**LA**	Left-Atrial
**LV**	Left-Ventricular
**LVEDD**	Left-Ventricle End-Diastolic Dimension
**LVEDV**	Left-Ventricle End-Diastolic Volume
**LVEF**	Left-Ventricular Ejection Fraction

For peer review only

For peer review only - http://bmjopen.bmj.com/site/about/guidelines.xhtml

**LVESD**	Left-Ventricle End-Systolic Dimension
**LVESV**	Left-Ventricle End-Systolic Volume
**LVSD**	Left-Ventricular Systolic Dysfunction
**MHRA**	Medicines and Healthcare Products Regulatory Agency
**MREC**	Main Research Ethics Committee
**NHS**	National Health Service
**NICE**	National Institute of Clinical Excellence
**NOAC**	Novel Oral Anticoagulants
**NYHA**	New York Health Association
**PEF**	Preserved Ejection Fraction
**PI**	Principal Investigator
**PIC**	Patient Identification Centre
**PIL**	Participant Information Leaflet
**PROBE**	Prospective Randomised Open Blinded End-point
**QALY**	Quality-Adjusted Life Year
**QoL**	Quality of Life
**R&D**	Research and Development
**RCT**	Randomised Controlled Trial
**REC**	Research Ethics Committee
**SAE**	Serious Adverse Event
**SAR**	Serious Adverse Reaction
**SD**	Standard Deviation
**SmPC**	Summary of Product Characteristics
**SUSAR**	Suspected Unexpected Serious Adverse Reaction
**TAPSE**	Tricuspid Annular Plane Systolic Excursion
**TDI**	Tissue Doppler Imaging
**TMF**	Trial Master File
**TMG**	Trial Management Group
**TSC**	Trial Steering Committee
**UHB**	University Hospitals Birmingham
**WTCRF**	NIHR Wellcome Trust Clinical Research Facility at Queen Elizabeth Hospital, Birmingham

For peer review only

For peer review only - http://bmjopen.bmj.com/site/about/guidelines.xhtml

1 Trial Summary

**Title**	**Evaluating different rate control therapies in permanent atrial fibrillation: A prospective, randomised, open-label, blinded endpoint trial comparing digoxin and beta-blockers as initial rate control therapy**<br><br>**RAte control Therapy Evaluation in Atrial Fibrillation: RATE-AF**
**Acronym**	**RATE-AF**
**Trial Design and Methods**	A prospective, randomised, open-label, blinded-endpoint (PROBE) trial design. The RATE-AF trial combines hypothesis testing (quality of life, cardiac function, exercise capacity and biomarkers), evaluation of measures (validity, reproducibility and correlation of outcomes) and a feasibility study for a future clinical event trial (assessing recruitment, retention and sample size).
**Trial Medications**	Digoxin 62.5 – 250 µg od<br>Bisoprolol 1.25 – 15 mg od
**Trial Outcomes**	**Primary Outcome:**
	Patient-reported quality of life (QoL): SF-36 physical component summary score at six months
	**Secondary Outcomes:**
	Patient-reported QoL:<br>• SF-36 global and domain-specific scores at 6 and 12 months<br>• EQ-5D-5L summary index and visual analogue scale at six and twelve months<br>• AFEQT overall score at six and twelve months
	Cardiac function:<br>• Echocardiographic LVEF at 12 months<br>• Diastolic function (E/e' and composite of diastolic indices) at 12 months
	Functional assessment:<br>• Six-minute walking distance at 6 and 12 months<br>• Change in European Heart Rhythm Association (EHRA) class at 6 and 12 months
	Biomarkers:<br>• Change in B-type natriuretic peptide (BNP) levels at 6 months
	Change in heart rate using 24-hour ambulatory ECG
	**Feasibility Outcomes:**
	Recruitment target of 3 patients per week across all participating centres.
	Compliance and reasons for non-compliance
	Number of withdrawals and losses to follow-up (with reasons)
	Drug discontinuation rate and adverse reactions requiring drug discontinuation.
	Number of patients needing therapy-induced requirement for additional treatment

For peer review only - http://bmjopen.bmj.com/site/about/guidelines.xhtml

	Population-specific standard deviations (SD) and proportions:<br>• SD of SF36 physical functioning score at 6 and 12 months<br>• SD of SF36 overall score at 6 and 12 months<br>• SD of AFEQT overall score at 6 and 12 months<br>• SD of LVEF and E/e' scores at 6 and 12 months<br>• Unplanned hospitalisation admissions rates
	Cardiovascular Events (particularly mortality, thromboembolic events, myocardial infarction and cardiovascular interventions)
**Trial Duration per Participant**	12 months of trial therapy
**Planned Trial Sites**	Multiple screening sites with single site recruitment
**Total Number of Participants**	160
**Main Inclusion/ Exclusion Criteria**	**Inclusion Criteria**
	Adult patients, aged 60 years or older
	Permanent AF, characterised (at time of randomisation) as a physician decision for rate-control with no plans for cardioversion, anti-arrhythmic medication, or ablation therapy
	Symptoms of breathlessness (New York Heart Association Class II or more)
	Able to provide written, informed consent
	**Exclusion Criteria**
	Established indication for beta-blocker therapy, e.g. myocardial infarction in the last 6 months
	Known contraindications for therapy with beta-blockers or digoxin, e.g. a history of severe bronchospasm that would preclude use of beta-blockers, or known intolerance to these medications
	Baseline heart rate <60 bpm
	History of second or third-degree heart block
	Supraventricular arrhythmias associated with accessory conducting pathways (e.g. Wolff-Parkinson-White syndrome) or a history of ventricular tachycardia or fibrillation
	Planned pacemaker implantation (including cardiac resynchronisation therapy), pacemaker-dependent rhythm or history of atrioventricular node ablation
	Decompensated heart failure (evidenced by need for intravenous inotropes, vasodilators or diuretics) within 14 days prior to randomisation
	A current diagnosis of obstructive hypertrophic cardiomyopathy, myocarditis or constrictive pericarditis
	Received or on waiting list for heart transplantation
	Receiving renal replacement therapy
	Major surgery, including thoracic or cardiac surgery, within 3 months of randomisation
	Severe, concomitant non-cardiovascular disease (including malignancy) that is expected to reduce life expectancy

For peer review only - http://bmjopen.bmj.com/site/about/guidelines.xhtml

1.1 Trial Schema

Figure 1

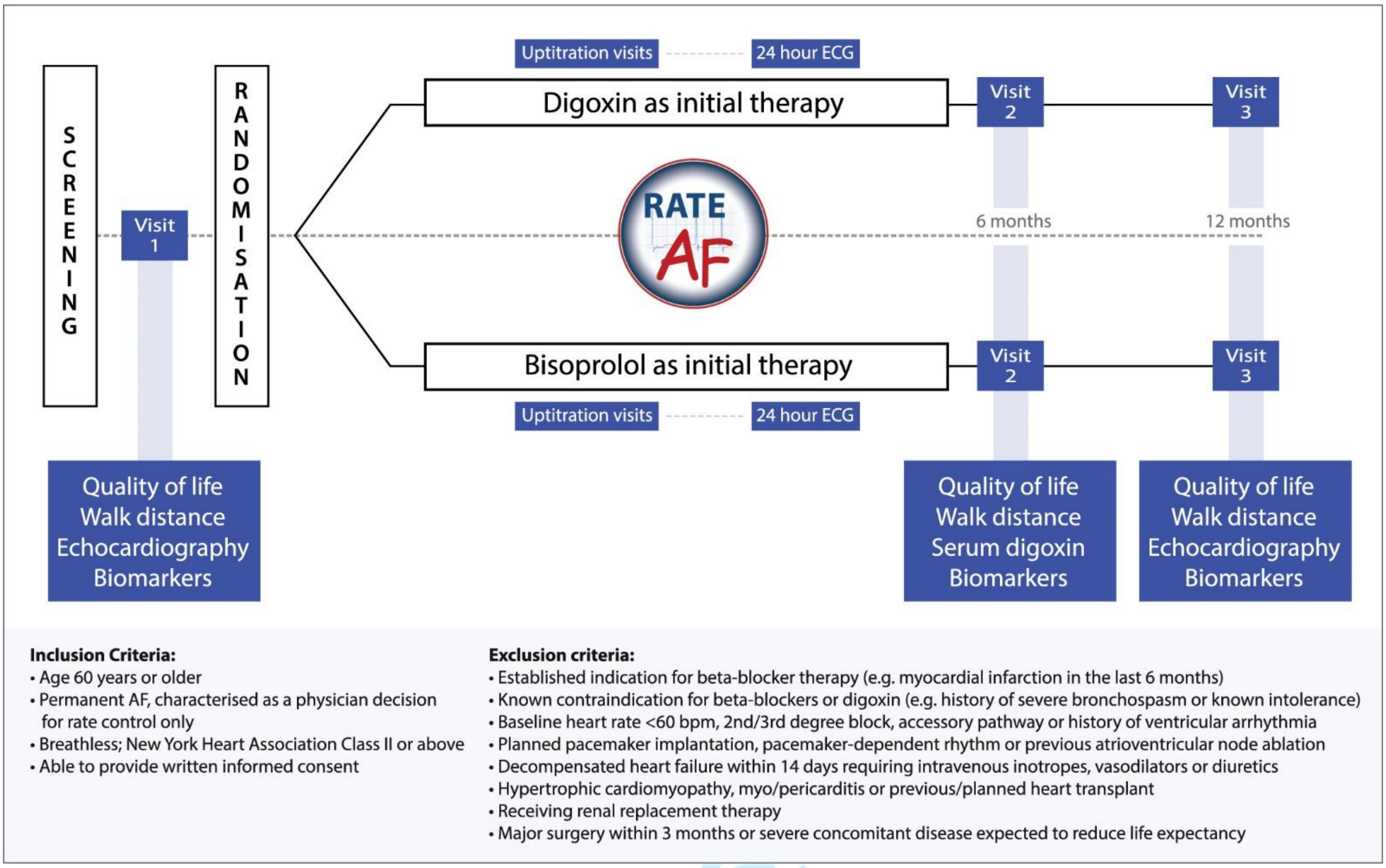

This protocol describes the **RATE-AF** trial only. The trial will be conducted in accordance with the protocol, Good Clinical Practice (GCP) and applicable regulatory requirements. Every care has been taken in the drafting of this protocol, but future amendments may be necessary, which will receive the required approvals prior to implementation.

For peer review only - http://bmjopen.bmj.com/site/about/guidelines.xhtml

2 Introduction

2.1 Background

Atrial fibrillation (AF) is an increasingly common cardiac condition that leads to a substantial burden on quality-of-life (QoL), an increased risk of cardiovascular events, hospitalisation and death, and significant healthcare costs for the NHS. In addition to anticoagulation and considerations for rhythm control therapy, most patients with AF are in need of pharmacological control of heart rate. This aspect of care has not received stringent investigation, with treatment guidelines based on small crossover studies and observational data rather than robust controlled trials.[1-3] Beta-blocker monotherapy remains the first-line option in the current NICE AF guidelines consultation document, with digoxin only for sedentary patients, although this recommendation is based on 'very low-quality evidence'.[4] The benefit of different rate-control therapies on symptoms and other intermediate outcomes (such as left-ventricular ejection fraction [LVEF] and diastolic function) are unknown, as are their effects on clinical events such as hospitalisation. This situation is unacceptable in light of the potential benefits and risk of different rate-control options in AF. It also limits our ability to personalise treatment according to patient characteristics.

The RAte control Therapy Evaluation in Atrial Fibrillation (**RATE-AF**) trial is informed by a number of in-depth systematic reviews of management and clinical outcomes in AF patients.[5-11] Taken together, this information provides a sound basis to plan a major randomised controlled trial (RCT).[12, 13] However as trials of rate-control in AF have typically been small or uncontrolled, further information is needed before designing a trial that can assess clinical outcomes. The **RATE-AF** trial will allow us to define appropriate primary and secondary outcome measures and their standard deviation in a contemporary population of patients with permanent AF. This information will allow us to estimate sample size, determination of recruitment, retention and adherence policies, and to ascertain the best methods of obtaining adverse event data and reliable economic costs for a larger trial assessing cardiovascular outcomes and hospitalisation. The **RATE-AF** trial will also be the largest RCT of its kind, allowing us to compare the effect of beta-blockers and digoxin on QoL as initial rate-control therapy in patients with permanent AF. The long-term aim of the research is to answer key questions about how to initiate therapy, stratified by relevant patient characteristics such as systolic and diastolic cardiac function, baseline symptoms and concurrent medication. The research will also define the patho-physiological mechanisms underlying AF-related symptoms, left-ventricular function and their association with adverse clinical outcomes, and to identify clinical markers for the response to different rate control therapy.

2.2 Epidemiology and Consequences of AF

AF is a common condition that is associated with increased rates of mortality and serious morbidity, including stroke, worsening of heart failure, sudden death, and reduced QoL.[1] The prevalence of AF increases with age, ranging from 0.7% in those aged 55–59 years to 17.8% in those aged above 85.[14] A doubling of both incidence and prevalence of AF is predicted in the next 20 years.[15]

For peer review only - http://bmjopen.bmj.com/site/about/guidelines.xhtml

Patients with AF are twice as likely to be hospitalised as propensity score-matched controls, with direct medical costs estimated to be 73% higher.[16] Further, AF is an independent predictor of all-cause mortality, with a two-fold adjusted increase in death.[17, 18] While most strokes in AF can be prevented by oral anticoagulation, AF patients still have high cardiovascular death rates due to sudden death or progressive heart failure.[19, 20] Patients with AF also have significantly poorer QoL[21], experiencing a variety of symptoms including lethargy, palpitations, dyspnoea, sleeping difficulties and psychosocial distress.[22, 23] In the context of patients diagnosed with heart failure, the presence of AF leads to higher rates of death and hospitalisation, independent of other risk variables or which condition comes first.[24, 25] From observational data, 40% of AF patients will be diagnosed with heart failure and vice-versa[16], representing a large and growing unmet clinical need for healthcare improvement.

2.3 Rhythm-Control in AF

Numerous large RCTs comparing rhythm-control (using arrhythmic drugs and/or cardioversion) versus rate-control have identified no significant difference in clinical outcomes in patients with persistent AF.[26-30] In a number of studies, hospitalisation rates were actually higher in those randomised to rhythm-control.[26, 29, 30] Similar findings have been shown in AF patients with heart failure[31, 32], both in those with impaired and preserved ejection fraction.[33-35] Although AF ablation is becoming increasingly popular to restore sinus rhythm, it remains a highly invasive method to improve AF-related symptoms.[36, 37] At present, European and NICE treatment guidelines recommend ablation only in symptomatic paroxysmal AF, or as a treatment option in symptomatic persistent AF that is refractory to other therapy.[3] Further trials are currently underway to determine the clinical value of prompt rhythm-control, including the Early treatment of Atrial fibrillation for Stroke prevention Trial (EAST).[38] In light of the high recurrence rate of AF (even in patients receiving intensive rhythm-control therapy), rate-control is an important part of AF management in almost all patients. Unfortunately, rate-control therapy has much less evidence underpinning its use.

2.4 Lack of Evidence to Guide Rate-Control Therapy

Rate-control in AF can be achieved with beta-blockers, non-dihydropyridine calcium-channel blockers (CCB), digoxin and their combinations. Unfortunately, little data exists to assist clinicians in choosing appropriate first-line and subsequent therapy. Current patterns of medication usage vary considerably (between and within countries). For example, in a worldwide registry, digoxin was prescribed in 2877 of 10,523 patients (27.3%), compared to 1599 of 3141 (50.9%) of patients in the German Competence NETwork on Atrial Fibrillation (AFNET).[39, 40]

Current European guidelines suggest “the choice of medication should be individualised and the dose modulated to avoid bradycardia”. This recommendation (Class 1, Level B) is based on a systematic review of trials addressing rate-control between 1983 and 1997.[41] Most of the studies included less than 50 participants (with several less than 10). The majority were low quality studies, as assessed by the risk of bias or confounding, and follow-up was typically in the order of

For peer review only - http://bmjopen.bmj.com/site/about/guidelines.xhtml

hours, days or weeks. Whilst this may be sufficient to assess an acute effect on heart-rate, it provides limited data on the longer-term effects of different treatments or the frequency of adverse reactions.

Beta-blockers are often preferred over other agents due to the prognostic benefit seen in patients with heart failure who are in sinus rhythm. However, in patients with heart failure, reduced LVEF and concomitant AF, we have shown that beta-blockers do not reduce mortality (hazard ratio 0·97, 95% CI 0.83-1.14; p=0.73) or cardiovascular hospital admissions (hazard ratio 0·91; 95% CI 0.79-1.04; p=0.15).[5] This distinctly contrasts with the significant benefit seen in patients with sinus rhythm and highlights the need for further comparative RCTs specifically in patients with AF.

The most highly cited trial comparing beta-blockers and digoxin for rate-control in chronic AF was an open-label two-week crossover study of 5 drug regimes in 12 patients.[42] Peak heart-rate after exercise was significantly higher in those taking digoxin compared to beta-blockers but there were no differences in exercise duration. In a trial of 42 patients, rate-control was improved with combination beta-blocker/digoxin therapy compared to digoxin alone, however there were similarly no differences in exercise capacity.[43] Systematic review of other small randomised studies identify no difference in exercise tolerance with beta-blockers, despite a lowering of heart-rate.[44] From observational data, such as the Atrial Fibrillation Follow-up Investigation of Rhythm Management (AFFIRM) study, more cardiac and non-cardiac adverse effects have been noted with beta-blockers than digoxin (n=67 vs. n=38).[28] In a 3-week crossover study of 60 participants, 10% withdrew during beta-blocker therapy due to adverse events.[45] Those in the beta-blocker group had a reduction in exercise capacity on cardio-pulmonary testing and a significant increase in B-type natriuretic peptide (BNP, a marker of ventricular strain) compared to patients treated with calcium-channel blockers.[46]

Only a single RCT has been published comparing digoxin and beta-blockers in patients with AF and heart failure (mean LVEF 24%, n=47).[47] Although there was a marginally-significant improvement in LVEF with carvedilol/digoxin versus placebo/digoxin, blinded withdrawal of digoxin then led to a deterioration in LVEF, accompanied by an increase in BNP. There was no difference in the number of heart-rate pauses >3 seconds or in daytime/exercise heart-rate comparing the two therapies alone.

Digoxin itself has been associated with an increased mortality in observational cohorts of AF patients[48], however careful adjustment of baseline differences reject a true excess in adverse outcomes.[49-51] In a detailed systematic review of all studies published on digoxin, we identified that confounding was the main reason that digoxin was associated with increased mortality in observational studies, and confirmed a neutral association in RCTs (risk ratio 0.99, 95% CI 0.93 to 1.05).[6] Lower rates of hospitalisation have been noted with digoxin therapy, independent of the type of heart failure[52], however the lack of randomised data versus placebo (despite widespread clinical use) makes true comparison difficult. Small RCTs comparing CCB with digoxin have been inconsistent; two have identified lower heart-rates with CCB but no significant difference in exercise capacity[42, 43], one demonstrated higher post-exercise cardiac output after

For peer review only - http://bmjopen.bmj.com/site/about/guidelines.xhtml

digoxin[53] and another showed improved exercise duration and QoL with CCB.[54] These results highlight the need for randomised data with appropriately-defined outcomes to accurately identify the benefits and risks of common therapies in patients with AF.

An example where RCT data have impacted on clinical practice is the Rate Control Efficacy in Permanent Atrial Fibrillation (RACE II) trial. This study challenged conventional wisdom that stricter control of heart-rate would allow time for diastolic filling and improve haemodynamics. In summary, 614 patients with permanent AF were randomised to strict or lenient rate-control and followed for 2-3 years.[55] There was no significant difference in the cumulative incidence of the composite primary outcome; 14.9% in the strict-control arm and 12.9% in the lenient-control group. There were also no differences in symptoms, New York Heart Association (NYHA) class or hospitalisations[55, 56], no interaction with baseline heart failure[57], and those participants achieving strict rate-control required more clinic visits and higher doses of medical therapy.[58] Current guidelines therefore suggest that lenient rate-control is acceptable, except for patients with adverse symptoms or clinical deterioration.[1] Whilst this study provides important data on the intensity of rate-control in AF, the more clinically-relevant questions of how to initiate therapy and the choice of optimal agents for individual patients remain unanswered.

2.5 Patient Wellbeing

Patient-reported outcomes are any report of a patient's health status (for example QoL) that is derived directly from the patient, without interpretation by a clinician.[59] There is limited data on the effect of pharmacological rate-control therapy on QoL and no comparative data assessing the benefit of different strategies.[22, 60] Rate-control has been associated with improved QoL scores in trials assessing rate versus rhythm-control.[61, 62] In the PIAF study, over 50% of participants randomised to calcium-channel blockers reported an improvement in health with significant benefits in the physical aspects of the SF-36.[63] A number of smaller studies have shown inconsistent effects on QoL in AF, although the data is limited by inclusion of patients with paroxysmal AF, a focus on heart rate and the use of a variety of QoL tools.

Current QoL questionnaires can be divided into disease-specific evaluations or generic health assessments (such as the Short Form Health Survey SF-36[64] or the EuroQol EQ-5D[65, 66]). However there is a distinct lack of knowledge regarding the mechanisms that underpin AF-related symptoms, the responsiveness of QoL questionnaires and their validity.[60] The Atrial Fibrillation Effect on QualiTy-of-life (AFEQT) questionnaire was designed to address these disparities by using more robust methods.[67] Although there is limited clinical application to-date, AFEQT has demonstrated sensitivity to clinical change.[68] An important objective of the research is to ascertain appropriate and responsive QoL tools for this population, as well as determine the acceptability and delivery of the questionnaires to patients.

2.6 Rationale for the RATE-AF Trial

Rate-control is an integral part of management in all AF patients but hardly any controlled trial evidence exists to guide the choice of agents. We have shown that neither beta-blockers nor

For peer review only - http://bmjopen.bmj.com/site/about/guidelines.xhtml

digoxin has an impact on mortality in AF patients, even with concomitant heart failure, which highlights the need to determine treatment effects on quality of life and cardiac function.

3 Trial Design and Objectives

RATE-AF is Prospective, Randomised Open-label Blinded Endpoint (PROBE) clinical trial comparing the use of digoxin and beta-blockers as initial rate control therapy.

In this section, we discuss the trial design and study objectives. Detailed outcome measures are listed in **Section 12**.

Justification for a PROBE rather than a Double Blind Trial Design

Although a double blind design would be the most robust trial design with respect to bias, it would not be ethical to do so in this scenario as clinicians would feel the need to add therapy according to heart rate. In addition, the RATE-AF Trial aims to test a strategy of initial care. PROBE trial design maintains the benefits associated with a strict randomisation procedure, while the blinded end points help to eliminate bias.

The trial design aims for a pragmatic ‘all-comers’ approach, applicable to those seen in clinical practice to allow transfer of the findings to routine clinical management of patients with permanent AF.

Assessment and Management of Risk

This trial is categorised by the Medicines and Healthcare products Regulatory Agency (MHRA) as:

Type A = No higher than the risk of standard medical care

The assessment and management of risk is detailed in the separate **RATE-AF** Risk Assessment document. An on-going evaluation of risk will continue throughout the recruitment period.

3.1 Hypothesis

Null Hypothesis for primary outcome:

No difference in patient-reported quality of life (measured using the physical functioning domain of the SF36 questionnaire) when comparing a strategy of digoxin versus beta-blocker therapy for initial rate control in patients with permanent AF.

Alternative Hypothesis:

Use of digoxin or beta-blocker therapy as initial rate control in patients with permanent AF is superior based on patient reported quality of life (measured using the physical functioning domain of the SF36 questionnaire).

For peer review only - http://bmjopen.bmj.com/site/about/guidelines.xhtml

3.2 Primary objective

- Patient-reported quality of life (QoL), with a predefined focus on physical well-being using the SF-36 physical component summary at six months.

3.3 Secondary objectives

- Generic and AF-specific patient-reported QoL using the SF-36 global and domain-specific scores, the AFEQT overall score and the EQ-5D-5L summary index and visual analogue scale at six and twelve months.
- Echocardiographic left-ventricular ejection fraction (LVEF) and diastolic function (E/e' and composite of diastolic indices) at twelve months.
- Functional assessment, including 6-minute walking distance achieved, change in European Heart Rhythm Association (EHRA) class and cognitive function at six and twelve months.
- Change in B-type natriuretic peptide (BNP) levels as a surrogate for total cardiac strain at six months.
- Change in heart rate from baseline and group comparison using 24-hour ambulatory ECG.

3.4 Feasibility objectives

- Successful methods for recruitment
- Key issues that affect retention of participants, such as convenience, compliance and cross-over (target of 85% study completion rate).
- Drug discontinuation rate and adverse reactions leading to drug discontinuation.
- Therapy-induced requirement for additional treatment (e.g. pacemaker implantation).
- Population-specific standard deviations and proportions to enable sample size calculation for a future trial.
- Assessment of cardiovascular outcomes including a composite of adverse clinical events (mortality, thromboembolic events, myocardial infarction and cardiovascular interventions).

3.5 Exploratory objectives

- Correlation of baseline measures, including QoL questionnaires and unblinded baseline investigations such as QoL, BNP, LVEF, E/e', EHRA, intracellular methods and heart rate.
- Impact of therapy on intracellular sodium and calcium concentration and cardiotonic steroid levels as biomarkers of cellular response at six and twelve months.

For peer review only - http://bmjopen.bmj.com/site/about/guidelines.xhtml

- Impact of combination therapy on outcomes, including comparison of bisoprolol/non-dihydropyridine calcium channel blocker (CCB) vs. bisoprolol/digoxin vs. digoxin/CCB vs. single therapies.
- Change in cognitive function at twelve months
- Qualitative research of patient-reported QoL using focus groups to explore patient acceptability, optimal delivery methods and responsiveness.
- Assessment of the validity and reproducibility of echocardiographic measures in patients with AF.
- Correlation of serum digoxin concentration with change in QoL and intracellular methods.
- Cost-consequence economic analysis from an NHS perspective.

4 Selection of Participants

Participants who potentially fulfil the inclusion criteria for this trial must have their eligibility confirmed by medically qualified personnel with access to and a full understanding of the potential participant's medical history. If eligibility has been assessed and documented by medically qualified personnel, then the process of obtaining informed consent may be delegated as appropriate and as documented on the **RATE-AF** Delegation and Signature Log.

4.1 Inclusion Criteria

- Adult patients aged 60 years or older
- Permanent AF, characterised (at time of randomisation) as a physician decision for rate-control with no plans for cardioversion, anti-arrhythmic medication, or ablation therapy
- Symptoms of breathlessness (New York Heart Association Class II or more)
- Able to provide written informed consent

4.2 Exclusion Criteria

- Established clinical indication for beta-blocker therapy, e.g. myocardial infarction in the last 6 months
- Known contraindications for therapy with beta-blockers or digoxin, e.g. a history of severe bronchospasm that would preclude use of beta-blockers, or known intolerance to these medications
- Baseline heart rate <60 bpm
- History of second or third-degree heart block
- Supraventricular arrhythmias associated with accessory conducting pathways (e.g. Wolff-Parkinson-White syndrome) or a history of ventricular tachycardia or fibrillation

For peer review only - http://bmjopen.bmj.com/site/about/guidelines.xhtml

- Planned pacemaker implantation (including cardiac resynchronisation therapy), pacemaker-dependent rhythm or history of atrioventricular node ablation
- Decompensated heart failure (evidenced by need for intravenous inotropes, vasodilators or diuretics) within 14 days prior to randomisation
- A current diagnosis of obstructive hypertrophic cardiomyopathy, myocarditis or constrictive pericarditis
- Received or on waiting list for heart transplantation
- Receiving renal replacement therapy
- Major surgery, including thoracic or cardiac surgery, within 3 months of randomisation
- Severe, concomitant non-cardiovascular disease (including malignancy) that is expected to reduce life expectancy

5 Informed Consent Process

It will be the responsibility of the Investigator to obtain written informed consent for each participant prior to performing any trial related procedure. If local practice allows, this responsibility may be delegated by the Principal Investigator, to a Research Nurse as captured on the Site Signature and Delegation Log. A Participant Information Leaflet (PIL) will be provided to facilitate this process. Investigators or delegate(s) will ensure that they adequately explain the aim, trial treatment, anticipated benefits and potential hazards of taking part in the trial to the participant. They will also stress that participation is voluntary and that the participant is free to refuse to take part and may withdraw from the trial at any time. The participant will be given adequate time to read the PIL and to discuss their participation with others outside of the site research team. The participant will be given the opportunity to ask questions.

If the participant expresses an interest in participating in the trial they will be asked to sign and date the latest version of the Informed Consent Form (ICF). The participant must give explicit consent for the regulatory authorities, members of the research team and representatives of the sponsor to be given direct access to the participant's medical records.

The Investigator or delegate(s) will then sign and date the form. A copy of the ICF will be given to the participant, a copy will be filed in the medical notes, and the original placed in the Investigator Site File (ISF). Once the participant is entered into the trial, the participant's unique trial identification number will be entered on the ICF maintained in the ISF. As part of the consent process, the participant will be asked to give explicit consent to their trial-related information being sent to the Trials Office at the University of Birmingham.

This trial will include **optional consent** to allow linkage to patient data available in NHS routine clinical datasets, including primary care data (e.g. Clinical Practice Research Datalink; CPRD, The Health Improvement Network; THIN, QResearch), secondary care data (Hospital Episode Statistics; HES) and mortality data from the Office of National Statistics (ONS) through The Health and Social Care Information Centre and other central UK NHS bodies. The consent will

For peer review only - http://bmjopen.bmj.com/site/about/guidelines.xhtml

also allow access to other new central UK NHS databases that will appear in the future. This will allow us to double check the main outcomes against routine data sources, and extend the follow-up of patients in the trial and collect long-term outcome and health resource usage data without needing further contact with the trial participants. This is important as it will link a trial of treatments that may become a clinical standard of care to long-term outcomes that are routinely collected in clinical data but which may be collected during the follow-up period of the trial.

Details of the informed consent discussions will be recorded in the participant's medical notes. This will include date of discussion, the name of the trial, summary of discussion, version number of the PIL given to participant and version number of ICF signed and date consent received. Where consent is obtained on the same day that the trial related assessments are due to start, a note will be made in the medical notes as to what time the consent was obtained and what time the procedures started.

At each visit the participant's willingness to continue in the trial will be ascertained and documented in the medical notes. Throughout the trial the participant will have the opportunity to ask questions about the trial. Any new information that may be relevant to the participant's continued participation will be provided. Where new information becomes available which may affect the participants' decision to continue, participants will be given time to consider and if happy to continue will be re-consented. Re-consent will be documented in the medical notes. The participant's right to withdraw from the trial will remain.

Electronic copies of the PIL and ICF will be available from the Trials Office and will be presented on the headed paper of the local institution. Details of all participants approached about the trial will be recorded on the Participant Screening/Enrolment Log and with the participant's prior consent, their General Practitioner (GP) will also be informed that they are taking part in the trial.

6 Enrolment and Randomisation

A flowchart of the recruitment process is shown in the Trial Schema (**Figure 1**) together with the schedule of investigation. **Section 9** gives more detailed information of trial procedures and assessments.

In the majority, potentially eligible participants will be identified by their Cardiologist, usually following referral from their General Practitioner (GP), and provided with an ethically-approved patient information leaflet (PIL). The patient will then be invited to attend a baseline visit at the NIHR Wellcome Trust Clinical Research Facility (WTCRF) at Queen Elizabeth Hospital, Birmingham. Potentially eligible participants may also be identified from inpatient referrals; these patients will be provided with a PIL and invited to attend a baseline visit following the same procedure.

GP Practices in the Birmingham area may be asked to refer patients that present with AF, but are not on medication, to the RATE-AF Research Team at University Hospitals Birmingham (UHB). These patients will be given a one-page, ethics committee-approved trial summary and asked to

For peer review only - http://bmjopen.bmj.com/site/about/guidelines.xhtml

sign a contact details form to confirm that they are happy to be contacted by a member of the Research Team to arrange an appointment.

Prior to patients undertaking any trial-related procedures, informed consent will be obtained.

Details of all patients approached about the trial should be recorded on the **RATE-AF** Screening & Enrolment Log. This Log should be maintained within the Investigator Site File.

6.1 Randomisation Procedures

After all eligibility criteria have been confirmed and informed consent has been received, the participants can be randomised into the **RATE-AF** trial.

Participants will be randomised in a 1:1 ratio to either **Digoxin 62.5 – 250 µg od or Bisoprolol 1.25 – 15 mg od**. The time between randomisation and commencement of trial therapy should be minimised (ideally <24 hours). Randomisation will be provided by a computer generated programme at the Birmingham Clinical Trials Unit (BCTU), using a minimisation algorithm to ensure balance between the arms with regard to important clinical variables, stratifying for baseline EHRA (class 1/2a and 2b/3/4) and gender.

Telephone and Online Randomisation

Participants can be randomised into the trial via a secure 24 hour internet based randomisation service (**https://www.trials.bham.ac.uk/RATEAF**) or by a telephone call to the BCTU (telephone number **0800 953 0274**). Telephone randomisations are available Monday-Friday, 09:00-17:00. For the secure internet randomisation, each site and each randomiser will be provided with a unique log-in username and password in order to access the online system. Online randomisation is available 24 hours a day, 7 days a week, apart from short periods of scheduled maintenance and occasional network problems.

Randomisation Forms will be provided to investigators and should be completed and used to collate the necessary information prior to randomisation. Once all eligibility criteria have been provided and confirmed, a Trial Number and treatment allocation be given and relevant parties notified, including the participant's GP.

Back-up Randomisation

If the internet based randomisation service is unavailable for an extended period of time, a back-up paper randomisation will also be available at the BCTU. The randomisation list will be produced using a random length block design. In this instance, investigators should ring the BCTU randomisation service (telephone number **0800 953 0274**).

For peer review only - http://bmjopen.bmj.com/site/about/guidelines.xhtml

7 Trial Treatment

7.1 Treatment

The Investigational Medicinal Products (IMPs) for this trial are Digoxin and Bisoprolol.

At randomisation, participants will be allocated to open-label treatment with either Digoxin 62.5 – 250 µg od or Bisoprolol 1.25 – 15 mg od.

Digoxin

Digoxin is a cardiac glycoside derived from the foxglove plant. The cardiac effects of digoxin therapy are summarised by:

- Positive inotropic effects: increased intracellular calcium due to direct inhibition of sodium-potassium adenosine triphosphatase (Na/K-ATPase)
- Negative chronotropic effects: decreased conduction velocity through the atrioventricular node, an increase in the effective refractory period and an increase in vagal activity leading to sinus node depression.

Clinically, digoxin is commonly prescribed in two conditions, heart failure and AF.

Bisoprolol

Bisoprolol fumarate is a highly beta-1 selective adrenoreceptor blocker first approved by the U.S. Food and Drug Administration in 1992. The cardiac effects of bisoprolol therapy are summarised by:

- Negative chronotropic effects: a reduction in resting and exercise heart rate due to prevention of norepinephrine and epinephrine from binding to the beta-receptor in cardiac conduction tissue.
- Negative (mild) inotropic effects: an initial fall in resting and exercise cardiac output with little observed change in stroke volume and only a small increase in right atrial pressure or pulmonary capillary wedge pressure.

Clinically, bisoprolol is commonly prescribed in a range of cardiology conditions, including post-myocardial infarction, heart failure and in patients with atrial tachyarrhythmia, including AF.

7.2 Treatment Supply and Storage

Due to the participant population and the fact that the trial closely aligns with standard care, trial medication may be dispensed from routine standard stock by both the pharmacy at the research site and community pharmacies local to the participant. Both treatments are used as per normal clinical practice therefore there is no additional requirement, above that of local policy, to monitor temperature during storage.

For peer review only - http://bmjopen.bmj.com/site/about/guidelines.xhtml

Digoxin

Digoxin is available as an oral tablet in doses of 62.5, 125 and 250 µg or as an elixir (50 µg/mL). It is packaged in 28 or 500 tablet packs under the generic title digoxin and trade label Lanoxin.[69] Digoxin should be stored according to local policy.

Bisoprolol

Bisoprolol is available as an oral tablet in doses of 1.25, 2.5, 3.75, 5.0, 7.5 and 10 mg. It is packaged as 28 tablets under the generic title bisoprolol fumarate and trade labels Cardicor and Emcor.[69] Bisoprolol should be stored according to local policy.

7.3 Dosing Schedule

Digoxin

An advice sheet for the investigator is presented in **Appendix A.**

Trial maintenance doses will initially be 62.5 or 125 µg orally (at the clinician's discretion, taking into account age and renal function), with planned up-titration to 125/250 µg. The maximum trial dose will be 250 µg daily.

A single loading dose of four tablets (250 or 500 µg according to target maintenance dose) will be prescribed in digoxin-naïve participants. The clinician is permitted to omit the loading dose or prescribe a second, where necessary.

Unblinded serum digoxin concentrations will be assessed at visits 2 and 3, with results reported back to the relevant clinician(s). This process will assist in monitoring compliance, adjusting dosage in cases of low serum levels and avoiding toxicity.

Bisoprolol

An advice sheet for the investigator is presented in **Appendix B**

Trial starting doses will be 1.25 or 2.5 or 5 mg (at the clinician's discretion), with planned up-titration to 10 mg in increments of 1.25 or 2.5 mg. The maximum trial dose will be 15 mg daily. No loading dose is required.

Plasma concentrations have not shown to be associated with toxicity and are not part of standard clinical practice.

7.4 Drug Interactions and Contraindications

Digoxin

Following oral administration of digoxin, approximately 60–85% of the dose is usually absorbed, mainly from the small intestine. The onset of action is 0.5-2 hours and maximal effects occur in

For peer review only - http://bmjopen.bmj.com/site/about/guidelines.xhtml

2-6 hours. Digoxin has a large volume of distribution and approximately 20-30% of digoxin in blood is bound to plasma proteins. Metabolism is minimal but variable, with the majority of drug excreted unchanged in the urine by glomerular filtration and tubular secretion. With normal renal function, the elimination half-life is 34-44 hours which is prolonged in patients with renal failure by two to threefold. Dose adjustment is unnecessary in patients with hepatic impairment. Therapeutic plasma concentrations of digoxin have been described as 0.5-2.0 ng/mL.[70] In digoxin-naïve patients with normal renal function, approximately seven days are required to reach steady-state therapeutic concentrations if a loading dose is omitted. As such, the majority of clinicians prescribe one or two loading doses, totalling 500 to 1000 µg over 24 hours.

Caution is recommended in patients with electrolyte disturbance (due to increased risk of toxicity) and reduced doses are recommended in patients with renal impairment. There are no concerns in pregnancy or with breast-feeding, although dose adjustment may be required.

Contraindications for digoxin therapy include heart block, accessory pathway supraventricular tachycardia and a current diagnosis of obstructive hypertrophic cardiomyopathy, myocarditis or constrictive pericarditis.

Digoxin has been associated with a number of adverse effects, although data from randomised trials show little difference in comparison to placebo, apart from cases of toxicity (2% versus 0.9% respectively in the DIG trial of patients with HF)[71]. The most common side effects are gastrointestinal upset, dizziness, blurred vision, headache and rash. In toxic states (serum levels >2 ng/mL), digoxin is pro-arrhythmic and can aggravate heart failure, particularly with co-existent hypokalaemia. In cases of overdose, repeated early doses of activated charcoal may be given to reduce absorption and in severe toxicity, digoxin-specific antibody fragments are available as an intravenous infusion.

In rigorous assessment, drug interactions with digoxin have proved inconsistent.[72] Serum digoxin concentrations are increased by amiodarone, dronedarone, propafenone and quinidine but increased bioavailability with CCB and certain antibiotics (such as erythromycin and tetracycline) only occur in selected patients. The risk of toxicity increases with drugs that cause electrolyte disturbances, such as thiazide and loop diuretics.

Bisoprolol

Following oral administration of digoxin, the absolute bioavailability is approximately 80%, first pass metabolism of 20% and 30% protein binding. Peak plasma concentrations occur within 2-4 hours, the elimination half-life is 9-12 hours and steady state is attained within 5 days. Elimination occurs equally by renal and non-renal pathways with about 50% of the dose remaining unchanged in the urine.

Caution is recommended in patients with first-degree heart block, portal hypertension, diabetes, a history of obstructive airways disease, myasthenia gravis, a history of hypersensitivity and psoriasis, although many cardiologists use beta-blockers frequently in these groups with appropriate supervision. In pregnancy, beta-blockers may cause intra-uterine growth restriction,

For peer review only - http://bmjopen.bmj.com/site/about/guidelines.xhtml

neonatal hypoglycaemia, and bradycardia (although as above, these agents are frequently used in pregnancy). There is a theoretical risk of toxicity in breast feeding, although the amount present in milk is likely too small to affect infants. Abrupt withdrawal should be avoided, especially in cases of ischaemic heart disease. Up-titration should be more cautious in patients with renal or hepatic impairment.

Contraindications for bisoprolol therapy include cardiogenic shock, overt cardiac failure, second or third degree heart block, marked sinus bradycardia and severe peripheral arterial disease.

Bisoprolol has been associated with a wide variety of adverse effects although data from RCTs suggest similar discontinuation rates compare to placebo.[5, 73] The most common adverse symptoms are lethargy, headache, peripheral oedema, upper respiratory tract symptoms, gastrointestinal upset and dizziness. In cases of overdose, bradycardia, hypotension, congestive heart failure, bronchospasm and hypoglycaemia may be expected, with treatment directed to supportive methods and atropine, fluids, glucagon or diuretics as required.

Pharmacokinetic interactions with beta-blockers have not shown to be clinically significant. Drugs that reduce absorption include aluminium salts and cholestyramine, whilst metabolism can be increased by barbiturates and rifampicin and decreased with cimetidine, erythromycin, fluvoxamine, and hydralazine.

7.5 Accountability Procedures and Labelling

Through the risk-adapted approach, a full risk assessment of the **RATE-AF** trial has been conducted including the drug accountability requirements. The IMPs will be used within their authorisations, prescribed on an NHS prescription and dispensed by pharmacy from standard stock. The risk assessment has determined that a normal dispensing label is appropriate and an additional clinical trial label is not necessary (as covered by Regulation 46 (2) of SI 2004/1031). Drug accountability will be according to standard practice for NHS prescriptions. Details of how compliance will be assessed can be found in **Section 7.7**.

7.6 Treatment Modification

Patients that withdraw from medication for any reason will do so under strict clinical supervision.

The trial is designed to assess the impact of **initial** impact of rate control therapy; it is expected that treatments will modify during the trial period (in particular, the addition of therapy to attain heart rate targets). Patients will not be withdrawn from the trial if they commence therapy from the other arm of the trial due to any absolute or relative clinical indications (for example, patients in the digoxin arm starting beta-blockers due to incident myocardial infarction, or heart failure with reduced LVEF).

For peer review only - http://bmjopen.bmj.com/site/about/guidelines.xhtml

7.7 Assessment of Compliance

We will ask participants about compliance with their trial medication at each follow-up visit and this will be documented in the CRFs. It may also be clinically evident from the heart rate check, performed as part of all visits, whether or not the patient has been compliant with their trial mediation.

In addition, patients that are randomised to the digoxin arm will have a serum digoxin sample taken as part of Visit 2 (month 6) and Visit 3 (month 12) follow-ups. The results will indicate whether the patient has been compliant with their trial medication.

8 Trial Procedures and Schedule of Assessments

8.1 Baseline Visit

The baseline visit will occur as soon as possible after screening and will involve the following procedures (see **Section 9** for procedure details):

- Verify inclusion/exclusion criteria.
- Obtain written informed consent from the potential participant.
- Randomise the patient via telephone or the secure web-based system as outlined in **Section 6**
- Administer QoL and functional capacity questionnaires.
- Review recent blood results (within 6 months of Baseline Visit)
    - Assessing renal function to aid in dose assignment and serum potassium level as part of standard clinical care.
- Document the use of oral anticoagulation and arrange appropriate prescription for patients not on therapy according to clinical guidelines. If the participant is already receiving vitamin-K antagonists (VKA), recent INR results will be documented.
- Record results of physical examinations.
- Collect blood samples for baseline blood tests and biomarker analysis.
- Complete case report form (CRF)
- Perform a 12-lead electrocardiogram.
- Supervise a 6-minute walk test, recording distance walked and peak heart rate.
- Arrange the baseline echocardiogram; images will be delivered to the echocardiographic core laboratory for blinded reporting.
- Discuss the randomised allocation with the participant including schedule for drug therapy and up-titration.

For peer review only - http://bmjopen.bmj.com/site/about/guidelines.xhtml

Participants will be followed up by telephone call two weeks after the Baseline Visit to ensure they have commenced trial medication.

8.2 Up-Titration Visits

For the majority of participants, two up-titration visits will be planned to supervise the appropriate use of medications as per the up-titration schedule (see **Appendices A and B**). Additional up-titration visits, as required, are acceptable in order to attain a heart rate at rest of ≤100bpm.

Up-titration visits will involve the following procedures:

- Record adverse events as reported by the participant or observed by the investigator.
- Review of medications and plan for trial drug up-titration
- Assessment of compliance
- Symptom-directed clinical examination
- Vital signs, including heart rate and blood pressure
- Administer QoL and functional capacity questionnaires (last uptitration visit only).
- Organise a 24-hour ambulatory ECG once up-titration completed (results to be forwarded to the clinician).

8.3 Visit 2, Month 6

Visit 2 will occur at an interval of six months (± four weeks) after the Baseline Visit and involve the following procedures:

- Administer QoL and functional capacity questionnaires.
- Record adverse events as reported by the participant or observed by the investigator.
- Confirm current rate control therapy (including dosage) and check concomitant medications.
- Assessment of compliance.
- Collect blood samples for biomarker analysis.
- Collect blood sample for serum digoxin concentration, potassium and creatinine as part of standard clinical care.
- Record time in therapeutic range for patients on anticoagulation with vitamin-K antagonists and compliance in patients receiving non-VKA oral anticoagulants.
- Obtain a twelve lead ECG.
- Record the results of physical examinations and vital signs.
- Supervise a 6-minute walk test, recording distance walked and peak heart rate.
- Complete other CRF requirements.
- If an echocardiogram has been performed for clinical reasons since the previous visit, images will be retrieved and sent to the core echocardiographic laboratory.

For peer review only - http://bmjopen.bmj.com/site/about/guidelines.xhtml

- Confirm appointment date for Visit 3.

8.4 Visit 3, Month 12 (Final Trial Assessment)

Visit 3 will occur at an interval of 12 months (± four weeks) after the Baseline Visit and involve the following procedures:

- Administer QoL and functional capacity questionnaires.
- Record adverse events as reported by the participant or observed by the investigator.
- Confirm current rate control therapy (including dosage) and check concomitant medications.
- Assessment of compliance.
- Transthoracic echocardiography (as per **Section 9.6**), with images delivered to the echocardiographic core laboratory for blinded reporting.
- Collect blood sample for serum digoxin concentration, potassium and creatinine as part of standard clinical care.
- Record time in therapeutic range for patients on anticoagulation with vitamin-K antagonists and compliance in patients receiving non-VKA oral anticoagulants.
- Obtain a twelve lead ECG.
- Record the results of physical examinations and vital signs.
- Supervise a 6-minute walk test, recording distance walked and peak heart rate.
- Complete other CRF requirements.
- If an echocardiogram has been performed for clinical reasons since the previous visit, images will be retrieved and sent to the core echocardiographic laboratory.
- Complete the end of trial standardised letter to the GP and clinician explaining that the participant has reached the end of the trial protocol and is no longer bound by their allocated medication strategy. Advise that all participants are invited for continued follow up and long term clinical outcome assessment.
- Provide final instructions to participant (e.g. follow-up of ongoing adverse events).

8.5 Investigator-blinded Endpoints

Investigator-blinded endpoints (PROMs, echocardiography and biomarkers) will be assessed by the core laboratory, identified only by the trial number. Ambulatory ECG and serum digoxin level will remain unblinded and results delivered to the responsible clinician.

8.6 Long Term Follow-Up

In patients who have agreed to NHS data linkage, a follow-up CRF will be completed. The CRF will capture items that include, but are not limited to death, hospital admissions and

For peer review only - http://bmjopen.bmj.com/site/about/guidelines.xhtml

cardiovascular events. The planned interval for outcome assessment is 2 and 5 years post-enrolment.

8.7 Withdrawal

Participants may withdraw at any time during the main **RATE-AF** trial if they choose not to continue or if their clinical team feel that continued participation in the trial is inappropriate.

An investigator may deem it necessary to withdraw a participant from the trial if:

1) Any clinical adverse event, laboratory abnormality, or other medical condition or situation occurs such that continued participation in the trial would not be in the best interest of the participant.
2) The participant meets an exclusion criterion (either newly developed or not previously recognised) that precludes further trial participation.

Full details of the reason(s) for withdrawal should be recorded on the Case Report Forms (CRFs) if healthcare professional-initiated, otherwise a simple statement reflecting patient preference will suffice.

Clear distinction will be made between withdrawals from trial treatments whilst allowing further follow-up, and any participants who refuse any follow-up. If a participant explicitly withdraws consent to any further data recording, then this decision will be respected. All communications surrounding the withdrawal will be noted in the participant's records and no further data will be collected for the participant.

In the case of missing echocardiographic outcome data due to withdrawal (but with consent for ongoing follow-up) or death, results of recent clinical echocardiography will be retrieved. The participant's permission to obtain such data will be obtained and documented during the consent process. As with all trial echocardiograms, the scan will be reported by the core echocardiographic laboratory. With respect to patient-reported outcomes, QoL questionnaires will be mailed to participants who withdraw from trial treatment but consent to ongoing follow up. Those patients where adverse symptoms were related to withdrawal will be invited to a focus group for further discussion.

8.8 Trial Duration

Patients will be on trial medication for 12 months and will be followed-up, during this period according to the protocol. At the end of the 12 months, the participants may, as determined by their clinician, continue on medication but it will not be considered part of the trial intervention. The trial will cease when the 12-month follow-up has been completed for the last participant recruited.

For peer review only - http://bmjopen.bmj.com/site/about/guidelines.xhtml

Table 1: Schedule of Assessments

Procedures		Baseline Visit	Up-titration Visits (Day 14 to 60)	Visit 2, Month 6 (± 4 weeks)	Visit 3, Month 12 (± 4 weeks)
Assessment of eligibility criteria		X			
Informed consent taken		X			
Review of medical history		X			
Review of medications		X	X	X	X
Physical exam	Complete	X			
	Symptom-directed		X	X	X
	Vital signs	X	X	X	X
Quality of life assessment		X	(X)	X	X
Functional and cognitive assessment		X		X	X
Transthoracic echocardiogram		X			X
12-lead electrocardiogram		X		X	X
6-minute walk test		X		X	X
24-hour ambulatory ECG			X	(X)	
Clinical labs	Chemistry	X		X	X
	Haematology	X		X	X
	Serum digoxin			(X)	(X)
Trial labs	BNP	X		X	
	Stored sample	X		X	
Assessment of compliance			X	X	X
Assessment of adverse events			X	X	X

Parentheses denote where a procedure is dependent on the stage of participants within the trial.

9 Trial Procedures

9.1 Procedures Defined as Standard Clinical Care

The following assessments are considered part of the standard clinical care of AF patients receiving heart rate control therapy and will occur at all trial visits:

- Blood tests for haemoglobin, serum creatinine, potassium and serum digoxin concentration; these will be obtained by the research nurse and submitted to the site-specific hospital laboratory as per local guidelines and SOPs, ensuring that all specimens are accurately labelled and handled appropriately. In the case of results requiring urgent action, local policies will be followed which may include the participant visiting their GP, local hospital or investigator. In all cases, appropriate trial documentation will be completed.
- A 12-lead ECG; these will be completed by appropriately trained local staff.

9.2 Medical History

Medical history will be obtained by interview and from medical records (physical and electronic) at the Baseline Visit comprising:

- Cardiovascular history, including prior ischaemic coronary disease, interventions and surgery, history of hypertension, heart failure or hyperlipidaemia, stroke or transient ischaemic attack, pulmonary embolus/deep vein thrombosis and peripheral vascular disease.
- AF history, including year of diagnosis, previous cardioversions, previous ablation therapy and anti-arrhythmic drug history.
- Pacemaker history, including date and reason for implantation, type of device (single-chamber, dual-chamber, biventricular, implanted defibrillator) and dependency.
- Non-cardiac history, including diabetes mellitus, airways disease (asthma/COPD), renal impairment, bleeding history and other major co-morbidities.
- Social and demographic history, including smoking status (current/ex/never), race (Caucasian/Indian subcontinent/Asian/African/other) and physical activity using the International Physical Activity Questionnaire (short form).

9.3 Medication History

Medications history will be assessed according to the categories below and include current dosage. Except for anticoagulation, antiarrhythmic and rate control therapies, only current medications will be included.

- Anticoagulation therapy (vitamin-K antagonists and novel agents), including past use, INR results and time in therapeutic range.
- Antiarrhythmic therapy, including past use.

For peer review only - http://bmjopen.bmj.com/site/about/guidelines.xhtml

- Rate control therapy (beta-blockers, digoxin, CCB), including past use.
- Antiplatelet therapy.
- Angiotensin-converting enzyme inhibitors or angiotensin receptor blockers.
- Aldosterone antagonists.
- Diuretics (loop, thiazide, potassium-sparing, others).
- Nitrates.
- Other anti-hypertensive/anti-anginal therapy.
- Statins.
- Other lipid-lowering medication.
- Diabetic medication and insulin.
- Asthma/COPD medication (including inhalers).
- Non-steroidal anti-inflammatory agents.

9.4 Physical Examination

Physical and vital signs will be assessed at all up-titration and trial visits. In most cases, a targeted physical examination will be performed, comprising of cardiovascular elements as summarised below:

- Heart rate (manual palpation at radial artery and apex).
- Heart sounds.
- Lung auscultation.
- Assessment of jugular venous pressure and/or peripheral oedema.
- Other focused examinations according to symptoms and complaints.
- Blood pressure (two measurements at the right brachial in a seated position preferred, using a validated oscillometric device).
- Height (Baseline Visit only), weight (all listed visits) and waist circumference (Baseline Visit; defined as the narrowest point between ribs and hips when viewed from the front after exhaling to the nearest centimetre).

9.5 Patient Reported Outcomes

9.5.1 Choice of Outcomes and Qualitative Research

A systematic review (according to and in collaboration with the COnsensus-based Standards for the selection of health Measurement Instruments, COSMIN[74]) is underway to evaluate PROMs in AF, with a focus on psychometric properties including internal consistency, reliability, and measurement error. Additional assessment and practical evaluation of PROMs will follow published guidance[75, 76], complementing qualitative research using patient focus groups, surveys

For peer review only - http://bmjopen.bmj.com/site/about/guidelines.xhtml

and directed interviews guided by the PROMs and qualitative research centres at the University of Birmingham.[77]

Instruments for assessment will be selected on the basis of overall validity, preferably in this patient population but including other groups where data are limited. Patient focus groups will allow exploration of patient perspectives on appropriate instruments that adequately reflect the experience of living with AF.[78] They will also allow comparison of QoL questionnaires that adequately summarise patient-prioritised components of their health and well-being. Additional focus groups and individual interviews will occur at interim and final follow-up during the trial. These aim to understand the patient experience of trial participation and processes, including the ease of completion of QoL questionnaires, relevance, reasons for non-completion and other feasibility issues that emerge during the trial e.g. non-compliance and recruitment, with reference to core outcome sets for this population.[79] A patient and public involvement (PPI) panel will contribute to all stages in the focus group process.[80]

This protocol was developed in accordance with the Standard Protocol Items for Randomized Trials [SPIRIT] statement[81], and the latest PROM-specific guidance from the International Society for Quality of Life Research (ISOQOL) Best Practice taskforce.[77, 82, 83]

9.5.2 Data Collection for PROMs

PROMs will be assessed at all main visits (Baseline, 2 and 3) and at the participants final up-titration visit (if applicable). The QoL tools used will be EQ-5D-5L, SF-36 and AFEQT. To avoid introducing co-intervention bias, all QoL data will be kept confidential and will not be used to inform clinical care.[84] Patients will be advised of this in the patient information sheet. PROMs will be collected at the start of each visit, before other trial procedures. In cases where the visit coincides with a clinician review, questionnaires should be completed in advance. The feasibility of using an online data collection tool will be explored, administered by trained research nurses and according to good-practice guidelines.[85] We will use this trial to perform an initial small-group assessment of electronic PROMs-equivalence to inform a future clinical event trial.

Qualitative research will be performed using a focus group of 10 volunteer patients enrolled at the start of the trial (5 in each randomised group). The focus group will meet after up-titration and then at 6 and 12 months. Detailed methods will be established before the first meeting, in collaboration with the University of Birmingham Qualitative Research Group.

All staff will receive training in QoL collection, with specific guidance on reducing introduced bias, minimising missing data and coaching participants to use the QoL software. Levels of missing PROMs data will be monitored. The site personnel responsible for collection of patient reported outcomes will be the Research Nurse under the supervision of the Principal Investigator.

For peer review only - http://bmjopen.bmj.com/site/about/guidelines.xhtml

9.5.3 Outcome Appraisal

Each QoL tool will be scored according to their published requirements (www.euroqol.org; www.sf-36.org; www.afeqt.org), using total and sub-category scores where appropriate.

To avoid dilution of effect over time, the primary analysis will be at six months (adjusting for baseline QOL and stratification variables). We have predefined a focus on physical well-being, which we hypothesize are where the greatest treatment effects will be observed, but will explore all aspects of QoL. Exploratory analysis of medication effects over the 12-month period will also be analysed and remain clinically important, as little data currently exists on the longer-term profile of QoL in AF.

Qualitative research outcomes will focus on the clinical responsiveness of the QoL instruments. The findings of the COSMIN systematic report will determine these outcomes and their relevant appraisal.

The RATE-AF trial will allow us to gain an initial understanding and framework of the patient experience of AF. We aim to begin the process of determining appropriate and responsive PROMs for AF patients and the optimum methods for delivery into a subsequent large-scale clinical trial.

9.6 Transthoracic Echocardiography

Echocardiography will be performed at Visits 1 and 3 and focus on systolic left-ventricular (LV) function, diastolic function and left-atrial assessment. Images will be obtained by an accredited echocardiographer. All trial echocardiograms will be labelled with the Trial Number and pseudoanonymised patient data, with specific instruction that the echocardiographer will remain blinded to the treatment assignment. All images will be archived to the core echocardiographic laboratory, with a copy retained in the site file.

9.6.1 Reproducibility and Validity of Measurements

Inter-observer and intra-observer variability in measurement will be assessed by comparing results of the stated methods discussed below across the cardiac cycle. To evaluate the minimum number of repeat measurements required that maintains clinical utility, reproducibility of single measurements will be compared to averages of 3/5/10 beats. This will also include the reliability of using an 'index beat' with a cycle length equivalent to a heart rate of 70-80 beats per minute, or with similar preceding and pre-preceding RR intervals.

9.6.2 Systolic LV Function

Systolic LV function will be determined by the following methods:

- Two-dimensional biplane Simpson's method utilising the simultaneous multi-planar approach to obtain LVEF in a single heartbeat (four and two-chamber views). In each

For peer review only - http://bmjopen.bmj.com/site/about/guidelines.xhtml

view, LV end-diastolic and end-systolic volumes (LVEDV, LVESV) are computed, with LVEF calculated as (LVEDV – LVESV) / LVEDV. Two-dimensional echocardiography has excellent spatial resolution but is limited by potential foreshortening of the ventricular apex and drop-out of the endocardial border.

- Standard Simpson's biplane method with four and two-chamber volumes obtained from separate heartbeats. This is the conventional method in current clinical use but is limited by varying RR intervals in AF.
- Fractional shortening on M-mode along the minor-axis of the left-ventricle (parasternal long-axis), calculated by the formula: (LV internal dimension in diastole - LV internal dimension in systole) / LV internal dimension in diastole. M-mode measurements are reproducible and easy to perform with excellent temporal resolution, but are limited in cases of regional wall motion abnormalities and in patients where the true minor-axis is difficult to visualise.
- Both automated endocardial tracking and speckle-tracking analysis will be utilised (where available) by the echocardiographic core laboratory. Multiple planes will be obtained (four-chamber, two-chamber and short-axis mid-ventricle views). These methods have the advantage of reducing operator time and are angle-independent, but rely on good ultrasound windowing. Global longitudinal systolic strain using 2D speckle-tracking has recently been proposed as an important marker for adverse cardiovascular outcomes in AF.[86]
- Three-dimensional full-volume analyses of LV function, with single-beat analysis where feasible. This method has the advantage of not relying on geometric assumptions and allows the acquisition of full volume data within a single heartbeat. It correlates well with gold standard methods such as cardiac magnetic resonance imaging, but relies on adequate ultrasound windowing.
- Peak S-wave on tissue Doppler imaging (TDI) of the mitral valve annular sub-endocardium. This method has good correlation with LVEF across a wide range of function and is obtainable in patients with poor acoustic windows, but is limited in cases of regional wall motion abnormality.

Where poor quality acoustic windows limit accurate assessment of LV function, use of an intravenous contrast agent is recommended in participants without known allergy.

9.6.3 Diastolic LV Function

Diastolic LV function will be determined using the following methods (in all cases repeated over 3-5 cardiac cycles):

- Mitral inflow pulse-wave Doppler peak E velocity and deceleration time (DT).
- Mitral annular TDI to calculate septal E', lateral E' and the individual and averaged E/E' ratios.
- LV outflow tract pulse-wave Doppler to calculate isovolumic relaxation time (IVRT).

For peer review only - http://bmjopen.bmj.com/site/about/guidelines.xhtml

- Pulmonary vein pulse-wave Doppler to calculate peak systolic (where present) and diastolic velocities, ratio of peak velocities and DT of diastolic PV flow.
- Colour M-mode Doppler assessment of mitral flow propagation velocity (Vp) and ratio of E/Vp.

Overall diastolic function will be categorised according to the British Society of Echocardiography guidelines into normal function or mild/moderate/severe dysfunction based on a combination of the above variables. Individual parameters will also be categorised using cut-points identified from published studies.[87]

9.6.4 Left Atrial Size and Function

Left atrial (LA) size will be measured in the anteroposterior (parasternal long-axis), transverse and longitudinal dimensions (apical 4-chamber). LA volumes will be calculated using the biplane area-length method: (0.85 x 4-chamber LA area x 2-chamber LA area) / LA length. The length is measured from the middle of the plane of the mitral annulus to the superior aspect of the LA (shortest of 4- and 2-chamber measurements). LA volumes will be indexed for body surface area.

Where suitable datasets are obtained, 3D LA volumetric analysis and assessment of LA function and strain will also be performed.

9.6.5 Additional Echocardiography Parameters

The following parameters will be obtained in all participants:

- Tricuspid annular plane systolic excursion (TAPSE) for estimation of right ventricular function using pulse-wave Doppler.
- Where applicable, mitral regurgitation dP/dt.

9.7 Laboratory Evaluations

The use of biomarkers than can affect treatment decisions in AF is at an early stage of development.[88] The RATE-AF trial will allow us to collect and store blood samples on patients at baseline and follow-up, providing a unique biobank of AF patients receiving rate-control. In collaboration with the Human Biomaterials Resource Centre (HBRC) at the University of Birmingham, we will also isolate DNA for future work on predictors of response, including known polymorphisms of rate-responsiveness.[89]

Laboratories at each clinical site will process the standard laboratory investigations required as part of standard clinical care (see **Section 9.1**). Trial laboratory evaluations will be performed at the core laboratory and processed according to the guidelines in **Sections 9.7.1**, **9.7.2** and **9.7.3**.

For peer review only - http://bmjopen.bmj.com/site/about/guidelines.xhtml

9.7.1 Laboratory Assays

NT-pro B-type natriuretic peptide will be analysed using a Sandwich immunoassay using monoclonal ruthenium labelled antibody and Roche Cobas 8000 e602. The total coefficient of variation for repeatability with this assay is <2% with an estimated volume of 250 microlitres required for each test and measurement range of 5‑35000 pg/mL (0.6‑4130 pmol/L).

9.7.2 Cellular Response to Rate Control

The effect of baseline and follow-up serum on intracellular sodium/calcium, force of contraction and activation of ERK1/2-dependent cascades will be examined in human induced pluripotent stem cell-derived cardiomyocytes, using well-established integrated fluorescence/contractility photometry and western blotting techniques.[90, 91] DigiFAB[92], will be used to determine whether changes are dependent on endogenous cardiotonic steroids, which can modulate intracellular ion concentration in cardiomyocytes[93, 94], and potentially contribute to treatment discontinuation (or the development of toxicity).[95] The concentration of serum cardiotonic steroids will be determined using liquid chromatography–tandem mass spectrometry. Individual change in cardiotonic steroids and intracellular sodium/calcium will be correlated with the change in heart rate, LVEF, B-type natriuretic peptide and quality of life. In addition, we will identify patterns in patients withdrawing from treatment or experiencing adverse reactions.

9.7.3 Stored Blood Samples

Blood samples will be stored at HBRC for future biomarker and genetic analysis, with participants providing explicit consent for this process. Any future use of these samples will be undertaken with ethical approval.

9.7.4 Specimen Preparation, Handling, Storage and Shipment

Specimens will be handled according to local standard operating procedures consisting of the time requirements for processing, required temperatures, aliquots of specimens, where they will be stored, how they will be labelled, the process for remnant samples/disposal and appropriate instructions for transportation.

9.8 Economic Evaluation

The RATE-AF trial will allow determination of the most appropriate data collection methods and ease of acquiring resource use and cost data for a subsequent outcomes trial. Specifically, how data is obtained from secondary care records, patient-reported resource use and the feasibility of obtaining primary care records. A preliminary economic evaluation from an NHS perspective will be performed to estimate costs over the 12-month period. The patient-level cost-analysis will determine all AF-related costs, with respect to trial interventions and secondary-care resource use (including adverse events) in the two arms of the trial. We will collect both cost and outcome data and present them in a cost-consequence analysis. Costing for this trial suggests that simplifying medication alone could result in a saving of £5900 over each 12-month period.

For peer review only - http://bmjopen.bmj.com/site/about/guidelines.xhtml

Considering the high and increasing prevalence of AF, this could result in a substantial NHS cost savings, particularly if adverse reactions are also reduced. The aim of this objective within the trial is to prepare the groundwork for a future cost-per-quality adjusted life year (QALY) analysis of rate-control in AF.

Costs of care will be derived from patient level resource-use data, focusing on secondary care costs, and including adverse effects, such as pacemaker implantation. Other major drivers of cost are hospitalisation (including visits to Accident & Emergency), unplanned outpatient visits and outpatient tests such as echocardiography or ambulatory ECG. The cost analysis will also consider therapy costs, both trial drug and additional treatments. Unit costs will be obtained from standard sources including NHS Reference Costs, Unit Costs of Health and Social Care[96] and health care providers. Total per-patient health care costs will initially be calculated thus allowing the estimation of mean costs per trial arm over 12 months follow-up. Responses to the EQ-5D-5L questionnaire at baseline, visit 2 (6 months) and visit 3 (12 months) will be used to plan a future QALY analysis.

Key feasibility elements are:

- Determining the best methods for obtaining hospitalisation data, including where participants have been hospitalised outside of research site
- Whether robust primary care costs can be estimated and the method(s) for acquiring this type of data
- How key cost drivers can be incorporated into data collection for any future trial

For peer review only

For peer review only - http://bmjopen.bmj.com/site/about/guidelines.xhtml

10 Pharmacovigilance

Definitions of different types of AE are listed in **Table 2**. The Investigator should assess the seriousness and causality (relatedness) of AEs experienced by the participant (this should be documented in the source data). For further information please refer to **Section 10.1**.

Table 2: Standard AE Definitions

Term	Definition
**Adverse Event (AE)**	Any untoward medical occurrence in a patient or clinical trial participant administered a medicinal product and which does not necessarily have a causal relationship with this treatment
**Adverse Reaction (AR)**	Any untoward and unintended response in a participant to an investigational medicinal product which is related to any dose administered to that participant
**Serious adverse event (SAE), serious adverse reaction (SAR) or unexpected serious adverse reaction**	Any adverse event, adverse reaction or unexpected adverse reaction, respectively, that:<br>• results in death;<br>• is life-threatening;<br>• requires hospitalisation or prolongation of existing hospitalisation;<br>• results in persistent or significant disability or incapacity; or<br>• consists of a congenital anomaly or birth defect
**Unexpected Adverse Reaction**	An adverse reaction the nature and severity of which is not consistent with the information about the medicinal product in question set out:<br>(a) in the case of a product with a marketing authorisation, in the summary of product characteristics for that product;<br>(b) in the case of any other investigational medicinal product, in the investigator's brochure relating to the trial in question.
**SUSAR**	Suspected Unexpected Serious Adverse Reaction

10.1 Recording and Assessment of Adverse Events

All adverse events will be reportable to the **RATE-AF** Trial Office up to 30 days post last IMP administration. Any SUSAR related to the IMP should to be reported irrespective of how long after IMP administration the reaction has occurred.

Adverse events will be recorded in the medical records and CRFs. Most AE/ARs that occur in this trial, whether they are serious or not, will be 'expected' treatment-related toxicities due to the drugs used in this trial.

For peer review only - http://bmjopen.bmj.com/site/about/guidelines.xhtml

Refer to **Table 3** for definition of expectedness.

Table 3: Expectedness

Category	Definition
**Expected**	An adverse event that is classed in nature as serious and which is consistent with the information about the IMP listed in the Investigator Brochure (or SmPC if Licensed IMP) or clearly defined in this protocol
**Unexpected**	An adverse event that is classed in nature as serious and which is not consistent with the information about the IMP listed in the Investigator Brochure (or SmPC if Licensed IMP)

Adverse events will be recorded with clinical symptoms and accompanied with a simple, brief description of the event, including dates as appropriate.

The assessment of relationship of adverse events to the administration of IMP is a clinical decision based on all available information at the time. The following categories as outlined in **Table 4** will be used to define the causality of the adverse event.

Table 4: Categorisation of Causality

Category	Definition
**Definitely**	There is clear evidence to suggest a causal relationship, and other possible contributing factors can be ruled out
**Probably**	There is evidence to suggest a causal relationship, and the influence of other factors is unlikely
**Possibly**	There is some evidence to suggest a causal relationship (e.g. the event occurred within a reasonable time after administration of the trial medication). However, the influence of other factors may have contributed to the event (e.g. the patient's clinical condition, other concomitant events)
**Unlikely**	There is little evidence to suggest there is a causal relationship (e.g. the event did not occur within a reasonable time after administration of the trial medication). There is another reasonable explanation for the event (e.g. the patient's clinical condition, other concomitant treatments)
**Not related**	There is no evidence of any causal relationship

The relevant SmPC for Digoxin and Bisoprolol (which will be dependent on which generic is being used according to local practice at each site) should be filed in the site file by the local research team.

For peer review only - http://bmjopen.bmj.com/site/about/guidelines.xhtml

The **RATE-AF** Trial protocol and the reference safety information will be used to assess disease related and/or expected events related to the trial treatment.

10.2 Non-Serious Adverse Events/ Adverse Reactions

Refer to Table 2 for definitions

Common adverse reactions (see Section 7.4) will be recorded on the relevant CRF and sent to the RATE-AF Trial Office.

10.3 Serious Adverse Events

Refer to Table 2 for definitions

All Serious Adverse Events (SAEs), that are not excluded from expedited reporting will be recorded in the hospital notes and should be reported to the **RATE-AF** Trial Office on a SAE Form. The completed form should be **faxed to the RATE-AF Trial Office on 0121 415 9135 or 0121 415 9136**, as soon as possible and ideally within one working day of becoming aware of the event. The site Investigator should be able to respond to any related queries raised by the **RATE-AF** Trial Office as soon as possible.

10.3.1 Expected SAEs NOT to be Reported on a SAE Form

Expected SAEs are those listed in the current SmPC for the trial IMPs and can be excluded from the expedited reporting outlined in **Section 10.1**, for example if they are expected to occur on a regular basis and offer no further new information to the safety profile. These events should continue to be recorded in the source data and relevant CRFs.

In addition, events **NOT** considered to be SAEs are hospitalisations for:

- Routine treatment or monitoring of the studied indication, not associated with any deterioration in condition
- Treatment, which was elective or pre-planned, for a pre-existing condition that is unrelated to the indication under trial, and has not worsened

Note: Death from any cause should be reported on an SAE Form and returned to the **RATE-AF** Trial Office.

10.4 SUSARs

Refer to Table 2 for definitions

SAEs classed by as both suspected to be related to the trial IMP and unexpected are categorised as SUSARs, and are always subject to expedited reporting. An SAE Form should be completed,

For peer review only - http://bmjopen.bmj.com/site/about/guidelines.xhtml

and faxed to the RATE-AF Trial Office within 24 hours of the research staff at site becoming aware of the event. The local investigator will provide the causality assessment.

The Chief Investigator (or nominated individual) will undertake urgent review of all such SAEs and may request further information immediately from the clinical team at site. The Chief Investigator will not overrule the causality or seriousness assessment given by the local investigator but may add additional comment on these. The Chief Investigator will provide the determination of expectedness according to the reference safety information.

SUSARs will be notified to the MHRA and REC by the RATE-AF Trial Office. SUSARs that are fatal or life-threatening will be notified to the MHRA and REC within 7 days after the **RATE-AF** Trial Office has been notified. Other SUSARs will be reported to the REC and MHRA within 15 days after the Trial Office has been notified.

10.5 Development Safety Update Reports

The **RATE-AF** Trial Office will provide the MHRA with Development Safety Update Reports (DSURs). The reports will be submitted within 60 days of the Developmental International Birth Date (DIBD) of the trial each year until the trial is declared ended.

10.6 Annual Progress Reports

An annual progress report will be submitted to the REC within 30 days of the anniversary date on which the favourable opinion was given, and annually until the trial is declared ended.

10.7 Pregnancy

Due to the age of participants that will be randomised into the **RATE-AF** Trial (≥ 60 years), it is highly improbable that female participants will be pregnant at the time of randomisation, or become pregnant during the trial. Any pregnancies will be followed up for outcome; any outcome meeting the definition of an SAE will be reported to the **RATE-AF** Trial Office on the relevant CRF.

10.8 Reporting Urgent Safety Measures

If any urgent safety measures are taken the Principal Investigator/BCTU/Sponsor shall immediately and in any event no later than 3 days from the date the measures are taken, give written notice to the MHRA and the REC of the measures taken and the circumstances giving rise to those measures.

For peer review only - http://bmjopen.bmj.com/site/about/guidelines.xhtml

11 Quality Control and Quality Assurance

11.1 Site Set-Up and Initiation

All participating Principal Investigators will be asked to sign the necessary agreements and supply a current CV to the Trials Office. All members of the site research team will also be required to sign a site signature and delegation log. Prior to commencing recruitment all sites will undergo a process of initiation and will have completed GCP training. Key members of the site research team will be required to attend either a meeting or a teleconference covering aspects of the trial design, protocol procedures, Adverse Event reporting, collection and reporting of data and record keeping. Sites will be provided with an Investigator Site File containing essential documentation, instructions, and other documentation required for the conduct of the trial. The Trials Office must be informed immediately of any change in the site research team.

11.2 Central Monitoring

Monitoring of this trial will be to ensure compliance with Good Clinical Practice. A risk proportionate approach to the initiation, management and monitoring of the trial will be adopted (as per the MRC/DH/MHRA Joint Project: Risk-adapted Approaches to the Management of Clinical Trials of Investigational Medicinal Products) and outlined in the trial-specific risk assessment.

The Trials Office will be in regular contact with the site research team to check on progress and address any queries that they may have. The Trials Office will check incoming CRFs for compliance with the protocol, data consistency, missing data and timing. Sites will be asked for missing data or clarification of inconsistencies or discrepancies. Sites will be requested to send in copies of signed Informed Consent Forms and other documentation for in-house review for all participants providing explicit consent.

Additional on-site monitoring visits may be triggered, for example by poor CRF return, poor data quality, low SAE reporting rates, excessive number of participant withdrawals or deviations. This will be detailed in the monitoring plan. If a monitoring visit is required the Trials Office will contact the site to arrange a date for the proposed visit and will provide the site with written confirmation. Investigators will allow the **RATE-AF** trial staff access to source documents as requested.

11.3 Audit and Inspection

The Principal Investigator will permit trial-related monitoring, quality checks, audits, ethical reviews, and regulatory inspection(s) at their site, providing direct access to source data/documents. The Principal Investigator will comply with these visits and any required follow up. Sites are also requested to notify the Trials Office of any MHRA inspections.

For peer review only - http://bmjopen.bmj.com/site/about/guidelines.xhtml

11.4 Notification of Serious Breaches

In accordance with Regulation 29A of the Medicines for Human Use (Clinical Trials) Regulations 2004 and its amendments the Sponsor of the trial is responsible for notifying the licensing authority in writing of any serious breach of the conditions and principles of GCP in connection with that trial or the protocol relating to that trial, within 7 days of becoming aware of that breach.

For the purposes of this regulation, a "serious breach" is a breach which is likely to effect to a significant degree the safety or physical or mental integrity of the participants of the trial; or the scientific value of the trial. Sites are therefore requested to notify the Trials office of any suspected trial-related serious breach of GCP and/or the trial protocol. Where the Trials Office is investigating whether or not a serious breach has occurred sites are also requested to cooperate with the Trials Office in providing sufficient information to report the breach to the MHRA where required and in undertaking any corrective and/or preventive action. Sites may be suspended from further recruitment in the event of serious and persistent non-compliance with the protocol and/or GCP, and/or poor recruitment. Any major problems identified during monitoring may be reported to Trial Management Group and Trial Oversight Committee, the REC and the relevant regulatory bodies. This includes reporting serious breaches of GCP and/or the trial protocol to the REC and MHRA. A copy is sent to the University of Birmingham Clinical Research Compliance Team at the time of reporting to the REC and/or relevant regulatory bodies.

11.5 Data Handling and Analysis

Paper CRFs must be completed, signed/ dated and either entered directly online or returned to the **RATE-AF** Trial Office by the PI or an authorised member of the site research team (as delegated on the **RATE-AF** Trial Signature and Delegation Log) within the timeframe listed in **Table 5**. Copies of all completed CRFs should be filed in the site file. Entries on paper CRFs should be made in ballpoint pen, in black ink, and must be legible. Any errors should be crossed out with a single stroke, the correction inserted and the change initialled and dated. If it is not obvious why a change has been made, an explanation should be written next to the change.

CRFs can be entered online at http://www.bctu.bham.ac.uk/RATEAF. Authorised staff at sites will require an individual secure login username and password to access this online data entry system.

Data reported on each CRF should be consistent with the source data or the discrepancies should be explained. If information is not known, this must be clearly indicated on the CRF. All missing and ambiguous data will be queried. All sections are to be completed.

CRF versions may be updated by the **RATE-AF** Trial Office, as appropriate, throughout the duration of the trial. Whilst this will not constitute a protocol amendment, new versions of the CRFs must be implemented by participating sites immediately on receipt.

For peer review only - http://bmjopen.bmj.com/site/about/guidelines.xhtml

It will be the responsibility of the Principal Investigator to ensure the accuracy of all data entered in the CRFs. The **RATE-AF** Trial Signature & Delegation Log will identify all those personnel with responsibilities for data collection.

Access to data, including the final trial dataset, will be limited to members of the Research Team.

The investigator(s)/institution(s) will permit trial-related monitoring, audits, REC review, and regulatory inspection(s), providing direct access to source data/documents. Trial participants are informed of this during the informed consent discussion and will consent to provide access to their medical notes.

Table 5: Data Collection Forms

Form Name	Schedule for submission
**Randomisation Form**	Collected at randomisation
**Baseline and Follow-Up CRFs**	As soon as possible after each follow-up assessment time point
**Serious Adverse Event Form**	Faxed within 24hrs of research staff at site becoming aware of event

11.6 End of Trial

The end of trial will be 30 days after the last data capture. This will allow sufficient time for the completion of protocol procedures, data collection and data input. The Trials Office will notify the MHRA and REC that the trial has ended within 90 days of the end of trial. Where the trial has terminated early, the Trials Office will inform the MHRA and REC within 15 days of the end of trial. The Trials Office will provide them with a summary of the clinical trial report within 12 months of the end of trial.

A copy of the end of trial notification as well as the summary report is also sent to the University of Birmingham Research Governance Team at the time of sending these are sent to the MHRA and REC.

11.7 Archiving

Archiving will be authorised by the BCTU on behalf of the Sponsor following submission of the end of trial report.

For peer review only - http://bmjopen.bmj.com/site/about/guidelines.xhtml

Principal Investigators are responsible for the secure archiving of essential trial documents (for their site) as per their NHS Trust policy. All essential documents will be archived for a minimum of 25 years after completion of trial.

Destruction of essential documents will require authorisation from the BCTU on behalf of the Sponsor.

12 Statistical Considerations

12.1 Outcome measures

12.1.1 Primary Outcome

Patient-reported quality of life (QoL) - SF-36 physical component summary score at six months

12.1.2 Secondary Outcomes

Patient-reported QoL:

- SF-36 global and domain-specific scores at 6 and 12 months
- EQ-5D-5L summary index and visual analogue scale at six and twelve months
- AFEQT overall score at six and twelve months

Cardiac function:

- Echocardiographic LVEF at 12 months
- Diastolic function (E/e' and composite of diastolic indices) at 12 months
- Functional assessment:
- Six-minute walking distance at 6 and 12 months
- Change in European Heart Rhythm Association (EHRA) class at 6 and 12 months

Biomarkers:

- Change in B-type natriuretic peptide (BNP) levels at 6 months
- Change in heart rate using 24-hour ambulatory ECG

12.1.3 Feasibility Outcomes

- Recruitment target of 3 patients per week across all participating centres.
- Compliance and reasons for non-compliance
- Number of withdrawals and losses to follow-up (with reasons)
- Drug discontinuation rate and adverse reactions requiring drug discontinuation.
- Number of patients needing therapy-induced requirement for additional treatment
- Population-specific standard deviations (SD) and proportions

For peer review only - http://bmjopen.bmj.com/site/about/guidelines.xhtml

- *SD of SF36 physical functioning score at 6 and 12 months*
- *SD of SF36 overall score at 6 and 12 months*
- *SD of AFEQT overall score at 6 and 12 months*
- *SD of LVEF and E/e' score at 6 and 12 months*
- *Unplanned hospitalisation admissions rates*

- Cardiovascular Events (particularly mortality, thromboembolic events, myocardial infarction and cardiovascular interventions)

The final analyses will follow a pre-specified analysis plan, drafted in conjunction with the Birmingham Clinical Trials Unit and submitted to the steering committee at the penultimate meeting. We intend to perform a primary intention-to-treat analysis, in addition to a per-protocol analysis.

Any additional (exploratory) analyses will be explicitly labelled as such in any subsequent manuscript.

12.2 Power Calculations

Randomising 144 patients we can assume an 85% power to detect an effect size of half a standard deviation in a continuous outcome measure of QoL (two-sided alpha of 0.05). A sample size of 160 patients would account for an estimated 10% loss to follow-up (including withdrawal and death prior to 12-month assessment). There is some evidence from existing research to support the notion that the treatment effect could be this large. The mean SF-36 role-physical score from the rate-control arm of the RACE study was 47, with a 17% improvement with rate-control over time.[62] In another study, CCB resulted in 22% improvement in a proprietary symptom-checklist, compared to a non-significant 8% change in those assigned to beta-blockers (SD 10-points in both groups). These values are also consistent with a 17% improvement in SF-36 scores in a third trial, PIAF.[63] Thus whilst it is possible that this trial may provide a clear indication of effect, it is accepted that the trial will be underpowered to detect smaller differences, reinforcing the requirement for a larger definitive trial which would also be powered to assess impact on clinical event rates.

12.3 Statistical analysis

A separate Statistical Analysis Plan for the RATE-AF trial provides a detailed description of the planned statistical analyses. A brief outline of these analyses is given below.

The primary comparison groups will be composed of those who are randomised to digoxin group and those randomised to the beta-blockers group. All analyses will be based on the intention to treat principle, with all patients analysed in the arms to which they were allocated irrespective of compliance with the randomised allocated treatment, and all patients will be included in the analyses. We will, as a sensitivity analysis, conduct per-protocol analyses, where appropriate.

For peer review only - http://bmjopen.bmj.com/site/about/guidelines.xhtml

For all analyses, a p-value <0.05 will be considered statistically significant.

12.3.1 Primary outcome analysis

The primary outcome for this trial is the continuous SF36 physical functioning domain score at 6 months. This outcome will be analysed using an ANCOVA model adjusting for treatment arm, baseline score and all minimisation variables. Results will be presented as difference in means and 95% confidence intervals.

12.3.2 Feasibility and Secondary outcomes analysis

The feasibility and secondary outcomes for the trial comprise of a combination of both continuous and categorical (dichotomous) data items.

Categorical endpoints:

For outcomes which are categorical (dichotomous) in nature, the proportion of participants and percentages will be analysed between arms.

Logistic/Log-binomial regression models will be fitted (where appropriate) to adjust for treatment arm, baseline scores and all minimisation variables.

Results will be presented as odds ratios/relative risks and 95% confidence intervals.

Continuous endpoints:

Any outcomes that are continuous in nature will be analysed in the same way as the primary outcome.

12.3.3 Missing data and sensitivity analyses

Primary analysis will concentrate on available data only, with no attempt made to impute missing data. Where appropriate, sensitivity analyses will be carried out to examine the possible impact of missing data on the results (full details will be discussed within the Statistical Analysis Plan).

12.3.4 Interim analyses and Stopping rules

Analysis of the data with respect to efficacy and safety will be performed at 12 months and sent to Data Monitoring Committee (DMC); see **Section 16**. The DMC will outline and agree the stopping rules for the trial which will be documented in the DMC charter. It is likely that the Haybittle-Peto boundary will be used. This states that if an interim analysis shows a probability of less than 0.001 that the treatments are different, then the trial should be stopped early. This will be used alongside data on important secondary endpoints and all other relevant evidence. A DMC report and charter outlining the terms of reference (including information on stopping rules) will be agreed with the DMEC.

For peer review only - http://bmjopen.bmj.com/site/about/guidelines.xhtml

12.4 Final analysis

The final analysis for the RATE-AF trial will occur once the last randomised participant completes their 12-month follow-up.

13 Ethics and Regulatory Requirements

The trial will be performed in accordance with the recommendations guiding physicians in biomedical research involving human participants, adopted by the 18th World Medical Association General Assembly, Helsinki, Finland, June 1964, amended at the 48th World Medical Association General Assembly, Somerset West, Republic of South Africa, October 1996 (website: http://www.wma.net/en/30publications/10policies/b3/index.html).

The trial will be conducted in accordance with the Research Governance Framework for Health and Social Care, the applicable UK Statutory Instruments, (which include the Medicines for Human Use Clinical Trials 2004 and subsequent amendments and the Data Protection Act 1998 and Human Tissue Act 2008, EU Clinical Trials Directive and amendment Regulations as appropriate) and Guidelines for Good Clinical Practice (GCP). This trial will be carried out under a Clinical Trial Authorisation in accordance with the Medicines for Human Use Clinical Trials regulations. The protocol will be submitted to and approved by the REC prior to circulation.

Before any participants are enrolled into the trial, the Principal Investigator at each site is required to obtain local R&D approval. Sites will not be permitted to enrol participants until written confirmation of R&D approval is received by the Principal Investigator.

It is the responsibility of the Principal Investigator to ensure that all subsequent amendments gain the necessary local approval. This does not affect the individual clinicians' responsibility to take immediate action if thought necessary to protect the health and interest of individual participants.

Within 90 days after the end of the trial, the Chief Investigator/Sponsor will ensure that the REC and the MHRA are notified that the trial has finished. If the trial is terminated prematurely, those reports will be made within 15 days after the end of the trial. The Chief Investigator will provide the Sponsor with a summary report of the clinical trial, which will then be submitted to the MHRA and REC within one year after the end of the trial.

14 Oversight Committees

14.1 Trial Management Group

The Trial Management Group (TMG) will comprise the CI, other lead investigators (clinical and non-clinical) and members of the BCTU. The TMG will be responsible for the day-to-day running and management of **RATE-AF**. The TMG will convene at regular intervals.

For peer review only - http://bmjopen.bmj.com/site/about/guidelines.xhtml

14.2 Trial Oversight Committee

A joint oversight committee comprising a Trial Steering Committee (TSC) and Data Monitoring Committee (DMC) will be engaged for this trial.

The role of the TSC is to provide the overall supervision of the trial. The TSC will monitor trial progress and conduct and advice on scientific credibility. The TSC will consider and act, as appropriate, upon the recommendations of the Data Monitoring Committee. Further details of the remit and role of the TSC are available in the TSC Charter.

An independent DMC will be established to oversee the safety of participants in the trial. The DMC will meet prior to the trial opening to enrolment, and then meet at least annually, or as per a timetable agreed by the DMC prior to trial commencement. Data analyses will be supplied in confidence to the DMC, which will be asked to give advice on whether the accumulated data from the trial, together with the results from other relevant research, justifies the continuing recruitment of further participants. The DMC will operate in accordance with the trial specific charter.

14.3 Protocol amendments

Where important protocol modifications are required as a result of oversight (for example, changes to eligibility criteria, outcomes or analyses), this information will be communicated to relevant parties, such as investigators, the REC, trial registries and regulators.

15 Finance

The **RATE-AF** Trial is funded through a Career Development Fellowship awarded to the Chief Investigator by the National Institute for Health Research (NIHR).

16 Confidentiality and Data Protection

Personal data recorded on all documents will be regarded as strictly confidential and will be handled and stored in accordance with the Data Protection Act 1998.

Participants will be identified using their unique trial identification number, date of birth and hospital number on the CRFs. and correspondence between the Trials Office and the participating site. Participants will give their explicit consent for the movement of their consent form, giving permission for the Trials Office to be sent a copy. This will be used to perform in-house monitoring of the consent process.

The Investigator must maintain documents not for submission to the Trials Office (e.g. Participant Identification Logs) in strict confidence. In the case of specific issues and/or queries from the

For peer review only - http://bmjopen.bmj.com/site/about/guidelines.xhtml

regulatory authorities, it will be necessary to have access to the complete trial records, provided that participant confidentiality is protected.

The Trials Office will maintain the confidentiality of all participants' data and will not disclose information by which participants may be identified to any third party other than those directly involved in the treatment of the participant and organisations for which the participant has given explicit consent for data transfer (e.g. competent authority, sponsor). Representatives of the **RATE-AF** Trials Office and sponsor may be required to have access to participant's notes for quality assurance purposes but participants should be reassured that their confidentiality will be respected at all times.

17 Insurance and Indemnity

The University of Birmingham has in place Clinical Trials indemnity coverage for this trial which provides cover to the University for harm which comes about through the University's, or its staff's, negligence in relation to the design or management of the trial and may alternatively, and at the University's discretion provide cover for non-negligent harm to participants.

With respect to the conduct of the trial at the Clinical Site and other clinical care of the patient, responsibility for the care of the patients remains with the NHS organisation responsible for the Clinical Site and is therefore indemnified through the NHS Litigation Authority.

The University of Birmingham is independent of any pharmaceutical company, and as such it is not covered by the Association of the British Pharmaceutical Industry (ABPI) guidelines for participant compensation.

18 Dissemination and Publication

Regular newsletters will keep collaborators informed of trial progress and regular meetings will be held to report the progress of the trial and to address any problems encountered in the conduct of the trial. The CI will coordinate dissemination of data from this trial. All publications and presentations, including abstracts, relating to the main trial will be authorised by the **RATE-AF** TMG. The results of the analysis will be published in the name of the **RATE-AF** Collaborative Group in a peer reviewed journal (provided that this does not conflict with the journal's policy).

Named authors must satisfy the International Committee of Medical Journal Editors (ICMJE) criteria for authorship (contribute to drafting of the article or revision for important intellectual content), provide timely approval of the final version to be published and supply detailed statements on any potential conflict of interest or financial relationship (http://www.icmje.org/). Members of the group who do not fulfil ICMJE criteria for authorship will be listed in the article appendix. Trial participants will be sent a lay summary of the final results of the trial, which will contain a reference to the full paper.

For peer review only - http://bmjopen.bmj.com/site/about/guidelines.xhtml

19 Statement of Compliance

The **RATE-AF** trial will be conducted in compliance with the approved protocol, EU GCP and the applicable regulatory requirements.

For peer review only

For peer review only - http://bmjopen.bmj.com/site/about/guidelines.xhtml

20 References

1. Camm AJ, Kirchhof P, Lip GY, Schotten U, Savelieva I, Ernst S, Van Gelder IC, Al-Attar N, Hindricks G, Prendergast B, Heidbuchel H, Alfieri O, Angelini A, Atar D, Colonna P, De Caterina R, De Sutter J, Goette A, Gorenek B, Heldal M, Hohloser SH, Kolh P, Le Heuzey JY, Ponikowski P, Rutten FH. Guidelines for the management of atrial fibrillation: the Task Force for the Management of Atrial Fibrillation of the European Society of Cardiology (ESC). *Eur Heart J*. 2010;31:2369-2429

2. Anderson JL, Halperin JL, Albert NM, Bozkurt B, Brindis RG, Curtis LH, DeMets D, Guyton RA, Hochman JS, Kovacs RJ, Ohman EM, Pressler SJ, Sellke FW, Shen W-K. Management of patients with atrial fibrillation (Compilation of 2006 ACCF/AHA/ESC and 2011 ACCF/AHA/HRS recommendations). *Circulation*. 2013;127:1916-1926

3. Camm AJ, Lip GY, De Caterina R, Savelieva I, Atar D, Hohnloser SH, Hindricks G, Kirchhof P, E. S. C. Committee for Practice Guidelines. 2012 focused update of the ESC Guidelines for the management of atrial fibrillation: an update of the 2010 ESC Guidelines for the management of atrial fibrillation. Developed with the special contribution of the European Heart Rhythm Association. *Eur Heart J*. 2012;33:2719-2747

4. National Institute for Health and Care Excellence. Atrial fibrillation: the management of atrial fibrillation. *NICE clinical guideline 180*. 2014; Accessed 15/09/2014; http://www.nice.org.uk/guidance/cg180/

5. Kotecha D, Holmes J, Krum H, Altman DG, Manzano L, Cleland JG, Lip GY, Coats AJ, Andersson B, Kirchhof P, von Lueder TG, Wedel H, Rosano G, Shibata MC, Rigby A, Flather MD, Beta-Blockers in Heart Failure Collaborative Group. Efficacy of beta blockers in patients with heart failure plus atrial fibrillation: an individual-patient data meta-analysis. *Lancet*. 2014;384:2235-2243

6. Ziff OJ, Lane DA, Samra M, Griffith M, Kirchhof P, Lip GY, Steeds RP, Townend J, Kotecha D. Safety and efficacy of digoxin: systematic review and meta-analysis of observational and controlled trial data. *BMJ*. 2015;351:h4451

7. Kotecha D, Banerjee A, Lip GY. Increased stroke risk in atrial fibrillation patients with heart failure: does ejection fraction matter? *Stroke*. 2015;46:608-609

8. Kotecha D, Kirchhof P. Rate and rhythm control have comparable effects on mortality and stroke in atrial fibrillation but better data are needed. *Evid Based Med*. 2014;19:222-223

9. Kotecha D, Piccini JP. Atrial fibrillation in heart failure: what should we do? *Eur Heart J*. 2015;36:3250-3257

10. Senoo K, Lip GY, Lane DA, Buller HR, Kotecha D. Residual risk of stroke and death in anticoagulated patients according to the type of atrial fibrillation: AMADEUS Trial. *Stroke*. 2015;46:2523-2528

11. Kotecha D, Chudasama R, Lane DA, Kirchhof P, Lip GY. Atrial fibrillation and heart failure due to reduced versus preserved ejection fraction: A systematic review and meta-analysis of death and adverse outcomes. *Int J Cardiol*. 2016;203:660-666

12. Arain M, Campbell MJ, Cooper CL, Lancaster GA. What is a pilot or feasibility study? A review of current practice and editorial policy. *BMC Med Res Methodol*. 2010;10:67

13. Thabane L, Ma J, Chu R, Cheng J, Ismaila A, Rios LP, Robson R, Thabane M, Giangregorio L, Goldsmith CH. A tutorial on pilot studies: the what, why and how. *BMC Med Res Methodol*. 2010;10:1

14. Heeringa J, van der Kuip DAM, Hofman A, Kors JA, van Herpen G, Stricker BHC, Stijnen T, Lip GYH, Witteman JCM. Prevalence, incidence and lifetime risk of atrial fibrillation: the Rotterdam study. *Eur Heart J*. 2006;27:949-953

For peer review only - http://bmjopen.bmj.com/site/about/guidelines.xhtml

15. Colilla S, Crow A, Petkun W, Singer DE, Simon T, Liu X. Estimates of Current and Future Incidence and Prevalence of Atrial Fibrillation in the U.S. Adult Population. *Am J Cardiol*. 2013;112:1142-1147

16. Kim MH, Johnston SS, Chu BC, Dalal MR, Schulman KL. Estimation of total incremental health care costs in patients with atrial fibrillation in the United States. *Circ Cardiovasc Qual Outcomes*. 2011;4:313-320

17. Benjamin EJ, Wolf PA, D'Agostino RB, Silbershatz H, Kannel WB, Levy D. Impact of Atrial Fibrillation on the Risk of Death: The Framingham Heart Study. *Circulation*. 1998;98:946-952

18. Stewart S, Hart CL, Hole DJ, McMurray JJV. A population-based study of the long-term risks associated with atrial fibrillation: 20-year follow-up of the Renfrew/Paisley study. *Am J Med*. 2002;113:359-364

19. Marijon E, Le Heuzey JY, Connolly S, Yang S, Pogue J, Brueckmann M, Eikelboom J, Themeles E, Ezekowitz M, Wallentin L, Yusuf S. Causes of death and influencing factors in patients with atrial fibrillation: a competing-risk analysis from the randomized evaluation of long-term anticoagulant therapy study. *Circulation*. 2013;128:2192-2201

20. Chen LY, Sotoodehnia N, Buzkova P, Lopez FL, Yee LM, Heckbert SR, Prineas R, Soliman EZ, Adabag S, Konety S, Folsom AR, Siscovick D, Alonso A. Atrial fibrillation and the risk of sudden cardiac death: the atherosclerosis risk in communities study and cardiovascular health study. *JAMA Intern Med*. 2013;173:29-35

21. Dorian P, Jung W, Newman D, Paquette M, Wood K, Ayers GM, Camm J, Akhtar M, Luderitz B. The impairment of health-related quality of life in patients with intermittent atrial fibrillation: implications for the assessment of investigational therapy. *J Am Coll Cardiol*. 2000;36:1303-1309

22. Thrall G, Lane D, Carroll D, Lip GYH. Quality of Life in Patients with Atrial Fibrillation: A Systematic Review. *Am J Med*. 2006;119:448.e441-419

23. Sears SF, Serber ER, Alvarez LG, Schwartzman DS, Hoyt RH, Ujhelyi MR. Understanding Atrial Symptom Reports: Objective versus Subjective Predictors. *Pacing Clin Electrophysiol*. 2005;28:801-807

24. Dries D, Exner D, Gersh B, Domanski M, Waclawiw M, Stevenson L. Atrial fibrillation is associated with an increased risk for mortality and heart failure progression in patients with asymptomatic and symptomatic left ventricular systolic dysfunction: a retrospective analysis of the SOLVD trials. *J Am Coll Cardiol*. 1998;32:695-703

25. Wang TJ, Larson MG, Levy D, Vasan RS, Leip EP, Wolf PA, D'Agostino RB, Murabito JM, Kannel WB, Benjamin EJ. Temporal Relations of Atrial Fibrillation and Congestive Heart Failure and Their Joint Influence on Mortality: The Framingham Heart Study. *Circulation*. 2003;107:2920-2925

26. The Atrial Fibrillation Follow-up Investigation of Rhythm Management (AFFIRM) Investigators. A Comparison of Rate Control and Rhythm Control in Patients with Atrial Fibrillation. *N Engl J Med*. 2002;347:1825-1833

27. Van Gelder IC, Hagens VE, Bosker HA, Kingma JH, Kamp O, Kingma T, Said SA, Darmanata JI, Timmermans AJM, Tijssen JGP, Crijns HJGM. A Comparison of Rate Control and Rhythm Control in Patients with Recurrent Persistent Atrial Fibrillation. *N Engl J Med*. 2002;347:1834-1840

28. Olshansky B, Rosenfeld LE, Warner AL, Solomon AJ, O'Neill G, Sharma A, Platia E, Feld GK, Akiyama T, Brodsky MA, Greene HL. The Atrial Fibrillation Follow-up Investigation of Rhythm Management (AFFIRM) study: approaches to control rate in atrial fibrillation. *J Am Coll Cardiol*. 2004;43:1201-1208

29. Hohnloser SH, Kuck KH, Lilienthal J. Rhythm or rate control in atrial fibrillation--Pharmacological Intervention in Atrial Fibrillation (PIAF): a randomised trial. *Lancet*. 2000;356:1789-1794

For peer review only - http://bmjopen.bmj.com/site/about/guidelines.xhtml

30. Carlsson J, Miketic S, Windeler J, Cuneo A, Haun S, Micus S, Walter S, Tebbe U, Investigators S. Randomized trial of rate-control versus rhythm-control in persistent atrial fibrillation: the Strategies of Treatment of Atrial Fibrillation (STAF) study. *J Am Coll Cardiol*. 2003;41:1690-1696

31. de Denus S, Sanoski CA, Carlsson J, Opolski G, Spinler SA. Rate vs rhythm control in patients with atrial fibrillation: a meta-analysis. *Arch Intern Med*. 2005;165:258-262

32. Chatterjee S, Sardar P, Lichstein E, Mukherjee D, Aikat S. Pharmacologic rate versus rhythm-control strategies in atrial fibrillation: an updated comprehensive review and meta-analysis. *PACE*. 2013;36:122-133

33. Shelton RJ, Clark AL, Goode K, Rigby AS, Houghton T, Kaye GC, Cleland JG. A randomised, controlled study of rate versus rhythm control in patients with chronic atrial fibrillation and heart failure: (CAFE-II Study). *Heart*. 2009;95:924-930

34. Roy D, Talajic M, Nattel S, Wyse DG, Dorian P, Lee KL, Bourassa MG, Arnold JMO, Buxton AE, Camm AJ, Connolly SJ, Dubuc M, Ducharme A, Guerra PG, Hohnloser SH, Lambert J, Le Heuzey J-Y, O'Hara G, Pedersen OD, Rouleau J-L, Singh BN, Stevenson LW, Stevenson WG, Thibault B, Waldo AL. Rhythm Control versus Rate Control for Atrial Fibrillation and Heart Failure. *N Engl J Med*. 2008;358:2667-2677

35. Kong MH, Shaw LK, O'Connor C, Califf RM, Blazing MA, Al-Khatib SM. Is rhythm-control superior to rate-control in patients with atrial fibrillation and diastolic heart failure? *Ann Noninvasive Electrocardiol*. 2010;15:209-217

36. Wazni O, Wilkoff B, Saliba W. Catheter Ablation for Atrial Fibrillation. *N Engl J Med*. 2011;365:2296-2304

37. Jones DG, Haldar SK, Hussain W, Sharma R, Francis DP, Rahman-Haley SL, McDonagh TA, Underwood SR, Markides V, Wong T. A randomized trial to assess catheter ablation versus rate control in the management of persistent atrial fibrillation in heart failure. *J Am Coll Cardiol*. 2013;61:1894-1903

38. Kirchhof P, Breithardt G, Camm AJ, Crijns HJ, Kuck KH, Vardas P, Wegscheider K. Improving outcomes in patients with atrial fibrillation: rationale and design of the Early treatment of Atrial fibrillation for Stroke prevention Trial. *Am Heart J*. 2013;166:442-448

39. Steg PG, Alam S, Chiang C-E, Gamra H, Goethals M, Inoue H, Krapf L, Lewalter T, Merioua I, Murin J, Naditch-Brûlé L, Ponikowski P, Rosenqvist M, Silva-Cardoso J, Zharinov O, Brette S, Neill JO. Symptoms, functional status and quality of life in patients with controlled and uncontrolled atrial fibrillation: data from the RealiseAF cross-sectional international registry. *Heart*. 2012;98:195-201

40. Nabauer M, Gerth A, Limbourg T, Schneider S, Oeff M, Kirchhof P, Goette A, Lewalter T, Ravens U, Meinertz T, Breithardt G, Steinbeck G. The Registry of the German Competence NETwork on Atrial Fibrillation: patient characteristics and initial management. *Europace*. 2009;11:423-434

41. Segal JB, McNamara RL, Miller MR, Kim N, Goodman SN, Powe NR, Robinson K, Yu D, Bass EB. The evidence regarding the drugs used for ventricular rate control. *J Fam Practice*. 2000;49:47-59

42. Farshi R, Kistner D, Sarma JSM, Longmate JA, Singh BN. Ventricular rate control in chronic atrial fibrillation during daily activity and programmed exercise: a crossover open-label study of five drug regimens. *J Am Coll Cardiol*. 1999;33:304-310

43. Koh KK, Kwon KS, Park HB, Baik SH, Park SJ, Lee KH, Kim EJ, Kim SH, Cho SK, Kim SS. Efficacy and safety of digoxin alone and in combination with low-dose diltiazem or betaxolol to control ventricular rate in chronic atrial fibrillation. *Am J Cardiol*. 1995;75:88-90

44. Nikolaidou T, Channer KS. Chronic atrial fibrillation: a systematic review of medical heart rate control management. *Postgrad Med J*. 2009;85:303-312

For peer review only - http://bmjopen.bmj.com/site/about/guidelines.xhtml

45. Ulimoen SR, Enger S, Carlson J, Platonov PG, Pripp AH, Abdelnoor M, Arnesen H, Gjesdal K, Tveit A. Comparison of four single-drug regimens on ventricular rate and arrhythmia-related symptoms in patients with permanent atrial fibrillation. *Am J Cardiol*. 2013;111:225-230

46. Ulimoen SR, Enger S, Pripp AH, Abdelnoor M, Arnesen H, Gjesdal K, Tveit A. Calcium channel blockers improve exercise capacity and reduce N-terminal Pro-B-type natriuretic peptide levels compared with beta-blockers in patients with permanent atrial fibrillation. *Eur Heart J*. 2014;35:517-524

47. Khand AU, Rankin AC, Martin W, Taylor J, Gemmell I, Cleland JG. Carvedilol alone or in combination with digoxin for the management of atrial fibrillation in patients with heart failure? *J Am Coll Cardiol*. 2003;42:1944-1951

48. Vamos M, Erath JW, Hohnloser SH. Digoxin-associated mortality: a systematic review and meta-analysis of the literature. *Eur Heart J*. 2015; In press: 10.1093/eurheartj/ehv143

49. Gheorghiade M, Fonarow GC, van Veldhuisen DJ, Cleland JG, Butler J, Epstein AE, Patel K, Aban IB, Aronow WS, Anker SD, Ahmed A. Lack of evidence of increased mortality among patients with atrial fibrillation taking digoxin: findings from post hoc propensity-matched analysis of the AFFIRM trial. *Eur Heart J*. 2013;34:1489-1497

50. Friberg L, Hammar N, Rosenqvist M. Digoxin in atrial fibrillation: report from the Stockholm Cohort study of Atrial Fibrillation (SCAF). *Heart*. 2010;96:275-280

51. Flory JH, Ky B, Haynes K, S MB, Munson J, Rowan C, Strom BL, Hennessy S. Observational cohort study of the safety of digoxin use in women with heart failure. *BMJ Open*. 2012;2:e000888

52. Andrey JL, Romero S, Garcia-Egido A, Escobar MA, Corzo R, Garcia-Dominguez G, Lechuga V, Gomez F. Mortality and morbidity of heart failure treated with digoxin. A propensity-matched study. *Int J Clin Pract*. 2011;65:1250-1258

53. Lewis RV, Irvine N, McDevitt DG. Relationships between heart rate, exercise tolerance and cardiac output in atrial fibrillation: the effects of treatment with digoxin, verapamil and diltiazem. *Eur Heart J*. 1988;9:777-781

54. Tsuneda T, Yamashita T, Fukunami M, Kumagai K, Niwano S, Okumura K, Inoue H. Rate control and quality of life in patients with permanent atrial fibrillation: the Quality of Life and Atrial Fibrillation (QOLAF) Study. *Circ J*. 2006;70:965-970

55. Van Gelder IC, Groenveld HF, Crijns HJGM, Tuininga YS, Tijssen JGP, Alings AM, Hillege HL, Bergsma-Kadijk JA, Cornel JH, Kamp O, Tukkie R, Bosker HA, Van Veldhuisen DJ, Van den Berg MP. Lenient versus Strict Rate Control in Patients with Atrial Fibrillation. *N Engl J Med*. 2010;362:1363-1373

56. Groenveld HF, Crijns HJGM, Van den Berg MP, Van Sonderen E, Alings AM, Tijssen JGP, Hillege HL, Tuininga YS, Van Veldhuisen DJ, Ranchor AV, Van Gelder IC. The Effect of Rate Control on Quality of Life in Patients With Permanent Atrial Fibrillation: Data From the RACE II (Rate Control Efficacy in Permanent Atrial Fibrillation II) Study. *J Am Coll Cardiol*. 2011;58:1795-1803

57. Mulder BA, Van Veldhuisen DJ, Crijns HJGM, Tijssen JGP, Hillege HL, Alings M, Rienstra M, Groenveld HF, Van den Berg MP, Van Gelder IC. Lenient vs. strict rate control in patients with atrial fibrillation and heart failure: a post-hoc analysis of the RACE II study. *Eur J Heart Fail*. 2013;15:1311-1318

58. Groenveld HF, Tijssen JG, Crijns HJ, Van den Berg MP, Hillege HL, Alings M, Van Veldhuisen DJ, Van Gelder IC. Rate control efficacy in permanent atrial fibrillation: successful and failed strict rate control against a background of lenient rate control: data from RACE II (Rate Control Efficacy in Permanent Atrial Fibrillation). *J Am Coll Cardiol*. 2013;61:741-748

59. US Department of Health and Human Services Food and Drug Administration. Guidance for industry: patient-reported outcome measures: use in medical product development to support

For peer review only - http://bmjopen.bmj.com/site/about/guidelines.xhtml

labeling claims. www.fda.gov/downloads/Drugs/GuidanceComplianceRegulatoryInformation/Guidances/UCM193282.pdf. 2009

60. Rienstra M, Lubitz SA, Mahida S, Magnani JW, Fontes JD, Sinner MF, Van Gelder IC, Ellinor PT, Benjamin EJ. Symptoms and Functional Status of Patients With Atrial Fibrillation: State of the Art and Future Research Opportunities. *Circulation*. 2012;125:2933-2943

61. Pepine CJ. Effects of pharmacologic therapy on health-related quality of life in elderly patients with atrial fibrillation: a systematic review of randomized and nonrandomized trials. *Clin Med Insights Cardiol*. 2013;7:1-20

62. Hagens VE, Ranchor AV, Van Sonderen E, Bosker HA, Kamp O, Tijssen JGP, Kingma JH, Crijns HJGM, Van Gelder IC. Effect of rate or rhythm control on quality of life in persistent atrial fibrillation: Results from the Rate Control Versus Electrical Cardioversion (RACE) study. *J Am Coll Cardiol*. 2004;43:241-247

63. Grönefeld GC, Lilienthal J, Kuck K-H, Hohnloser SH. Impact of rate versus rhythm control on quality of life in patients with persistent atrial fibrillation: Results from a prospective randomized study. *Eur Heart J*. 2003;24:1430-1436

64. Ware Jr JE, Gandek B. Overview of the SF-36 Health Survey and the International Quality of Life Assessment (IQOLA) Project. *J Clin Epidemiol*. 1998;51:903-912

65. Herdman M, Gudex C, Lloyd A, Janssen M, Kind P, Parkin D, Bonsel G, Badia X. Development and preliminary testing of the new five-level version of EQ-5D (EQ-5D-5L). *Qual Life Res*. 2011;20:1727-1736

66. Devlin NJ, Krabbe PF. The development of new research methods for the valuation of EQ-5D-5L. *Eur J Health Econ*. 2013;14 Suppl 1:S1-3

67. Spertus J, Dorian P, Bubien R, Lewis S, Godejohn D, Reynolds MR, Lakkireddy DR, Wimmer AP, Bhandari A, Burk C. Development and validation of the Atrial Fibrillation Effect on QualiTy-of-Life (AFEQT) Questionnaire in patients with atrial fibrillation. *Circ Arrhythm Electrophysiol*. 2011;4:15-25

68. Dorian P, Burk C, Mullin CM, Bubien R, Godejohn D, Reynolds MR, Lakkireddy DR, Wimmer AP, Bhandari A, Spertus J. Interpreting changes in quality of life in atrial fibrillation: How much change is meaningful? *Am Heart J*. 2013;166:381-387.e388

69. Joint Formulary Committee. *British National Formulary*. London: BMJ Group and Pharmaceutical Press; 2013.

70. American Hospital Formulary Service. *Drug Information*. Bethesda: American Society of Health-System Pharmacists; 2013.

71. The Digitalis Investigation Group. The effect of digoxin on mortality and morbidity in patients with heart failure. *N Engl J Med*. 1997;336:525-533

72. Magnani B, Malini PL. Cardiac glycosides. Drug interactions of clinical significance. *Drug Safety*. 1995;12:97-109

73. Ko DT, Hebert PR, Coffey CS, Sedrakyan A, Curtis JP, Krumholz HM. Beta-blocker therapy and symptoms of depression, fatigue, and sexual dysfunction. *JAMA*. 2002;288:351-357

74. Terwee C, Mokkink L, Knol D, Ostelo RJG, Bouter L, Vet HW. Rating the methodological quality in systematic reviews of studies on measurement properties: a scoring system for the COSMIN checklist. *Qual Life Res*. 2012;21:651-657

75. Streiner DL, Norman GR. *Health measurement scales : a practical guide to their development and use*. Oxford ; New York: Oxford University Press; 2008.

For peer review only - http://bmjopen.bmj.com/site/about/guidelines.xhtml

76. Staniszewska S, Haywood KL, Brett J, Tutton L. Patient and public involvement in patient-reported outcome measures: evolution not revolution. *Patient*. 2012;5:79-87

77. Calvert M, Kyte D, Duffy H, Gheorghe A, Mercieca-Bebber R, Ives J, Draper H, Brundage M, Blazeby J, King M. Patient-reported outcome (PRO) assessment in clinical trials: a systematic review of guidance for trial protocol writers. *PLoS One*. 2014;9:e110216

78. McCabe PJ, Schumacher K, Barnason SA. Living with atrial fibrillation: a qualitative study. *J Cardiovasc Nurs*. 2011;26:336-344

79. Kirchhof P, Auricchio A, Bax J, Crijns H, Camm J, Diener H-C, Goette A, Hindricks G, Hohnloser S, Kappenberger L, Kuck K-H, Lip GYH, Olsson B, Meinertz T, Priori S, Ravens U, Steinbeck G, Svernhage E, Tijssen J, Vincent A, Breithardt G. Outcome parameters for trials in atrial fibrillation: Recommendations from a consensus conference organized by the German Atrial Fibrillation Competence NETwork (AFNET) and the European Heart Rhythm Association (EHRA). *Eur Heart J*. 2007;28:2803-2817

80. Haywood K, Brett J, Salek S, Marlett N, Penman C, Shklarov S, Norris C, Santana MJ, Staniszewska S. Patient and public engagement in health-related quality of life and patient-reported outcomes research: what is important and why should we care? Findings from the first ISOQOL patient engagement symposium. *Qual Life Res*. 2014: Epub ahead of print; PMID 25194573

81. Chan AW, Tetzlaff JM, Gotzsche PC, Altman DG, Mann H, Berlin JA, Dickersin K, Hrobjartsson A, Schulz KF, Parulekar WR, Krleza-Jeric K, Laupacis A, Moher D. SPIRIT 2013 explanation and elaboration: guidance for protocols of clinical trials. *BMJ*. 2013;346:e7586

82. Calvert M, Kyte D, von Hildebrand M, King M, Moher D. Putting patients at the heart of health-care research. *Lancet*. 2015;385:1073-1074

83. Kyte D, Duffy H, Fletcher B, Gheorghe A, Mercieca-Bebber R, King M, Draper H, Ives J, Brundage M, Blazeby J, Calvert M. Systematic evaluation of the patient-reported outcome (PRO) content of clinical trial protocols. *PLoS One*. 2014;9:e110229

84. Kyte D, Draper H, Calvert M. Patient-reported outcome alerts: ethical and logistical considerations in clinical trials. *JAMA*. 2013;310:1229-1230

85. Zbrozek A, Hebert J, Gogates G, Thorell R, Dell C, Molsen E, Craig G, Grice K, Kern S, Hines S. Validation of electronic systems to collect patient-reported outcome (PRO) data-recommendations for clinical trial teams: report of the ISPOR ePRO systems validation good research practices task force. *Value Health*. 2013;16:480-489

86. Su H-M, Lin T-H, Hsu P-C, Lee W-H, Chu C-Y, Lee C-S, Voon W-C, Lai W-T, Sheu S-H. Global left ventricular longitudinal systolic strain as a major predictor of cardiovascular events in patients with atrial fibrillation. *Heart*. 2013;99:1588-1596

87. Al-Omari MA, Finstuen J, Appleton CP, Barnes ME, Tsang TSM. Echocardiographic assessment of left ventricular diastolic function and filling pressure in atrial fibrillation. *Am J Cardiol*. 2008;101:1759-1765

88. Hijazi Z, Oldgren J, Siegbahn A, Granger CB, Wallentin L. Biomarkers in atrial fibrillation: a clinical review. *Eur Heart J*. 2013;34:1475-1480

89. Parvez B, Chopra N, Rowan S, Vaglio JC, Muhammad R, Roden DM, Darbar D. A common beta1-adrenergic receptor polymorphism predicts favorable response to rate-control therapy in atrial fibrillation. *J Am Coll Cardiol*. 2012;59:49-56

90. Mahmmoud YA, Shattock M, Cornelius F, Pavlovic D. Inhibition of K+ transport through Na+, K+-ATPase by capsazepine: role of membrane span 10 of the alpha-subunit in the modulation of ion gating. *PLoS One*. 2014;9:e96909

For peer review only - http://bmjopen.bmj.com/site/about/guidelines.xhtml

91. Pavlovic D, Hall AR, Kennington EJ, Aughton K, Boguslavskyi A, Fuller W, Despa S, Bers DM, Shattock MJ. Nitric oxide regulates cardiac intracellular Na(+) and Ca(2)(+) by modulating Na/K ATPase via PKCepsilon and phospholemman-dependent mechanism. *J Mol Cell Cardiol*. 2013;61:164-171

92. Pullen MA, Brooks DP, Edwards RM. Characterization of the neutralizing activity of digoxin-specific Fab toward ouabain-like steroids. *J Pharmacol Exp Ther*. 2004;310:319-325

93. Manunta P, Messaggio E, Casamassima N, Gatti G, Carpini SD, Zagato L, Hamlyn JM. Endogenous ouabain in renal Na(+) handling and related diseases. *Biochim Biophys Acta*. 2010;1802:1214-1218

94. Pavlovic D. The role of cardiotonic steroids in the pathogenesis of cardiomyopathy in chronic kidney disease. *Nephron Clin Pract*. 2014;128:11-21

95. Song H, Karashima E, Hamlyn JM, Blaustein MP. Ouabain-digoxin antagonism in rat arteries and neurones. *J Physiol*. 2014;592:941-969

96. Curtis L. Unit Costs of Health & Social Care 2012. *Personal Social Services Research Unit*. 2012:http://www.pssru.ac.uk/archive/pdf/uc/uc2012/full-with-covers.pdf

97. Jerosch-Herold M. Quantification of myocardial perfusion by cardiovascular magnetic resonance. *Journal of Cardiovascular Magnetic Resonance*. 2010;12:57

For peer review only

For peer review only - http://bmjopen.bmj.com/site/about/guidelines.xhtml

APPENDIX A – Dosing Schedule (Digoxin)

Randomised treatment arm: Group A

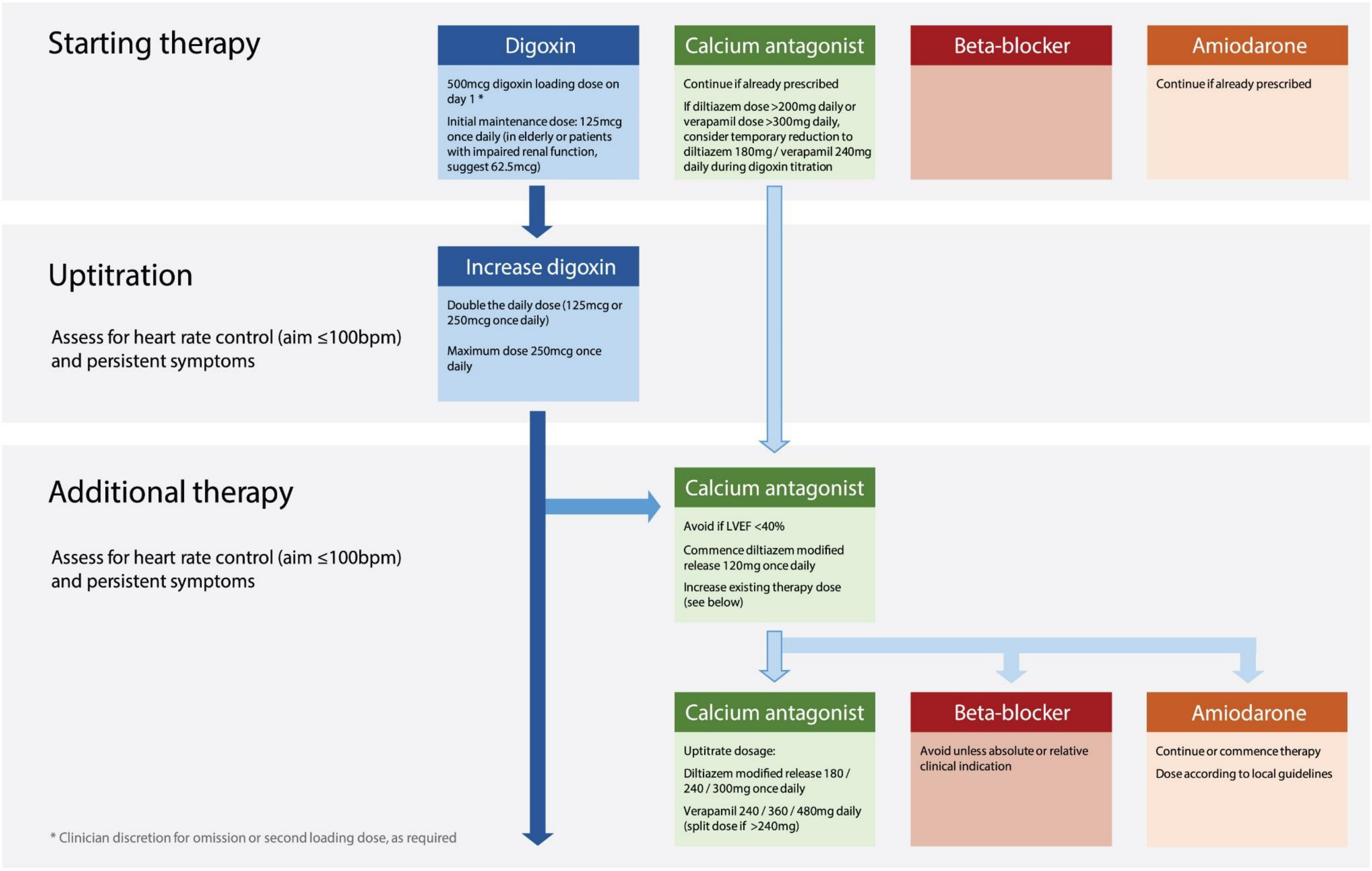

APPENDIX B – Dosing Schedule (Bisoprolol)

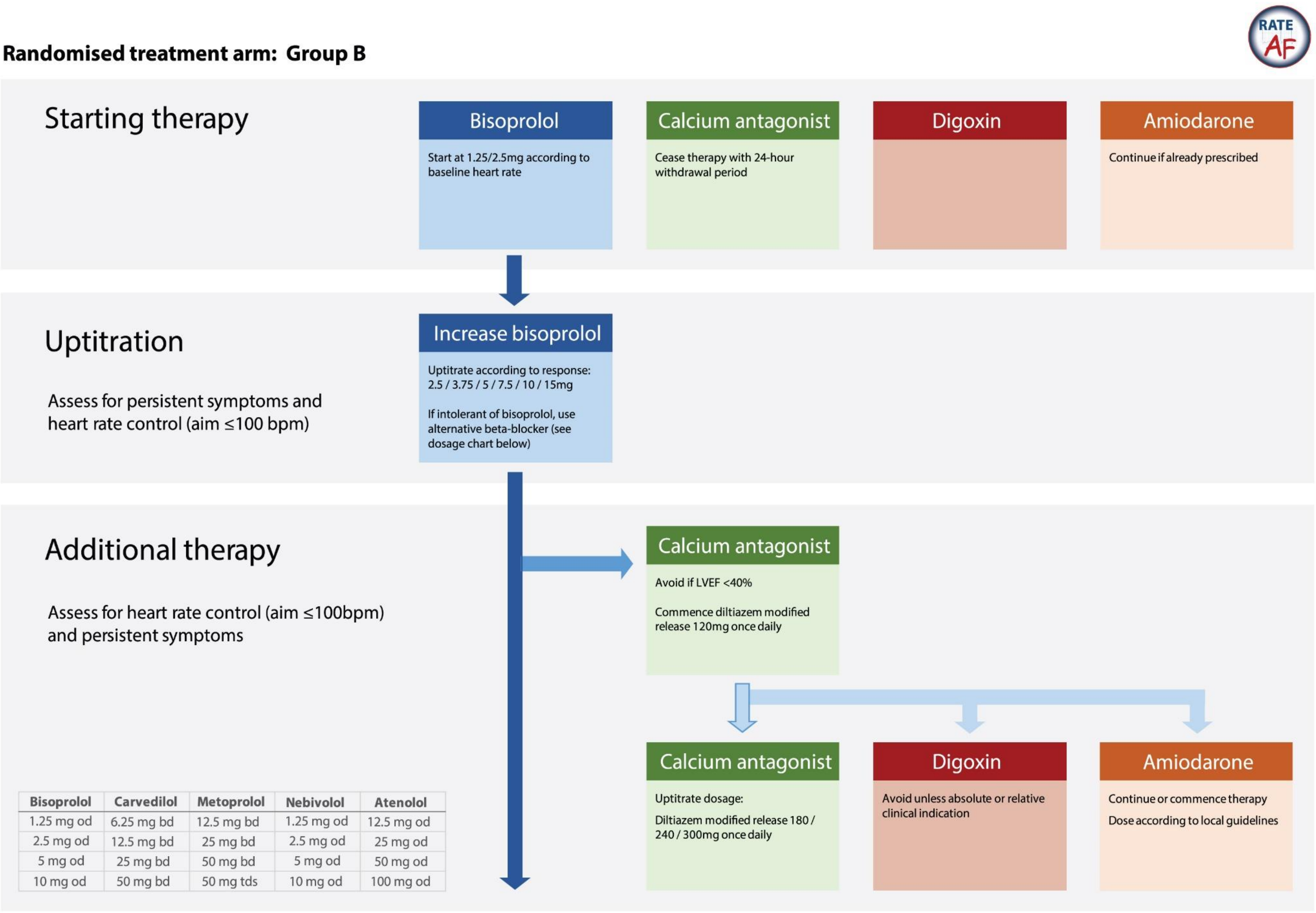

Bisoprolol	Carvedilol	Metoprolol	Nebivolol	Atenolol
1.25 mg od	6.25 mg bd	12.5 mg bd	1.25 mg od	12.5 mg od
2.5 mg od	12.5 mg bd	25 mg bd	2.5 mg od	25 mg od
5 mg od	25 mg bd	50 mg bd	5 mg od	50 mg od
10 mg od	50 mg bd	50 mg tds	10 mg od	100 mg od

For peer review only - http://bmjopen.bmj.com/site/about/guidelines.xhtml

SPIRIT 2013 Checklist: Recommended items to address in a clinical trial protocol and related documents*

Section/item	Item No	Description	Addressed on page number
**Administrative information**			
Title	1	Descriptive title identifying the study design, population, interventions, and, if applicable, trial acronym	________
Trial registration	2a	Trial identifier and registry name. If not yet registered, name of intended registry	________
	2b	All items from the World Health Organization Trial Registration Data Set	________
Protocol version	3	Date and version identifier	________
Funding	4	Sources and types of financial, material, and other support	________
Roles and responsibilities	5a	Names, affiliations, and roles of protocol contributors	________
	5b	Name and contact information for the trial sponsor	________
	5c	Role of study sponsor and funders, if any, in study design; collection, management, analysis, and interpretation of data; writing of the report; and the decision to submit the report for publication, including whether they will have ultimate authority over any of these activities	________
	5d	Composition, roles, and responsibilities of the coordinating centre, steering committee, endpoint adjudication committee, data management team, and other individuals or groups overseeing the trial, if applicable (see Item 21a for data monitoring committee)	________

For peer review only

For peer review only - http://bmjopen.bmj.com/site/about/guidelines.xhtml

**Introduction**			
Background and rationale	6a	Description of research question and justification for undertaking the trial, including summary of relevant studies (published and unpublished) examining benefits and harms for each intervention	______________
	6b	Explanation for choice of comparators	______________
Objectives	7	Specific objectives or hypotheses	______________
Trial design	8	Description of trial design including type of trial (eg, parallel group, crossover, factorial, single group), allocation ratio, and framework (eg, superiority, equivalence, noninferiority, exploratory)	______________
**Methods: Participants, interventions, and outcomes**			
Study setting	9	Description of study settings (eg, community clinic, academic hospital) and list of countries where data will be collected. Reference to where list of study sites can be obtained	______________
Eligibility criteria	10	Inclusion and exclusion criteria for participants. If applicable, eligibility criteria for study centres and individuals who will perform the interventions (eg, surgeons, psychotherapists)	______________
Interventions	11a	Interventions for each group with sufficient detail to allow replication, including how and when they will be administered	______________
	11b	Criteria for discontinuing or modifying allocated interventions for a given trial participant (eg, drug dose change in response to harms, participant request, or improving/worsening disease)	______________
	11c	Strategies to improve adherence to intervention protocols, and any procedures for monitoring adherence (eg, drug tablet return, laboratory tests)	______________
	11d	Relevant concomitant care and interventions that are permitted or prohibited during the trial	______________
Outcomes	12	Primary, secondary, and other outcomes, including the specific measurement variable (eg, systolic blood pressure), analysis metric (eg, change from baseline, final value, time to event), method of aggregation (eg, median, proportion), and time point for each outcome. Explanation of the clinical relevance of chosen efficacy and harm outcomes is strongly recommended	______________
Participant timeline	13	Time schedule of enrolment, interventions (including any run-ins and washouts), assessments, and visits for participants. A schematic diagram is highly recommended (see Figure)	______________

For peer review only - http://bmjopen.bmj.com/site/about/guidelines.xhtml

Sample size	14	Estimated number of participants needed to achieve study objectives and how it was determined, including clinical and statistical assumptions supporting any sample size calculations	______________
Recruitment	15	Strategies for achieving adequate participant enrolment to reach target sample size	______________

Methods: Assignment of interventions (for controlled trials)

Allocation:

Sequence generation	16a	Method of generating the allocation sequence (eg, computer-generated random numbers), and list of any factors for stratification. To reduce predictability of a random sequence, details of any planned restriction (eg, blocking) should be provided in a separate document that is unavailable to those who enrol participants or assign interventions	______________
Allocation concealment mechanism	16b	Mechanism of implementing the allocation sequence (eg, central telephone; sequentially numbered, opaque, sealed envelopes), describing any steps to conceal the sequence until interventions are assigned	______________
Implementation	16c	Who will generate the allocation sequence, who will enrol participants, and who will assign participants to interventions	______________
Blinding (masking)	17a	Who will be blinded after assignment to interventions (eg, trial participants, care providers, outcome assessors, data analysts), and how	______________
	17b	If blinded, circumstances under which unblinding is permissible, and procedure for revealing a participant's allocated intervention during the trial	______________

Methods: Data collection, management, and analysis

Data collection methods	18a	Plans for assessment and collection of outcome, baseline, and other trial data, including any related processes to promote data quality (eg, duplicate measurements, training of assessors) and a description of study instruments (eg, questionnaires, laboratory tests) along with their reliability and validity, if known. Reference to where data collection forms can be found, if not in the protocol	______________
	18b	Plans to promote participant retention and complete follow-up, including list of any outcome data to be collected for participants who discontinue or deviate from intervention protocols	______________

For peer review only - http://bmjopen.bmj.com/site/about/guidelines.xhtml

Data management	19	Plans for data entry, coding, security, and storage, including any related processes to promote data quality (eg, double data entry; range checks for data values). Reference to where details of data management procedures can be found, if not in the protocol	
Statistical methods	20a	Statistical methods for analysing primary and secondary outcomes. Reference to where other details of the statistical analysis plan can be found, if not in the protocol	
	20b	Methods for any additional analyses (eg, subgroup and adjusted analyses)	
	20c	Definition of analysis population relating to protocol non-adherence (eg, as randomised analysis), and any statistical methods to handle missing data (eg, multiple imputation)	
**Methods: Monitoring**			
Data monitoring	21a	Composition of data monitoring committee (DMC); summary of its role and reporting structure; statement of whether it is independent from the sponsor and competing interests; and reference to where further details about its charter can be found, if not in the protocol. Alternatively, an explanation of why a DMC is not needed	
	21b	Description of any interim analyses and stopping guidelines, including who will have access to these interim results and make the final decision to terminate the trial	
Harms	22	Plans for collecting, assessing, reporting, and managing solicited and spontaneously reported adverse events and other unintended effects of trial interventions or trial conduct	
Auditing	23	Frequency and procedures for auditing trial conduct, if any, and whether the process will be independent from investigators and the sponsor	
**Ethics and dissemination**			
Research ethics approval	24	Plans for seeking research ethics committee/institutional review board (REC/IRB) approval	
Protocol amendments	25	Plans for communicating important protocol modifications (eg, changes to eligibility criteria, outcomes, analyses) to relevant parties (eg, investigators, REC/IRBs, trial participants, trial registries, journals, regulators)	

For peer review only - http://bmjopen.bmj.com/site/about/guidelines.xhtml

Consent or assent	26a	Who will obtain informed consent or assent from potential trial participants or authorised surrogates, and how (see Item 32)	______________
	26b	Additional consent provisions for collection and use of participant data and biological specimens in ancillary studies, if applicable	______________
Confidentiality	27	How personal information about potential and enrolled participants will be collected, shared, and maintained in order to protect confidentiality before, during, and after the trial	______________
Declaration of interests	28	Financial and other competing interests for principal investigators for the overall trial and each study site	______________
Access to data	29	Statement of who will have access to the final trial dataset, and disclosure of contractual agreements that limit such access for investigators	______________
Ancillary and post-trial care	30	Provisions, if any, for ancillary and post-trial care, and for compensation to those who suffer harm from trial participation	______________
Dissemination policy	31a	Plans for investigators and sponsor to communicate trial results to participants, healthcare professionals, the public, and other relevant groups (eg, via publication, reporting in results databases, or other data sharing arrangements), including any publication restrictions	______________
	31b	Authorship eligibility guidelines and any intended use of professional writers	______________
	31c	Plans, if any, for granting public access to the full protocol, participant-level dataset, and statistical code	______________
**Appendices**			
Informed consent materials	32	Model consent form and other related documentation given to participants and authorised surrogates	______________
Biological specimens	33	Plans for collection, laboratory evaluation, and storage of biological specimens for genetic or molecular analysis in the current trial and for future use in ancillary studies, if applicable	______________

*It is strongly recommended that this checklist be read in conjunction with the SPIRIT 2013 Explanation & Elaboration for important clarification on the items. Amendments to the protocol should be tracked and dated. The SPIRIT checklist is copyrighted by the SPIRIT Group under the Creative Commons "Attribution-NonCommercial-NoDerivs 3.0 Unported" license.

BMJ Open

A review of rate control in atrial fibrillation, and the rationale and protocol for the RATE-AF trial

Journal:	*BMJ Open*
Manuscript ID	bmjopen-2016-015099.R1
Article Type:	Protocol
Date Submitted by the Author:	28-Feb-2017
Complete List of Authors:	Kotecha, Dipak; University of Birmingham, Institute of Cardiovascular Sciences; University Hospitals Birmingham NHS Trust, Cardiology<br>Calvert, Melanie; University of Birmingham, Centre for Patient Reported Outcomes Research; University of Birmingham, Institute of Applied Health Research<br>Deeks, Jon; University of Birmingham, Birmingham Clinical Trials Unit; University of Birmingham, Institute of Applied Health Research<br>Griffith, Mike; University Hospitals Birmingham NHS Trust, Cardiology<br>Kirchhof, Paulus; University of Birmingham, Institute of Cardiovascular Sciences; Sandwell & West Birmingham Hospitals NHS Trust, Cardiology<br>Lip, Gregory; University of Birmingham, Institute of Cardiovascular Sciences; Sandwell & West Birmingham Hospitals NHS Trust, Cardiology<br>Mehta, Samir; University of Birmingham, Birmingham Clinical Trials Unit<br>Slinn, Gemma; University of Birmingham, Birmingham Clinical Trials Unit<br>Stanbury, Mary; (Lead for the patient involvement panel)<br>Steeds, Richard; University Hospitals Birmingham NHS Trust, Cardiology; University of Birmingham, Institute of Cardiovascular Sciences<br>Townend, Jonathan; University Hospitals Birmingham NHS Trust, Cardiology; University of Birmingham, Institute of Cardiovascular Sciences
**Primary Subject Heading**:	Cardiovascular medicine
Secondary Subject Heading:	Evidence based practice, Medical management, Patient-centred medicine
Keywords:	Atrial fibrillation, heart rate, quality of life, Echocardiography < CARDIOLOGY, Protocols & guidelines < HEALTH SERVICES ADMINISTRATION & MANAGEMENT, RATE-AF

SCHOLARONE™
Manuscripts

Title: A review of rate control in atrial fibrillation, and the rationale and protocol for the RATE-AF trial

Brief Title: Kotecha *et al*, Rate control therapy evaluation in atrial fibrillation

Authors: Dr Dipak Kotecha MBChB PhD MRCP FESC FHEA[1,2,3,4]*, Prof Melanie Calvert BSc PhD FHEA[4,5], Prof Jonathan J Deeks BSc MSc PhD CStat[5,6], Dr Michael Griffith MD FRCP[2], Prof Paulus Kirchhof MD FRCP FESC[1,2,3,4], Prof Gregory YH Lip MD FRCP DFM FESC[1,3,4], Samir Mehta MSc BSc[6], Gemma Slinn BSc MPhil[6], Mary Stanbury RGN RDN RHV**, Dr Richard P Steeds MA MD FRCP FESC[1],[2] and Prof Jonathan N Townend BSc MBChB MD FESC[1,2].

From the (1) Institute of Cardiovascular Sciences, University of Birmingham, Birmingham, UK; (2) University Hospitals Birmingham NHS Trust, Birmingham, UK; (3) Sandwell & West Birmingham Hospitals NHS Trust, Birmingham, UK; (4) Centre for Patient Reported Outcomes Research, University of Birmingham, Birmingham, UK; (5) Institute of Applied Health Research, University of Birmingham, Birmingham, UK; and (6) Birmingham Clinical Trials Unit, University of Birmingham, Birmingham, UK. * Authors after the Chief Investigator listed in alphabetical order. ** Lead for the Patient Involvement Panel.

Address for correspondence:

Dr Dipak Kotecha

University of Birmingham Institute of Cardiovascular Sciences, Institute of Translational Medicine, Heritage Building, Queen Elizabeth Hospital Birmingham, B15 2TH, UK.

Email: d.kotecha@bham.ac.uk Tel: +44 121 371 8122 Fax: +44 121 554 4083

Word count (text): 2886 Word count (abstract): 298

For peer review only

Key Words: Atrial fibrillation; heart rate; Protocols & guidelines<health services administration & management; RATE-AF trial; quality of life; echocardiography<cardiology.

For peer review only

Abstract

Background & Objective: Atrial fibrillation (AF) is common and causes impaired quality of life, an increased risk of stroke and death, as well as frequent hospital admissions. The majority of patients with AF require control of heart rate. In this article we summarise the limited evidence from clinical trials that guides prescription, and present the rationale and protocol for a new randomised trial. As rate control has not, as yet, been shown to reduce mortality, there is a clear need to compare the impact of therapy on quality of life, cardiac function and exercise capacity. Such a trial should concentrate on the longer-term effects of treatment in the largest proportion of AF patients, those with symptomatic permanent AF, with the aim of improving patient well-being.

Design & Intervention: The RAte control Therapy Evaluation in permanent Atrial Fibrillation (RATE-AF) trial will enrol 160 participants with a prospective, randomised, open-label, blinded end-point design comparing initial rate control with digoxin or bisoprolol. This will be the first head-to-head randomised trial of digoxin and beta-blockers in AF.

Participants: Recruited patients will be aged ≥60 years with permanent AF and symptoms of breathlessness (NYHA Class II or above), with few exclusion criteria to maximise generalisability to routine clinical practice.

Outcome measures: The primary outcome is patient-reported quality of life, with secondary outcomes including echocardiographic ventricular function, exercise capacity and biomarkers of cellular and clinical response. Follow-up will occur at 6 and 12 months, with feasibility components to inform the design of a future trial powered to detect a difference in hospital admission. The RATE-AF trial will underpin an integrated approach to management including biomarkers, function and symptoms that will guide future research into optimal, personalised rate control in patients with AF.

Ethical approval: East Midlands-Derby Research Ethics Committee (16/EM/0178).

Trial registration: clinicaltrials.gov:NCT02391337; ISRCTN:95259705.

For peer review only

Strengths and limitations of this study

- Control of heart rate is universally used in patients with atrial fibrillation, but evidence from good quality randomised trials is extremely limited.
- Despite common clinical use, there has never been a direct randomised comparison of beta-blockers and digoxin for heart rate control in AF patients (with or without heart failure).
- The RATE-AF trial will assess the effect of therapy on patient-reported quality of life, and improve methods to capture this information in patients with AF. The trial will also evaluate the longer-term impact on cardiac function, define reproducible methods to measure systolic and diastolic function in AF, and develop new biomarkers for personalisation of treatment.
- The trial will not have the power to identify differences in clinical events, but will allow us to plan a future trial designed to detect a difference in the need for admissions to hospital.

For peer review only

Introduction

Atrial fibrillation (AF) is a common cause of stroke and cardiovascular death, leads to poor quality of life and doubles the risk of hospital admission.[1] We are currently in the midst of an epidemic of AF, with both incidence and prevalence expected to double in the next 20 years.[2-4] Although AF can affect any age-group, patients are typically elderly with significant comorbidities, including up to 50% suffering from heart failure.[5] AF is both a cause and consequence of heart failure, with complex interactions leading to impairment of systolic and diastolic function.[6 7] The combination of these two conditions is expected to have a dramatic impact on the burden of healthcare worldwide.[8-11]

Management of AF involves anticoagulation to prevent strokes, selecting appropriate patients for restoration of sinus rhythm and almost universal need for control of heart rate. In contrast to other management strategies, the choice of rate control therapy has a very low-quality evidence-base (**Figure 1**).[12] Guidelines from the National Institute for Health and Care Excellence (NICE) and the European Society of Cardiology (ESC) have mandated further research specifically on rate control[1 13], which is also reflected in the level of recommendations from the American Heart Association.[14] The small studies currently available are often uncontrolled or with short follow-up[15-19], providing few insights on the biological effects of treatment or the mechanisms underpinning the response to therapy. With no evidence for any impact of rate control on mortality[20 21], and limited data for any difference in quality of life or functional outcomes, the choice of rate control agent is currently informed by expert consensus and physician experience.

In this paper, we review the current evidence-base for rate control in AF and the rationale for a new randomised controlled trial (RCT). The <u>RA</u>te control <u>T</u>herapy <u>E</u>valuation in permanent <u>A</u>trial <u>F</u>ibrillation (RATE-AF) trial will compare initial therapy with beta-blockers versus digoxin in older patients with symptomatic permanent AF, assessing quality of life, functional

capacity, left-ventricular ejection fraction (LVEF), diastolic function and biomarkers of treatment response.

Rationale for a new trial of rate control in AF

Why not choose a rhythm control strategy?

A number of RCTs have assessed the addition of rhythm control strategies to control of heart rate in AF patients, most often with anti-arrhythmic drugs (AAD) and direct current cardioversion. Neither of the two largest trials (AFFIRM or RACE) found any difference in clinical outcomes comparing these approaches.[22 23] Meta-analyses and other smaller trials have confirmed that rhythm control is not superior to regulation of heart rate alone,[24-26] including heart failure patients with both impaired and preserved ejection fraction.[27 28] These studies have analysed heterogeneous populations, including both paroxysmal and permanent AF that may differ with regards to mechanism, prognosis and the response to treatment.[15] However there is also evidence that a rhythm control strategy may increase hospital admissions. A meta-analysis of major published trials is presented in **Figure 2**, highlighting a 17% increase in the risk of hospitalisation in the rhythm control group (after exclusion of hospital visits related to cardioversion). Although limited by patient crossover and the association between AAD and adverse events,[29] the results highlight the importance of trials comparing different rate control options and associated healthcare costs.

Although AF ablation is becoming increasingly popular it remains a highly invasive method to restore sinus rhythm.[30 31] Current guidelines recommend ablation to improve AF-related symptoms in patients with paroxysmal AF, or as a treatment option in symptomatic persistent AF that is refractory to other therapy.[1 14] Long-term outcome studies are awaited and need to be balanced against procedural complications and AF recurrence. Even in patients

For peer review only

receiving intensive rhythm control therapy, rate control is often necessary to reduce symptoms during AF paroxysms. Further, 40-50% of AF patients are deemed as unsuitable for rhythm control (permanent AF),[5 32] and are maintained on rate control therapy to reduce potential symptoms and avoid tachycardia that may worsen ventricular function.[6] Patients with permanent AF have a higher residual risk of cardiovascular death, stroke or systemic embolism, despite anticoagulation.[33]

What is the optimal heart rate target in AF?

There is clinical uncertainty about how to control heart rate and the intensity of rate-reduction. In the RACE II trial of 614 randomised patients with permanent AF, there were no benefits of strict (<80 bpm at rest) compared to lenient rate control (resting heart rate <110 bpm) over 3 years of follow-up.[34] Although interpretation was limited by the narrow difference in heart rate between groups, lenient rate control was found to be non-inferior with an adjusted hazard ratio of 0.80 (90% CI 0.55-1.17) for a wide composite of adverse clinical outcomes (12.9%, compared to 14.9% in the strict control arm). In addition, there were no differences in symptoms or NYHA class,[34 35] and patients achieving strict rate control required more clinic visits.[36] These findings are consistent with other trials,[37-39] registries,[32] and even observational cohorts in patients with concomitant heart failure,[40] suggesting that intensity of heart rate control is not the key determinant of outcomes in AF.

Do outcomes vary with different rate control therapies?

Medical therapy to achieve rate control in AF can be achieved with beta-blockers, digoxin and non-dihydropiridine calcium channel blockers (CCB; diltiazem or verapamil).[1] Only a limited evidence-base is available to assist clinicians in choosing first-line and subsequent therapy, resulting in wide variations in clinical practice,[41-43] and frequent use of combination therapy. Guidelines suggest the choice of medication should be individualised, dependent on the

For peer review only

presence of ongoing symptoms.[1 14] However, these recommendations are based on low quality trials and observational data, often with small numbers of participants and follow-up over a few weeks.[16] There are no RCTs comparing long-term rate control options in AF. Demonstrating any reduction in hard clinical outcomes with rate control has proved elusive. In patients with heart failure, reduced ejection fraction and concomitant AF, an individual patient level meta-analysis of double-blind RCT data has suggested that beta-blockers do not reduce all-cause mortality or hospital admissions compared to placebo[20], in contrast to the substantial benefit seen in sinus rhythm.[44] Similarly, the use of digoxin was not associated with any increase, or reduction, in mortality in a comprehensive systematic review.[21] This finding deviates from prior observational analyses which are confounded by the fact that sicker patients tend to receive digoxin more often, which can only be addressed within a randomised trial. Although digoxin is known to reduce hospital admissions in patients with heart failure and reduced ejection fraction in sinus rhythm[45], the impact in patients with AF is unknown.

If rate control has limited effect on mortality, what about evidence for a differential effect on other outcomes, such as functional capacity, cardiac function or quality of life? Beta-blockers are the most commonly-used rate control agents and although they have a greater impact than digoxin on heart rate during exertion, there is no evidence that this results in better exercise capacity.[17 18 46-48] Beta-blockers were not associated with any improvement in arrhythmia-related symptoms in a small RCT of 60 low-risk patients with permanent AF, compared to diltiazem and verapamil which reduced the frequency of symptoms.[49] Those in the beta-blocker group had a reduction in exercise capacity and increase in B-type natriuretic peptide (BNP) compared to those treated with CCB.[50] Analysis of smaller trials comparing beta-blockers with CCB are inconsistent.[17] Compared to verapamil or diltiazem, digoxin has less effect on heart rate but there is no consistent evidence for any difference in functional outcomes.[17 18 46 48 51] Importantly, diltiazem and verapamil are usually avoided in patients with

For peer review only

reduced ejection fraction due to the risk of adverse outcomes,[52-56] leaving only beta-blockers or digoxin as suitable therapy. Only a single RCT has been published comparing beta-blockers with digoxin in patients with AF and heart failure (mean LVEF 24%, n=47).[57] Although there was a marginally-significant improvement in LVEF with combined carvedilol/digoxin versus placebo/digoxin, blinded withdrawal of digoxin then led to a deterioration in LVEF, accompanied by an increase in BNP. The direct effects of digoxin on LVEF and diastolic function have only been studied in patients with sinus rhythm where digoxin increased LVEF by 3-11% and improved diastolic filling.[58-60] Magnesium has been shown to complement digoxin therapy to achieve lower ventricular rates in AF [61], but is not in common use due to the availability of beta-blockers and CCB which are more potent agents for acute heart rate control.[1] Although data on patient-reported quality of life is limited,[62 63] rate control has been associated with improved quality of life in trials assessing rate versus rhythm control.[64-66] The mechanism by which rate control therapy mediates an increase in physical functioning and quality of life is unknown but conceivably due to improvements in LVEF and/or diastolic function.

In summary, rate control is an important part of treatment in all AF patients but the evidence-base is poor, particularly in those with permanent AF who form the majority of patients in clinical practice. Rate control in AF is also subject to considerable, and poorly characterised individual variability in response, with limited information about the effects of therapy on cardiac function, quality of life and functional capacity.

The RATE-AF trial

The RATE-AF trial is the first head-to-head randomised assessment of beta-blockers versus digoxin as the initial rate control agent in patients with AF. The trial has a prospective,

For peer review only

randomised, open-label, investigator-blinded endpoint (PROBE) design, and is planned as an inclusive study that reflects and will have an important impact on clinical practice (see Information for Patients in **Table 1**). The primary outcome is patient-reported quality of life using the SF-36 physical component summary score at 6 months' post-randomisation. The major secondary outcomes are change in LVEF and diastolic function on echocardiography, functional capacity, global and AF-specific quality of life, and cardiovascular biomarkers (see **Table 2**). A key objective of the trial is to improve the methods used for measuring quality of life in AF patients, as well as optimising the validity, reproducibility and acquisition of echocardiographic left-ventricular function. The RATE-AF trial will also act as a feasibility study to plan a future, event-driven clinical trial exploring the impact of different rate control strategies on cardiovascular events and unplanned hospital admissions. The study is sponsored by the University of Birmingham and funded by the National Institute for Health Research (NIHR), as part of a Career Development Fellowship awarded to the Chief Investigator (DK).

Methods

Patients

Inclusion criteria are patients aged 60 years or older with breathlessness (New York Heart Association Class II or more) and permanent AF, characterised as a physician decision for rate control with no plans for cardioversion, AAD or ablation therapy. Only limited exclusion criteria apply (**Figure 3**), reflecting any clear requirements or contraindications for either beta-blockers or digoxin. As neither agent impacts on mortality in patients with heart failure[20 21], reduced LVEF is not an exclusion criterion. All patients are expected to be anticoagulated if appropriate, according to their clinical risk of stroke and thromboembolism.

For peer review only - http://bmjopen.bmj.com/site/about/guidelines.xhtml

Study procedures and outcomes

One hundred and sixty eligible patients in need of rate control will be invited to participate in the study from primary and secondary care across two major NHS Trusts in Birmingham, UK. The RATE-AF trial is managed by the Birmingham Clinical Trials Unit (BCTU; University of Birmingham) and situated within the Birmingham NIHR/Wellcome Trust Clinical Research Facility.

Following written informed consent, participants will be randomised in a 1:1 ratio to either bisoprolol or digoxin therapy. Randomisation will be provided by a computer-generated minimisation algorithm to ensure balance between the treatment arms for baseline European Heart Rhythm Association (EHRA) class and gender. Allocation will be concealed until the patient has been recruited and consented, thereafter the trial will be open-label.

Baseline assessment procedures will include patient-reported quality of life questionnaires (**Table 3**), 6-minute walk distance, echocardiography and biomarker assessment. Participants will then receive study medication (bisoprolol 1.25-15 mg once daily or digoxin 62.5-250 μg once daily), with scheduled uptitration visits to attain a heart rate at rest of ≤100 bpm. This heart rate is in line with international recommendations[1] and was chosen pragmatically to reflect the opinion of many cardiologists that tachycardia can lead to, or worsen, systolic and diastolic dysfunction. Ambulatory 24-hour ECG monitoring will be performed at the end of uptitration (unblinded). Investigator-blinded endpoints will be assessed at the interim (6 month) and final (12 month) visit, which include patient-reported quality of life, echocardiographic parameters of systolic and diastolic left-ventricular function and biomarker assessment (**see Figure 3**).

Exploratory work and clinical practice improvement

During the trial, qualitative research using focus groups and structured interviews will assess whether the quality of life questionnaires adequately and acceptably assess changes in

For peer review only

symptom burden in a sample of patients from each treatment arm. We will also compare and contrast the generic and AF-specific questionnaires. The aim of this work is to improve the methods used for measuring patient-reported outcomes in AF, and to address some of the limitations we have identified in published validation studies.[67]

Optimal acquisition of echocardiography in patients with AF will be determined by reproducibility studies, comparing repeated measures of systolic/diastolic function according to cardiac cycle length. The RATE-AF trial will address the evidence-gaps we have identified in a systematic review of echocardiography in patients with AF[68], and explore the diagnostic difficulty of categorising heart failure in the context of AF (particularly with preserved ejection fraction, where symptom classification is confounded and BNP levels are consistently raised due to AF[7]).

Blood samples from participants will analysed for the cellular effects of rate control (intracellular sodium, calcium and cardiotonic steroids) using integrated fluorescence/contractility photometry in human cardiomyocytes. This work will give mechanistic insight into the cellular response to beta-blockers and digoxin, and identify novel markers of treatment effect. Serum will also be stored for the development of new blood-based and genetic biomarkers that aid in personalisation of rate control therapy.

Statistical considerations

The null hypothesis is of no difference in the physical functioning domain of the SF-36 quality of life questionnaire when comparing a strategy of digoxin versus beta-blocker therapy for initial rate control in older patients with permanent AF. The alternative hypothesis is superiority of one over the other therapy as an initial strategy of care. Randomising 144 patients we can assume an 85% power to detect an effect size of half a standard deviation in a continuous outcome measure of quality of life (two-sided alpha of 0.05). Assuming that 10% of patients will be lost to follow-up, 160 patients are needed. There is some evidence from

For peer review only

existing research to support the notion that the treatment effect could be this large. This includes a 17% improvement in SF-36 role-physical score in the rate control arm of the RACE study,[65] a 22% improvement in a proprietary symptom-checklist with CCB (compared to 8% change in those assigned beta-blockers),[19] and 17% improvement with rate control using SF-36 in the PIAF trial.[66] The RATE-AF trial will also us to explore surrogates for clinical outcomes, such as LVEF using echocardiography and B-type natriuretic peptide, and provide estimates for a future definitive trial of rate control in AF, including reliable information on recruitment rates, study drug titration, cross-over, retention and healthcare costs.

Trial oversight, management and registration

RATE-AF will be conducted in accordance with guidelines for Good Clinical Practice (GCP) and the Declaration of Helsinki, and has regulatory approval from the Medicines and Healthcare products Regulatory Agency (MHRA).

Oversight will be provided by a Trial Steering Committee, comprising an independent Data Monitoring Committee and members of the RATE-AF Trial Management Group. This includes representatives of the patient and public involvement panel, involved in both the design and management of the trial. A Clinical Events Committee will be formed to adjudicate on adverse events.

The RATE-AF trial is registered at clinicaltrials.gov (NCT02391337), ISRCTN (95259705) and EudraCT (2015-005043-13). Further information can be obtained from the trial website, http://www.birmingham.ac.uk/rate-af, and the trial protocol (see **Appendix**). The protocol was developed in accordance with the Standard Protocol Items for Randomized Trials [SPIRIT] statement[69], and the latest guidance from the International Society for Quality of Life Research (ISOQOL) Best Practice taskforce.[70-72]

Ethics and Dissemination

For peer review only

The trial has ethical approval from the East Midlands - Derby Research Ethics Committee (16/EM/0178) and approval from the National Health Service (NHS) Health Research Authority (IRAS project ID: 191437).

The research findings will be submitted for publication to peer-reviewed journals after review by the oversight committees and the Patient Involvement Panel, and presented to relevant national and international meetings. Trial participants will be sent a lay summary of the final results of the trial, written by the Patient Involvement Panel.

For peer review only

Conclusion

Defining appropriate rate control therapy is vital, particularly in the rapidly growing number of older patients with permanent AF where current evidence is extremely limited. Rate control is an integral part of management in almost all AF patients but hardly any controlled trial evidence exists to guide the choice of agents. This is unacceptable in light of the potential benefits and possible adverse effects of treatment. In addition, the complete lack of data on the impact of medical therapy on symptom burden and heart function necessitate a programme of reproducibility and validity of both patient-reported quality of life and cardiac imaging in AF. The RATE-AF trial will answer key clinical questions about how to initiate therapy in order to improve patient well-being, stratified by relevant patient characteristics such as baseline symptoms, systolic and diastolic cardiac function, and biomarkers of treatment effect.

For peer review only

Acknowledgements

We would like to acknowledge other members of the wider RATE-AF team, including Karina Bunting, Dannie Fobian, Margaret Grant, Hannah Lack, Susan Jowett, Jonathan Mathers, and Davor Pavlovic (University of Birmingham). We are indebted to the independent members of the trial oversight committees, as well as the Patient and Public Involvement (PPI) Team.

Competing interests

None of the authors report a conflict of interest. All authors have completed the ICMJE uniform disclosure form (www.icmje.org/coi_disclosure.pdf) and declare:

DK reports grants from Menarini, during the conduct of the study; non-financial support from Daiichi Sankyo and personal fees from AtriCure, outside the submitted work. MC reports grants from the National Institute of Health Research, during the conduct of the study; and personal fees from Astella Pharma and Ferring Pharma, outside the submitted work. PK reports consulting fees and honoraria from Bayer Healthcare, Boehringer Ingelheim, Bristol-Myers Squibb, Daiichi-Sankyo, Medtronic, Pfizer and Servier, all outside the submitted work; research grants from Bristol-Myers Squibb, Pfizer, Cardiovascular Therapeutics, Daiichi Sankyo, Sanofi, St. Jude Medical, German Federal Ministry for Education and Research (BMBF), Fondation Leducq, German Research Foundation (DFG), European Union, British Heart Foundation and Medical Research Council UK, all outside the submitted work; and is listed on two patent applications on AF therapy and markers for AF, both outside the submitted work. GYHL has served as a consultant for Bayer, Astellas, Merck, AstraZeneca, Sanofi, BMS/Pfizer, Biotronik, Portola and Boehringer Ingelheim, and has been on the speaker's bureau for Bayer, BMS/Pfizer, Boehringer Ingelheim, and Sanofi-Aventis. RPS is the President of the British Society of Echocardiography. JJD, MG, MS. JNT, SM, GS report no competing interests.

For peer review only

Authors' contributions

The manuscript was drafted by DK who is the Chief Investigator for the RATE-AF trial. MG and GYHL are Principal Investigators. MC, PK, RPS and JNT are members of the Trial Management Group. JJD, SM and GS are representatives from the Clinical Trials Unit. MS is the Lead for the Patient Involvement Panel, and a member of the Steering Committee. All authors contributed to the writing of the RATE-AF protocol or patient information, and edited this manuscript for intellectual content.

Funding

DK and the RATE-AF trial are supported by the National Institute of Health Research (NIHR) as part of a Career Development Fellowship (CDF-2015-08-074). The opinions expressed in this paper are those of the authors and do not represent the NIHR or the UK Department of Health.

Data Sharing Statement

No additional data available at this time.

For peer review only

References

1. Kirchhof P, Benussi S, Kotecha D, et al. 2016 ESC Guidelines for the management of atrial fibrillation developed in collaboration with EACTS. *Eur Heart J* 2016;37:2893-962.

2. Chugh SS, Havmoeller R, Narayanan K, et al. Worldwide epidemiology of atrial fibrillation: a Global Burden of Disease 2010 Study. *Circulation* 2014;129:837-47.

3. Krijthe BP, Kunst A, Benjamin EJ, et al. Projections on the number of individuals with atrial fibrillation in the European Union, from 2000 to 2060. *Eur Heart J* 2013;34:2746-51.

4. Lane DA, Skøjth F, Larsen TB, Lip GYH, Kotecha D. Temporal trends in atrial fibrillation incidence, comorbidity and mortality: comprehensive linked data from primary care. *J Am Heart Assoc* 2017: in press.

5. Chiang CE, Naditch-Brule L, Murin J, et al. Distribution and risk profile of paroxysmal, persistent, and permanent atrial fibrillation in routine clinical practice: insight from the real-life global survey evaluating patients with atrial fibrillation international registry. *Circ Arrhythm Electrophysiol* 2012;5:632-9.

6. Kotecha D, Piccini JP. Atrial fibrillation in heart failure: what should we do? *Eur Heart J* 2015;36:3250-7.

7. Kotecha D, Lam CS, Van Veldhuisen DJ, Van Gelder IC, Voors AA, Rienstra M. Heart Failure With Preserved Ejection Fraction and Atrial Fibrillation: Vicious Twins. *J Am Coll Cardiol* 2016;68:2217-28.

8. Kotecha D, Banerjee A, Lip GY. Increased stroke risk in atrial fibrillation patients with heart failure: does ejection fraction matter? *Stroke* 2015;46:608-9.

9. Christiansen CB, Olesen JB, Gislason G, Lock-Hansen M, Torp-Pedersen C. Cardiovascular and non-cardiovascular hospital admissions associated with atrial fibrillation: a Danish nationwide, retrospective cohort study. *BMJ Open* 2013;3.

10. Ambrosy AP, Fonarow GC, Butler J, et al. The global health and economic burden of hospitalizations for heart failure: lessons learned from hospitalized heart failure registries. *J Am Coll Cardiol* 2014;63:1123-33.

11. Kotecha D, Chudasama R, Lane DA, Kirchhof P, Lip GY. Atrial fibrillation and heart

For peer review only

failure due to reduced versus preserved ejection fraction: A systematic review and meta-analysis of death and adverse outcomes. *Int J Cardiol* 2016;203:660-6.

12. Kirchhof P, Breithardt G, Bax J, et al. A roadmap to improve the quality of atrial fibrillation management: proceedings from the fifth Atrial Fibrillation Network/European Heart Rhythm Association consensus conference. *Europace* 2016;18:37-50.

13. National Institute for Health and Care Excellence. Atrial fibrillation: the management of atrial fibrillation. *NICE clinical guideline 180* 2014; Accessed 15/09/2016; http://www.nice.org.uk/guidance/cg180/.

14. January CT, Wann LS, Alpert JS, et al. 2014 AHA/ACC/HRS guideline for the management of patients with atrial fibrillation: executive summary: a report of the American College of Cardiology/American Heart Association Task Force on practice guidelines and the Heart Rhythm Society. *Circulation* 2014;130:2071-104.

15. Kotecha D, Kirchhof P. Rate and rhythm control have comparable effects on mortality and stroke in atrial fibrillation but better data are needed. *Evid Based Med* 2014;19:222-3.

16. Segal JB, McNamara RL, Miller MR, et al. The evidence regarding the drugs used for ventricular rate control. J Fam Practice, 2000:47-59.

17. Nikolaidou T, Channer KS. Chronic atrial fibrillation: a systematic review of medical heart rate control management. *Postgrad Med J* 2009;85:303-12.

18. Farshi R, Kistner D, Sarma JSM, Longmate JA, Singh BN. Ventricular rate control in chronic atrial fibrillation during daily activity and programmed exercise: a crossover open-label study of five drug regimens. *J Am Coll Cardiol* 1999;33:304-10.

19. Ulimoen SR, Enger S, Carlson J, et al. Comparison of four single-drug regimens on ventricular rate and arrhythmia-related symptoms in patients with permanent atrial fibrillation. *Am J Cardiol* 2013;111:225-30.

20. Kotecha D, Holmes J, Krum H, et al. Efficacy of beta blockers in patients with heart failure plus atrial fibrillation: an individual-patient data meta-analysis. *Lancet* 2014;384:2235-43.

21. Ziff OJ, Lane DA, Samra M, et al. Safety and efficacy of digoxin: systematic review and

For peer review only

meta-analysis of observational and controlled trial data. *BMJ* 2015;351:h4451.

22. Wyse DG, Waldo AL, DiMarco JP, et al. A comparison of rate control and rhythm control in patients with atrial fibrillation. *N Engl J Med* 2002;347:1825-33.

23. Van Gelder IC, Hagens VE, Bosker HA, et al. A Comparison of Rate Control and Rhythm Control in Patients with Recurrent Persistent Atrial Fibrillation. *N Engl J Med* 2002;347:1834-40.

24. de Denus S, Sanoski CA, Carlsson J, Opolski G, Spinler SA. Rate vs rhythm control in patients with atrial fibrillation: a meta-analysis. *Arch Intern Med* 2005;165:258-62.

25. Chatterjee S, Sardar P, Lichstein E, Mukherjee D, Aikat S. Pharmacologic rate versus rhythm-control strategies in atrial fibrillation: an updated comprehensive review and meta-analysis. *PACE* 2013;36:122-33.

26. Al-Khatib SM, Allen LaPointe NM, Chatterjee R, et al. Rate- and rhythm-control therapies in patients with atrial fibrillation: a systematic review. *Ann Intern Med* 2014;160:760-73.

27. Roy D, Talajic M, Nattel S, et al. Rhythm Control versus Rate Control for Atrial Fibrillation and Heart Failure. *N Engl J Med* 2008;358:2667-77.

28. Kong MH, Shaw LK, O'Connor C, Califf RM, Blazing MA, Al-Khatib SM. Is rhythm-control superior to rate-control in patients with atrial fibrillation and diastolic heart failure? *Ann Noninvasive Electrocardiol* 2010;15:209-17.

29. Corley SD, Epstein AE, DiMarco JP, et al. Relationships between sinus rhythm, treatment, and survival in the Atrial Fibrillation Follow-Up Investigation of Rhythm Management (AFFIRM) Study. *Circulation* 2004;109:1509-13.

30. Wazni O, Wilkoff B, Saliba W. Catheter Ablation for Atrial Fibrillation. *N Engl J Med* 2011;365:2296-304.

31. Jones DG, Haldar SK, Hussain W, et al. A randomized trial to assess catheter ablation versus rate control in the management of persistent atrial fibrillation in heart failure. *J Am Coll Cardiol* 2013;61:1894-903.

32. Kirchhof P, Ammentorp B, Darius H, et al. Management of atrial fibrillation in seven European countries after the publication of the 2010 ESC Guidelines on atrial

For peer review only

fibrillation: primary results of the PREvention oF thromboemolic events--European Registry in Atrial Fibrillation (PREFER in AF). *Europace* 2014;16:6-14.

33. Senoo K, Lip GY, Lane DA, Buller HR, Kotecha D. Residual risk of stroke and death in anticoagulated patients according to the type of atrial fibrillation: AMADEUS Trial. *Stroke* 2015;46:2523-8.

34. Van Gelder IC, Groenveld HF, Crijns HJGM, et al. Lenient versus Strict Rate Control in Patients with Atrial Fibrillation. *N Engl J Med* 2010;362:1363-73.

35. Groenveld HF, Crijns HJGM, Van den Berg MP, et al. The Effect of Rate Control on Quality of Life in Patients With Permanent Atrial Fibrillation: Data From the RACE II (Rate Control Efficacy in Permanent Atrial Fibrillation II) Study. *J Am Coll Cardiol* 2011;58:1795-803.

36. Groenveld HF, Tijssen JG, Crijns HJ, et al. Rate control efficacy in permanent atrial fibrillation: successful and failed strict rate control against a background of lenient rate control: data from RACE II (Rate Control Efficacy in Permanent Atrial Fibrillation). *J Am Coll Cardiol* 2013;61:741-8.

37. Van Gelder IC, Wyse DG, Chandler ML, et al. Does intensity of rate-control influence outcome in atrial fibrillation? An analysis of pooled data from the RACE and AFFIRM studies. *Europace* 2006;8:935-42.

38. Cooper HA, Bloomfield DA, Bush DE, et al. Relation between achieved heart rate and outcomes in patients with atrial fibrillation (from the Atrial Fibrillation Follow-up Investigation of Rhythm Management [AFFIRM] Study). *Am J Cardiol* 2004;93:1247-53.

39. Groenveld HF, Crijns HJ, Rienstra M, et al. Does intensity of rate control influence outcome in persistent atrial fibrillation? Data of the RACE study. *Am Heart J* 2009;158:785-91.

40. Cullington D, Goode KM, Zhang J, Cleland JG, Clark AL. Is heart rate important for patients with heart failure in atrial fibrillation? *JACC Heart Fail* 2014;2:213-20.

41. Steg PG, Alam S, Chiang C-E, et al. Symptoms, functional status and quality of life in patients with controlled and uncontrolled atrial fibrillation: data from the RealiseAF cross-sectional international registry. *Heart* 2012;98:195-201.

For peer review only

42. Nabauer M, Gerth A, Limbourg T, et al. The Registry of the German Competence NETwork on Atrial Fibrillation: patient characteristics and initial management. *Europace* 2009;11:423-34.

43. Lip GY, Laroche C, Dan GA, et al. A prospective survey in European Society of Cardiology member countries of atrial fibrillation management: baseline results of EURObservational Research Programme Atrial Fibrillation (EORP-AF) Pilot General Registry. *Europace* 2014;16:308-19.

44. Kotecha D, Manzano L, Krum H, et al. Effect of age and sex on efficacy and tolerability of beta blockers in patients with heart failure with reduced ejection fraction: individual patient data meta-analysis. *BMJ* 2016;353:i1855.

45. The Digitalis Investigation Group. The effect of digoxin on mortality and morbidity in patients with heart failure. *N Engl J Med* 1997;336:525-33.

46. Koh KK, Kwon KS, Park HB, et al. Efficacy and safety of digoxin alone and in combination with low-dose diltiazem or betaxolol to control ventricular rate in chronic atrial fibrillation. *Am J Cardiol* 1995;75:88-90.

47. Lewis RV, McMurray J, McDevitt DG. Effects of atenolol, verapamil, and xamoterol on heart rate and exercise tolerance in digitalised patients with chronic atrial fibrillation. *J Cardiovasc Pharmacol* 1989;13:1-6.

48. Tsuneda T, Yamashita T, Fukunami M, et al. Rate control and quality of life in patients with permanent atrial fibrillation: the Quality of Life and Atrial Fibrillation (QOLAF) Study. *Circ J* 2006;70:965-70.

49. Ulimoen SR, Enger S, Carlson J, et al. Comparison of four single-drug regimens on ventricular rate and arrhythmia-related symptoms in patients with permanent atrial fibrillation. *Am J Cardiol* 2013;111:225-30.

50. Ulimoen SR, Enger S, Pripp AH, et al. Calcium channel blockers improve exercise capacity and reduce N-terminal Pro-B-type natriuretic peptide levels compared with beta-blockers in patients with permanent atrial fibrillation. *Eur Heart J* 2014;35:517-24.

51. Lewis RV, Irvine N, McDevitt DG. Relationships between heart rate, exercise tolerance and cardiac output in atrial fibrillation: the effects of treatment with digoxin, verapamil

For peer review only

and diltiazem. *Eur Heart J* 1988;9:777-81.

52. McMurray JJ, Adamopoulos S, Anker SD, et al. ESC Guidelines for the diagnosis and treatment of acute and chronic heart failure 2012: The Task Force for the Diagnosis and Treatment of Acute and Chronic Heart Failure 2012 of the European Society of Cardiology. Developed in collaboration with the Heart Failure Association (HFA) of the ESC. *Eur Heart J* 2012;33:1787-847.

53. Elkayam U. Calcium channel blockers in heart failure. *Cardiology* 1998;89 Suppl 1:38-46.

54. The effect of diltiazem on mortality and reinfarction after myocardial infarction. The Multicenter Diltiazem Postinfarction Trial Research Group. *N Engl J Med* 1988;319:385-92.

55. Goldstein RE, Boccuzzi SJ, Cruess D, Nattel S. Diltiazem increases late-onset congestive heart failure in postinfarction patients with early reduction in ejection fraction. *Circulation* 1991;83:52-60.

56. The Danish Study Group on Verapamil in Myocardial Infarction. Secondary prevention with verapamil after myocardial infarction. *Am J Cardiol* 1990;66:33-40.

57. Khand AU, Rankin AC, Martin W, Taylor J, Gemmell I, Cleland JG. Carvedilol alone or in combination with digoxin for the management of atrial fibrillation in patients with heart failure? *J Am Coll Cardiol* 2003;42:1944-51.

58. Partanen J, Heikkila J, Pellinen T, Nieminen MS. Effect of digoxin on the heart in normal subjects: influence of isometric exercise and autonomic blockade: a noninvasive study. *Br J Clin Pharmacol* 1988;25:331-40.

59. Dernellis JM, Panaretou MP. Effects of digoxin on left atrial function in heart failure. *Heart* 2003;89:1308-15.

60. Giunta A, Maione S, Arnese MR, et al. Effects of intravenous digoxin on pulmonary venous and transmitral flows in patients with chronic heart failure of different degrees. *Clin Cardiol* 1995;18:27-33.

61. Kotecha D. Magnesium for Atrial Fibrillation, Myth or Magic? *Circ Arrhythm Electrophysiol* 2016;9.

62. Thrall G, Lane D, Carroll D, Lip GYH. Quality of Life in Patients with Atrial Fibrillation:

For peer review only

A Systematic Review. *Am J Med* 2006;119:448.e1-19.

63. Rienstra M, Lubitz SA, Mahida S, et al. Symptoms and Functional Status of Patients With Atrial Fibrillation: State of the Art and Future Research Opportunities. *Circulation* 2012;125:2933-43.

64. Pepine CJ. Effects of pharmacologic therapy on health-related quality of life in elderly patients with atrial fibrillation: a systematic review of randomized and nonrandomized trials. *Clin Med Insights Cardiol* 2013;7:1-20.

65. Hagens VE, Ranchor AV, Van Sonderen E, et al. Effect of rate or rhythm control on quality of life in persistent atrial fibrillation: Results from the Rate Control Versus Electrical Cardioversion (RACE) study. *J Am Coll Cardiol* 2004;43:241-47.

66. Grönefeld GC, Lilienthal J, Kuck K-H, Hohnloser SH. Impact of rate versus rhythm control on quality of life in patients with persistent atrial fibrillation: Results from a prospective randomized study. *Eur Heart J* 2003;24:1430-36.

67. Kotecha D, Ahmed A, Calvert M, Lencioni M, Terwee CB, Lane DA. Patient-Reported Outcomes for Quality of Life Assessment in Atrial Fibrillation: A Systematic Review of Measurement Properties. *PLoS ONE* 2016;11:e0165790.

68. Kotecha D, Mohamed M, Shantsila E, Popescu BA, Steeds RP. Is echocardiography valid and reproducible in patients with atrial fibrillation? A systematic review. *Europace* 2017: *in press.*

69. Chan AW, Tetzlaff JM, Gotzsche PC, et al. SPIRIT 2013 explanation and elaboration: guidance for protocols of clinical trials. *BMJ* 2013;346:e7586.

70. Calvert M, Kyte D, von Hildebrand M, King M, Moher D. Putting patients at the heart of health-care research. *Lancet* 2015;385:1073-74.

71. Calvert M, Kyte D, Duffy H, et al. Patient-reported outcome (PRO) assessment in clinical trials: a systematic review of guidance for trial protocol writers. *PLoS One* 2014;9:e110216.

72. Kyte D, Duffy H, Fletcher B, et al. Systematic evaluation of the patient-reported outcome (PRO) content of clinical trial protocols. *PLoS One* 2014;9:e110229.

73. Ware JE, Gandek B. Overview of the SF-36 Health Survey and the International Quality of

For peer review only

Life Assessment (IQOLA) Project. *J Clin Epidemiol* 1998;51:903-12.

74. Gandek B, Sinclair SJ, Kosinski M, Ware JE, Jr. Psychometric evaluation of the SF-36 health survey in Medicare managed care. *Health Care Financ Rev* 2004;25:5-25.

75. Herdman M, Gudex C, Lloyd A, et al. Development and preliminary testing of the new five-level version of EQ-5D (EQ-5D-5L). *Qual Life Res* 2011;20:1727-36.

76. Devlin NJ, Krabbe PF. The development of new research methods for the valuation of EQ-5D-5L. *Eur J Health Econ* 2013;14 Suppl 1:S1-3.

77. Janssen MF, Pickard AS, Golicki D, et al. Measurement properties of the EQ-5D-5L compared to the EQ-5D-3L across eight patient groups: a multi-country study. *Qual Life Res* 2013;22:1717-27.

78. Spertus J, Dorian P, Bubien R, et al. Development and validation of the Atrial Fibrillation Effect on QualiTy-of-Life (AFEQT) Questionnaire in patients with atrial fibrillation. *Circ Arrhythm Electrophysiol* 2011;4:15-25.

79. Dorian P, Burk C, Mullin CM, et al. Interpreting changes in quality of life in atrial fibrillation: How much change is meaningful? *Am Heart J* 2013;166:381-87.e8.

80. Wynn GJ, Todd DM, Webber M, et al. The European Heart Rhythm Association symptom classification for atrial fibrillation: validation and improvement through a simple modification. *Europace* 2014;16:965-72.

81. Carlsson J, Miketic S, Windeler J, et al. Randomized trial of rate-control versus rhythm-control in persistent atrial fibrillation: the Strategies of Treatment of Atrial Fibrillation (STAF) study. *J Am Coll Cardiol* 2003;41:1690-6.

82. Hohnloser SH, Kuck KH, Lilienthal J. Rhythm or rate control in atrial fibrillation--Pharmacological Intervention in Atrial Fibrillation (PIAF): a randomised trial. *Lancet* 2000;356:1789-94.

83. Opolski G, Torbicki A, Kosior DA, et al. Rate control vs rhythm control in patients with nonvalvular persistent atrial fibrillation: the results of the Polish How to Treat Chronic Atrial Fibrillation (HOT CAFE) Study. *Chest* 2004;126:476-86.

84. Vora A, Karnad D, Goyal V, et al. Control of heart rate versus rhythm in rheumatic atrial fibrillation: a randomized study. *J Cardiovasc Pharmacol Ther* 2004;9:65-73.

For peer review only

Table 1: The RATE-AF trial – Information for Patients

About atrial fibrillation
Atrial fibrillation is a common heart condition that leads to an irregular and often rapid heart rate. Atrial fibrillation causes 1 in 4 strokes, and patients have frequent hospital admissions and a higher risk of dying. In addition, atrial fibrillation makes many patients feel unwell, with reduced quality of life.
**What is the purpose of the trial?**
Atrial fibrillation usually requires medication to control heart rate, but we currently don't know which medication is better for patients. The aim of this study is to find out which of two treatments improves quality of life and the function of the heart, digoxin or bisoprolol (a beta-blocker).
**What will happen in the trial?**
The RATE-AF trial is designed to compare two approaches for control of heart rate, based on initial treatment with either digoxin or beta-blockers, medications which are commonly used by doctors. The main objective of the trial is to research the effects of treatment on quality of life in patients with atrial fibrillation. We will also test whether quality of life questionnaires respond to changes in symptoms experienced by patients, how we use ultrasound to look at the function of the heart, and develop new markers in the blood to personalise treatment.
**More information**
RATE-AF trial video: https://www.youtube.com/watch?v=4oxe8AcVo0E or search 'rateaf' in YouTube.<br>Patient information (British Heart Foundation): https://www.bhf.org.uk/heart-health/conditions/atrial-fibrillation.

For peer review only

Table 2: Outcomes and objectives of the RATE-AF trial

**Primary outcome:**
Comparison of two strategies for rate control on patient-reported quality of life, based on initial use of digoxin versus beta-blocker therapy, with a predefined focus on physical well-being using the SF-36 physical component summary at six months.
**Secondary outcomes:**
Patient-reported quality of life at six and twelve months, including SF-36 global and domain-specific scores, EQ-5D-5L summary index and visual analogue scale, and AFEQT overall score.
Echocardiographic left-ventricular function at 12 months, including LVEF and diastolic function (E/e' and composite of diastolic indices).
Functional assessment at 6 and 12 months, including six-minute walking distance and change in EHRA class.
Change in BNP levels at 6 months.
Change in heart rate from baseline and group comparison using 24-hour ambulatory ECG at end of uptitration.
**Feasibility assessment:**
Successful methods for recruitment across primary and secondary care.
Key issues that affect retention of participants, such as convenience, compliance and cross-over.
Drug discontinuation rate and adverse reactions leading to drug discontinuation.
Therapy-induced requirement for additional treatment (e.g. pacemaker implantation).
Population-specific standard deviations and proportions to enable sample size calculation for a future trial.
Assessment of unplanned hospital admissions and cardiovascular outcomes.
**Exploratory objectives:**
Assessment of the validity and reproducibility of echocardiographic measures in patients with AF.
Correlation of baseline measures, including quality of life questionnaires and unblinded baseline investigations such as quality of life, BNP, LVEF, E/e', EHRA class, intracellular biomarkers and heart rate.
Impact of therapy on intracellular sodium and calcium concentration and cardiotonic steroid levels as biomarkers of cellular response.
Impact of combination therapy on outcomes.
Change in cognitive function at twelve months.
Qualitative research of patient-reported quality of life using focus groups to explore patient acceptability, optimal delivery methods and responsiveness.
Correlation of serum digoxin concentration with change in quality of life and intracellular methods.
Cost-consequence economic analysis from an NHS healthcare perspective.

AF, Atrial Fibrillation; AFEQT, Atrial Fibrillation Effect on QualiTy-of-life questionnaire; BNP, B-type natriuretic peptide; ECG, electrocardiogram; EHRA, European Heart Rhythm Association functional class; EQ-5D-5L, EuroQol five dimensions five level questionnaire; LVEF, left ventricular ejection fraction; NHS, National Health Service; SF-36, Short Form (36) Health Survey.

For peer review only

Table 3: Patient-reported quality of life questionnaires used in RATE-AF

Questionnaire	Details	Advantages and disadvantages
SF-36<br>Short Form (36) Health Survey [73]	Generic instrument with 4-week recall period in eights domains (vitality, physical functioning, bodily pain, general health perceptions, physical role functioning, emotional role functioning, social role functioning and mental health).<br>11 subdivided questions, each recorded with a Likert scale.<br>Scoring: Each response is given a numerical value (0 to 100, with 100 representing the best level of functioning possible), which are averaged across each domain.	Extensively validated across a wide variety of conditions and the elderly.[74]<br>Not specific to AF and hence other comorbidities may dominate responses.<br>Requires a license fee.
EQ-5D-5L<br>EuroQol five dimensions five level questionnaire [75 76]	Generic instrument about today's health with a five-answer scale in five domains (mobility, self-care, usual activities, pain/discomfort and anxiety/depression).<br>Scoring: Each question is scored (1 to 5, with 1 representing the best health). The overall profile can be indexed to country specific value sets giving a continuous value.<br>Also includes a visual analogue scale denoting current health perception (0 to 100 scale, with 100 representing the best health the patient can imagine).	Simple questionnaire that is quick to complete and includes a visual scale.<br>Extensive utilisation, particularly for heath economic assessment, with improvement discrimination over prior versions.[77]<br>Not specific to AF and hence other comorbidities may dominate responses.
AFEQT<br><br>Atrial Fibrillation Effect on QualiTy-of-life questionnaire [78]	AF-specific quality of life instrument with 4-week recall period in domains relating to symptoms, daily activities and treatment.<br>20 questions (18 on health-related quality and life and 2 on treatment satisfaction), each recorded with a 7-point Likert scale.<br>Scoring: Responses to the 18 questions are summed and converted to a continuous score (0 to 100, with 100 corresponding to no patient concerns nor disability due to AF). Component domains are scored in a similar way.	Specific to the impact of AF on quality of life.<br>Better than other AF-specific tools in a systematic review of methodological/psychometric assessment.[67]<br>Limited validation as yet in comparison to generic tools[79 80], particularly for clinical responsiveness.<br>License fee may apply.

For peer review only

Figure legends

Figure 1: Evidenced-based summary for management of AF

Summary of evidence for main components of clinical management, highlighting paucity of robust data for key issues regarding rate control therapy. RCT, randomised controlled trial; LV, left-ventricular; NOAC, novel oral anticoagulants.

Figure 2: Hospitalisation in rate versus rhythm control trials

Meta-analysis of hospitalisation in the six largest rate versus rhythm control trials, excluding hospital visits for cardioversion procedures, where applicable. Studies are pooled with a random-effects model. Significant heterogeneity was identified, with an I^2 value of 66.8% (p=0.01). Grey boxes represent the comparative weight of the study.

STAF, Strategies of Treatment of Atrial Fibrillation study (cardioversion/AAD versus rate control in persistent AF)[81]; PIAF, Pharmacological Intervention in Atrial Fibrillation trial (amiodarone/cardioversion versus diltiazem in persistent AF)[82]; HOT CAFE, How to Treat Chronic Atrial Fibrillation study (cardioversion/AAD versus rate control in persistent AF)[83]; AF-CHF, Atrial Fibrillation and Congestive Heart Failure trial (cardioversion/AAD versus rate control in paroxysmal/persistent AF with LVEF ≤35%)[27]; CRAAFT, Control of Rate versus Rhythm in rheumatic Atrial Fibrillation Trial (cardioversion/amiodarone versus diltiazem in persistent AF due to rheumatic heart disease)[84]; AFFIRM, Atrial Fibrillation Follow-up Investigation of Rhythm Management study (AAD/cardioversion versus rate control in paroxysmal/persistent AF).[22]

Figure 3: RATE-AF trial schema

Trial flowchart, including major endpoints and inclusion/exclusion criteria.

For peer review only

Appendix: RATE-AF trial protocol

Please see attached file.

For peer review only

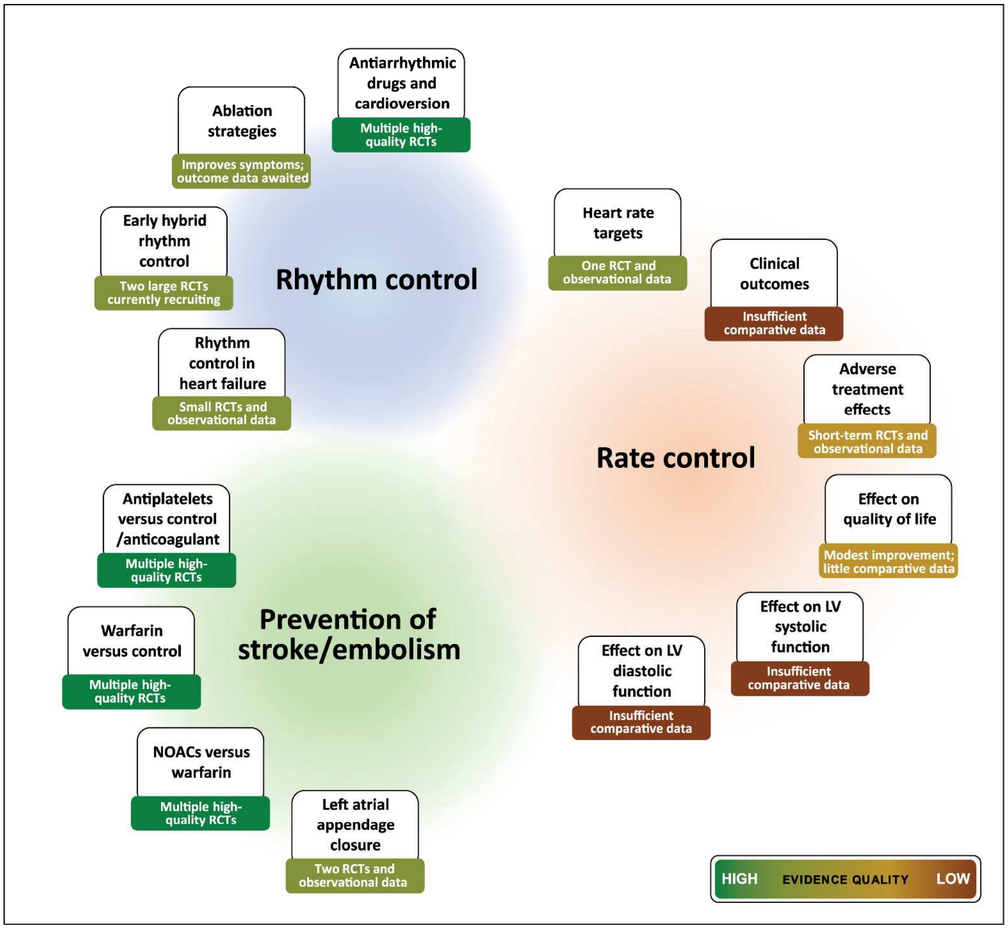

Figure 1: Evidenced-based summary for management of AF
Summary of evidence for main components of clinical management, highlighting paucity of robust data for key issues regarding rate control therapy. RCT, randomised controlled trial; LV, left-ventricular; NOAC, novel oral anticoagulants.

291x267mm (300 x 300 DPI)

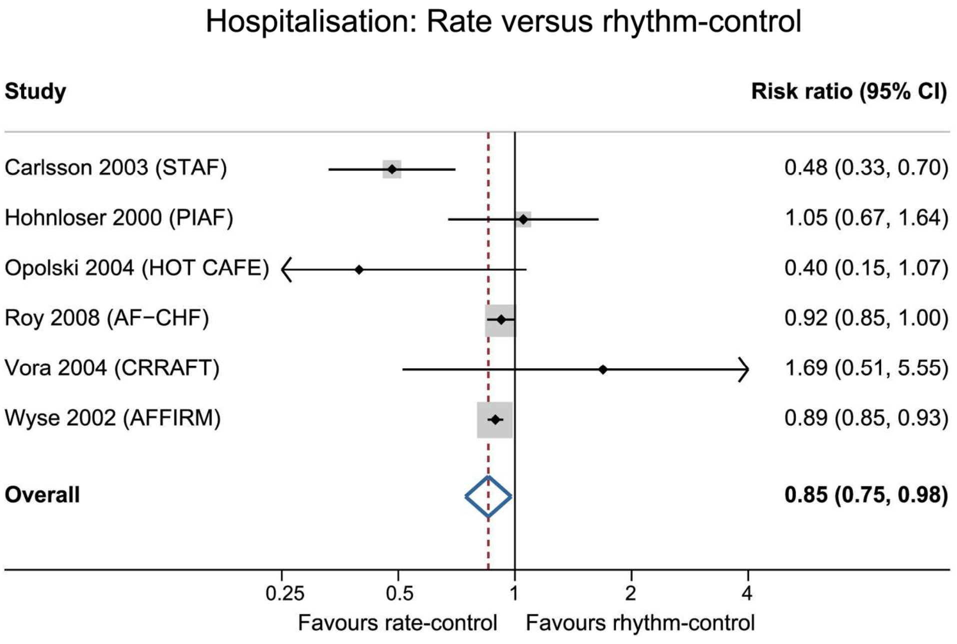

Figure 2: Hospitalisation in rate versus rhythm control trials
Meta-analysis of hospitalisation in the six largest rate versus rhythm control trials, excluding hospital visits for cardioversion procedures, where applicable. Studies are pooled with a random-effects model. Significant heterogeneity was identified, with an I2 value of 66.8% (p=0.01). Grey boxes represent the comparative weight of the study.
STAF, Strategies of Treatment of Atrial Fibrillation study (cardioversion/AAD versus rate control in persistent AF)81; PIAF, Pharmacological Intervention in Atrial Fibrillation trial (amiodarone/cardioversion versus diltiazem in persistent AF)82; HOT CAFE, How to Treat Chronic Atrial Fibrillation study (cardioversion/AAD versus rate control in persistent AF)83; AF-CHF, Atrial Fibrillation and Congestive Heart Failure trial (cardioversion/AAD versus rate control in paroxysmal/persistent AF with LVEF ≤35%)27; CRAAFT, Control of Rate versus Rhythm in rheumatic Atrial Fibrillation Trial (cardioversion/amiodarone versus diltiazem in persistent AF due to rheumatic heart disease)84; AFFIRM, Atrial Fibrillation Follow-up Investigation of Rhythm Management study (AAD/cardioversion versus rate control in paroxysmal/persistent AF).22

157x106mm (300 x 300 DPI)

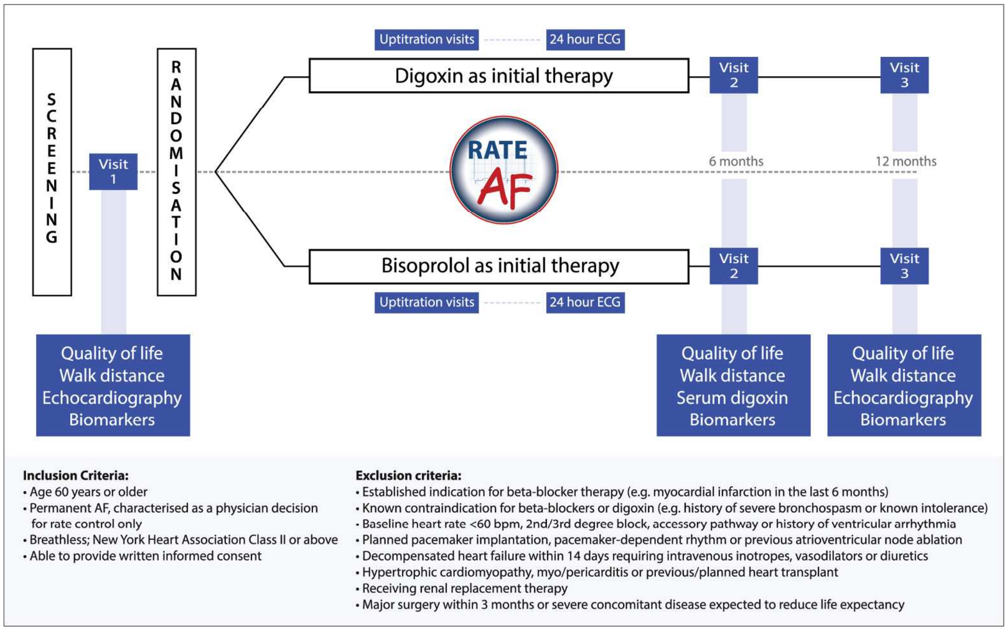

Figure 3: RATE-AF trial schema
Trial flowchart, including major endpoints and inclusion/exclusion criteria.

183x114mm (300 x 300 DPI)

For peer review only - http://bmjopen.bmj.com/site/about/guidelines.xhtml

Evaluating different rate control therapies in permanent atrial fibrillation: A prospective, randomised, open-label, blinded endpoint trial comparing digoxin and beta-blockers as initial rate control therapy

RAte control Therapy Evaluation in Atrial Fibrillation:

RATE-AF

RATE-AF TRIAL PROTOCOL

Version 1.0, 23rd March 2016

**Sponsor:**	**University of Birmingham**
**Chief Investigator:**	**Dr Dipak Kotecha**
**Coordinating Unit:**	**Birmingham Clinical Trials Unit**
**Funder:**	**National Institute for Health Research (NIHR) Career Development Fellowship**

**ISRCTN:**	**TBC**
**EudraCT No.:**	**2015-005043-13**
**REC Ref. No.:**	**TBC**

UNIVERSITY OF BIRMINGHAM

BIRMINGHAM CLINICAL TRIALS UNIT

NHS National Institute for Health Research

For peer review only

For peer review only - http://bmjopen.bmj.com/site/about/guidelines.xhtml

TRIAL COMMITTEES AND CONTACT DETAILS

Trial Management Group	
**Chief Investigator**<br>**Dr Dipak Kotecha**	**NIHR Career Development Fellow & Clinician Scientist**<br><br>Institute of Cardiovascular Sciences, University of Birmingham, The Medical School, Vincent Drive, Birmingham, B15 2TT, UK<br>Email: d.kotecha@bham.ac.uk<br>Telephone: 07974 115676
**Prof Paulus Kirchhof**	**Professor of Cardiovascular Medicine**<br><br>Institute of Cardiovascular Sciences, University of Birmingham, Institute of Biomedical Research, Vincent Drive, Birmingham B15 2TT, UK<br>Email: p.kirchhof@bham.ac.uk<br>Telephone: 0121 414 7042
**Dr Michael Griffith**	**Consultant Electrophysiologist**<br><br>University Hospitals Birmingham NHS Trust, Nuffield House, Queen Elizabeth Hospital, Birmingham, B15 2TH, UK<br>Email: michael.griffith@uhb.nhs.uk<br>Telephone: 0121 371 4038
**Prof Gregory Y H Lip**	**Professor of Cardiovascular Medicine & Director, Haemostasis Thrombosis & Vascular Biology Unit**<br><br>Institute of Cardiovascular Sciences, University of Birmingham, City Hospital, Birmingham, B18 7QH, UK<br>Email g.y.h.lip@bham.ac.uk<br>Telephone: 0121 5075080
**Prof Jonathan Townend**	**Professor of Cardiology**<br><br>University Hospitals Birmingham NHS Trust, Nuffield House, Queen Elizabeth Hospital, Birmingham, B15 2TH, UK<br>Email: john.townend@uhb.nhs.uk<br>Telephone: 0121 371 4623
**Dr Rick Steeds**	**Consultant Cardiologist and Head of Cardiac Imaging**<br><br>University Hospitals Birmingham NHS Trust, Nuffield House, Queen Elizabeth Hospital, Birmingham, B15 2TH, UK<br>Email: rick.steeds@uhb.nhs.uk<br>Telephone: 0121 371 6130
**Prof Melanie Calvert**	**Professor of Outcomes Methodology**<br><br>Institute of Applied Health Research, University of Birmingham, Birmingham, B15 2TT, UK<br>Email: m.calvert@bham.ac.uk<br>Telephone: 0121 414 8595
**Dr Susan Jowett**	**Senior Lecturer, Health Economics**<br><br>Institute of Applied Health Research, University of Birmingham, Birmingham, B15 2TT, UK<br>Email s.jowett@bham.ac.uk<br>Telephone: 0121 414 7898
**Dr Jonathan Mathers**	**Senior Lecturer, Qualitative and Mixed Methods Applied Health Research**<br><br>Institute of Applied Health Research, University of Birmingham, Birmingham, B15 2TT, UK<br>Email j.m.mathers@bham.ac.uk<br>Telephone: 0121 414 6024

For peer review only - http://bmjopen.bmj.com/site/about/guidelines.xhtml

Birmingham Clinical Trials Unit	
**Prof Jon Deeks**	**Professor of Biostatistics and Director, BCTU**<br><br>Birmingham Clinical Trials Unit, University of Birmingham, Birmingham, B15 2TT, UK<br>Email j.deeks@bham.ac.uk<br>Telephone: 0121 414 5328
**Dr Margaret Grant**	**Operations Manager**<br><br>Birmingham Clinical Trials Unit, University of Birmingham, Birmingham, B15 2TT, UK<br>Email m.r.grant@bham.ac.uk<br>Telephone: 0121 415 9106
**Gemma Slinn**	**Senior Trial Coordinator**<br><br>Birmingham Clinical Trials Unit, University of Birmingham, Birmingham, B15 2TT, UK<br>Email g.slinn@bham.ac.uk<br>Telephone: 0121 415 8445
**Samir Mehta**	**Statistician**<br><br>Birmingham Clinical Trials Unit, University of Birmingham, Birmingham, B15 2TT, UK<br>Email s.mehta.1@bham.ac.uk<br>Telephone: 0121 415 9117
**Trial Oversight Committee**	
**Co-Chairs**	
**Dr Kazem Rahimi**	**Associate Professor of Cardiovascular Medicine, University of Oxford**<br>**Deputy Director, The George Institute for Global Health**<br><br>The George Institute for Global Health, University of Oxford, 34 Broad Street, Oxford OX1 3BD, UK<br>Email: kazem.rahimi@georgeinstitute.ox.ac.uk<br>Telephone: 01865 617 201
**Prof. John Camm**	**BHF Professor of Clinical Cardiology**<br><br>St George's University of London, Cranmer Terrace, London SW17 0RE, UK<br>Email: jcamm@sgul.ac.uk<br>Telephone: 0208 725 3414
**Patient Representative**	
**Mary Stanbury**	Lead PPI Representative<br>Email: dms27@btinternet.com
**On behalf of the Trial Management Group**	
**Dr Dipak Kotecha**	
**Prof. Jon Deeks**	For contact details, see Trial Management Group
**Prof. Paulus Kirchhof**	

For peer review only - http://bmjopen.bmj.com/site/about/guidelines.xhtml

RATE-AF Trial Office

For general protocol related queries and supply of trial materials:

Birmingham Clinical Trials Unit (BCTU), Institute of Applied Health Research, College of Medical & Dental Sciences, Public Health Building, University of Birmingham, Edgbaston, Birmingham B15 2TT

Telephone: 0121 415 8445
Fax: 0121 415 9135
Email: RATE-AF@trials.bham.ac.uk
Website: www.birmingham.ac.uk/RATE-AF

Randomisation

Telephone: 0800 953 0274

Website: https://www.birmingham.ac.uk/RATEAF

Safety Reporting

Fax SAE Forms to: 0121 415 9135 or 0121 415 9136

Protocol Development and Sign Off

Protocol Amendments

The following amendments and/ or administrative changes have been made to this protocol since the implementation of the first approved version

Amendment number	Date of amendment	Protocol version number	Type of amendment	Summary of amendment

For peer review only - http://bmjopen.bmj.com/site/about/guidelines.xhtml

Chief Investigator Signature Page

Trial Name: **RATE-AF**

Protocol Version Number: Version: __ __

Protocol Version Date: __ __ / __ __ __ / __ __ __ __

This protocol has been approved by:

CI Name: Dr Dipak Kotecha

Trial Role: Chief Investigator

Signature and date: ______________________ __ __ / __ __ __ / __ __ __ __

Sponsor Statement

Where the University of Birmingham takes on the sponsor role for protocol development oversight, the signing of the IRAS form by the Sponsor will serve as confirmation of the approval of this protocol.

For peer review only

For peer review only - http://bmjopen.bmj.com/site/about/guidelines.xhtml

Principal Investigator Signature Page

Principal Investigator:

I have read and agree to the protocol, as detailed in this document. I agree to adhere to the protocol as outlined and agree that any suggested changes to the protocol must be approved by the Trial Oversight Committee prior to seeking approval from the Research Ethics Committee and Regulatory Authority.

I am aware of my responsibilities as an Investigator under the guidelines of Good Clinical Practice (GCP), the Declaration of Helsinki, local regulations (as applicable) and the trial protocol and I agree to conduct the trial according to these guidelines and to appropriately direct and assist the staff under my control, who will be involved in the trial.

For peer review only

Trial Name: **RATE-AF**

Protocol Version Number: Version: __ __

Protocol Version Date: __ __ / __ __ __ / __ __ __ __

PI Name: <Enter>

Trial Role: Principal Investigator

Signature and date: ______________________ __ __ / __ __ __ / __ __ __ __

The Principal Investigator should sign this page and return a copy to the RATE-AF Trial Office

For peer review only - http://bmjopen.bmj.com/site/about/guidelines.xhtml

Table of Contents

1 Trial Summary ... 13

1.1 Trial Schema ... 15

2 Introduction ... 16

2.1 Background ... 16

2.2 Epidemiology and Consequences of AF ... 16

2.3 Rhythm-Control in AF ... 17

2.4 Lack of Evidence to Guide Rate-Control Therapy ... 17

2.5 Patient Wellbeing ... 19

2.6 Rationale for the RATE-AF Trial ... 19

3 Trial Design and Objectives ... 20

3.1 Hypothesis ... 20

3.2 Primary objective ... 21

3.3 Secondary objectives ... 21

3.4 Feasibility objectives ... 21

3.5 Exploratory objectives ... 21

4 Selection of Participants ... 22

4.1 Inclusion Criteria ... 22

4.2 Exclusion Criteria ... 22

5 Informed Consent Process ... 23

6 Enrolment and Randomisation ... 24

6.1 Randomisation Procedures ... 25

7 Trial Treatment ... 26

7.1 Treatment ... 26

7.2 Treatment Supply and Storage ... 26

7.3 Dosing Schedule ... 27

7.4 Drug Interactions and Contraindications ... 27

7.5 Accountability Procedures and Labelling ... 29

7.6 Treatment Modification ... 29

7.7 Assessment of Compliance ... 30

8 Trial Procedures and Schedule of Assessments ... 30

8.1 Baseline Visit ... 30

For peer review only

For peer review only - http://bmjopen.bmj.com/site/about/guidelines.xhtml

8.2 Up-Titration Visits 31
8.3 Visit 2, Month 6........ 31
8.4 Visit 3, Month 12 (Final Trial Assessment)........ 32
8.5 Investigator-blinded Endpoints 32
8.6 Long Term Follow-Up 32
8.7 Withdrawal 33
8.8 Trial Duration........ 33
9 Trial Procedures........ 35
9.1 Procedures Defined as Standard Clinical Care 35
9.2 Medical History........ 35
9.3 Medication History 35
9.4 Physical Examination 36
9.5 Patient Reported Outcomes 36
9.5.1 Choice of Outcomes and Qualitative Research 36
9.5.2 Data Collection for PROMs........ 37
9.5.3 Outcome Appraisal........ 38
9.6 Transthoracic Echocardiography 38
9.6.1 Reproducibility and Validity of Measurements 38
9.6.2 Systolic LV Function 38
9.6.3 Diastolic LV Function........ 39
9.6.4 Left Atrial Size and Function........ 40
9.6.5 Additional Echocardiography Parameters........ 40
9.7 Laboratory Evaluations........ 40
9.7.1 Laboratory Assays........ 41
9.7.2 Cellular Response to Rate Control 41
9.7.3 Stored Blood Samples........ 41
9.7.4 Specimen Preparation, Handling, Storage and Shipment........ 41
9.8 Economic Evaluation 41
10 Pharmacovigilance 43
10.1 Recording and Assessment of Adverse Events 43
10.2 Non-Serious Adverse Events/ Adverse Reactions 45
10.3 Serious Adverse Events 45

For peer review only - http://bmjopen.bmj.com/site/about/guidelines.xhtml

10.3.1 Expected SAEs NOT to be Reported on a SAE Form 45

10.4 SUSARs 45

10.5 Development Safety Update Reports 46

10.6 Annual Progress Reports 46

10.7 Pregnancy 46

10.8 Reporting Urgent Safety Measures 46

11 Quality Control and Quality Assurance 47

11.1 Site Set-Up and Initiation 47

11.2 Central Monitoring 47

11.3 Audit and Inspection 47

11.4 Notification of Serious Breaches 48

11.5 Data Handling and Analysis 48

11.6 End of Trial 49

11.7 Archiving 49

12 Statistical Considerations 50

12.1 Outcome measures 50

12.1.1 Primary Outcome 50

12.1.2 Secondary Outcomes 50

12.1.3 Feasibility Outcomes 50

12.2 Power Calculations 51

12.3 Statistical analysis 51

12.3.1 Primary outcome analysis 52

12.3.2 Feasibility and Secondary outcomes analysis 52

12.3.3 Missing data and sensitivity analyses 52

12.3.4 Interim analyses and Stopping rules 52

12.4 Final analysis 53

13 Ethics and Regulatory Requirements 53

14 Oversight Committees 53

14.1 Trial Management Group 53

14.2 Trial Oversight Committee 54

14.3 Protocol amendments 54

15 Finance 54

For peer review only - http://bmjopen.bmj.com/site/about/guidelines.xhtml

16 Confidentiality and Data Protection 54

17 Insurance and Indemnity 55

18 Dissemination and Publication 55

19 Statement of Compliance 56

20 References 57

Appendix A: Randomised treatment arm - Digoxin

Appendix B: Randomised treatment arm - Bisoprolol

For peer review only

For peer review only - http://bmjopen.bmj.com/site/about/guidelines.xhtml

List of Abbreviations

**ABPI**	Association of the British Pharmaceutical Industry
**AE**	Adverse Event
**AF**	Atrial Fibrillation
**BCTU**	Birmingham Clinical Trials Unit
**BNP**	B-type Natriuretic Peptide
**BPM**	Beats per Minute
**CCB**	Calcium Channel Blocker
**CI**	Chief Investigator
**CMR**	Cardiac Magnetic Resonance
**CRF**	Case Report Form
**CTA**	Clinical Trial Authorisation
**CTIMP**	Clinical Trial of Medicinal Product
**DIBD**	Developmental International Birth Date
**DMC**	Data Monitoring Committee
**DSUR**	Developmental Safety Update Report
**DT**	Deceleration Time
**ECG**	Electrocardiogram
**EHRA**	European Heart Rhythm Association
**EU**	European Union
**EudraCT No.**	European Union Drug Regulating Authorities Clinical Trials Number
**GCP**	Good Clinical Practice
**GFR**	Glomerular Filtration Rate
**GP**	General Practitioner
**HF**	Heart Failure
**HR**	Hazard Ratio
**IMP**	Investigational Medicinal Product
**INR**	International Normalised Ratio
**ISRCTN**	International Standard Randomised Controlled Trial Number
**IVRT**	Isovolumic Relaxation Time
**LA**	Left-Atrial
**LV**	Left-Ventricular
**LVEDD**	Left-Ventricle End-Diastolic Dimension
**LVEDV**	Left-Ventricle End-Diastolic Volume
**LVEF**	Left-Ventricular Ejection Fraction

For peer review only

For peer review only - http://bmjopen.bmj.com/site/about/guidelines.xhtml

**LVESD**	Left-Ventricle End-Systolic Dimension
**LVESV**	Left-Ventricle End-Systolic Volume
**LVSD**	Left-Ventricular Systolic Dysfunction
**MHRA**	Medicines and Healthcare Products Regulatory Agency
**MREC**	Main Research Ethics Committee
**NHS**	National Health Service
**NICE**	National Institute of Clinical Excellence
**NOAC**	Novel Oral Anticoagulants
**NYHA**	New York Health Association
**PEF**	Preserved Ejection Fraction
**PI**	Principal Investigator
**PIC**	Patient Identification Centre
**PIL**	Participant Information Leaflet
**PROBE**	Prospective Randomised Open Blinded End-point
**QALY**	Quality-Adjusted Life Year
**QoL**	Quality of Life
**R&D**	Research and Development
**RCT**	Randomised Controlled Trial
**REC**	Research Ethics Committee
**SAE**	Serious Adverse Event
**SAR**	Serious Adverse Reaction
**SD**	Standard Deviation
**SmPC**	Summary of Product Characteristics
**SUSAR**	Suspected Unexpected Serious Adverse Reaction
**TAPSE**	Tricuspid Annular Plane Systolic Excursion
**TDI**	Tissue Doppler Imaging
**TMF**	Trial Master File
**TMG**	Trial Management Group
**TSC**	Trial Steering Committee
**UHB**	University Hospitals Birmingham
**WTCRF**	NIHR Wellcome Trust Clinical Research Facility at Queen Elizabeth Hospital, Birmingham

For peer review only - http://bmjopen.bmj.com/site/about/guidelines.xhtml

1 Trial Summary

**Title**	**Evaluating different rate control therapies in permanent atrial fibrillation: A prospective, randomised, open-label, blinded endpoint trial comparing digoxin and beta-blockers as initial rate control therapy**<br><br>**RAte control Therapy Evaluation in Atrial Fibrillation: RATE-AF**
**Acronym**	**RATE-AF**
**Trial Design and Methods**	A prospective, randomised, open-label, blinded-endpoint (PROBE) trial design. The RATE-AF trial combines hypothesis testing (quality of life, cardiac function, exercise capacity and biomarkers), evaluation of measures (validity, reproducibility and correlation of outcomes) and a feasibility study for a future clinical event trial (assessing recruitment, retention and sample size).
**Trial Medications**	Digoxin 62.5 – 250 μg od<br>Bisoprolol 1.25 – 15 mg od
**Trial Outcomes**	**Primary Outcome:**
	Patient-reported quality of life (QoL): SF-36 physical component summary score at six months
	**Secondary Outcomes:**
	Patient-reported QoL:<br>• SF-36 global and domain-specific scores at 6 and 12 months<br>• EQ-5D-5L summary index and visual analogue scale at six and twelve months<br>• AFEQT overall score at six and twelve months
	Cardiac function:<br>• Echocardiographic LVEF at 12 months<br>• Diastolic function (E/e’ and composite of diastolic indices) at 12 months
	Functional assessment:<br>• Six-minute walking distance at 6 and 12 months<br>• Change in European Heart Rhythm Association (EHRA) class at 6 and 12 months
	Biomarkers:<br>• Change in B-type natriuretic peptide (BNP) levels at 6 months
	Change in heart rate using 24-hour ambulatory ECG
	**Feasibility Outcomes:**
	Recruitment target of 3 patients per week across all participating centres.
	Compliance and reasons for non-compliance
	Number of withdrawals and losses to follow-up (with reasons)
	Drug discontinuation rate and adverse reactions requiring drug discontinuation.
	Number of patients needing therapy-induced requirement for additional treatment

For peer review only - http://bmjopen.bmj.com/site/about/guidelines.xhtml

	Population-specific standard deviations (SD) and proportions:<br>• SD of SF36 physical functioning score at 6 and 12 months<br>• SD of SF36 overall score at 6 and 12 months<br>• SD of AFEQT overall score at 6 and 12 months<br>• SD of LVEF and E/e' scores at 6 and 12 months<br>• Unplanned hospitalisation admissions rates
	Cardiovascular Events (particularly mortality, thromboembolic events, myocardial infarction and cardiovascular interventions)
**Trial Duration per Participant**	12 months of trial therapy
**Planned Trial Sites**	Multiple screening sites with single site recruitment
**Total Number of Participants**	160
**Main Inclusion/ Exclusion Criteria**	**Inclusion Criteria**
	Adult patients, aged 60 years or older
	Permanent AF, characterised (at time of randomisation) as a physician decision for rate-control with no plans for cardioversion, anti-arrhythmic medication, or ablation therapy
	Symptoms of breathlessness (New York Heart Association Class II or more)
	Able to provide written, informed consent
	**Exclusion Criteria**
	Established indication for beta-blocker therapy, e.g. myocardial infarction in the last 6 months
	Known contraindications for therapy with beta-blockers or digoxin, e.g. a history of severe bronchospasm that would preclude use of beta-blockers, or known intolerance to these medications
	Baseline heart rate <60 bpm
	History of second or third-degree heart block
	Supraventricular arrhythmias associated with accessory conducting pathways (e.g. Wolff-Parkinson-White syndrome) or a history of ventricular tachycardia or fibrillation
	Planned pacemaker implantation (including cardiac resynchronisation therapy), pacemaker-dependent rhythm or history of atrioventricular node ablation
	Decompensated heart failure (evidenced by need for intravenous inotropes, vasodilators or diuretics) within 14 days prior to randomisation
	A current diagnosis of obstructive hypertrophic cardiomyopathy, myocarditis or constrictive pericarditis
	Received or on waiting list for heart transplantation
	Receiving renal replacement therapy
	Major surgery, including thoracic or cardiac surgery, within 3 months of randomisation
	Severe, concomitant non-cardiovascular disease (including malignancy) that is expected to reduce life expectancy

For peer review only - http://bmjopen.bmj.com/site/about/guidelines.xhtml

1.1 Trial Schema

Figure 1

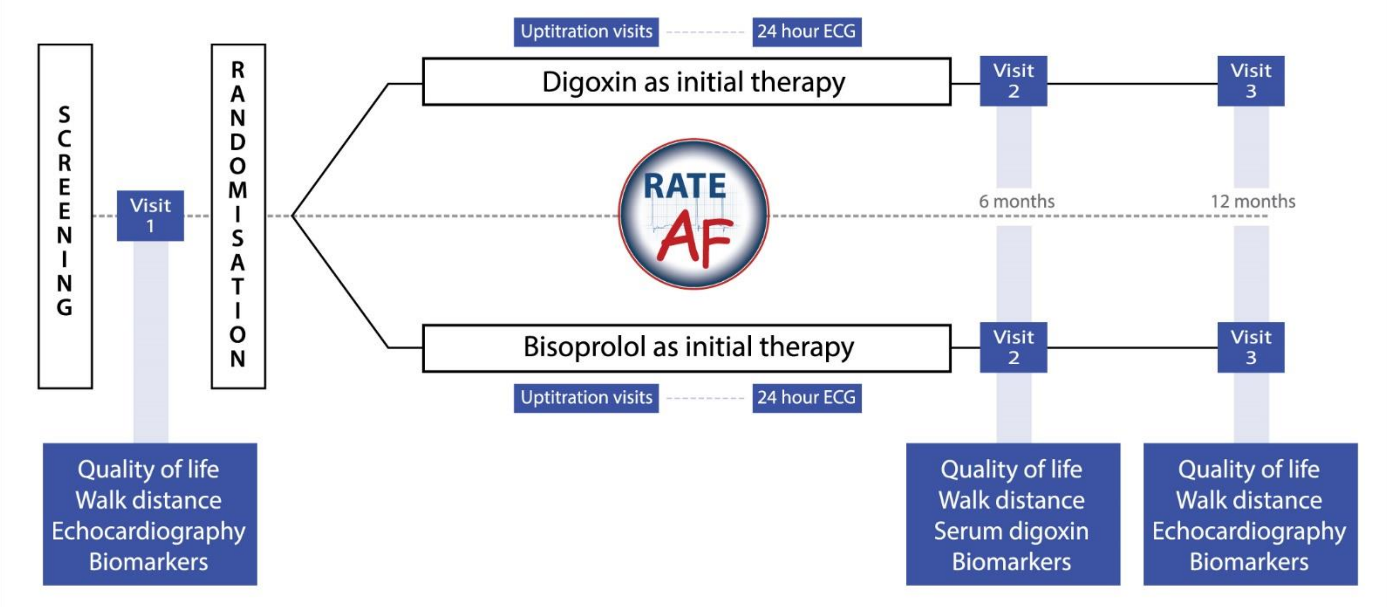

Inclusion Criteria:

- Age 60 years or older
- Permanent AF, characterised as a physician decision for rate control only
- Breathless; New York Heart Association Class II or above
- Able to provide written informed consent

Exclusion criteria:

- Established indication for beta-blocker therapy (e.g. myocardial infarction in the last 6 months)
- Known contraindication for beta-blockers or digoxin (e.g. history of severe bronchospasm or known intolerance)
- Baseline heart rate <60 bpm, 2nd/3rd degree block, accessory pathway or history of ventricular arrhythmia
- Planned pacemaker implantation, pacemaker-dependent rhythm or previous atrioventricular node ablation
- Decompensated heart failure within 14 days requiring intravenous inotropes, vasodilators or diuretics
- Hypertrophic cardiomyopathy, myo/pericarditis or previous/planned heart transplant
- Receiving renal replacement therapy
- Major surgery within 3 months or severe concomitant disease expected to reduce life expectancy

This protocol describes the **RATE-AF** trial only. The trial will be conducted in accordance with the protocol, Good Clinical Practice (GCP) and applicable regulatory requirements. Every care has been taken in the drafting of this protocol, but future amendments may be necessary, which will receive the required approvals prior to implementation.

For peer review only - http://bmjopen.bmj.com/site/about/guidelines.xhtml

2 Introduction

2.1 Background

Atrial fibrillation (AF) is an increasingly common cardiac condition that leads to a substantial burden on quality-of-life (QoL), an increased risk of cardiovascular events, hospitalisation and death, and significant healthcare costs for the NHS. In addition to anticoagulation and considerations for rhythm control therapy, most patients with AF are in need of pharmacological control of heart rate. This aspect of care has not received stringent investigation, with treatment guidelines based on small crossover studies and observational data rather than robust controlled trials.[1-3] Beta-blocker monotherapy remains the first-line option in the current NICE AF guidelines consultation document, with digoxin only for sedentary patients, although this recommendation is based on 'very low-quality evidence'.[4] The benefit of different rate-control therapies on symptoms and other intermediate outcomes (such as left-ventricular ejection fraction [LVEF] and diastolic function) are unknown, as are their effects on clinical events such as hospitalisation. This situation is unacceptable in light of the potential benefits and risk of different rate-control options in AF. It also limits our ability to personalise treatment according to patient characteristics.

The RAte control Therapy Evaluation in Atrial Fibrillation (**RATE-AF**) trial is informed by a number of in-depth systematic reviews of management and clinical outcomes in AF patients.[5-11] Taken together, this information provides a sound basis to plan a major randomised controlled trial (RCT).[12, 13] However as trials of rate-control in AF have typically been small or uncontrolled, further information is needed before designing a trial that can assess clinical outcomes. The **RATE-AF** trial will allow us to define appropriate primary and secondary outcome measures and their standard deviation in a contemporary population of patients with permanent AF. This information will allow us to estimate sample size, determination of recruitment, retention and adherence policies, and to ascertain the best methods of obtaining adverse event data and reliable economic costs for a larger trial assessing cardiovascular outcomes and hospitalisation. The **RATE-AF** trial will also be the largest RCT of its kind, allowing us to compare the effect of beta-blockers and digoxin on QoL as initial rate-control therapy in patients with permanent AF. The long-term aim of the research is to answer key questions about how to initiate therapy, stratified by relevant patient characteristics such as systolic and diastolic cardiac function, baseline symptoms and concurrent medication. The research will also define the patho-physiological mechanisms underlying AF-related symptoms, left-ventricular function and their association with adverse clinical outcomes, and to identify clinical markers for the response to different rate control therapy.

2.2 Epidemiology and Consequences of AF

AF is a common condition that is associated with increased rates of mortality and serious morbidity, including stroke, worsening of heart failure, sudden death, and reduced QoL.[1] The prevalence of AF increases with age, ranging from 0.7% in those aged 55–59 years to 17.8% in those aged above 85.[14] A doubling of both incidence and prevalence of AF is predicted in the next 20 years.[15]

For peer review only - http://bmjopen.bmj.com/site/about/guidelines.xhtml

Patients with AF are twice as likely to be hospitalised as propensity score-matched controls, with direct medical costs estimated to be 73% higher.[16] Further, AF is an independent predictor of all-cause mortality, with a two-fold adjusted increase in death.[17, 18] While most strokes in AF can be prevented by oral anticoagulation, AF patients still have high cardiovascular death rates due to sudden death or progressive heart failure.[19, 20] Patients with AF also have significantly poorer QoL[21], experiencing a variety of symptoms including lethargy, palpitations, dyspnoea, sleeping difficulties and psychosocial distress.[22, 23] In the context of patients diagnosed with heart failure, the presence of AF leads to higher rates of death and hospitalisation, independent of other risk variables or which condition comes first.[24, 25] From observational data, 40% of AF patients will be diagnosed with heart failure and vice-versa[16], representing a large and growing unmet clinical need for healthcare improvement.

2.3 Rhythm-Control in AF

Numerous large RCTs comparing rhythm-control (using arrhythmic drugs and/or cardioversion) versus rate-control have identified no significant difference in clinical outcomes in patients with persistent AF.[26-30] In a number of studies, hospitalisation rates were actually higher in those randomised to rhythm-control.[26, 29, 30] Similar findings have been shown in AF patients with heart failure[31, 32], both in those with impaired and preserved ejection fraction.[33-35] Although AF ablation is becoming increasingly popular to restore sinus rhythm, it remains a highly invasive method to improve AF-related symptoms.[36, 37] At present, European and NICE treatment guidelines recommend ablation only in symptomatic paroxysmal AF, or as a treatment option in symptomatic persistent AF that is refractory to other therapy.[3] Further trials are currently underway to determine the clinical value of prompt rhythm-control, including the Early treatment of Atrial fibrillation for Stroke prevention Trial (EAST).[38] In light of the high recurrence rate of AF (even in patients receiving intensive rhythm-control therapy), rate-control is an important part of AF management in almost all patients. Unfortunately, rate-control therapy has much less evidence underpinning its use.

2.4 Lack of Evidence to Guide Rate-Control Therapy

Rate-control in AF can be achieved with beta-blockers, non-dihydropyridine calcium-channel blockers (CCB), digoxin and their combinations. Unfortunately, little data exists to assist clinicians in choosing appropriate first-line and subsequent therapy. Current patterns of medication usage vary considerably (between and within countries). For example, in a worldwide registry, digoxin was prescribed in 2877 of 10,523 patients (27.3%), compared to 1599 of 3141 (50.9%) of patients in the German Competence NETwork on Atrial Fibrillation (AFNET).[39, 40]

Current European guidelines suggest “the choice of medication should be individualised and the dose modulated to avoid bradycardia”. This recommendation (Class 1, Level B) is based on a systematic review of trials addressing rate-control between 1983 and 1997.[41] Most of the studies included less than 50 participants (with several less than 10). The majority were low quality studies, as assessed by the risk of bias or confounding, and follow-up was typically in the order of

For peer review only - http://bmjopen.bmj.com/site/about/guidelines.xhtml

hours, days or weeks. Whilst this may be sufficient to assess an acute effect on heart-rate, it provides limited data on the longer-term effects of different treatments or the frequency of adverse reactions.

Beta-blockers are often preferred over other agents due to the prognostic benefit seen in patients with heart failure who are in sinus rhythm. However, in patients with heart failure, reduced LVEF and concomitant AF, we have shown that beta-blockers do not reduce mortality (hazard ratio 0·97, 95% CI 0.83-1.14; p=0.73) or cardiovascular hospital admissions (hazard ratio 0·91; 95% CI 0.79-1.04; p=0.15).[5] This distinctly contrasts with the significant benefit seen in patients with sinus rhythm and highlights the need for further comparative RCTs specifically in patients with AF.

The most highly cited trial comparing beta-blockers and digoxin for rate-control in chronic AF was an open-label two-week crossover study of 5 drug regimes in 12 patients.[42] Peak heart-rate after exercise was significantly higher in those taking digoxin compared to beta-blockers but there were no differences in exercise duration. In a trial of 42 patients, rate-control was improved with combination beta-blocker/digoxin therapy compared to digoxin alone, however there were similarly no differences in exercise capacity.[43] Systematic review of other small randomised studies identify no difference in exercise tolerance with beta-blockers, despite a lowering of heart-rate.[44] From observational data, such as the Atrial Fibrillation Follow-up Investigation of Rhythm Management (AFFIRM) study, more cardiac and non-cardiac adverse effects have been noted with beta-blockers than digoxin (n=67 vs. n=38).[28] In a 3-week crossover study of 60 participants, 10% withdrew during beta-blocker therapy due to adverse events.[45] Those in the beta-blocker group had a reduction in exercise capacity on cardio-pulmonary testing and a significant increase in B-type natriuretic peptide (BNP, a marker of ventricular strain) compared to patients treated with calcium-channel blockers.[46]

Only a single RCT has been published comparing digoxin and beta-blockers in patients with AF and heart failure (mean LVEF 24%, n=47).[47] Although there was a marginally-significant improvement in LVEF with carvedilol/digoxin versus placebo/digoxin, blinded withdrawal of digoxin then led to a deterioration in LVEF, accompanied by an increase in BNP. There was no difference in the number of heart-rate pauses >3 seconds or in daytime/exercise heart-rate comparing the two therapies alone.

Digoxin itself has been associated with an increased mortality in observational cohorts of AF patients[48], however careful adjustment of baseline differences reject a true excess in adverse outcomes.[49-51] In a detailed systematic review of all studies published on digoxin, we identified that confounding was the main reason that digoxin was associated with increased mortality in observational studies, and confirmed a neutral association in RCTs (risk ratio 0.99, 95% CI 0.93 to 1.05).[6] Lower rates of hospitalisation have been noted with digoxin therapy, independent of the type of heart failure[52], however the lack of randomised data versus placebo (despite widespread clinical use) makes true comparison difficult. Small RCTs comparing CCB with digoxin have been inconsistent; two have identified lower heart-rates with CCB but no significant difference in exercise capacity[42, 43], one demonstrated higher post-exercise cardiac output after

For peer review only - http://bmjopen.bmj.com/site/about/guidelines.xhtml

digoxin[53] and another showed improved exercise duration and QoL with CCB.[54] These results highlight the need for randomised data with appropriately-defined outcomes to accurately identify the benefits and risks of common therapies in patients with AF.

An example where RCT data have impacted on clinical practice is the Rate Control Efficacy in Permanent Atrial Fibrillation (RACE II) trial. This study challenged conventional wisdom that stricter control of heart-rate would allow time for diastolic filling and improve haemodynamics. In summary, 614 patients with permanent AF were randomised to strict or lenient rate-control and followed for 2-3 years.[55] There was no significant difference in the cumulative incidence of the composite primary outcome; 14.9% in the strict-control arm and 12.9% in the lenient-control group. There were also no differences in symptoms, New York Heart Association (NYHA) class or hospitalisations[55, 56], no interaction with baseline heart failure[57], and those participants achieving strict rate-control required more clinic visits and higher doses of medical therapy.[58] Current guidelines therefore suggest that lenient rate-control is acceptable, except for patients with adverse symptoms or clinical deterioration.[1] Whilst this study provides important data on the intensity of rate-control in AF, the more clinically-relevant questions of how to initiate therapy and the choice of optimal agents for individual patients remain unanswered.

2.5 Patient Wellbeing

Patient-reported outcomes are any report of a patient's health status (for example QoL) that is derived directly from the patient, without interpretation by a clinician.[59] There is limited data on the effect of pharmacological rate-control therapy on QoL and no comparative data assessing the benefit of different strategies.[22, 60] Rate-control has been associated with improved QoL scores in trials assessing rate versus rhythm-control.[61, 62] In the PIAF study, over 50% of participants randomised to calcium-channel blockers reported an improvement in health with significant benefits in the physical aspects of the SF-36.[63] A number of smaller studies have shown inconsistent effects on QoL in AF, although the data is limited by inclusion of patients with paroxysmal AF, a focus on heart rate and the use of a variety of QoL tools.

Current QoL questionnaires can be divided into disease-specific evaluations or generic health assessments (such as the Short Form Health Survey SF-36[64] or the EuroQol EQ-5D[65, 66]). However there is a distinct lack of knowledge regarding the mechanisms that underpin AF-related symptoms, the responsiveness of QoL questionnaires and their validity.[60] The Atrial Fibrillation Effect on QualiTy-of-life (AFEQT) questionnaire was designed to address these disparities by using more robust methods.[67] Although there is limited clinical application to-date, AFEQT has demonstrated sensitivity to clinical change.[68] An important objective of the research is to ascertain appropriate and responsive QoL tools for this population, as well as determine the acceptability and delivery of the questionnaires to patients.

2.6 Rationale for the RATE-AF Trial

Rate-control is an integral part of management in all AF patients but hardly any controlled trial evidence exists to guide the choice of agents. We have shown that neither beta-blockers nor

For peer review only - http://bmjopen.bmj.com/site/about/guidelines.xhtml

digoxin has an impact on mortality in AF patients, even with concomitant heart failure, which highlights the need to determine treatment effects on quality of life and cardiac function.

3 Trial Design and Objectives

RATE-AF is Prospective, Randomised Open-label Blinded Endpoint (PROBE) clinical trial comparing the use of digoxin and beta-blockers as initial rate control therapy.

In this section, we discuss the trial design and study objectives. Detailed outcome measures are listed in **Section 12**.

For peer review only

Justification for a PROBE rather than a Double Blind Trial Design

Although a double blind design would be the most robust trial design with respect to bias, it would not be ethical to do so in this scenario as clinicians would feel the need to add therapy according to heart rate. In addition, the RATE-AF Trial aims to test a strategy of initial care. PROBE trial design maintains the benefits associated with a strict randomisation procedure, while the blinded end points help to eliminate bias.

The trial design aims for a pragmatic 'all-comers' approach, applicable to those seen in clinical practice to allow transfer of the findings to routine clinical management of patients with permanent AF.

Assessment and Management of Risk

This trial is categorised by the Medicines and Healthcare products Regulatory Agency (MHRA) as:

Type A = No higher than the risk of standard medical care

The assessment and management of risk is detailed in the separate **RATE-AF** Risk Assessment document. An on-going evaluation of risk will continue throughout the recruitment period.

3.1 Hypothesis

Null Hypothesis for primary outcome:

No difference in patient-reported quality of life (measured using the physical functioning domain of the SF36 questionnaire) when comparing a strategy of digoxin versus beta-blocker therapy for initial rate control in patients with permanent AF.

Alternative Hypothesis:

Use of digoxin or beta-blocker therapy as initial rate control in patients with permanent AF is superior based on patient reported quality of life (measured using the physical functioning domain of the SF36 questionnaire).

For peer review only - http://bmjopen.bmj.com/site/about/guidelines.xhtml

3.2 Primary objective

- Patient-reported quality of life (QoL), with a predefined focus on physical well-being using the SF-36 physical component summary at six months.

3.3 Secondary objectives

- Generic and AF-specific patient-reported QoL using the SF-36 global and domain-specific scores, the AFEQT overall score and the EQ-5D-5L summary index and visual analogue scale at six and twelve months.
- Echocardiographic left-ventricular ejection fraction (LVEF) and diastolic function (E/e' and composite of diastolic indices) at twelve months.
- Functional assessment, including 6-minute walking distance achieved, change in European Heart Rhythm Association (EHRA) class and cognitive function at six and twelve months.
- Change in B-type natriuretic peptide (BNP) levels as a surrogate for total cardiac strain at six months.
- Change in heart rate from baseline and group comparison using 24-hour ambulatory ECG.

3.4 Feasibility objectives

- Successful methods for recruitment
- Key issues that affect retention of participants, such as convenience, compliance and cross-over (target of 85% study completion rate).
- Drug discontinuation rate and adverse reactions leading to drug discontinuation.
- Therapy-induced requirement for additional treatment (e.g. pacemaker implantation).
- Population-specific standard deviations and proportions to enable sample size calculation for a future trial.
- Assessment of cardiovascular outcomes including a composite of adverse clinical events (mortality, thromboembolic events, myocardial infarction and cardiovascular interventions).

3.5 Exploratory objectives

- Correlation of baseline measures, including QoL questionnaires and unblinded baseline investigations such as QoL, BNP, LVEF, E/e', EHRA, intracellular methods and heart rate.
- Impact of therapy on intracellular sodium and calcium concentration and cardiotonic steroid levels as biomarkers of cellular response at six and twelve months.

For peer review only - http://bmjopen.bmj.com/site/about/guidelines.xhtml

- Impact of combination therapy on outcomes, including comparison of bisoprolol/non-dihydropyridine calcium channel blocker (CCB) vs. bisoprolol/digoxin vs. digoxin/CCB vs. single therapies.
- Change in cognitive function at twelve months
- Qualitative research of patient-reported QoL using focus groups to explore patient acceptability, optimal delivery methods and responsiveness.
- Assessment of the validity and reproducibility of echocardiographic measures in patients with AF.
- Correlation of serum digoxin concentration with change in QoL and intracellular methods.
- Cost-consequence economic analysis from an NHS perspective.

4 Selection of Participants

Participants who potentially fulfil the inclusion criteria for this trial must have their eligibility confirmed by medically qualified personnel with access to and a full understanding of the potential participant's medical history. If eligibility has been assessed and documented by medically qualified personnel, then the process of obtaining informed consent may be delegated as appropriate and as documented on the **RATE-AF** Delegation and Signature Log.

4.1 Inclusion Criteria

- Adult patients aged 60 years or older
- Permanent AF, characterised (at time of randomisation) as a physician decision for rate-control with no plans for cardioversion, anti-arrhythmic medication, or ablation therapy
- Symptoms of breathlessness (New York Heart Association Class II or more)
- Able to provide written informed consent

4.2 Exclusion Criteria

- Established clinical indication for beta-blocker therapy, e.g. myocardial infarction in the last 6 months
- Known contraindications for therapy with beta-blockers or digoxin, e.g. a history of severe bronchospasm that would preclude use of beta-blockers, or known intolerance to these medications
- Baseline heart rate <60 bpm
- History of second or third-degree heart block
- Supraventricular arrhythmias associated with accessory conducting pathways (e.g. Wolff-Parkinson-White syndrome) or a history of ventricular tachycardia or fibrillation

For peer review only - http://bmjopen.bmj.com/site/about/guidelines.xhtml

- Planned pacemaker implantation (including cardiac resynchronisation therapy), pacemaker-dependent rhythm or history of atrioventricular node ablation
- Decompensated heart failure (evidenced by need for intravenous inotropes, vasodilators or diuretics) within 14 days prior to randomisation
- A current diagnosis of obstructive hypertrophic cardiomyopathy, myocarditis or constrictive pericarditis
- Received or on waiting list for heart transplantation
- Receiving renal replacement therapy
- Major surgery, including thoracic or cardiac surgery, within 3 months of randomisation
- Severe, concomitant non-cardiovascular disease (including malignancy) that is expected to reduce life expectancy

5 Informed Consent Process

It will be the responsibility of the Investigator to obtain written informed consent for each participant prior to performing any trial related procedure. If local practice allows, this responsibility may be delegated by the Principal Investigator, to a Research Nurse as captured on the Site Signature and Delegation Log. A Participant Information Leaflet (PIL) will be provided to facilitate this process. Investigators or delegate(s) will ensure that they adequately explain the aim, trial treatment, anticipated benefits and potential hazards of taking part in the trial to the participant. They will also stress that participation is voluntary and that the participant is free to refuse to take part and may withdraw from the trial at any time. The participant will be given adequate time to read the PIL and to discuss their participation with others outside of the site research team. The participant will be given the opportunity to ask questions.

If the participant expresses an interest in participating in the trial they will be asked to sign and date the latest version of the Informed Consent Form (ICF). The participant must give explicit consent for the regulatory authorities, members of the research team and representatives of the sponsor to be given direct access to the participant's medical records.

The Investigator or delegate(s) will then sign and date the form. A copy of the ICF will be given to the participant, a copy will be filed in the medical notes, and the original placed in the Investigator Site File (ISF). Once the participant is entered into the trial, the participant's unique trial identification number will be entered on the ICF maintained in the ISF. As part of the consent process, the participant will be asked to give explicit consent to their trial-related information being sent to the Trials Office at the University of Birmingham.

This trial will include **optional consent** to allow linkage to patient data available in NHS routine clinical datasets, including primary care data (e.g. Clinical Practice Research Datalink; CPRD, The Health Improvement Network; THIN, QResearch), secondary care data (Hospital Episode Statistics; HES) and mortality data from the Office of National Statistics (ONS) through The Health and Social Care Information Centre and other central UK NHS bodies. The consent will

For peer review only - http://bmjopen.bmj.com/site/about/guidelines.xhtml

also allow access to other new central UK NHS databases that will appear in the future. This will allow us to double check the main outcomes against routine data sources, and extend the follow-up of patients in the trial and collect long-term outcome and health resource usage data without needing further contact with the trial participants. This is important as it will link a trial of treatments that may become a clinical standard of care to long-term outcomes that are routinely collected in clinical data but which may be collected during the follow-up period of the trial.

Details of the informed consent discussions will be recorded in the participant's medical notes. This will include date of discussion, the name of the trial, summary of discussion, version number of the PIL given to participant and version number of ICF signed and date consent received. Where consent is obtained on the same day that the trial related assessments are due to start, a note will be made in the medical notes as to what time the consent was obtained and what time the procedures started.

At each visit the participant's willingness to continue in the trial will be ascertained and documented in the medical notes. Throughout the trial the participant will have the opportunity to ask questions about the trial. Any new information that may be relevant to the participant's continued participation will be provided. Where new information becomes available which may affect the participants' decision to continue, participants will be given time to consider and if happy to continue will be re-consented. Re-consent will be documented in the medical notes. The participant's right to withdraw from the trial will remain.

Electronic copies of the PIL and ICF will be available from the Trials Office and will be presented on the headed paper of the local institution. Details of all participants approached about the trial will be recorded on the Participant Screening/Enrolment Log and with the participant's prior consent, their General Practitioner (GP) will also be informed that they are taking part in the trial.

6 Enrolment and Randomisation

A flowchart of the recruitment process is shown in the Trial Schema (**Figure 1**) together with the schedule of investigation. **Section 9** gives more detailed information of trial procedures and assessments.

In the majority, potentially eligible participants will be identified by their Cardiologist, usually following referral from their General Practitioner (GP), and provided with an ethically-approved patient information leaflet (PIL). The patient will then be invited to attend a baseline visit at the NIHR Wellcome Trust Clinical Research Facility (WTCRF) at Queen Elizabeth Hospital, Birmingham. Potentially eligible participants may also be identified from inpatient referrals; these patients will be provided with a PIL and invited to attend a baseline visit following the same procedure.

GP Practices in the Birmingham area may be asked to refer patients that present with AF, but are not on medication, to the RATE-AF Research Team at University Hospitals Birmingham (UHB). These patients will be given a one-page, ethics committee-approved trial summary and asked to

For peer review only - http://bmjopen.bmj.com/site/about/guidelines.xhtml

sign a contact details form to confirm that they are happy to be contacted by a member of the Research Team to arrange an appointment.

Prior to patients undertaking any trial-related procedures, informed consent will be obtained.

Details of all patients approached about the trial should be recorded on the **RATE-AF** Screening & Enrolment Log. This Log should be maintained within the Investigator Site File.

6.1 Randomisation Procedures

After all eligibility criteria have been confirmed and informed consent has been received, the participants can be randomised into the **RATE-AF** trial.

Participants will be randomised in a 1:1 ratio to either **Digoxin 62.5 – 250 µg od or Bisoprolol 1.25 – 15 mg od**. The time between randomisation and commencement of trial therapy should be minimised (ideally <24 hours). Randomisation will be provided by a computer generated programme at the Birmingham Clinical Trials Unit (BCTU), using a minimisation algorithm to ensure balance between the arms with regard to important clinical variables, stratifying for baseline EHRA (class 1/2a and 2b/3/4) and gender.

Telephone and Online Randomisation

Participants can be randomised into the trial via a secure 24 hour internet based randomisation service (https://www.trials.bham.ac.uk/RATEAF) or by a telephone call to the BCTU (telephone number **0800 953 0274**). Telephone randomisations are available Monday-Friday, 09:00-17:00. For the secure internet randomisation, each site and each randomiser will be provided with a unique log-in username and password in order to access the online system. Online randomisation is available 24 hours a day, 7 days a week, apart from short periods of scheduled maintenance and occasional network problems.

Randomisation Forms will be provided to investigators and should be completed and used to collate the necessary information prior to randomisation. Once all eligibility criteria have been provided and confirmed, a Trial Number and treatment allocation be given and relevant parties notified, including the participant's GP.

Back-up Randomisation

If the internet based randomisation service is unavailable for an extended period of time, a back-up paper randomisation will also be available at the BCTU. The randomisation list will be produced using a random length block design. In this instance, investigators should ring the BCTU randomisation service (telephone number **0800 953 0274**).

For peer review only - http://bmjopen.bmj.com/site/about/guidelines.xhtml

7 Trial Treatment

7.1 Treatment

The Investigational Medicinal Products (IMPs) for this trial are Digoxin and Bisoprolol.

At randomisation, participants will be allocated to open-label treatment with either Digoxin 62.5 – 250 µg od or Bisoprolol 1.25 – 15 mg od.

Digoxin

Digoxin is a cardiac glycoside derived from the foxglove plant. The cardiac effects of digoxin therapy are summarised by:

- Positive inotropic effects: increased intracellular calcium due to direct inhibition of sodium-potassium adenosine triphosphatase (Na/K-ATPase)
- Negative chronotropic effects: decreased conduction velocity through the atrioventricular node, an increase in the effective refractory period and an increase in vagal activity leading to sinus node depression.

Clinically, digoxin is commonly prescribed in two conditions, heart failure and AF.

Bisoprolol

Bisoprolol fumarate is a highly beta-1 selective adrenoreceptor blocker first approved by the U.S. Food and Drug Administration in 1992. The cardiac effects of bisoprolol therapy are summarised by:

- Negative chronotropic effects: a reduction in resting and exercise heart rate due to prevention of norepinephrine and epinephrine from binding to the beta-receptor in cardiac conduction tissue.
- Negative (mild) inotropic effects: an initial fall in resting and exercise cardiac output with little observed change in stroke volume and only a small increase in right atrial pressure or pulmonary capillary wedge pressure.

Clinically, bisoprolol is commonly prescribed in a range of cardiology conditions, including post-myocardial infarction, heart failure and in patients with atrial tachyarrhythmia, including AF.

7.2 Treatment Supply and Storage

Due to the participant population and the fact that the trial closely aligns with standard care, trial medication may be dispensed from routine standard stock by both the pharmacy at the research site and community pharmacies local to the participant. Both treatments are used as per normal clinical practice therefore there is no additional requirement, above that of local policy, to monitor temperature during storage.

For peer review only - http://bmjopen.bmj.com/site/about/guidelines.xhtml

Digoxin

Digoxin is available as an oral tablet in doses of 62.5, 125 and 250 µg or as an elixir (50 µg/mL). It is packaged in 28 or 500 tablet packs under the generic title digoxin and trade label Lanoxin.[69] Digoxin should be stored according to local policy.

Bisoprolol

Bisoprolol is available as an oral tablet in doses of 1.25, 2.5, 3.75, 5.0, 7.5 and 10 mg. It is packaged as 28 tablets under the generic title bisoprolol fumarate and trade labels Cardicor and Emcor.[69] Bisoprolol should be stored according to local policy.

7.3 Dosing Schedule

Digoxin

An advice sheet for the investigator is presented in **Appendix A.**

Trial maintenance doses will initially be 62.5 or 125 µg orally (at the clinician's discretion, taking into account age and renal function), with planned up-titration to 125/250 µg. The maximum trial dose will be 250 µg daily.

A single loading dose of four tablets (250 or 500 µg according to target maintenance dose) will be prescribed in digoxin-naïve participants. The clinician is permitted to omit the loading dose or prescribe a second, where necessary.

Unblinded serum digoxin concentrations will be assessed at visits 2 and 3, with results reported back to the relevant clinician(s). This process will assist in monitoring compliance, adjusting dosage in cases of low serum levels and avoiding toxicity.

Bisoprolol

An advice sheet for the investigator is presented in **Appendix B**

Trial starting doses will be 1.25 or 2.5 or 5 mg (at the clinician's discretion), with planned up-titration to 10 mg in increments of 1.25 or 2.5 mg. The maximum trial dose will be 15 mg daily. No loading dose is required.

Plasma concentrations have not shown to be associated with toxicity and are not part of standard clinical practice.

7.4 Drug Interactions and Contraindications

Digoxin

Following oral administration of digoxin, approximately 60–85% of the dose is usually absorbed, mainly from the small intestine. The onset of action is 0.5-2 hours and maximal effects occur in

For peer review only - http://bmjopen.bmj.com/site/about/guidelines.xhtml

2-6 hours. Digoxin has a large volume of distribution and approximately 20-30% of digoxin in blood is bound to plasma proteins. Metabolism is minimal but variable, with the majority of drug excreted unchanged in the urine by glomerular filtration and tubular secretion. With normal renal function, the elimination half-life is 34-44 hours which is prolonged in patients with renal failure by two to threefold. Dose adjustment is unnecessary in patients with hepatic impairment. Therapeutic plasma concentrations of digoxin have been described as 0.5-2.0 ng/mL.[70] In digoxin-naïve patients with normal renal function, approximately seven days are required to reach steady-state therapeutic concentrations if a loading dose is omitted. As such, the majority of clinicians prescribe one or two loading doses, totalling 500 to 1000 μg over 24 hours.

Caution is recommended in patients with electrolyte disturbance (due to increased risk of toxicity) and reduced doses are recommended in patients with renal impairment. There are no concerns in pregnancy or with breast-feeding, although dose adjustment may be required.

Contraindications for digoxin therapy include heart block, accessory pathway supraventricular tachycardia and a current diagnosis of obstructive hypertrophic cardiomyopathy, myocarditis or constrictive pericarditis.

Digoxin has been associated with a number of adverse effects, although data from randomised trials show little difference in comparison to placebo, apart from cases of toxicity (2% versus 0.9% respectively in the DIG trial of patients with HF)[71]. The most common side effects are gastrointestinal upset, dizziness, blurred vision, headache and rash. In toxic states (serum levels >2 ng/mL), digoxin is pro-arrhythmic and can aggravate heart failure, particularly with co-existent hypokalaemia. In cases of overdose, repeated early doses of activated charcoal may be given to reduce absorption and in severe toxicity, digoxin-specific antibody fragments are available as an intravenous infusion.

In rigorous assessment, drug interactions with digoxin have proved inconsistent.[72] Serum digoxin concentrations are increased by amiodarone, dronedarone, propafenone and quinidine but increased bioavailability with CCB and certain antibiotics (such as erythromycin and tetracycline) only occur in selected patients. The risk of toxicity increases with drugs that cause electrolyte disturbances, such as thiazide and loop diuretics.

Bisoprolol

Following oral administration of digoxin, the absolute bioavailability is approximately 80%, first pass metabolism of 20% and 30% protein binding. Peak plasma concentrations occur within 2-4 hours, the elimination half-life is 9-12 hours and steady state is attained within 5 days. Elimination occurs equally by renal and non-renal pathways with about 50% of the dose remaining unchanged in the urine.

Caution is recommended in patients with first-degree heart block, portal hypertension, diabetes, a history of obstructive airways disease, myasthenia gravis, a history of hypersensitivity and psoriasis, although many cardiologists use beta-blockers frequently in these groups with appropriate supervision. In pregnancy, beta-blockers may cause intra-uterine growth restriction,

For peer review only - http://bmjopen.bmj.com/site/about/guidelines.xhtml

neonatal hypoglycaemia, and bradycardia (although as above, these agents are frequently used in pregnancy). There is a theoretical risk of toxicity in breast feeding, although the amount present in milk is likely too small to affect infants. Abrupt withdrawal should be avoided, especially in cases of ischaemic heart disease. Up-titration should be more cautious in patients with renal or hepatic impairment.

Contraindications for bisoprolol therapy include cardiogenic shock, overt cardiac failure, second or third degree heart block, marked sinus bradycardia and severe peripheral arterial disease.

Bisoprolol has been associated with a wide variety of adverse effects although data from RCTs suggest similar discontinuation rates compare to placebo.[5, 73] The most common adverse symptoms are lethargy, headache, peripheral oedema, upper respiratory tract symptoms, gastrointestinal upset and dizziness. In cases of overdose, bradycardia, hypotension, congestive heart failure, bronchospasm and hypoglycaemia may be expected, with treatment directed to supportive methods and atropine, fluids, glucagon or diuretics as required.

Pharmacokinetic interactions with beta-blockers have not shown to be clinically significant. Drugs that reduce absorption include aluminium salts and cholestyramine, whilst metabolism can be increased by barbiturates and rifampicin and decreased with cimetidine, erythromycin, fluvoxamine, and hydralazine.

7.5 Accountability Procedures and Labelling

Through the risk-adapted approach, a full risk assessment of the **RATE-AF** trial has been conducted including the drug accountability requirements. The IMPs will be used within their authorisations, prescribed on an NHS prescription and dispensed by pharmacy from standard stock. The risk assessment has determined that a normal dispensing label is appropriate and an additional clinical trial label is not necessary (as covered by Regulation 46 (2) of SI 2004/1031). Drug accountability will be according to standard practice for NHS prescriptions. Details of how compliance will be assessed can be found in **Section 7.7**.

7.6 Treatment Modification

Patients that withdraw from medication for any reason will do so under strict clinical supervision.

The trial is designed to assess the impact of **initial** impact of rate control therapy; it is expected that treatments will modify during the trial period (in particular, the addition of therapy to attain heart rate targets). Patients will not be withdrawn from the trial if they commence therapy from the other arm of the trial due to any absolute or relative clinical indications (for example, patients in the digoxin arm starting beta-blockers due to incident myocardial infarction, or heart failure with reduced LVEF).

For peer review only - http://bmjopen.bmj.com/site/about/guidelines.xhtml

7.7 Assessment of Compliance

We will ask participants about compliance with their trial medication at each follow-up visit and this will be documented in the CRFs. It may also be clinically evident from the heart rate check, performed as part of all visits, whether or not the patient has been compliant with their trial mediation.

In addition, patients that are randomised to the digoxin arm will have a serum digoxin sample taken as part of Visit 2 (month 6) and Visit 3 (month 12) follow-ups. The results will indicate whether the patient has been compliant with their trial medication.

For peer review only

8 Trial Procedures and Schedule of Assessments

8.1 Baseline Visit

The baseline visit will occur as soon as possible after screening and will involve the following procedures (see **Section 9** for procedure details):

- Verify inclusion/exclusion criteria.
- Obtain written informed consent from the potential participant.
- Randomise the patient via telephone or the secure web-based system as outlined in **Section 6**
- Administer QoL and functional capacity questionnaires.
- Review recent blood results (within 6 months of Baseline Visit)
  - Assessing renal function to aid in dose assignment and serum potassium level as part of standard clinical care.
- Document the use of oral anticoagulation and arrange appropriate prescription for patients not on therapy according to clinical guidelines. If the participant is already receiving vitamin-K antagonists (VKA), recent INR results will be documented.
- Record results of physical examinations.
- Collect blood samples for baseline blood tests and biomarker analysis.
- Complete case report form (CRF)
- Perform a 12-lead electrocardiogram.
- Supervise a 6-minute walk test, recording distance walked and peak heart rate.
- Arrange the baseline echocardiogram; images will be delivered to the echocardiographic core laboratory for blinded reporting.
- Discuss the randomised allocation with the participant including schedule for drug therapy and up-titration.

For peer review only - http://bmjopen.bmj.com/site/about/guidelines.xhtml

Participants will be followed up by telephone call two weeks after the Baseline Visit to ensure they have commenced trial medication.

8.2 Up-Titration Visits

For the majority of participants, two up-titration visits will be planned to supervise the appropriate use of medications as per the up-titration schedule (see **Appendices A and B**). Additional up-titration visits, as required, are acceptable in order to attain a heart rate at rest of ≤100bpm.

Up-titration visits will involve the following procedures:

- Record adverse events as reported by the participant or observed by the investigator.
- Review of medications and plan for trial drug up-titration
- Assessment of compliance
- Symptom-directed clinical examination
- Vital signs, including heart rate and blood pressure
- Administer QoL and functional capacity questionnaires (last uptitration visit only).
- Organise a 24-hour ambulatory ECG once up-titration completed (results to be forwarded to the clinician).

8.3 Visit 2, Month 6

Visit 2 will occur at an interval of six months (± four weeks) after the Baseline Visit and involve the following procedures:

- Administer QoL and functional capacity questionnaires.
- Record adverse events as reported by the participant or observed by the investigator.
- Confirm current rate control therapy (including dosage) and check concomitant medications.
- Assessment of compliance.
- Collect blood samples for biomarker analysis.
- Collect blood sample for serum digoxin concentration, potassium and creatinine as part of standard clinical care.
- Record time in therapeutic range for patients on anticoagulation with vitamin-K antagonists and compliance in patients receiving non-VKA oral anticoagulants.
- Obtain a twelve lead ECG.
- Record the results of physical examinations and vital signs.
- Supervise a 6-minute walk test, recording distance walked and peak heart rate.
- Complete other CRF requirements.
- If an echocardiogram has been performed for clinical reasons since the previous visit, images will be retrieved and sent to the core echocardiographic laboratory.

For peer review only - http://bmjopen.bmj.com/site/about/guidelines.xhtml

- Confirm appointment date for Visit 3.

8.4 Visit 3, Month 12 (Final Trial Assessment)

Visit 3 will occur at an interval of 12 months (± four weeks) after the Baseline Visit and involve the following procedures:

- Administer QoL and functional capacity questionnaires.
- Record adverse events as reported by the participant or observed by the investigator.
- Confirm current rate control therapy (including dosage) and check concomitant medications.
- Assessment of compliance.
- Transthoracic echocardiography (as per **Section 9.6**), with images delivered to the echocardiographic core laboratory for blinded reporting.
- Collect blood sample for serum digoxin concentration, potassium and creatinine as part of standard clinical care.
- Record time in therapeutic range for patients on anticoagulation with vitamin-K antagonists and compliance in patients receiving non-VKA oral anticoagulants.
- Obtain a twelve lead ECG.
- Record the results of physical examinations and vital signs.
- Supervise a 6-minute walk test, recording distance walked and peak heart rate.
- Complete other CRF requirements.
- If an echocardiogram has been performed for clinical reasons since the previous visit, images will be retrieved and sent to the core echocardiographic laboratory.
- Complete the end of trial standardised letter to the GP and clinician explaining that the participant has reached the end of the trial protocol and is no longer bound by their allocated medication strategy. Advise that all participants are invited for continued follow up and long term clinical outcome assessment.
- Provide final instructions to participant (e.g. follow-up of ongoing adverse events).

8.5 Investigator-blinded Endpoints

Investigator-blinded endpoints (PROMs, echocardiography and biomarkers) will be assessed by the core laboratory, identified only by the trial number. Ambulatory ECG and serum digoxin level will remain unblinded and results delivered to the responsible clinician.

8.6 Long Term Follow-Up

In patients who have agreed to NHS data linkage, a follow-up CRF will be completed. The CRF will capture items that include, but are not limited to death, hospital admissions and

For peer review only - http://bmjopen.bmj.com/site/about/guidelines.xhtml

cardiovascular events. The planned interval for outcome assessment is 2 and 5 years post-enrolment.

8.7 Withdrawal

Participants may withdraw at any time during the main **RATE-AF** trial if they choose not to continue or if their clinical team feel that continued participation in the trial is inappropriate.

An investigator may deem it necessary to withdraw a participant from the trial if:

1) Any clinical adverse event, laboratory abnormality, or other medical condition or situation occurs such that continued participation in the trial would not be in the best interest of the participant.
2) The participant meets an exclusion criterion (either newly developed or not previously recognised) that precludes further trial participation.

Full details of the reason(s) for withdrawal should be recorded on the Case Report Forms (CRFs) if healthcare professional-initiated, otherwise a simple statement reflecting patient preference will suffice.

Clear distinction will be made between withdrawals from trial treatments whilst allowing further follow-up, and any participants who refuse any follow-up. If a participant explicitly withdraws consent to any further data recording, then this decision will be respected. All communications surrounding the withdrawal will be noted in the participant's records and no further data will be collected for the participant.

In the case of missing echocardiographic outcome data due to withdrawal (but with consent for ongoing follow-up) or death, results of recent clinical echocardiography will be retrieved. The participant's permission to obtain such data will be obtained and documented during the consent process. As with all trial echocardiograms, the scan will be reported by the core echocardiographic laboratory. With respect to patient-reported outcomes, QoL questionnaires will be mailed to participants who withdraw from trial treatment but consent to ongoing follow up. Those patients where adverse symptoms were related to withdrawal will be invited to a focus group for further discussion.

8.8 Trial Duration

Patients will be on trial medication for 12 months and will be followed-up, during this period according to the protocol. At the end of the 12 months, the participants may, as determined by their clinician, continue on medication but it will not be considered part of the trial intervention. The trial will cease when the 12-month follow-up has been completed for the last participant recruited.

For peer review only - http://bmjopen.bmj.com/site/about/guidelines.xhtml

Table 1: Schedule of Assessments

Procedures		Baseline Visit	Up-titration Visits (Day 14 to 60)	Visit 2, Month 6 (± 4 weeks)	Visit 3, Month 12 (± 4 weeks)
Assessment of eligibility criteria		X			
Informed consent taken		X			
Review of medical history		X			
Review of medications		X	X	X	X
Physical exam	Complete	X			
	Symptom-directed		X	X	X
	Vital signs	X	X	X	X
Quality of life assessment		X	(X)	X	X
Functional and cognitive assessment		X		X	X
Transthoracic echocardiogram		X			X
12-lead electrocardiogram		X		X	X
6-minute walk test		X		X	X
24-hour ambulatory ECG			X	(X)	
Clinical labs	Chemistry	X		X	X
	Haematology	X		X	X
	Serum digoxin			(X)	(X)
Trial labs	BNP	X		X	
	Stored sample	X		X	
Assessment of compliance			X	X	X
Assessment of adverse events			X	X	X

Parentheses denote where a procedure is dependent on the stage of participants within the trial.

For peer review only - http://bmjopen.bmj.com/site/about/guidelines.xhtml

9 Trial Procedures

9.1 Procedures Defined as Standard Clinical Care

The following assessments are considered part of the standard clinical care of AF patients receiving heart rate control therapy and will occur at all trial visits:

- Blood tests for haemoglobin, serum creatinine, potassium and serum digoxin concentration; these will be obtained by the research nurse and submitted to the site-specific hospital laboratory as per local guidelines and SOPs, ensuring that all specimens are accurately labelled and handled appropriately. In the case of results requiring urgent action, local policies will be followed which may include the participant visiting their GP, local hospital or investigator. In all cases, appropriate trial documentation will be completed.
- A 12-lead ECG; these will be completed by appropriately trained local staff.

9.2 Medical History

Medical history will be obtained by interview and from medical records (physical and electronic) at the Baseline Visit comprising:

- Cardiovascular history, including prior ischaemic coronary disease, interventions and surgery, history of hypertension, heart failure or hyperlipidaemia, stroke or transient ischaemic attack, pulmonary embolus/deep vein thrombosis and peripheral vascular disease.
- AF history, including year of diagnosis, previous cardioversions, previous ablation therapy and anti-arrhythmic drug history.
- Pacemaker history, including date and reason for implantation, type of device (single-chamber, dual-chamber, biventricular, implanted defibrillator) and dependency.
- Non-cardiac history, including diabetes mellitus, airways disease (asthma/COPD), renal impairment, bleeding history and other major co-morbidities.
- Social and demographic history, including smoking status (current/ex/never), race (Caucasian/Indian subcontinent/Asian/African/other) and physical activity using the International Physical Activity Questionnaire (short form).

9.3 Medication History

Medications history will be assessed according to the categories below and include current dosage. Except for anticoagulation, antiarrhythmic and rate control therapies, only current medications will be included.

- Anticoagulation therapy (vitamin-K antagonists and novel agents), including past use, INR results and time in therapeutic range.
- Antiarrhythmic therapy, including past use.

For peer review only - http://bmjopen.bmj.com/site/about/guidelines.xhtml

- Rate control therapy (beta-blockers, digoxin, CCB), including past use.
- Antiplatelet therapy.
- Angiotensin-converting enzyme inhibitors or angiotensin receptor blockers.
- Aldosterone antagonists.
- Diuretics (loop, thiazide, potassium-sparing, others).
- Nitrates.
- Other anti-hypertensive/anti-anginal therapy.
- Statins.
- Other lipid-lowering medication.
- Diabetic medication and insulin.
- Asthma/COPD medication (including inhalers).
- Non-steroidal anti-inflammatory agents.

9.4 Physical Examination

Physical and vital signs will be assessed at all up-titration and trial visits. In most cases, a targeted physical examination will be performed, comprising of cardiovascular elements as summarised below:

- Heart rate (manual palpation at radial artery and apex).
- Heart sounds.
- Lung auscultation.
- Assessment of jugular venous pressure and/or peripheral oedema.
- Other focused examinations according to symptoms and complaints.
- Blood pressure (two measurements at the right brachial in a seated position preferred, using a validated oscillometric device).
- Height (Baseline Visit only), weight (all listed visits) and waist circumference (Baseline Visit; defined as the narrowest point between ribs and hips when viewed from the front after exhaling to the nearest centimetre).

9.5 Patient Reported Outcomes

9.5.1 Choice of Outcomes and Qualitative Research

A systematic review (according to and in collaboration with the COnsensus-based Standards for the selection of health Measurement Instruments, COSMIN[74]) is underway to evaluate PROMs in AF, with a focus on psychometric properties including internal consistency, reliability, and measurement error. Additional assessment and practical evaluation of PROMs will follow published guidance[75, 76], complementing qualitative research using patient focus groups, surveys

For peer review only - http://bmjopen.bmj.com/site/about/guidelines.xhtml

and directed interviews guided by the PROMs and qualitative research centres at the University of Birmingham.[77]

Instruments for assessment will be selected on the basis of overall validity, preferably in this patient population but including other groups where data are limited. Patient focus groups will allow exploration of patient perspectives on appropriate instruments that adequately reflect the experience of living with AF.[78] They will also allow comparison of QoL questionnaires that adequately summarise patient-prioritised components of their health and well-being. Additional focus groups and individual interviews will occur at interim and final follow-up during the trial. These aim to understand the patient experience of trial participation and processes, including the ease of completion of QoL questionnaires, relevance, reasons for non-completion and other feasibility issues that emerge during the trial e.g. non-compliance and recruitment, with reference to core outcome sets for this population.[79] A patient and public involvement (PPI) panel will contribute to all stages in the focus group process.[80]

This protocol was developed in accordance with the Standard Protocol Items for Randomized Trials [SPIRIT] statement[81], and the latest PROM-specific guidance from the International Society for Quality of Life Research (ISOQOL) Best Practice taskforce.[77, 82, 83]

9.5.2 Data Collection for PROMs

PROMs will be assessed at all main visits (Baseline, 2 and 3) and at the participants final up-titration visit (if applicable). The QoL tools used will be EQ-5D-5L, SF-36 and AFEQT. To avoid introducing co-intervention bias, all QoL data will be kept confidential and will not be used to inform clinical care.[84] Patients will be advised of this in the patient information sheet. PROMs will be collected at the start of each visit, before other trial procedures. In cases where the visit coincides with a clinician review, questionnaires should be completed in advance. The feasibility of using an online data collection tool will be explored, administered by trained research nurses and according to good-practice guidelines.[85] We will use this trial to perform an initial small-group assessment of electronic PROMs-equivalence to inform a future clinical event trial.

Qualitative research will be performed using a focus group of 10 volunteer patients enrolled at the start of the trial (5 in each randomised group). The focus group will meet after up-titration and then at 6 and 12 months. Detailed methods will be established before the first meeting, in collaboration with the University of Birmingham Qualitative Research Group.

All staff will receive training in QoL collection, with specific guidance on reducing introduced bias, minimising missing data and coaching participants to use the QoL software. Levels of missing PROMs data will be monitored. The site personnel responsible for collection of patient reported outcomes will be the Research Nurse under the supervision of the Principal Investigator.

For peer review only - http://bmjopen.bmj.com/site/about/guidelines.xhtml

9.5.3 Outcome Appraisal

Each QoL tool will be scored according to their published requirements (www.euroqol.org; www.sf-36.org; www.afeqt.org), using total and sub-category scores where appropriate.

To avoid dilution of effect over time, the primary analysis will be at six months (adjusting for baseline QOL and stratification variables). We have predefined a focus on physical well-being, which we hypothesize are where the greatest treatment effects will be observed, but will explore all aspects of QoL. Exploratory analysis of medication effects over the 12-month period will also be analysed and remain clinically important, as little data currently exists on the longer-term profile of QoL in AF.

Qualitative research outcomes will focus on the clinical responsiveness of the QoL instruments. The findings of the COSMIN systematic report will determine these outcomes and their relevant appraisal.

The RATE-AF trial will allow us to gain an initial understanding and framework of the patient experience of AF. We aim to begin the process of determining appropriate and responsive PROMs for AF patients and the optimum methods for delivery into a subsequent large-scale clinical trial.

9.6 Transthoracic Echocardiography

Echocardiography will be performed at Visits 1 and 3 and focus on systolic left-ventricular (LV) function, diastolic function and left-atrial assessment. Images will be obtained by an accredited echocardiographer. All trial echocardiograms will be labelled with the Trial Number and pseudoanonymised patient data, with specific instruction that the echocardiographer will remain blinded to the treatment assignment. All images will be archived to the core echocardiographic laboratory, with a copy retained in the site file.

9.6.1 Reproducibility and Validity of Measurements

Inter-observer and intra-observer variability in measurement will be assessed by comparing results of the stated methods discussed below across the cardiac cycle. To evaluate the minimum number of repeat measurements required that maintains clinical utility, reproducibility of single measurements will be compared to averages of 3/5/10 beats. This will also include the reliability of using an 'index beat' with a cycle length equivalent to a heart rate of 70-80 beats per minute, or with similar preceding and pre-preceding RR intervals.

9.6.2 Systolic LV Function

Systolic LV function will be determined by the following methods:

- Two-dimensional biplane Simpson's method utilising the simultaneous multi-planar approach to obtain LVEF in a single heartbeat (four and two-chamber views). In each

For peer review only - http://bmjopen.bmj.com/site/about/guidelines.xhtml

view, LV end-diastolic and end-systolic volumes (LVEDV, LVESV) are computed, with LVEF calculated as (LVEDV – LVESV) / LVEDV. Two-dimensional echocardiography has excellent spatial resolution but is limited by potential foreshortening of the ventricular apex and drop-out of the endocardial border.

- Standard Simpson's biplane method with four and two-chamber volumes obtained from separate heartbeats. This is the conventional method in current clinical use but is limited by varying RR intervals in AF.
- Fractional shortening on M-mode along the minor-axis of the left-ventricle (parasternal long-axis), calculated by the formula: (LV internal dimension in diastole - LV internal dimension in systole) / LV internal dimension in diastole. M-mode measurements are reproducible and easy to perform with excellent temporal resolution, but are limited in cases of regional wall motion abnormalities and in patients where the true minor-axis is difficult to visualise.
- Both automated endocardial tracking and speckle-tracking analysis will be utilised (where available) by the echocardiographic core laboratory. Multiple planes will be obtained (four-chamber, two-chamber and short-axis mid-ventricle views). These methods have the advantage of reducing operator time and are angle-independent, but rely on good ultrasound windowing. Global longitudinal systolic strain using 2D speckle-tracking has recently been proposed as an important marker for adverse cardiovascular outcomes in AF.[86]
- Three-dimensional full-volume analyses of LV function, with single-beat analysis where feasible. This method has the advantage of not relying on geometric assumptions and allows the acquisition of full volume data within a single heartbeat. It correlates well with gold standard methods such as cardiac magnetic resonance imaging, but relies on adequate ultrasound windowing.
- Peak S-wave on tissue Doppler imaging (TDI) of the mitral valve annular sub-endocardium. This method has good correlation with LVEF across a wide range of function and is obtainable in patients with poor acoustic windows, but is limited in cases of regional wall motion abnormality.

Where poor quality acoustic windows limit accurate assessment of LV function, use of an intravenous contrast agent is recommended in participants without known allergy.

9.6.3 Diastolic LV Function

Diastolic LV function will be determined using the following methods (in all cases repeated over 3-5 cardiac cycles):

- Mitral inflow pulse-wave Doppler peak E velocity and deceleration time (DT).
- Mitral annular TDI to calculate septal E', lateral E' and the individual and averaged E/E' ratios.
- LV outflow tract pulse-wave Doppler to calculate isovolumic relaxation time (IVRT).

For peer review only - http://bmjopen.bmj.com/site/about/guidelines.xhtml

- Pulmonary vein pulse-wave Doppler to calculate peak systolic (where present) and diastolic velocities, ratio of peak velocities and DT of diastolic PV flow.
- Colour M-mode Doppler assessment of mitral flow propagation velocity (Vp) and ratio of E/Vp.

Overall diastolic function will be categorised according to the British Society of Echocardiography guidelines into normal function or mild/moderate/severe dysfunction based on a combination of the above variables. Individual parameters will also be categorised using cut-points identified from published studies.[87]

9.6.4 Left Atrial Size and Function

Left atrial (LA) size will be measured in the anteroposterior (parasternal long-axis), transverse and longitudinal dimensions (apical 4-chamber). LA volumes will be calculated using the biplane area-length method: (0.85 x 4-chamber LA area x 2-chamber LA area) / LA length. The length is measured from the middle of the plane of the mitral annulus to the superior aspect of the LA (shortest of 4- and 2-chamber measurements). LA volumes will be indexed for body surface area.

Where suitable datasets are obtained, 3D LA volumetric analysis and assessment of LA function and strain will also be performed.

9.6.5 Additional Echocardiography Parameters

The following parameters will be obtained in all participants:

- Tricuspid annular plane systolic excursion (TAPSE) for estimation of right ventricular function using pulse-wave Doppler.
- Where applicable, mitral regurgitation dP/dt.

9.7 Laboratory Evaluations

The use of biomarkers than can affect treatment decisions in AF is at an early stage of development.[88] The RATE-AF trial will allow us to collect and store blood samples on patients at baseline and follow-up, providing a unique biobank of AF patients receiving rate-control. In collaboration with the Human Biomaterials Resource Centre (HBRC) at the University of Birmingham, we will also isolate DNA for future work on predictors of response, including known polymorphisms of rate-responsiveness.[89]

Laboratories at each clinical site will process the standard laboratory investigations required as part of standard clinical care (see **Section 9.1**). Trial laboratory evaluations will be performed at the core laboratory and processed according to the guidelines in **Sections 9.7.1**, **9.7.2** and **9.7.3**.

For peer review only - http://bmjopen.bmj.com/site/about/guidelines.xhtml

9.7.1 Laboratory Assays

NT-pro B-type natriuretic peptide will be analysed using a Sandwich immunoassay using monoclonal ruthenium labelled antibody and Roche Cobas 8000 e602. The total coefficient of variation for repeatability with this assay is <2% with an estimated volume of 250 microlitres required for each test and measurement range of 5-35000 pg/mL (0.6-4130 pmol/L).

9.7.2 Cellular Response to Rate Control

The effect of baseline and follow-up serum on intracellular sodium/calcium, force of contraction and activation of ERK1/2-dependent cascades will be examined in human induced pluripotent stem cell-derived cardiomyocytes, using well-established integrated fluorescence/contractility photometry and western blotting techniques.[90, 91] DigiFAB[92], will be used to determine whether changes are dependent on endogenous cardiotonic steroids, which can modulate intracellular ion concentration in cardiomyocytes[93, 94], and potentially contribute to treatment discontinuation (or the development of toxicity).[95] The concentration of serum cardiotonic steroids will be determined using liquid chromatography–tandem mass spectrometry. Individual change in cardiotonic steroids and intracellular sodium/calcium will be correlated with the change in heart rate, LVEF, B-type natriuretic peptide and quality of life. In addition, we will identify patterns in patients withdrawing from treatment or experiencing adverse reactions.

9.7.3 Stored Blood Samples

Blood samples will be stored at HBRC for future biomarker and genetic analysis, with participants providing explicit consent for this process. Any future use of these samples will be undertaken with ethical approval.

9.7.4 Specimen Preparation, Handling, Storage and Shipment

Specimens will be handled according to local standard operating procedures consisting of the time requirements for processing, required temperatures, aliquots of specimens, where they will be stored, how they will be labelled, the process for remnant samples/disposal and appropriate instructions for transportation.

9.8 Economic Evaluation

The RATE-AF trial will allow determination of the most appropriate data collection methods and ease of acquiring resource use and cost data for a subsequent outcomes trial. Specifically, how data is obtained from secondary care records, patient-reported resource use and the feasibility of obtaining primary care records. A preliminary economic evaluation from an NHS perspective will be performed to estimate costs over the 12-month period. The patient-level cost-analysis will determine all AF-related costs, with respect to trial interventions and secondary-care resource use (including adverse events) in the two arms of the trial. We will collect both cost and outcome data and present them in a cost-consequence analysis. Costing for this trial suggests that simplifying medication alone could result in a saving of £5900 over each 12-month period.

For peer review only - http://bmjopen.bmj.com/site/about/guidelines.xhtml

Considering the high and increasing prevalence of AF, this could result in a substantial NHS cost savings, particularly if adverse reactions are also reduced. The aim of this objective within the trial is to prepare the groundwork for a future cost-per-quality adjusted life year (QALY) analysis of rate-control in AF.

Costs of care will be derived from patient level resource-use data, focusing on secondary care costs, and including adverse effects, such as pacemaker implantation. Other major drivers of cost are hospitalisation (including visits to Accident & Emergency), unplanned outpatient visits and outpatient tests such as echocardiography or ambulatory ECG. The cost analysis will also consider therapy costs, both trial drug and additional treatments. Unit costs will be obtained from standard sources including NHS Reference Costs, Unit Costs of Health and Social Care[96] and health care providers. Total per-patient health care costs will initially be calculated thus allowing the estimation of mean costs per trial arm over 12 months follow-up. Responses to the EQ-5D-5L questionnaire at baseline, visit 2 (6 months) and visit 3 (12 months) will be used to plan a future QALY analysis.

Key feasibility elements are:

- Determining the best methods for obtaining hospitalisation data, including where participants have been hospitalised outside of research site
- Whether robust primary care costs can be estimated and the method(s) for acquiring this type of data
- How key cost drivers can be incorporated into data collection for any future trial

For peer review only - http://bmjopen.bmj.com/site/about/guidelines.xhtml

10 Pharmacovigilance

Definitions of different types of AE are listed in **Table 2**. The Investigator should assess the seriousness and causality (relatedness) of AEs experienced by the participant (this should be documented in the source data). For further information please refer to **Section 10.1**.

Table 2: Standard AE Definitions

Term	Definition
**Adverse Event (AE)**	Any untoward medical occurrence in a patient or clinical trial participant administered a medicinal product and which does not necessarily have a causal relationship with this treatment
**Adverse Reaction (AR)**	Any untoward and unintended response in a participant to an investigational medicinal product which is related to any dose administered to that participant
**Serious adverse event (SAE), serious adverse reaction (SAR) or unexpected serious adverse reaction**	Any adverse event, adverse reaction or unexpected adverse reaction, respectively, that:<br>• results in death;<br>• is life-threatening;<br>• requires hospitalisation or prolongation of existing hospitalisation;<br>• results in persistent or significant disability or incapacity; or<br>• consists of a congenital anomaly or birth defect
**Unexpected Adverse Reaction**	An adverse reaction the nature and severity of which is not consistent with the information about the medicinal product in question set out:<br>(a) in the case of a product with a marketing authorisation, in the summary of product characteristics for that product;<br>(b) in the case of any other investigational medicinal product, in the investigator's brochure relating to the trial in question.
**SUSAR**	Suspected Unexpected Serious Adverse Reaction

10.1 Recording and Assessment of Adverse Events

All adverse events will be reportable to the **RATE-AF** Trial Office up to 30 days post last IMP administration. Any SUSAR related to the IMP should to be reported irrespective of how long after IMP administration the reaction has occurred.

Adverse events will be recorded in the medical records and CRFs. Most AE/ARs that occur in this trial, whether they are serious or not, will be 'expected' treatment-related toxicities due to the drugs used in this trial.

For peer review only - http://bmjopen.bmj.com/site/about/guidelines.xhtml

Refer to **Table 3** for definition of expectedness.

Table 3: Expectedness

Category	Definition
**Expected**	An adverse event that is classed in nature as serious and which is consistent with the information about the IMP listed in the Investigator Brochure (or SmPC if Licensed IMP) or clearly defined in this protocol
**Unexpected**	An adverse event that is classed in nature as serious and which is not consistent with the information about the IMP listed in the Investigator Brochure (or SmPC if Licensed IMP)

Adverse events will be recorded with clinical symptoms and accompanied with a simple, brief description of the event, including dates as appropriate.

The assessment of relationship of adverse events to the administration of IMP is a clinical decision based on all available information at the time. The following categories as outlined in **Table 4** will be used to define the causality of the adverse event.

Table 4: Categorisation of Causality

Category	Definition
**Definitely**	There is clear evidence to suggest a causal relationship, and other possible contributing factors can be ruled out
**Probably**	There is evidence to suggest a causal relationship, and the influence of other factors is unlikely
**Possibly**	There is some evidence to suggest a causal relationship (e.g. the event occurred within a reasonable time after administration of the trial medication). However, the influence of other factors may have contributed to the event (e.g. the patient's clinical condition, other concomitant events)
**Unlikely**	There is little evidence to suggest there is a causal relationship (e.g. the event did not occur within a reasonable time after administration of the trial medication). There is another reasonable explanation for the event (e.g. the patient's clinical condition, other concomitant treatments)
**Not related**	There is no evidence of any causal relationship

The relevant SmPC for Digoxin and Bisoprolol (which will be dependent on which generic is being used according to local practice at each site) should be filed in the site file by the local research team.

For peer review only - http://bmjopen.bmj.com/site/about/guidelines.xhtml

The **RATE-AF** Trial protocol and the reference safety information will be used to assess disease related and/or expected events related to the trial treatment.

10.2 Non-Serious Adverse Events/ Adverse Reactions

Refer to Table 2 for definitions

Common adverse reactions (see Section 7.4) will be recorded on the relevant CRF and sent to the RATE-AF Trial Office.

10.3 Serious Adverse Events

Refer to Table 2 for definitions

All Serious Adverse Events (SAEs), that are not excluded from expedited reporting will be recorded in the hospital notes and should be reported to the **RATE-AF** Trial Office on a SAE Form. The completed form should be **faxed to the RATE-AF Trial Office on 0121 415 9135 or 0121 415 9136**, as soon as possible and ideally within one working day of becoming aware of the event. The site Investigator should be able to respond to any related queries raised by the **RATE-AF** Trial Office as soon as possible.

10.3.1 Expected SAEs NOT to be Reported on a SAE Form

Expected SAEs are those listed in the current SmPC for the trial IMPs and can be excluded from the expedited reporting outlined in **Section 10.1**, for example if they are expected to occur on a regular basis and offer no further new information to the safety profile. These events should continue to be recorded in the source data and relevant CRFs.

In addition, events **NOT** considered to be SAEs are hospitalisations for:

- Routine treatment or monitoring of the studied indication, not associated with any deterioration in condition
- Treatment, which was elective or pre-planned, for a pre-existing condition that is unrelated to the indication under trial, and has not worsened

Note: Death from any cause should be reported on an SAE Form and returned to the **RATE-AF** Trial Office.

10.4 SUSARs

Refer to Table 2 for definitions

SAEs classed by as both suspected to be related to the trial IMP and unexpected are categorised as SUSARs, and are always subject to expedited reporting. An SAE Form should be completed,

For peer review only - http://bmjopen.bmj.com/site/about/guidelines.xhtml

and faxed to the RATE-AF Trial Office within 24 hours of the research staff at site becoming aware of the event. The local investigator will provide the causality assessment.

The Chief Investigator (or nominated individual) will undertake urgent review of all such SAEs and may request further information immediately from the clinical team at site. The Chief Investigator will not overrule the causality or seriousness assessment given by the local investigator but may add additional comment on these. The Chief Investigator will provide the determination of expectedness according to the reference safety information.

SUSARs will be notified to the MHRA and REC by the RATE-AF Trial Office. SUSARs that are fatal or life-threatening will be notified to the MHRA and REC within 7 days after the **RATE-AF** Trial Office has been notified. Other SUSARs will be reported to the REC and MHRA within 15 days after the Trial Office has been notified.

10.5 Development Safety Update Reports

The **RATE-AF** Trial Office will provide the MHRA with Development Safety Update Reports (DSURs). The reports will be submitted within 60 days of the Developmental International Birth Date (DIBD) of the trial each year until the trial is declared ended.

10.6 Annual Progress Reports

An annual progress report will be submitted to the REC within 30 days of the anniversary date on which the favourable opinion was given, and annually until the trial is declared ended.

10.7 Pregnancy

Due to the age of participants that will be randomised into the **RATE-AF** Trial (≥ 60 years), it is highly improbable that female participants will be pregnant at the time of randomisation, or become pregnant during the trial. Any pregnancies will be followed up for outcome; any outcome meeting the definition of an SAE will be reported to the **RATE-AF** Trial Office on the relevant CRF.

10.8 Reporting Urgent Safety Measures

If any urgent safety measures are taken the Principal Investigator/BCTU/Sponsor shall immediately and in any event no later than 3 days from the date the measures are taken, give written notice to the MHRA and the REC of the measures taken and the circumstances giving rise to those measures.

For peer review only - http://bmjopen.bmj.com/site/about/guidelines.xhtml

11 Quality Control and Quality Assurance

11.1 Site Set-Up and Initiation

All participating Principal Investigators will be asked to sign the necessary agreements and supply a current CV to the Trials Office. All members of the site research team will also be required to sign a site signature and delegation log. Prior to commencing recruitment all sites will undergo a process of initiation and will have completed GCP training. Key members of the site research team will be required to attend either a meeting or a teleconference covering aspects of the trial design, protocol procedures, Adverse Event reporting, collection and reporting of data and record keeping. Sites will be provided with an Investigator Site File containing essential documentation, instructions, and other documentation required for the conduct of the trial. The Trials Office must be informed immediately of any change in the site research team.

11.2 Central Monitoring

Monitoring of this trial will be to ensure compliance with Good Clinical Practice. A risk proportionate approach to the initiation, management and monitoring of the trial will be adopted (as per the MRC/DH/MHRA Joint Project: Risk-adapted Approaches to the Management of Clinical Trials of Investigational Medicinal Products) and outlined in the trial-specific risk assessment.

The Trials Office will be in regular contact with the site research team to check on progress and address any queries that they may have. The Trials Office will check incoming CRFs for compliance with the protocol, data consistency, missing data and timing. Sites will be asked for missing data or clarification of inconsistencies or discrepancies. Sites will be requested to send in copies of signed Informed Consent Forms and other documentation for in-house review for all participants providing explicit consent.

Additional on-site monitoring visits may be triggered, for example by poor CRF return, poor data quality, low SAE reporting rates, excessive number of participant withdrawals or deviations. This will be detailed in the monitoring plan. If a monitoring visit is required the Trials Office will contact the site to arrange a date for the proposed visit and will provide the site with written confirmation. Investigators will allow the **RATE-AF** trial staff access to source documents as requested.

11.3 Audit and Inspection

The Principal Investigator will permit trial-related monitoring, quality checks, audits, ethical reviews, and regulatory inspection(s) at their site, providing direct access to source data/documents. The Principal Investigator will comply with these visits and any required follow up. Sites are also requested to notify the Trials Office of any MHRA inspections.

For peer review only - http://bmjopen.bmj.com/site/about/guidelines.xhtml

11.4 Notification of Serious Breaches

In accordance with Regulation 29A of the Medicines for Human Use (Clinical Trials) Regulations 2004 and its amendments the Sponsor of the trial is responsible for notifying the licensing authority in writing of any serious breach of the conditions and principles of GCP in connection with that trial or the protocol relating to that trial, within 7 days of becoming aware of that breach.

For the purposes of this regulation, a "serious breach" is a breach which is likely to effect to a significant degree the safety or physical or mental integrity of the participants of the trial; or the scientific value of the trial. Sites are therefore requested to notify the Trials office of any suspected trial-related serious breach of GCP and/or the trial protocol. Where the Trials Office is investigating whether or not a serious breach has occurred sites are also requested to cooperate with the Trials Office in providing sufficient information to report the breach to the MHRA where required and in undertaking any corrective and/or preventive action. Sites may be suspended from further recruitment in the event of serious and persistent non-compliance with the protocol and/or GCP, and/or poor recruitment. Any major problems identified during monitoring may be reported to Trial Management Group and Trial Oversight Committee, the REC and the relevant regulatory bodies. This includes reporting serious breaches of GCP and/or the trial protocol to the REC and MHRA. A copy is sent to the University of Birmingham Clinical Research Compliance Team at the time of reporting to the REC and/or relevant regulatory bodies.

11.5 Data Handling and Analysis

Paper CRFs must be completed, signed/ dated and either entered directly online or returned to the **RATE-AF** Trial Office by the PI or an authorised member of the site research team (as delegated on the **RATE-AF** Trial Signature and Delegation Log) within the timeframe listed in **Table 5**. Copies of all completed CRFs should be filed in the site file. Entries on paper CRFs should be made in ballpoint pen, in black ink, and must be legible. Any errors should be crossed out with a single stroke, the correction inserted and the change initialled and dated. If it is not obvious why a change has been made, an explanation should be written next to the change.

CRFs can be entered online at http://www.bctu.bham.ac.uk/RATEAF. Authorised staff at sites will require an individual secure login username and password to access this online data entry system.

Data reported on each CRF should be consistent with the source data or the discrepancies should be explained. If information is not known, this must be clearly indicated on the CRF. All missing and ambiguous data will be queried. All sections are to be completed.

CRF versions may be updated by the **RATE-AF** Trial Office, as appropriate, throughout the duration of the trial. Whilst this will not constitute a protocol amendment, new versions of the CRFs must be implemented by participating sites immediately on receipt.

For peer review only - http://bmjopen.bmj.com/site/about/guidelines.xhtml

It will be the responsibility of the Principal Investigator to ensure the accuracy of all data entered in the CRFs. The **RATE-AF** Trial Signature & Delegation Log will identify all those personnel with responsibilities for data collection.

Access to data, including the final trial dataset, will be limited to members of the Research Team.

The investigator(s)/institution(s) will permit trial-related monitoring, audits, REC review, and regulatory inspection(s), providing direct access to source data/documents. Trial participants are informed of this during the informed consent discussion and will consent to provide access to their medical notes.

Table 5: Data Collection Forms

Form Name	Schedule for submission
**Randomisation Form**	Collected at randomisation
**Baseline and Follow-Up CRFs**	As soon as possible after each follow-up assessment time point
**Serious Adverse Event Form**	Faxed within 24hrs of research staff at site becoming aware of event

11.6 End of Trial

The end of trial will be 30 days after the last data capture. This will allow sufficient time for the completion of protocol procedures, data collection and data input. The Trials Office will notify the MHRA and REC that the trial has ended within 90 days of the end of trial. Where the trial has terminated early, the Trials Office will inform the MHRA and REC within 15 days of the end of trial. The Trials Office will provide them with a summary of the clinical trial report within 12 months of the end of trial.

A copy of the end of trial notification as well as the summary report is also sent to the University of Birmingham Research Governance Team at the time of sending these are sent to the MHRA and REC.

11.7 Archiving

Archiving will be authorised by the BCTU on behalf of the Sponsor following submission of the end of trial report.

For peer review only - http://bmjopen.bmj.com/site/about/guidelines.xhtml

Principal Investigators are responsible for the secure archiving of essential trial documents (for their site) as per their NHS Trust policy. All essential documents will be archived for a minimum of 25 years after completion of trial.

Destruction of essential documents will require authorisation from the BCTU on behalf of the Sponsor.

12 Statistical Considerations

12.1 Outcome measures

12.1.1 Primary Outcome

Patient-reported quality of life (QoL) - SF-36 physical component summary score at six months

12.1.2 Secondary Outcomes

Patient-reported QoL:

- SF-36 global and domain-specific scores at 6 and 12 months
- EQ-5D-5L summary index and visual analogue scale at six and twelve months
- AFEQT overall score at six and twelve months

Cardiac function:

- Echocardiographic LVEF at 12 months
- Diastolic function (E/e' and composite of diastolic indices) at 12 months
- Functional assessment:
- Six-minute walking distance at 6 and 12 months
- Change in European Heart Rhythm Association (EHRA) class at 6 and 12 months

Biomarkers:

- Change in B-type natriuretic peptide (BNP) levels at 6 months
- Change in heart rate using 24-hour ambulatory ECG

12.1.3 Feasibility Outcomes

- Recruitment target of 3 patients per week across all participating centres.
- Compliance and reasons for non-compliance
- Number of withdrawals and losses to follow-up (with reasons)
- Drug discontinuation rate and adverse reactions requiring drug discontinuation.
- Number of patients needing therapy-induced requirement for additional treatment
- Population-specific standard deviations (SD) and proportions

For peer review only - http://bmjopen.bmj.com/site/about/guidelines.xhtml

- *SD of SF36 physical functioning score at 6 and 12 months*
- *SD of SF36 overall score at 6 and 12 months*
- *SD of AFEQT overall score at 6 and 12 months*
- *SD of LVEF and E/e' score at 6 and 12 months*
- *Unplanned hospitalisation admissions rates*

- Cardiovascular Events (particularly mortality, thromboembolic events, myocardial infarction and cardiovascular interventions)

The final analyses will follow a pre-specified analysis plan, drafted in conjunction with the Birmingham Clinical Trials Unit and submitted to the steering committee at the penultimate meeting. We intend to perform a primary intention-to-treat analysis, in addition to a per-protocol analysis.

Any additional (exploratory) analyses will be explicitly labelled as such in any subsequent manuscript.

12.2 Power Calculations

Randomising 144 patients we can assume an 85% power to detect an effect size of half a standard deviation in a continuous outcome measure of QoL (two-sided alpha of 0.05). A sample size of 160 patients would account for an estimated 10% loss to follow-up (including withdrawal and death prior to 12-month assessment). There is some evidence from existing research to support the notion that the treatment effect could be this large. The mean SF-36 role-physical score from the rate-control arm of the RACE study was 47, with a 17% improvement with rate-control over time.[62] In another study, CCB resulted in 22% improvement in a proprietary symptom-checklist, compared to a non-significant 8% change in those assigned to beta-blockers (SD 10-points in both groups). These values are also consistent with a 17% improvement in SF-36 scores in a third trial, PIAF.[63] Thus whilst it is possible that this trial may provide a clear indication of effect, it is accepted that the trial will be underpowered to detect smaller differences, reinforcing the requirement for a larger definitive trial which would also be powered to assess impact on clinical event rates.

12.3 Statistical analysis

A separate Statistical Analysis Plan for the RATE-AF trial provides a detailed description of the planned statistical analyses. A brief outline of these analyses is given below.

The primary comparison groups will be composed of those who are randomised to digoxin group and those randomised to the beta-blockers group. All analyses will be based on the intention to treat principle, with all patients analysed in the arms to which they were allocated irrespective of compliance with the randomised allocated treatment, and all patients will be included in the analyses. We will, as a sensitivity analysis, conduct per-protocol analyses, where appropriate.

For peer review only - http://bmjopen.bmj.com/site/about/guidelines.xhtml

For all analyses, a p-value <0.05 will be considered statistically significant.

12.3.1 Primary outcome analysis

The primary outcome for this trial is the continuous SF36 physical functioning domain score at 6 months. This outcome will be analysed using an ANCOVA model adjusting for treatment arm, baseline score and all minimisation variables. Results will be presented as difference in means and 95% confidence intervals.

12.3.2 Feasibility and Secondary outcomes analysis

The feasibility and secondary outcomes for the trial comprise of a combination of both continuous and categorical (dichotomous) data items.

Categorical endpoints:

For outcomes which are categorical (dichotomous) in nature, the proportion of participants and percentages will be analysed between arms.

Logistic/Log-binomial regression models will be fitted (where appropriate) to adjust for treatment arm, baseline scores and all minimisation variables.

Results will be presented as odds ratios/relative risks and 95% confidence intervals.

Continuous endpoints:

Any outcomes that are continuous in nature will be analysed in the same way as the primary outcome.

12.3.3 Missing data and sensitivity analyses

Primary analysis will concentrate on available data only, with no attempt made to impute missing data. Where appropriate, sensitivity analyses will be carried out to examine the possible impact of missing data on the results (full details will be discussed within the Statistical Analysis Plan).

12.3.4 Interim analyses and Stopping rules

Analysis of the data with respect to efficacy and safety will be performed at 12 months and sent to Data Monitoring Committee (DMC); see **Section 16**. The DMC will outline and agree the stopping rules for the trial which will be documented in the DMC charter. It is likely that the Haybittle-Peto boundary will be used. This states that if an interim analysis shows a probability of less than 0.001 that the treatments are different, then the trial should be stopped early. This will be used alongside data on important secondary endpoints and all other relevant evidence. A DMC report and charter outlining the terms of reference (including information on stopping rules) will be agreed with the DMEC.

For peer review only - http://bmjopen.bmj.com/site/about/guidelines.xhtml

12.4 Final analysis

The final analysis for the RATE-AF trial will occur once the last randomised participant completes their 12-month follow-up.

13 Ethics and Regulatory Requirements

The trial will be performed in accordance with the recommendations guiding physicians in biomedical research involving human participants, adopted by the 18th World Medical Association General Assembly, Helsinki, Finland, June 1964, amended at the 48th World Medical Association General Assembly, Somerset West, Republic of South Africa, October 1996 (website: http://www.wma.net/en/30publications/10policies/b3/index.html).

The trial will be conducted in accordance with the Research Governance Framework for Health and Social Care, the applicable UK Statutory Instruments, (which include the Medicines for Human Use Clinical Trials 2004 and subsequent amendments and the Data Protection Act 1998 and Human Tissue Act 2008, EU Clinical Trials Directive and amendment Regulations as appropriate) and Guidelines for Good Clinical Practice (GCP). This trial will be carried out under a Clinical Trial Authorisation in accordance with the Medicines for Human Use Clinical Trials regulations. The protocol will be submitted to and approved by the REC prior to circulation.

Before any participants are enrolled into the trial, the Principal Investigator at each site is required to obtain local R&D approval. Sites will not be permitted to enrol participants until written confirmation of R&D approval is received by the Principal Investigator.

It is the responsibility of the Principal Investigator to ensure that all subsequent amendments gain the necessary local approval. This does not affect the individual clinicians' responsibility to take immediate action if thought necessary to protect the health and interest of individual participants.

Within 90 days after the end of the trial, the Chief Investigator/Sponsor will ensure that the REC and the MHRA are notified that the trial has finished. If the trial is terminated prematurely, those reports will be made within 15 days after the end of the trial. The Chief Investigator will provide the Sponsor with a summary report of the clinical trial, which will then be submitted to the MHRA and REC within one year after the end of the trial.

14 Oversight Committees

14.1 Trial Management Group

The Trial Management Group (TMG) will comprise the CI, other lead investigators (clinical and non-clinical) and members of the BCTU. The TMG will be responsible for the day-to-day running and management of **RATE-AF**. The TMG will convene at regular intervals.

For peer review only - http://bmjopen.bmj.com/site/about/guidelines.xhtml

14.2 Trial Oversight Committee

A joint oversight committee comprising a Trial Steering Committee (TSC) and Data Monitoring Committee (DMC) will be engaged for this trial.

The role of the TSC is to provide the overall supervision of the trial. The TSC will monitor trial progress and conduct and advice on scientific credibility. The TSC will consider and act, as appropriate, upon the recommendations of the Data Monitoring Committee. Further details of the remit and role of the TSC are available in the TSC Charter.

An independent DMC will be established to oversee the safety of participants in the trial. The DMC will meet prior to the trial opening to enrolment, and then meet at least annually, or as per a timetable agreed by the DMC prior to trial commencement. Data analyses will be supplied in confidence to the DMC, which will be asked to give advice on whether the accumulated data from the trial, together with the results from other relevant research, justifies the continuing recruitment of further participants. The DMC will operate in accordance with the trial specific charter.

14.3 Protocol amendments

Where important protocol modifications are required as a result of oversight (for example, changes to eligibility criteria, outcomes or analyses), this information will be communicated to relevant parties, such as investigators, the REC, trial registries and regulators.

15 Finance

The **RATE-AF** Trial is funded through a Career Development Fellowship awarded to the Chief Investigator by the National Institute for Health Research (NIHR).

16 Confidentiality and Data Protection

Personal data recorded on all documents will be regarded as strictly confidential and will be handled and stored in accordance with the Data Protection Act 1998.

Participants will be identified using their unique trial identification number, date of birth and hospital number on the CRFs. and correspondence between the Trials Office and the participating site. Participants will give their explicit consent for the movement of their consent form, giving permission for the Trials Office to be sent a copy. This will be used to perform in-house monitoring of the consent process.

The Investigator must maintain documents not for submission to the Trials Office (e.g. Participant Identification Logs) in strict confidence. In the case of specific issues and/or queries from the

For peer review only - http://bmjopen.bmj.com/site/about/guidelines.xhtml

regulatory authorities, it will be necessary to have access to the complete trial records, provided that participant confidentiality is protected.

The Trials Office will maintain the confidentiality of all participants' data and will not disclose information by which participants may be identified to any third party other than those directly involved in the treatment of the participant and organisations for which the participant has given explicit consent for data transfer (e.g. competent authority, sponsor). Representatives of the **RATE-AF** Trials Office and sponsor may be required to have access to participant's notes for quality assurance purposes but participants should be reassured that their confidentiality will be respected at all times.

17 Insurance and Indemnity

The University of Birmingham has in place Clinical Trials indemnity coverage for this trial which provides cover to the University for harm which comes about through the University's, or its staff's, negligence in relation to the design or management of the trial and may alternatively, and at the University's discretion provide cover for non-negligent harm to participants.

With respect to the conduct of the trial at the Clinical Site and other clinical care of the patient, responsibility for the care of the patients remains with the NHS organisation responsible for the Clinical Site and is therefore indemnified through the NHS Litigation Authority.

The University of Birmingham is independent of any pharmaceutical company, and as such it is not covered by the Association of the British Pharmaceutical Industry (ABPI) guidelines for participant compensation.

18 Dissemination and Publication

Regular newsletters will keep collaborators informed of trial progress and regular meetings will be held to report the progress of the trial and to address any problems encountered in the conduct of the trial. The CI will coordinate dissemination of data from this trial. All publications and presentations, including abstracts, relating to the main trial will be authorised by the **RATE-AF** TMG. The results of the analysis will be published in the name of the **RATE-AF** Collaborative Group in a peer reviewed journal (provided that this does not conflict with the journal's policy).

Named authors must satisfy the International Committee of Medical Journal Editors (ICMJE) criteria for authorship (contribute to drafting of the article or revision for important intellectual content), provide timely approval of the final version to be published and supply detailed statements on any potential conflict of interest or financial relationship (http://www.icmje.org/). Members of the group who do not fulfil ICMJE criteria for authorship will be listed in the article appendix. Trial participants will be sent a lay summary of the final results of the trial, which will contain a reference to the full paper.

For peer review only - http://bmjopen.bmj.com/site/about/guidelines.xhtml

19 Statement of Compliance

The **RATE-AF** trial will be conducted in compliance with the approved protocol, EU GCP and the applicable regulatory requirements.

For peer review only

20 References

1. Camm AJ, Kirchhof P, Lip GY, Schotten U, Savelieva I, Ernst S, Van Gelder IC, Al-Attar N, Hindricks G, Prendergast B, Heidbuchel H, Alfieri O, Angelini A, Atar D, Colonna P, De Caterina R, De Sutter J, Goette A, Gorenek B, Heldal M, Hohloser SH, Kolh P, Le Heuzey JY, Ponikowski P, Rutten FH. Guidelines for the management of atrial fibrillation: the Task Force for the Management of Atrial Fibrillation of the European Society of Cardiology (ESC). *Eur Heart J*. 2010;31:2369-2429

2. Anderson JL, Halperin JL, Albert NM, Bozkurt B, Brindis RG, Curtis LH, DeMets D, Guyton RA, Hochman JS, Kovacs RJ, Ohman EM, Pressler SJ, Sellke FW, Shen W-K. Management of patients with atrial fibrillation (Compilation of 2006 ACCF/AHA/ESC and 2011 ACCF/AHA/HRS recommendations). *Circulation*. 2013;127:1916-1926

3. Camm AJ, Lip GY, De Caterina R, Savelieva I, Atar D, Hohnloser SH, Hindricks G, Kirchhof P, E. S. C. Committee for Practice Guidelines. 2012 focused update of the ESC Guidelines for the management of atrial fibrillation: an update of the 2010 ESC Guidelines for the management of atrial fibrillation. Developed with the special contribution of the European Heart Rhythm Association. *Eur Heart J*. 2012;33:2719-2747

4. National Institute for Health and Care Excellence. Atrial fibrillation: the management of atrial fibrillation. *NICE clinical guideline 180*. 2014; Accessed 15/09/2014; http://www.nice.org.uk/guidance/cg180/

5. Kotecha D, Holmes J, Krum H, Altman DG, Manzano L, Cleland JG, Lip GY, Coats AJ, Andersson B, Kirchhof P, von Lueder TG, Wedel H, Rosano G, Shibata MC, Rigby A, Flather MD, Beta-Blockers in Heart Failure Collaborative Group. Efficacy of beta blockers in patients with heart failure plus atrial fibrillation: an individual-patient data meta-analysis. *Lancet*. 2014;384:2235-2243

6. Ziff OJ, Lane DA, Samra M, Griffith M, Kirchhof P, Lip GY, Steeds RP, Townend J, Kotecha D. Safety and efficacy of digoxin: systematic review and meta-analysis of observational and controlled trial data. *BMJ*. 2015;351:h4451

7. Kotecha D, Banerjee A, Lip GY. Increased stroke risk in atrial fibrillation patients with heart failure: does ejection fraction matter? *Stroke*. 2015;46:608-609

8. Kotecha D, Kirchhof P. Rate and rhythm control have comparable effects on mortality and stroke in atrial fibrillation but better data are needed. *Evid Based Med*. 2014;19:222-223

9. Kotecha D, Piccini JP. Atrial fibrillation in heart failure: what should we do? *Eur Heart J*. 2015;36:3250-3257

10. Senoo K, Lip GY, Lane DA, Buller HR, Kotecha D. Residual risk of stroke and death in anticoagulated patients according to the type of atrial fibrillation: AMADEUS Trial. *Stroke*. 2015;46:2523-2528

11. Kotecha D, Chudasama R, Lane DA, Kirchhof P, Lip GY. Atrial fibrillation and heart failure due to reduced versus preserved ejection fraction: A systematic review and meta-analysis of death and adverse outcomes. *Int J Cardiol*. 2016;203:660-666

12. Arain M, Campbell MJ, Cooper CL, Lancaster GA. What is a pilot or feasibility study? A review of current practice and editorial policy. *BMC Med Res Methodol*. 2010;10:67

13. Thabane L, Ma J, Chu R, Cheng J, Ismaila A, Rios LP, Robson R, Thabane M, Giangregorio L, Goldsmith CH. A tutorial on pilot studies: the what, why and how. *BMC Med Res Methodol*. 2010;10:1

14. Heeringa J, van der Kuip DAM, Hofman A, Kors JA, van Herpen G, Stricker BHC, Stijnen T, Lip GYH, Witteman JCM. Prevalence, incidence and lifetime risk of atrial fibrillation: the Rotterdam study. *Eur Heart J*. 2006;27:949-953

For peer review only - http://bmjopen.bmj.com/site/about/guidelines.xhtml

15. Colilla S, Crow A, Petkun W, Singer DE, Simon T, Liu X. Estimates of Current and Future Incidence and Prevalence of Atrial Fibrillation in the U.S. Adult Population. *Am J Cardiol*. 2013;112:1142-1147

16. Kim MH, Johnston SS, Chu BC, Dalal MR, Schulman KL. Estimation of total incremental health care costs in patients with atrial fibrillation in the United States. *Circ Cardiovasc Qual Outcomes*. 2011;4:313-320

17. Benjamin EJ, Wolf PA, D'Agostino RB, Silbershatz H, Kannel WB, Levy D. Impact of Atrial Fibrillation on the Risk of Death: The Framingham Heart Study. *Circulation*. 1998;98:946-952

18. Stewart S, Hart CL, Hole DJ, McMurray JJV. A population-based study of the long-term risks associated with atrial fibrillation: 20-year follow-up of the Renfrew/Paisley study. *Am J Med*. 2002;113:359-364

19. Marijon E, Le Heuzey JY, Connolly S, Yang S, Pogue J, Brueckmann M, Eikelboom J, Themeles E, Ezekowitz M, Wallentin L, Yusuf S. Causes of death and influencing factors in patients with atrial fibrillation: a competing-risk analysis from the randomized evaluation of long-term anticoagulant therapy study. *Circulation*. 2013;128:2192-2201

20. Chen LY, Sotoodehnia N, Buzkova P, Lopez FL, Yee LM, Heckbert SR, Prineas R, Soliman EZ, Adabag S, Konety S, Folsom AR, Siscovick D, Alonso A. Atrial fibrillation and the risk of sudden cardiac death: the atherosclerosis risk in communities study and cardiovascular health study. *JAMA Intern Med*. 2013;173:29-35

21. Dorian P, Jung W, Newman D, Paquette M, Wood K, Ayers GM, Camm J, Akhtar M, Luderitz B. The impairment of health-related quality of life in patients with intermittent atrial fibrillation: implications for the assessment of investigational therapy. *J Am Coll Cardiol*. 2000;36:1303-1309

22. Thrall G, Lane D, Carroll D, Lip GYH. Quality of Life in Patients with Atrial Fibrillation: A Systematic Review. *Am J Med*. 2006;119:448.e441-419

23. Sears SF, Serber ER, Alvarez LG, Schwartzman DS, Hoyt RH, Ujhelyi MR. Understanding Atrial Symptom Reports: Objective versus Subjective Predictors. *Pacing Clin Electrophysiol*. 2005;28:801-807

24. Dries D, Exner D, Gersh B, Domanski M, Waclawiw M, Stevenson L. Atrial fibrillation is associated with an increased risk for mortality and heart failure progression in patients with asymptomatic and symptomatic left ventricular systolic dysfunction: a retrospective analysis of the SOLVD trials. *J Am Coll Cardiol*. 1998;32:695-703

25. Wang TJ, Larson MG, Levy D, Vasan RS, Leip EP, Wolf PA, D'Agostino RB, Murabito JM, Kannel WB, Benjamin EJ. Temporal Relations of Atrial Fibrillation and Congestive Heart Failure and Their Joint Influence on Mortality: The Framingham Heart Study. *Circulation*. 2003;107:2920-2925

26. The Atrial Fibrillation Follow-up Investigation of Rhythm Management (AFFIRM) Investigators. A Comparison of Rate Control and Rhythm Control in Patients with Atrial Fibrillation. *N Engl J Med*. 2002;347:1825-1833

27. Van Gelder IC, Hagens VE, Bosker HA, Kingma JH, Kamp O, Kingma T, Said SA, Darmanata JI, Timmermans AJM, Tijssen JGP, Crijns HJGM. A Comparison of Rate Control and Rhythm Control in Patients with Recurrent Persistent Atrial Fibrillation. *N Engl J Med*. 2002;347:1834-1840

28. Olshansky B, Rosenfeld LE, Warner AL, Solomon AJ, O'Neill G, Sharma A, Platia E, Feld GK, Akiyama T, Brodsky MA, Greene HL. The Atrial Fibrillation Follow-up Investigation of Rhythm Management (AFFIRM) study: approaches to control rate in atrial fibrillation. *J Am Coll Cardiol*. 2004;43:1201-1208

29. Hohnloser SH, Kuck KH, Lilienthal J. Rhythm or rate control in atrial fibrillation--Pharmacological Intervention in Atrial Fibrillation (PIAF): a randomised trial. *Lancet*. 2000;356:1789-1794

For peer review only - http://bmjopen.bmj.com/site/about/guidelines.xhtml

30. Carlsson J, Miketic S, Windeler J, Cuneo A, Haun S, Micus S, Walter S, Tebbe U, Investigators S. Randomized trial of rate-control versus rhythm-control in persistent atrial fibrillation: the Strategies of Treatment of Atrial Fibrillation (STAF) study. *J Am Coll Cardiol*. 2003;41:1690-1696

31. de Denus S, Sanoski CA, Carlsson J, Opolski G, Spinler SA. Rate vs rhythm control in patients with atrial fibrillation: a meta-analysis. *Arch Intern Med*. 2005;165:258-262

32. Chatterjee S, Sardar P, Lichstein E, Mukherjee D, Aikat S. Pharmacologic rate versus rhythm-control strategies in atrial fibrillation: an updated comprehensive review and meta-analysis. *PACE*. 2013;36:122-133

33. Shelton RJ, Clark AL, Goode K, Rigby AS, Houghton T, Kaye GC, Cleland JG. A randomised, controlled study of rate versus rhythm control in patients with chronic atrial fibrillation and heart failure: (CAFE-II Study). *Heart*. 2009;95:924-930

34. Roy D, Talajic M, Nattel S, Wyse DG, Dorian P, Lee KL, Bourassa MG, Arnold JMO, Buxton AE, Camm AJ, Connolly SJ, Dubuc M, Ducharme A, Guerra PG, Hohnloser SH, Lambert J, Le Heuzey J-Y, O'Hara G, Pedersen OD, Rouleau J-L, Singh BN, Stevenson LW, Stevenson WG, Thibault B, Waldo AL. Rhythm Control versus Rate Control for Atrial Fibrillation and Heart Failure. *N Engl J Med*. 2008;358:2667-2677

35. Kong MH, Shaw LK, O'Connor C, Califf RM, Blazing MA, Al-Khatib SM. Is rhythm-control superior to rate-control in patients with atrial fibrillation and diastolic heart failure? *Ann Noninvasive Electrocardiol*. 2010;15:209-217

36. Wazni O, Wilkoff B, Saliba W. Catheter Ablation for Atrial Fibrillation. *N Engl J Med*. 2011;365:2296-2304

37. Jones DG, Haldar SK, Hussain W, Sharma R, Francis DP, Rahman-Haley SL, McDonagh TA, Underwood SR, Markides V, Wong T. A randomized trial to assess catheter ablation versus rate control in the management of persistent atrial fibrillation in heart failure. *J Am Coll Cardiol*. 2013;61:1894-1903

38. Kirchhof P, Breithardt G, Camm AJ, Crijns HJ, Kuck KH, Vardas P, Wegscheider K. Improving outcomes in patients with atrial fibrillation: rationale and design of the Early treatment of Atrial fibrillation for Stroke prevention Trial. *Am Heart J*. 2013;166:442-448

39. Steg PG, Alam S, Chiang C-E, Gamra H, Goethals M, Inoue H, Krapf L, Lewalter T, Merioua I, Murin J, Naditch-Brûlé L, Ponikowski P, Rosenqvist M, Silva-Cardoso J, Zharinov O, Brette S, Neill JO. Symptoms, functional status and quality of life in patients with controlled and uncontrolled atrial fibrillation: data from the RealiseAF cross-sectional international registry. *Heart*. 2012;98:195-201

40. Nabauer M, Gerth A, Limbourg T, Schneider S, Oeff M, Kirchhof P, Goette A, Lewalter T, Ravens U, Meinertz T, Breithardt G, Steinbeck G. The Registry of the German Competence NETwork on Atrial Fibrillation: patient characteristics and initial management. *Europace*. 2009;11:423-434

41. Segal JB, McNamara RL, Miller MR, Kim N, Goodman SN, Powe NR, Robinson K, Yu D, Bass EB. The evidence regarding the drugs used for ventricular rate control. *J Fam Practice*. 2000;49:47-59

42. Farshi R, Kistner D, Sarma JSM, Longmate JA, Singh BN. Ventricular rate control in chronic atrial fibrillation during daily activity and programmed exercise: a crossover open-label study of five drug regimens. *J Am Coll Cardiol*. 1999;33:304-310

43. Koh KK, Kwon KS, Park HB, Baik SH, Park SJ, Lee KH, Kim EJ, Kim SH, Cho SK, Kim SS. Efficacy and safety of digoxin alone and in combination with low-dose diltiazem or betaxolol to control ventricular rate in chronic atrial fibrillation. *Am J Cardiol*. 1995;75:88-90

44. Nikolaidou T, Channer KS. Chronic atrial fibrillation: a systematic review of medical heart rate control management. *Postgrad Med J*. 2009;85:303-312

For peer review only - http://bmjopen.bmj.com/site/about/guidelines.xhtml

45. Ulimoen SR, Enger S, Carlson J, Platonov PG, Pripp AH, Abdelnoor M, Arnesen H, Gjesdal K, Tveit A. Comparison of four single-drug regimens on ventricular rate and arrhythmia-related symptoms in patients with permanent atrial fibrillation. *Am J Cardiol*. 2013;111:225-230

46. Ulimoen SR, Enger S, Pripp AH, Abdelnoor M, Arnesen H, Gjesdal K, Tveit A. Calcium channel blockers improve exercise capacity and reduce N-terminal Pro-B-type natriuretic peptide levels compared with beta-blockers in patients with permanent atrial fibrillation. *Eur Heart J*. 2014;35:517-524

47. Khand AU, Rankin AC, Martin W, Taylor J, Gemmell I, Cleland JG. Carvedilol alone or in combination with digoxin for the management of atrial fibrillation in patients with heart failure? *J Am Coll Cardiol*. 2003;42:1944-1951

48. Vamos M, Erath JW, Hohnloser SH. Digoxin-associated mortality: a systematic review and meta-analysis of the literature. *Eur Heart J*. 2015; In press: 10.1093/eurheartj/ehv143

49. Gheorghiade M, Fonarow GC, van Veldhuisen DJ, Cleland JG, Butler J, Epstein AE, Patel K, Aban IB, Aronow WS, Anker SD, Ahmed A. Lack of evidence of increased mortality among patients with atrial fibrillation taking digoxin: findings from post hoc propensity-matched analysis of the AFFIRM trial. *Eur Heart J*. 2013;34:1489-1497

50. Friberg L, Hammar N, Rosenqvist M. Digoxin in atrial fibrillation: report from the Stockholm Cohort study of Atrial Fibrillation (SCAF). *Heart*. 2010;96:275-280

51. Flory JH, Ky B, Haynes K, S MB, Munson J, Rowan C, Strom BL, Hennessy S. Observational cohort study of the safety of digoxin use in women with heart failure. *BMJ Open*. 2012;2:e000888

52. Andrey JL, Romero S, Garcia-Egido A, Escobar MA, Corzo R, Garcia-Dominguez G, Lechuga V, Gomez F. Mortality and morbidity of heart failure treated with digoxin. A propensity-matched study. *Int J Clin Pract*. 2011;65:1250-1258

53. Lewis RV, Irvine N, McDevitt DG. Relationships between heart rate, exercise tolerance and cardiac output in atrial fibrillation: the effects of treatment with digoxin, verapamil and diltiazem. *Eur Heart J*. 1988;9:777-781

54. Tsuneda T, Yamashita T, Fukunami M, Kumagai K, Niwano S, Okumura K, Inoue H. Rate control and quality of life in patients with permanent atrial fibrillation: the Quality of Life and Atrial Fibrillation (QOLAF) Study. *Circ J*. 2006;70:965-970

55. Van Gelder IC, Groenveld HF, Crijns HJGM, Tuininga YS, Tijssen JGP, Alings AM, Hillege HL, Bergsma-Kadijk JA, Cornel JH, Kamp O, Tukkie R, Bosker HA, Van Veldhuisen DJ, Van den Berg MP. Lenient versus Strict Rate Control in Patients with Atrial Fibrillation. *N Engl J Med*. 2010;362:1363-1373

56. Groenveld HF, Crijns HJGM, Van den Berg MP, Van Sonderen E, Alings AM, Tijssen JGP, Hillege HL, Tuininga YS, Van Veldhuisen DJ, Ranchor AV, Van Gelder IC. The Effect of Rate Control on Quality of Life in Patients With Permanent Atrial Fibrillation: Data From the RACE II (Rate Control Efficacy in Permanent Atrial Fibrillation II) Study. *J Am Coll Cardiol*. 2011;58:1795-1803

57. Mulder BA, Van Veldhuisen DJ, Crijns HJGM, Tijssen JGP, Hillege HL, Alings M, Rienstra M, Groenveld HF, Van den Berg MP, Van Gelder IC. Lenient vs. strict rate control in patients with atrial fibrillation and heart failure: a post-hoc analysis of the RACE II study. *Eur J Heart Fail*. 2013;15:1311-1318

58. Groenveld HF, Tijssen JG, Crijns HJ, Van den Berg MP, Hillege HL, Alings M, Van Veldhuisen DJ, Van Gelder IC. Rate control efficacy in permanent atrial fibrillation: successful and failed strict rate control against a background of lenient rate control: data from RACE II (Rate Control Efficacy in Permanent Atrial Fibrillation). *J Am Coll Cardiol*. 2013;61:741-748

59. US Department of Health and Human Services Food and Drug Administration. Guidance for industry: patient-reported outcome measures: use in medical product development to support

For peer review only - http://bmjopen.bmj.com/site/about/guidelines.xhtml

labeling claims. www.fda.gov/downloads/Drugs/GuidanceComplianceRegulatoryInformation/Guidances/UCM193282.pdf. 2009

60. Rienstra M, Lubitz SA, Mahida S, Magnani JW, Fontes JD, Sinner MF, Van Gelder IC, Ellinor PT, Benjamin EJ. Symptoms and Functional Status of Patients With Atrial Fibrillation: State of the Art and Future Research Opportunities. *Circulation*. 2012;125:2933-2943

61. Pepine CJ. Effects of pharmacologic therapy on health-related quality of life in elderly patients with atrial fibrillation: a systematic review of randomized and nonrandomized trials. *Clin Med Insights Cardiol*. 2013;7:1-20

62. Hagens VE, Ranchor AV, Van Sonderen E, Bosker HA, Kamp O, Tijssen JGP, Kingma JH, Crijns HJGM, Van Gelder IC. Effect of rate or rhythm control on quality of life in persistent atrial fibrillation: Results from the Rate Control Versus Electrical Cardioversion (RACE) study. *J Am Coll Cardiol*. 2004;43:241-247

63. Grönefeld GC, Lilienthal J, Kuck K-H, Hohnloser SH. Impact of rate versus rhythm control on quality of life in patients with persistent atrial fibrillation: Results from a prospective randomized study. *Eur Heart J*. 2003;24:1430-1436

64. Ware Jr JE, Gandek B. Overview of the SF-36 Health Survey and the International Quality of Life Assessment (IQOLA) Project. *J Clin Epidemiol*. 1998;51:903-912

65. Herdman M, Gudex C, Lloyd A, Janssen M, Kind P, Parkin D, Bonsel G, Badia X. Development and preliminary testing of the new five-level version of EQ-5D (EQ-5D-5L). *Qual Life Res*. 2011;20:1727-1736

66. Devlin NJ, Krabbe PF. The development of new research methods for the valuation of EQ-5D-5L. *Eur J Health Econ*. 2013;14 Suppl 1:S1-3

67. Spertus J, Dorian P, Bubien R, Lewis S, Godejohn D, Reynolds MR, Lakkireddy DR, Wimmer AP, Bhandari A, Burk C. Development and validation of the Atrial Fibrillation Effect on QualiTy-of-Life (AFEQT) Questionnaire in patients with atrial fibrillation. *Circ Arrhythm Electrophysiol*. 2011;4:15-25

68. Dorian P, Burk C, Mullin CM, Bubien R, Godejohn D, Reynolds MR, Lakkireddy DR, Wimmer AP, Bhandari A, Spertus J. Interpreting changes in quality of life in atrial fibrillation: How much change is meaningful? *Am Heart J*. 2013;166:381-387.e388

69. Joint Formulary Committee. *British National Formulary*. London: BMJ Group and Pharmaceutical Press; 2013.

70. American Hospital Formulary Service. *Drug Information*. Bethesda: American Society of Health-System Pharmacists; 2013.

71. The Digitalis Investigation Group. The effect of digoxin on mortality and morbidity in patients with heart failure. *N Engl J Med*. 1997;336:525-533

72. Magnani B, Malini PL. Cardiac glycosides. Drug interactions of clinical significance. *Drug Safety*. 1995;12:97-109

73. Ko DT, Hebert PR, Coffey CS, Sedrakyan A, Curtis JP, Krumholz HM. Beta-blocker therapy and symptoms of depression, fatigue, and sexual dysfunction. *JAMA*. 2002;288:351-357

74. Terwee C, Mokkink L, Knol D, Ostelo RJG, Bouter L, Vet HW. Rating the methodological quality in systematic reviews of studies on measurement properties: a scoring system for the COSMIN checklist. *Qual Life Res*. 2012;21:651-657

75. Streiner DL, Norman GR. *Health measurement scales : a practical guide to their development and use*. Oxford ; New York: Oxford University Press; 2008.

For peer review only - http://bmjopen.bmj.com/site/about/guidelines.xhtml

76. Staniszewska S, Haywood KL, Brett J, Tutton L. Patient and public involvement in patient-reported outcome measures: evolution not revolution. *Patient*. 2012;5:79-87

77. Calvert M, Kyte D, Duffy H, Gheorghe A, Mercieca-Bebber R, Ives J, Draper H, Brundage M, Blazeby J, King M. Patient-reported outcome (PRO) assessment in clinical trials: a systematic review of guidance for trial protocol writers. *PLoS One*. 2014;9:e110216

78. McCabe PJ, Schumacher K, Barnason SA. Living with atrial fibrillation: a qualitative study. *J Cardiovasc Nurs*. 2011;26:336-344

79. Kirchhof P, Auricchio A, Bax J, Crijns H, Camm J, Diener H-C, Goette A, Hindricks G, Hohnloser S, Kappenberger L, Kuck K-H, Lip GYH, Olsson B, Meinertz T, Priori S, Ravens U, Steinbeck G, Svernhage E, Tijssen J, Vincent A, Breithardt G. Outcome parameters for trials in atrial fibrillation: Recommendations from a consensus conference organized by the German Atrial Fibrillation Competence NETwork (AFNET) and the European Heart Rhythm Association (EHRA). *Eur Heart J*. 2007;28:2803-2817

80. Haywood K, Brett J, Salek S, Marlett N, Penman C, Shklarov S, Norris C, Santana MJ, Staniszewska S. Patient and public engagement in health-related quality of life and patient-reported outcomes research: what is important and why should we care? Findings from the first ISOQOL patient engagement symposium. *Qual Life Res*. 2014: Epub ahead of print; PMID 25194573

81. Chan AW, Tetzlaff JM, Gotzsche PC, Altman DG, Mann H, Berlin JA, Dickersin K, Hrobjartsson A, Schulz KF, Parulekar WR, Krleza-Jeric K, Laupacis A, Moher D. SPIRIT 2013 explanation and elaboration: guidance for protocols of clinical trials. *BMJ*. 2013;346:e7586

82. Calvert M, Kyte D, von Hildebrand M, King M, Moher D. Putting patients at the heart of health-care research. *Lancet*. 2015;385:1073-1074

83. Kyte D, Duffy H, Fletcher B, Gheorghe A, Mercieca-Bebber R, King M, Draper H, Ives J, Brundage M, Blazeby J, Calvert M. Systematic evaluation of the patient-reported outcome (PRO) content of clinical trial protocols. *PLoS One*. 2014;9:e110229

84. Kyte D, Draper H, Calvert M. Patient-reported outcome alerts: ethical and logistical considerations in clinical trials. *JAMA*. 2013;310:1229-1230

85. Zbrozek A, Hebert J, Gogates G, Thorell R, Dell C, Molsen E, Craig G, Grice K, Kern S, Hines S. Validation of electronic systems to collect patient-reported outcome (PRO) data-recommendations for clinical trial teams: report of the ISPOR ePRO systems validation good research practices task force. *Value Health*. 2013;16:480-489

86. Su H-M, Lin T-H, Hsu P-C, Lee W-H, Chu C-Y, Lee C-S, Voon W-C, Lai W-T, Sheu S-H. Global left ventricular longitudinal systolic strain as a major predictor of cardiovascular events in patients with atrial fibrillation. *Heart*. 2013;99:1588-1596

87. Al-Omari MA, Finstuen J, Appleton CP, Barnes ME, Tsang TSM. Echocardiographic assessment of left ventricular diastolic function and filling pressure in atrial fibrillation. *Am J Cardiol*. 2008;101:1759-1765

88. Hijazi Z, Oldgren J, Siegbahn A, Granger CB, Wallentin L. Biomarkers in atrial fibrillation: a clinical review. *Eur Heart J*. 2013;34:1475-1480

89. Parvez B, Chopra N, Rowan S, Vaglio JC, Muhammad R, Roden DM, Darbar D. A common beta1-adrenergic receptor polymorphism predicts favorable response to rate-control therapy in atrial fibrillation. *J Am Coll Cardiol*. 2012;59:49-56

90. Mahmmoud YA, Shattock M, Cornelius F, Pavlovic D. Inhibition of K+ transport through Na+, K+-ATPase by capsazepine: role of membrane span 10 of the alpha-subunit in the modulation of ion gating. *PLoS One*. 2014;9:e96909

For peer review only - http://bmjopen.bmj.com/site/about/guidelines.xhtml

91. Pavlovic D, Hall AR, Kennington EJ, Aughton K, Boguslavskyi A, Fuller W, Despa S, Bers DM, Shattock MJ. Nitric oxide regulates cardiac intracellular Na(+) and Ca(2)(+) by modulating Na/K ATPase via PKCepsilon and phospholemman-dependent mechanism. *J Mol Cell Cardiol*. 2013;61:164-171

92. Pullen MA, Brooks DP, Edwards RM. Characterization of the neutralizing activity of digoxin-specific Fab toward ouabain-like steroids. *J Pharmacol Exp Ther*. 2004;310:319-325

93. Manunta P, Messaggio E, Casamassima N, Gatti G, Carpini SD, Zagato L, Hamlyn JM. Endogenous ouabain in renal Na(+) handling and related diseases. *Biochim Biophys Acta*. 2010;1802:1214-1218

94. Pavlovic D. The role of cardiotonic steroids in the pathogenesis of cardiomyopathy in chronic kidney disease. *Nephron Clin Pract*. 2014;128:11-21

95. Song H, Karashima E, Hamlyn JM, Blaustein MP. Ouabain-digoxin antagonism in rat arteries and neurones. *J Physiol*. 2014;592:941-969

96. Curtis L. Unit Costs of Health & Social Care 2012. *Personal Social Services Research Unit*. 2012:http://www.pssru.ac.uk/archive/pdf/uc/uc2012/full-with-covers.pdf

97. Jerosch-Herold M. Quantification of myocardial perfusion by cardiovascular magnetic resonance. *Journal of Cardiovascular Magnetic Resonance*. 2010;12:57

For peer review only

For peer review only - http://bmjopen.bmj.com/site/about/guidelines.xhtml

APPENDIX A – Dosing Schedule (Digoxin)

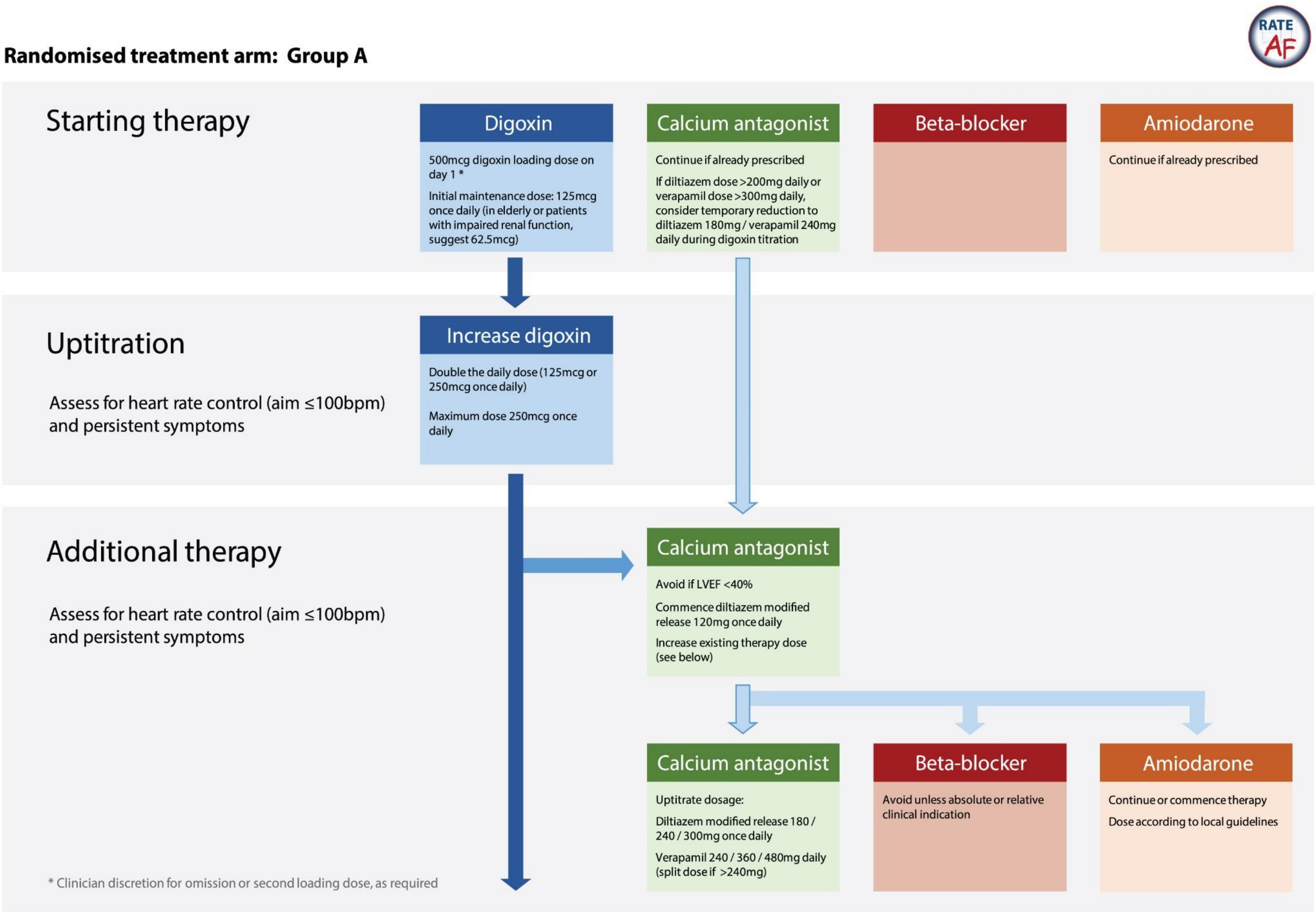

For peer review only - http://bmjopen.bmj.com/site/about/guidelines.xhtml

APPENDIX B – Dosing Schedule (Bisoprolol)

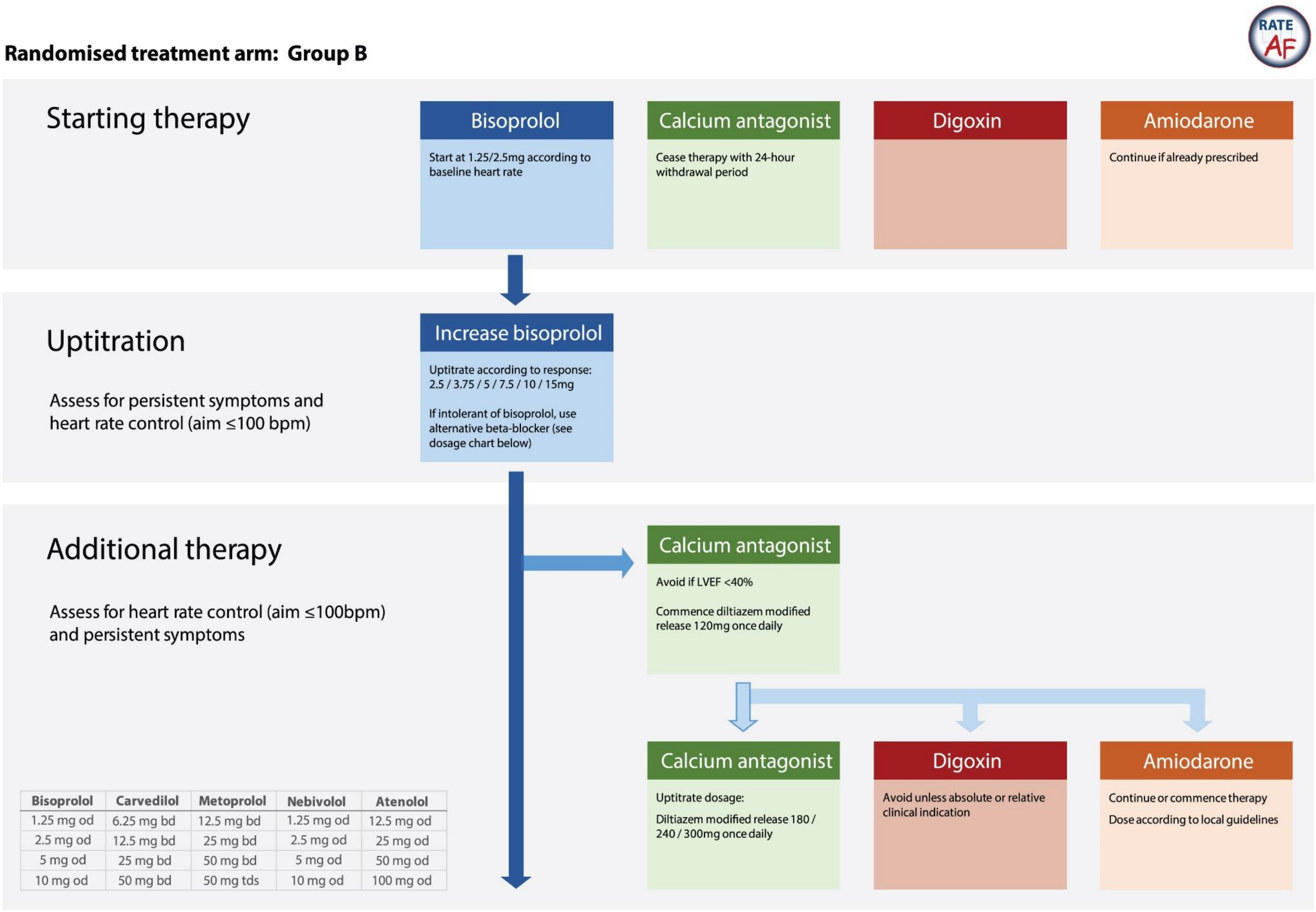

Bisoprolol	Carvedilol	Metoprolol	Nebivolol	Atenolol
1.25 mg od	6.25 mg bd	12.5 mg bd	1.25 mg od	12.5 mg od
2.5 mg od	12.5 mg bd	25 mg bd	2.5 mg od	25 mg od
5 mg od	25 mg bd	50 mg bd	5 mg od	50 mg od
10 mg od	50 mg bd	50 mg tds	10 mg od	100 mg od

SPIRIT

STANDARD PROTOCOL ITEMS: RECOMMENDATIONS FOR INTERVENTIONAL TRIALS

SPIRIT 2013 Checklist: Recommended items to address in a clinical trial protocol and related documents*

Section/item	Item No	Description	Addressed on page number
**Administrative information**			
Title	1	Descriptive title identifying the study design, population, interventions, and, if applicable, trial acronym	______________
Trial registration	2a	Trial identifier and registry name. If not yet registered, name of intended registry	______________
	2b	All items from the World Health Organization Trial Registration Data Set	______________
Protocol version	3	Date and version identifier	______________
Funding	4	Sources and types of financial, material, and other support	______________
Roles and responsibilities	5a	Names, affiliations, and roles of protocol contributors	______________
	5b	Name and contact information for the trial sponsor	______________
	5c	Role of study sponsor and funders, if any, in study design; collection, management, analysis, and interpretation of data; writing of the report; and the decision to submit the report for publication, including whether they will have ultimate authority over any of these activities	______________
	5d	Composition, roles, and responsibilities of the coordinating centre, steering committee, endpoint adjudication committee, data management team, and other individuals or groups overseeing the trial, if applicable (see Item 21a for data monitoring committee)	______________

For peer review only

**Introduction**			
Background and rationale	6a	Description of research question and justification for undertaking the trial, including summary of relevant studies (published and unpublished) examining benefits and harms for each intervention	
	6b	Explanation for choice of comparators	
Objectives	7	Specific objectives or hypotheses	
Trial design	8	Description of trial design including type of trial (eg, parallel group, crossover, factorial, single group), allocation ratio, and framework (eg, superiority, equivalence, noninferiority, exploratory)	
**Methods: Participants, interventions, and outcomes**			
Study setting	9	Description of study settings (eg, community clinic, academic hospital) and list of countries where data will be collected. Reference to where list of study sites can be obtained	
Eligibility criteria	10	Inclusion and exclusion criteria for participants. If applicable, eligibility criteria for study centres and individuals who will perform the interventions (eg, surgeons, psychotherapists)	
Interventions	11a	Interventions for each group with sufficient detail to allow replication, including how and when they will be administered	
	11b	Criteria for discontinuing or modifying allocated interventions for a given trial participant (eg, drug dose change in response to harms, participant request, or improving/worsening disease)	
	11c	Strategies to improve adherence to intervention protocols, and any procedures for monitoring adherence (eg, drug tablet return, laboratory tests)	
	11d	Relevant concomitant care and interventions that are permitted or prohibited during the trial	
Outcomes	12	Primary, secondary, and other outcomes, including the specific measurement variable (eg, systolic blood pressure), analysis metric (eg, change from baseline, final value, time to event), method of aggregation (eg, median, proportion), and time point for each outcome. Explanation of the clinical relevance of chosen efficacy and harm outcomes is strongly recommended	
Participant timeline	13	Time schedule of enrolment, interventions (including any run-ins and washouts), assessments, and visits for participants. A schematic diagram is highly recommended (see Figure)	

For peer review only - http://bmjopen.bmj.com/site/about/guidelines.xhtml

Sample size	14	Estimated number of participants needed to achieve study objectives and how it was determined, including clinical and statistical assumptions supporting any sample size calculations	______________
Recruitment	15	Strategies for achieving adequate participant enrolment to reach target sample size	______________

Methods: Assignment of interventions (for controlled trials)

Allocation:

Sequence generation	16a	Method of generating the allocation sequence (eg, computer-generated random numbers), and list of any factors for stratification. To reduce predictability of a random sequence, details of any planned restriction (eg, blocking) should be provided in a separate document that is unavailable to those who enrol participants or assign interventions	______________
Allocation concealment mechanism	16b	Mechanism of implementing the allocation sequence (eg, central telephone; sequentially numbered, opaque, sealed envelopes), describing any steps to conceal the sequence until interventions are assigned	______________
Implementation	16c	Who will generate the allocation sequence, who will enrol participants, and who will assign participants to interventions	______________
Blinding (masking)	17a	Who will be blinded after assignment to interventions (eg, trial participants, care providers, outcome assessors, data analysts), and how	______________
	17b	If blinded, circumstances under which unblinding is permissible, and procedure for revealing a participant's allocated intervention during the trial	______________

Methods: Data collection, management, and analysis

Data collection methods	18a	Plans for assessment and collection of outcome, baseline, and other trial data, including any related processes to promote data quality (eg, duplicate measurements, training of assessors) and a description of study instruments (eg, questionnaires, laboratory tests) along with their reliability and validity, if known. Reference to where data collection forms can be found, if not in the protocol	______________
	18b	Plans to promote participant retention and complete follow-up, including list of any outcome data to be collected for participants who discontinue or deviate from intervention protocols	______________

For peer review only - http://bmjopen.bmj.com/site/about/guidelines.xhtml

Data management	19	Plans for data entry, coding, security, and storage, including any related processes to promote data quality (eg, double data entry; range checks for data values). Reference to where details of data management procedures can be found, if not in the protocol	______________
Statistical methods	20a	Statistical methods for analysing primary and secondary outcomes. Reference to where other details of the statistical analysis plan can be found, if not in the protocol	______________
	20b	Methods for any additional analyses (eg, subgroup and adjusted analyses)	______________
	20c	Definition of analysis population relating to protocol non-adherence (eg, as randomised analysis), and any statistical methods to handle missing data (eg, multiple imputation)	______________
**Methods: Monitoring**			
Data monitoring	21a	Composition of data monitoring committee (DMC); summary of its role and reporting structure; statement of whether it is independent from the sponsor and competing interests; and reference to where further details about its charter can be found, if not in the protocol. Alternatively, an explanation of why a DMC is not needed	______________
	21b	Description of any interim analyses and stopping guidelines, including who will have access to these interim results and make the final decision to terminate the trial	______________
Harms	22	Plans for collecting, assessing, reporting, and managing solicited and spontaneously reported adverse events and other unintended effects of trial interventions or trial conduct	______________
Auditing	23	Frequency and procedures for auditing trial conduct, if any, and whether the process will be independent from investigators and the sponsor	______________
**Ethics and dissemination**			
Research ethics approval	24	Plans for seeking research ethics committee/institutional review board (REC/IRB) approval	______________
Protocol amendments	25	Plans for communicating important protocol modifications (eg, changes to eligibility criteria, outcomes, analyses) to relevant parties (eg, investigators, REC/IRBs, trial participants, trial registries, journals, regulators)	______________

For peer review only - http://bmjopen.bmj.com/site/about/guidelines.xhtml

Consent or assent	26a	Who will obtain informed consent or assent from potential trial participants or authorised surrogates, and how (see Item 32)	______________
	26b	Additional consent provisions for collection and use of participant data and biological specimens in ancillary studies, if applicable	______________
Confidentiality	27	How personal information about potential and enrolled participants will be collected, shared, and maintained in order to protect confidentiality before, during, and after the trial	______________
Declaration of interests	28	Financial and other competing interests for principal investigators for the overall trial and each study site	______________
Access to data	29	Statement of who will have access to the final trial dataset, and disclosure of contractual agreements that limit such access for investigators	______________
Ancillary and post-trial care	30	Provisions, if any, for ancillary and post-trial care, and for compensation to those who suffer harm from trial participation	______________
Dissemination policy	31a	Plans for investigators and sponsor to communicate trial results to participants, healthcare professionals, the public, and other relevant groups (eg, via publication, reporting in results databases, or other data sharing arrangements), including any publication restrictions	______________
	31b	Authorship eligibility guidelines and any intended use of professional writers	______________
	31c	Plans, if any, for granting public access to the full protocol, participant-level dataset, and statistical code	______________
**Appendices**			
Informed consent materials	32	Model consent form and other related documentation given to participants and authorised surrogates	______________
Biological specimens	33	Plans for collection, laboratory evaluation, and storage of biological specimens for genetic or molecular analysis in the current trial and for future use in ancillary studies, if applicable	______________

*It is strongly recommended that this checklist be read in conjunction with the SPIRIT 2013 Explanation & Elaboration for important clarification on the items. Amendments to the protocol should be tracked and dated. The SPIRIT checklist is copyrighted by the SPIRIT Group under the Creative Commons "Attribution-NonCommercial-NoDerivs 3.0 Unported" license.

For peer review only

For peer review only - http://bmjopen.bmj.com/site/about/guidelines.xhtml

BMJ Open

A review of rate control in atrial fibrillation, and the rationale and protocol for the RATE-AF trial

Journal:	*BMJ Open*
Manuscript ID	bmjopen-2016-015099.R2
Article Type:	Protocol
Date Submitted by the Author:	29-Apr-2017

Complete List of Authors:	Kotecha, Dipak; University of Birmingham, Institute of Cardiovascular Sciences; University Hospitals Birmingham NHS Trust, Cardiology<br>Calvert, Melanie; University of Birmingham, Centre for Patient Reported Outcomes Research; University of Birmingham, Institute of Applied Health Research<br>Deeks, Jon; University of Birmingham, Birmingham Clinical Trials Unit; University of Birmingham, Institute of Applied Health Research<br>Griffith, Mike; University Hospitals Birmingham NHS Trust, Cardiology<br>Kirchhof, Paulus; University of Birmingham, Institute of Cardiovascular Sciences; Sandwell & West Birmingham Hospitals NHS Trust, Cardiology<br>Lip, Gregory; University of Birmingham, Institute of Cardiovascular Sciences; Sandwell & West Birmingham Hospitals NHS Trust, Cardiology<br>Mehta, Samir; University of Birmingham, Birmingham Clinical Trials Unit<br>Slinn, Gemma; University of Birmingham, Birmingham Clinical Trials Unit<br>Stanbury, Mary; (Lead for the patient involvement panel)<br>Steeds, Richard; University Hospitals Birmingham NHS Trust, Cardiology; University of Birmingham, Institute of Cardiovascular Sciences<br>Townend, Jonathan; University Hospitals Birmingham NHS Trust, Cardiology; University of Birmingham, Institute of Cardiovascular Sciences

**Primary Subject Heading**:	Cardiovascular medicine
Secondary Subject Heading:	Evidence based practice, Medical management, Patient-centred medicine
Keywords:	Atrial fibrillation, heart rate, quality of life, Echocardiography < CARDIOLOGY, Protocols & guidelines < HEALTH SERVICES ADMINISTRATION & MANAGEMENT, RATE-AF

SCHOLARONE™ Manuscripts

Title: A review of rate control in atrial fibrillation, and the rationale and protocol for the RATE-AF trial

Brief Title: Kotecha *et al*, Rate control therapy evaluation in atrial fibrillation

For peer review only

Authors: Dr Dipak Kotecha MBChB PhD MRCP FESC FHEA[1,2,3,4]*, Prof Melanie Calvert BSc PhD FHEA[4,5], Prof Jonathan J Deeks BSc MSc PhD CStat[5,6], Dr Michael Griffith MD FRCP[2], Prof Paulus Kirchhof MD FRCP FESC[1,2,3,4], Prof Gregory YH Lip MD FRCP DFM FESC[1,3,4], Samir Mehta MSc BSc[6], Gemma Slinn BSc MPhil[6], Mary Stanbury RGN RDN RHV**, Dr Richard P Steeds MA MD FRCP FESC[1,2] and Prof Jonathan N Townend BSc MBChB MD FESC[1,2].

From the (1) Institute of Cardiovascular Sciences, University of Birmingham, Birmingham, UK; (2) University Hospitals Birmingham NHS Trust, Birmingham, UK; (3) Sandwell & West Birmingham Hospitals NHS Trust, Birmingham, UK; (4) Centre for Patient Reported Outcomes Research, University of Birmingham, Birmingham, UK; (5) Institute of Applied Health Research, University of Birmingham, Birmingham, UK; and (6) Birmingham Clinical Trials Unit, University of Birmingham, Birmingham, UK. * Authors after the Chief Investigator listed in alphabetical order. ** Lead for the Patient Involvement Panel.

Address for correspondence:

Dr Dipak Kotecha

University of Birmingham Institute of Cardiovascular Sciences, Medical School, Vincent Drive, Birmingham, B15 2TT, UK.

Email: d.kotecha@bham.ac.uk Tel: +44 121 371 8122 Fax: +44 121 554 4083

Word count (text): 2963 Word count (abstract): 300

For peer review only - http://bmjopen.bmj.com/site/about/guidelines.xhtml

Key Words: Atrial fibrillation; heart rate; Protocols & guidelines<health services administration & management; RATE-AF trial; quality of life; echocardiography<cardiology.

For peer review only

For peer review only - http://bmjopen.bmj.com/site/about/guidelines.xhtml

Abstract

Background & Objective: Atrial fibrillation (AF) is common and causes impaired quality of life, an increased risk of stroke and death, as well as frequent hospital admissions. The majority of patients with AF require control of heart rate. In this article we summarise the limited evidence from clinical trials that guides prescription, and present the rationale and protocol for a new randomised trial. As rate control has not yet been shown to reduce mortality, there is a clear need to compare the impact of therapy on quality of life, cardiac function and exercise capacity. Such a trial should concentrate on the longer-term effects of treatment in the largest proportion of AF patients, those with symptomatic permanent AF, with the aim of improving patient well-being.

Design & Intervention: The RAte control Therapy Evaluation in permanent Atrial Fibrillation (RATE-AF) trial will enrol 160 participants with a prospective, randomised, open-label, blinded end-point design comparing initial rate control with digoxin or bisoprolol. This will be the first head-to-head randomised trial of digoxin and beta-blockers in AF.

Participants: Recruited patients will be aged ≥60 years with permanent AF and symptoms of breathlessness (NYHA Class II or above), with few exclusion criteria to maximise generalisability to routine clinical practice.

Outcome measures: The primary outcome is patient-reported quality of life, with secondary outcomes including echocardiographic ventricular function, exercise capacity and biomarkers of cellular and clinical response. Follow-up will occur at 6 and 12 months, with feasibility components to inform the design of a future trial powered to detect a difference in hospital admission. The RATE-AF trial will underpin an integrated approach to management including biomarkers, function and symptoms that will guide future research into optimal, personalised rate control in patients with AF.

Ethics and Dissemination: East Midlands-Derby Research Ethics Committee (16/EM/0178); Peer-reviewed publications.

For peer review only - http://bmjopen.bmj.com/site/about/guidelines.xhtml

Trial registration: clinicaltrials.gov:NCT02391337; ISRCTN:95259705.

For peer review only

For peer review only - http://bmjopen.bmj.com/site/about/guidelines.xhtml

Strengths and limitations of this study

- Control of heart rate is universally used in patients with atrial fibrillation, but evidence from good quality randomised trials is extremely limited.
- Despite common clinical use, there has never been a direct randomised comparison of beta-blockers and digoxin for heart rate control in AF patients (with or without heart failure).
- The RATE-AF trial will assess the effect of therapy on patient-reported quality of life, and improve methods to capture this information in patients with AF. The trial will also evaluate the longer-term impact on cardiac function, define reproducible methods to measure systolic and diastolic function in AF, and develop new biomarkers for personalisation of treatment.
- The trial will not have the power to identify differences in clinical events, but will allow us to plan a future trial designed to detect a difference in the need for admissions to hospital.

For peer review only

For peer review only - http://bmjopen.bmj.com/site/about/guidelines.xhtml

Introduction

Atrial fibrillation (AF) is a common cause of stroke and cardiovascular death, leads to poor quality of life and doubles the risk of hospital admission.[1] We are currently in the midst of an epidemic of AF, with both incidence and prevalence expected to double in the next 20 years.[2-4] Although AF can affect any age-group, patients are typically elderly with significant comorbidities, including up to 50% suffering from heart failure.[5] AF is both a cause and consequence of heart failure, with complex interactions leading to impairment of systolic and diastolic function.[6 7] The combination of these two conditions is expected to have a dramatic impact on the burden of healthcare worldwide.[8-11]

Management of AF involves anticoagulation to prevent strokes, selecting appropriate patients for restoration of sinus rhythm and almost universal need for control of heart rate. In contrast to other management strategies, the choice of rate control therapy has a very low-quality evidence-base (**Figure 1**).[12] Guidelines from the National Institute for Health and Care Excellence (NICE) and the European Society of Cardiology (ESC) have mandated further research specifically on rate control[1 13], which is also reflected in the level of recommendations from the American Heart Association.[14] The small studies currently available are often uncontrolled or with short follow-up[15-19], providing few insights on the biological effects of treatment or the mechanisms underpinning the response to therapy. With no evidence for any impact of rate control on mortality[20 21], and limited data for any difference in quality of life or functional outcomes, the choice of rate control agent is currently informed by expert consensus and physician experience.

In this paper, we review the current evidence-base for rate control in AF and the rationale for a new randomised controlled trial (RCT). The <u>RA</u>te control <u>T</u>herapy <u>E</u>valuation in permanent <u>A</u>trial <u>F</u>ibrillation (RATE-AF) trial will compare initial therapy with beta-blockers versus digoxin in older patients with symptomatic permanent AF, assessing quality of life, functional

For peer review only - http://bmjopen.bmj.com/site/about/guidelines.xhtml

capacity, left-ventricular ejection fraction (LVEF), diastolic function and biomarkers of treatment response.

Rationale for a new trial of rate control in AF

Why not choose a rhythm control strategy?

A number of RCTs have assessed the addition of rhythm control strategies to control of heart rate in AF patients, most often with anti-arrhythmic drugs (AAD) and direct current cardioversion. Neither of the two largest trials (AFFIRM or RACE) found any difference in clinical outcomes comparing these approaches.[22 23] Meta-analyses and other smaller trials have confirmed that rhythm control is not superior to regulation of heart rate alone,[24-26] including heart failure patients with both impaired and preserved ejection fraction.[27 28] These studies have analysed heterogeneous populations, including both paroxysmal and permanent AF that may differ with regards to mechanism, prognosis and the response to treatment.[15] However there is also evidence that a rhythm control strategy may increase hospital admissions. A meta-analysis of major published trials is presented in **Figure 2**, highlighting a 17% increase in the risk of hospitalisation in the rhythm control group (after exclusion of hospital visits related to cardioversion). Although limited by patient crossover and the association between AAD and adverse events,[29] the results highlight the importance of trials comparing different rate control options and associated healthcare costs.

Although AF ablation is becoming increasingly popular, it remains a highly invasive method to restore sinus rhythm.[30 31] Current guidelines recommend ablation to improve AF-related symptoms in patients with paroxysmal AF, or as a treatment option in symptomatic persistent AF that is refractory to other therapy.[1 14] Long-term outcome studies are awaited and need to be balanced against procedural complications and AF recurrence. Even in patients

For peer review only - http://bmjopen.bmj.com/site/about/guidelines.xhtml

receiving intensive rhythm control therapy, rate control is often necessary to reduce symptoms during AF paroxysms. Further, 40-50% of AF patients are deemed as unsuitable for rhythm control (permanent AF),[5 32] and are maintained on rate control therapy to reduce potential symptoms and avoid tachycardia that may worsen ventricular function.[6] Patients with permanent AF have a higher residual risk of cardiovascular death, stroke or systemic embolism, despite anticoagulation.[33]

What is the optimal heart rate target in AF?

There is clinical uncertainty about how to control heart rate and the intensity of rate-reduction. In the RACE II trial of 614 randomised patients with permanent AF, there were no benefits of strict (<80 bpm at rest) compared to lenient rate control (resting heart rate <110 bpm) over 3 years of follow-up.[34] Although interpretation was limited by the narrow difference in heart rate between groups, lenient rate control was found to be non-inferior with an adjusted hazard ratio of 0.80 (90% CI 0.55-1.17) for a wide composite of adverse clinical outcomes (12.9%, compared to 14.9% in the strict control arm). In addition, there were no differences in symptoms or NYHA class,[34 35] and patients achieving strict rate control required more clinic visits.[36] These findings are consistent with other trials,[37-39] registries,[32] and even randomized[40] and observational[41] cohorts in patients with concomitant heart failure, suggesting that intensity of heart rate control is not the key determinant of outcomes in AF.

Do outcomes vary with different rate control therapies?

Medical therapy to achieve rate control in AF can be achieved with beta-blockers, digoxin and non-dihydropiridine calcium channel blockers (CCB; diltiazem or verapamil).[1] Only a limited evidence-base is available to assist clinicians in choosing first-line and subsequent therapy, resulting in wide variations in clinical practice[42-44] and frequent use of combination therapy. Guidelines suggest the choice of medication should be individualised, dependent on the

For peer review only - http://bmjopen.bmj.com/site/about/guidelines.xhtml

presence of ongoing symptoms.[1 14] However, these recommendations are based on low quality trials and observational data, often with small numbers of participants and follow-up over a few weeks.[16] There are no RCTs comparing long-term rate control options in AF. Demonstrating any reduction in hard clinical outcomes with rate control has proved elusive. In patients with heart failure, reduced ejection fraction and concomitant AF, an individual patient level meta-analysis of double-blind RCT data has suggested that beta-blockers do not reduce all-cause mortality or hospital admissions compared to placebo[20], in contrast to the substantial benefit seen in sinus rhythm.[45] Similarly, the use of digoxin was not associated with any increase, or reduction, in mortality in a comprehensive systematic review.[21] This finding deviates from prior observational analyses which are confounded by the fact that sicker patients tend to receive digoxin more often, which can only be addressed within a randomised trial. Although digoxin is known to reduce hospital admissions in patients with heart failure and reduced ejection fraction in sinus rhythm[46], the impact in patients with AF is unknown.

If rate control has limited effect on mortality, what about evidence for a differential effect on other outcomes, such as functional capacity, cardiac function or quality of life? Beta-blockers are the most commonly-used rate control agents and although they have a greater impact than digoxin on heart rate during exertion, there is no evidence that this results in better exercise capacity.[17 18 47-49] Beta-blockers were not associated with any improvement in arrhythmia-related symptoms in a small RCT of 60 low-risk patients with permanent AF, compared to diltiazem and verapamil which reduced the frequency of symptoms.[50] Those in the beta-blocker group had a reduction in exercise capacity and increase in B-type natriuretic peptide (BNP) compared to those treated with CCB.[51] Analysis of smaller trials comparing beta-blockers with CCB are inconsistent.[17] Compared to verapamil or diltiazem, digoxin has less effect on heart rate but there is no consistent evidence for any difference in functional outcomes.[17 18 47 49 52] Importantly, diltiazem and verapamil are usually avoided in patients with

For peer review only - http://bmjopen.bmj.com/site/about/guidelines.xhtml

reduced ejection fraction due to the risk of adverse outcomes,[53-57] leaving only beta-blockers or digoxin as suitable therapy. Only a single RCT has been published comparing beta-blockers with digoxin in patients with AF and heart failure (mean LVEF 24%, n=47).[58] Although there was a marginally-significant improvement in LVEF with combined carvedilol/digoxin versus placebo/digoxin, blinded withdrawal of digoxin then led to a deterioration in LVEF, accompanied by an increase in BNP. The direct effects of digoxin on LVEF and diastolic function have only been studied in patients with sinus rhythm, where digoxin increased LVEF by 3-11% and improved diastolic filling.[59-61] Magnesium has been shown to complement digoxin therapy to achieve lower ventricular rates in AF [62], but is not in common use due to the availability of beta-blockers and CCB which are more potent agents for acute heart rate control.[1] Although data on patient-reported quality of life is limited,[63 64] rate control has been associated with improved quality of life in trials assessing rate versus rhythm control.[65-67] The mechanism by which rate control therapy mediates an increase in physical functioning and quality of life is unknown but conceivably due to improvements in LVEF and/or diastolic function.

In summary, rate control is an important part of treatment in all AF patients but the evidence-base is poor, particularly in those with permanent AF who form the majority of patients in clinical practice. Rate control in AF is also subject to considerable, and poorly characterised individual variability in response, with limited information about the effects of therapy on cardiac function, quality of life and functional capacity.

The RATE-AF trial

The RATE-AF trial is the first head-to-head randomised assessment of beta-blockers versus digoxin as the initial rate control agent in patients with AF. The trial has a prospective,

For peer review only - http://bmjopen.bmj.com/site/about/guidelines.xhtml

randomised, open-label, investigator-blinded endpoint (PROBE) design, and is planned as an inclusive study that reflects and will have an important impact on clinical practice (see Information for Patients in **Table 1**). The primary outcome is patient-reported quality of life using the SF-36 physical component summary score at 6 months' post-randomisation. The major secondary outcomes are change in LVEF and diastolic function on echocardiography, functional capacity, global and AF-specific quality of life, and cardiovascular biomarkers (**Table 2**). A key objective of the trial is to improve the methods used for measuring quality of life in AF patients, as well as optimising the validity, reproducibility and acquisition of echocardiographic left-ventricular function. The RATE-AF trial will also act as a feasibility study to plan a future, event-driven clinical trial exploring the impact of different rate control strategies on cardiovascular events and unplanned hospital admissions. The study is sponsored by the University of Birmingham and funded by the National Institute for Health Research (NIHR), as part of a Career Development Fellowship awarded to the Chief Investigator (DK).

Methods

Patients

Inclusion criteria are patients aged 60 years or older with breathlessness (equivalent to New York Heart Association Class II or more) and permanent AF, characterised as a physician decision for rate control with no plans for cardioversion, AAD or ablation therapy. Only limited exclusion criteria apply (**Figure 3**), reflecting any clear requirements or contraindications for either beta-blockers or digoxin. As neither agent impacts on mortality in patients with heart failure[20 21], reduced LVEF is not an exclusion criterion. All patients are expected to be anticoagulated if appropriate, according to their clinical risk of stroke and thromboembolism.

For peer review only - http://bmjopen.bmj.com/site/about/guidelines.xhtml

Study procedures and outcomes

One hundred and sixty eligible patients in need of rate control will be invited to participate in the study from primary and secondary care across two major NHS Trusts in Birmingham, UK. The RATE-AF trial is managed by the Birmingham Clinical Trials Unit (BCTU; University of Birmingham) and situated within the Birmingham NIHR/Wellcome Trust Clinical Research Facility.

Following written informed consent, participants will be randomised in a 1:1 ratio to either bisoprolol or digoxin therapy. Randomisation will be provided by a computer-generated minimisation algorithm to ensure balance between the treatment arms for baseline European Heart Rhythm Association (EHRA) class and gender. Allocation will be concealed until the patient has been recruited and consented, thereafter the trial will be open-label.

Baseline assessment procedures will include patient-reported quality of life questionnaires (**Table 3**), 6-minute walk distance, echocardiography and biomarker assessment. Participants will then receive study medication (bisoprolol 1.25-15 milligrams once daily or low-dose digoxin 62.5-250 micrograms once daily), with scheduled uptitration visits to attain a heart rate at rest of ≤100 bpm. This heart rate is in line with international recommendations[1] and was chosen pragmatically to reflect the opinion of many cardiologists that tachycardia can lead to, or worsen, systolic and diastolic dysfunction. Ambulatory 24-hour ECG monitoring will be performed at the end of uptitration (unblinded). Investigator-blinded endpoints will be assessed at the interim (6 month) and final (12 month) visit, which include patient-reported quality of life, echocardiographic parameters of systolic and diastolic left-ventricular function and biomarker assessment (**Figure 3**).

Exploratory work and clinical practice improvement

During the trial, qualitative research using focus groups and structured interviews will assess whether the quality of life questionnaires adequately and acceptably assess changes in

For peer review only - http://bmjopen.bmj.com/site/about/guidelines.xhtml

symptom burden in a sample of patients from each treatment arm. We will also compare and contrast the generic and AF-specific questionnaires. The aim of this work is to improve the methods used for measuring patient-reported outcomes in AF, and to address some of the limitations we have identified in published validation studies.[68]

Optimal acquisition of echocardiography in patients with AF will be determined by reproducibility studies, comparing repeated measures of systolic/diastolic function according to cardiac cycle length. The RATE-AF trial will address the evidence-gaps we have identified in a systematic review of echocardiography in patients with AF[69], and explore the diagnostic difficulty of categorising heart failure in the context of AF (particularly with preserved ejection fraction, where symptom classification is confounded and BNP levels are consistently raised due to AF[7]).

Blood samples from participants will analysed for the cellular effects of rate control, including intracellular sodium, calcium and endogenous cardiotonic steroids (CTS) using photometry in cultured human cardiomyocytes. This work will give mechanistic insight into the cellular response to beta-blockers and digoxin, identify novel markers of treatment effect, and develop assays that are more robust than serum digoxin concentration (SDC) for determining individual patient dosage. SDC is an immunoassay known to be a poor marker of digoxin toxicity[70], which can cross-react with other targets[71] (for example, endogenous CTS). Although SDC will be performed at six months follow-up and as required during the trial to advise clinicians on dose and avoid high digoxin levels, digoxin toxicity remains a clinical diagnosis at present. Serum will also be stored for the development of new blood-based and genetic biomarkers that aid in personalisation of rate control therapy.

Statistical considerations

The null hypothesis is of no difference in the physical functioning domain of the SF-36 quality of life questionnaire when comparing a strategy of digoxin versus beta-blocker therapy for

For peer review only - http://bmjopen.bmj.com/site/about/guidelines.xhtml
For peer review only

initial rate control in older patients with permanent AF. The alternative hypothesis is superiority of one over the other therapy as an initial strategy of care. Randomising 144 patients we can assume an 85% power to detect an effect size of half a standard deviation in a continuous outcome measure of quality of life (two-sided alpha of 0.05). Assuming that 10% of patients will be lost to follow-up, 160 patients are needed. There is some evidence from existing research to support the notion that the treatment effect could be this large. This includes a 17% improvement in SF-36 role-physical score in the rate control arm of the RACE study,[66] a 22% improvement in a proprietary symptom-checklist with CCB (compared to 8% change in those assigned beta-blockers),[19] and 17% improvement with rate control using SF-36 in the PIAF trial.[67] The RATE-AF trial will also us to explore surrogates for clinical outcomes, such as LVEF using echocardiography and B-type natriuretic peptide, and provide estimates for a future definitive trial of rate control in AF, including reliable information on recruitment rates, study drug titration, cross-over, retention and healthcare costs.

Trial oversight, management and registration

RATE-AF will be conducted in accordance with guidelines for Good Clinical Practice (GCP) and the Declaration of Helsinki, and has regulatory approval from the Medicines and Healthcare products Regulatory Agency (MHRA).

Oversight will be provided by a Trial Steering Committee, comprising an independent Data Monitoring Committee and members of the RATE-AF Trial Management Group. This includes representatives of the patient and public involvement panel, involved in both the design and management of the trial. A Clinical Events Committee will be formed to adjudicate on adverse events.

The RATE-AF trial is registered at clinicaltrials.gov (NCT02391337), ISRCTN (95259705) and EudraCT (2015-005043-13). Further information can be obtained from the trial website, http://www.birmingham.ac.uk/rate-af, and the trial protocol (see **Appendix)**. The protocol was

For peer review only - http://bmjopen.bmj.com/site/about/guidelines.xhtml

developed in accordance with the Standard Protocol Items for Randomized Trials [SPIRIT] statement[72], and the latest guidance from the International Society for Quality of Life Research (ISOQOL) Best Practice taskforce.[73-75]

Ethics and Dissemination

The trial has ethical approval from the East Midlands - Derby Research Ethics Committee (16/EM/0178) and approval from the National Health Service (NHS) Health Research Authority (IRAS project ID: 191437).

The research findings will be submitted for publication to peer-reviewed journals after review by the oversight committees and the Patient Involvement Panel, and presented to relevant national and international meetings. Trial participants will be sent a lay summary of the final results of the trial, written by the Patient Involvement Panel.

For peer review only

For peer review only - http://bmjopen.bmj.com/site/about/guidelines.xhtml

Conclusion

Defining appropriate rate control therapy is vital, particularly in the rapidly growing number of older patients with permanent AF where current evidence is extremely limited. Rate control is an integral part of management in almost all AF patients but hardly any controlled trial evidence exists to guide the choice of agents. This is unacceptable in light of the potential benefits and possible adverse effects of treatment. In addition, the complete lack of data on the impact of medical therapy on symptom burden and heart function necessitate a programme of reproducibility and validity of both patient-reported quality of life and cardiac imaging in AF. The RATE-AF trial will answer key clinical questions about how to initiate therapy in order to improve patient well-being, stratified by relevant patient characteristics such as baseline symptoms, systolic and diastolic cardiac function, and biomarkers of treatment effect.

For peer review only - http://bmjopen.bmj.com/site/about/guidelines.xhtml

Acknowledgements

We would like to acknowledge other members of the wider RATE-AF team, including Karina Bunting, Patience Domingos, Dannie Fobian, Margaret Grant, Emma Hayes, Hannah Lack, Susan Jowett, Jonathan Mathers, and Davor Pavlovic (University of Birmingham). We are indebted to the independent members of the trial oversight committees, as well as the Patient and Public Involvement (PPI) Team.

Competing interests

None of the authors report a conflict of interest. All authors have completed the ICMJE uniform disclosure form (www.icmje.org/coi_disclosure.pdf) and declare: DK reports grants from Menarini, during the conduct of the study; non-financial support from Daiichi Sankyo and personal fees from AtriCure, outside the submitted work. MC reports grants from the National Institute of Health Research, during the conduct of the study; and personal fees from Astella Pharma and Ferring Pharma, outside the submitted work. PK reports consulting fees and honoraria from Bayer Healthcare, Boehringer Ingelheim, Bristol-Myers Squibb, Daiichi-Sankyo, Medtronic, Pfizer and Servier, all outside the submitted work; research grants from Bristol-Myers Squibb, Pfizer, Cardiovascular Therapeutics, Daiichi Sankyo, Sanofi, St. Jude Medical, German Federal Ministry for Education and Research (BMBF), Fondation Leducq, German Research Foundation (DFG), European Union, British Heart Foundation and Medical Research Council UK, all outside the submitted work; and is listed on two patent applications on AF therapy and markers for AF, both outside the submitted work. GYHL has served as a consultant for Bayer, Astellas, Merck, AstraZeneca, Sanofi, BMS/Pfizer, Biotronik, Portola and Boehringer Ingelheim, and has been on the speaker's bureau for Bayer, BMS/Pfizer, Boehringer Ingelheim, and Sanofi-Aventis. RPS is the President of the British Society of Echocardiography. JJD, MG, MS. JNT, SM, GS report no

For peer review only

competing interests.

Authors' contributions

The manuscript was drafted by DK who is the Chief Investigator for the RATE-AF trial. MG and GYHL are Principal Investigators. MC, PK, RPS and JNT are members of the Trial Management Group. JJD, SM and GS are representatives from the Clinical Trials Unit. MS is the Lead for the Patient Involvement Panel, and a member of the Steering Committee. All authors contributed to the writing of the RATE-AF protocol or patient information, and edited this manuscript for intellectual content.

Funding

DK and the RATE-AF trial are supported by the National Institute of Health Research (NIHR) as part of a Career Development Fellowship (CDF-2015-08-074). The opinions expressed in this paper are those of the authors and do not represent the NIHR or the UK Department of Health.

Data Sharing Statement

No additional data available at this time.

For peer review only

For peer review only - http://bmjopen.bmj.com/site/about/guidelines.xhtml

References

1. Kirchhof P, Benussi S, Kotecha D, et al. 2016 ESC Guidelines for the management of atrial fibrillation developed in collaboration with EACTS. *Eur Heart J* 2016;37:2893-962.

2. Chugh SS, Havmoeller R, Narayanan K, et al. Worldwide epidemiology of atrial fibrillation: a Global Burden of Disease 2010 Study. *Circulation* 2014;129:837-47.

3. Krijthe BP, Kunst A, Benjamin EJ, et al. Projections on the number of individuals with atrial fibrillation in the European Union, from 2000 to 2060. *Eur Heart J* 2013;34:2746-51.

4. Lane DA, Skøjth F, Larsen TB, Lip GYH, Kotecha D. Temporal trends in atrial fibrillation incidence, comorbidity and mortality: comprehensive linked data from primary care. *J Am Heart Assoc* 2017; 10.1161/JAHA.116.005155.

5. Chiang CE, Naditch-Brule L, Murin J, et al. Distribution and risk profile of paroxysmal, persistent, and permanent atrial fibrillation in routine clinical practice: insight from the real-life global survey evaluating patients with atrial fibrillation international registry. *Circ Arrhythm Electrophysiol* 2012;5:632-9.

6. Kotecha D, Piccini JP. Atrial fibrillation in heart failure: what should we do? *Eur Heart J* 2015;36:3250-7.

7. Kotecha D, Lam CS, Van Veldhuisen DJ, Van Gelder IC, Voors AA, Rienstra M. Heart failure with preserved ejection fraction and atrial fibrillation: Vicious twins. *J Am Coll Cardiol* 2016;68:2217-28.

8. Kotecha D, Banerjee A, Lip GY. Increased stroke risk in atrial fibrillation patients with heart failure: does ejection fraction matter? *Stroke* 2015;46:608-9.

9. Christiansen CB, Olesen JB, Gislason G, Lock-Hansen M, Torp-Pedersen C. Cardiovascular and non-cardiovascular hospital admissions associated with atrial fibrillation: a Danish nationwide, retrospective cohort study. *BMJ Open* 2013;3.

10. Ambrosy AP, Fonarow GC, Butler J, et al. The global health and economic burden of hospitalizations for heart failure: lessons learned from hospitalized heart failure registries. *J Am Coll Cardiol* 2014;63:1123-33.

11. Kotecha D, Chudasama R, Lane DA, Kirchhof P, Lip GY. Atrial fibrillation and heart

For peer review only - http://bmjopen.bmj.com/site/about/guidelines.xhtml

failure due to reduced versus preserved ejection fraction: A systematic review and meta-analysis of death and adverse outcomes. *Int J Cardiol* 2016;203:660-6.

12. Kirchhof P, Breithardt G, Bax J, et al. A roadmap to improve the quality of atrial fibrillation management: proceedings from the fifth Atrial Fibrillation Network/European Heart Rhythm Association consensus conference. *Europace* 2016;18:37-50.

13. National Institute for Health and Care Excellence. Atrial fibrillation: the management of atrial fibrillation. *NICE clinical guideline 180* 2014; Accessed 15/09/2016; http://www.nice.org.uk/guidance/cg180/.

14. January CT, Wann LS, Alpert JS, et al. 2014 AHA/ACC/HRS guideline for the management of patients with atrial fibrillation: executive summary: a report of the American College of Cardiology/American Heart Association Task Force on practice guidelines and the Heart Rhythm Society. *Circulation* 2014;130:2071-104.

15. Kotecha D, Kirchhof P. Rate and rhythm control have comparable effects on mortality and stroke in atrial fibrillation but better data are needed. *Evid Based Med* 2014;19:222-3.

16. Segal JB, McNamara RL, Miller MR, et al. The evidence regarding the drugs used for ventricular rate control. J Fam Practice, 2000:47-59.

17. Nikolaidou T, Channer KS. Chronic atrial fibrillation: a systematic review of medical heart rate control management. *Postgrad Med J* 2009;85:303-12.

18. Farshi R, Kistner D, Sarma JSM, Longmate JA, Singh BN. Ventricular rate control in chronic atrial fibrillation during daily activity and programmed exercise: a crossover open-label study of five drug regimens. *J Am Coll Cardiol* 1999;33:304-10.

19. Ulimoen SR, Enger S, Carlson J, et al. Comparison of four single-drug regimens on ventricular rate and arrhythmia-related symptoms in patients with permanent atrial fibrillation. *Am J Cardiol* 2013;111:225-30.

20. Kotecha D, Holmes J, Krum H, et al. Efficacy of beta blockers in patients with heart failure plus atrial fibrillation: an individual-patient data meta-analysis. *Lancet* 2014;384:2235-43.

21. Ziff OJ, Lane DA, Samra M, et al. Safety and efficacy of digoxin: systematic review and

For peer review only

meta-analysis of observational and controlled trial data. *BMJ* 2015;351:h4451.

22. Wyse DG, Waldo AL, DiMarco JP, et al. A comparison of rate control and rhythm control in patients with atrial fibrillation. *N Engl J Med* 2002;347:1825-33.

23. Van Gelder IC, Hagens VE, Bosker HA, et al. A Comparison of Rate Control and Rhythm Control in Patients with Recurrent Persistent Atrial Fibrillation. *N Engl J Med* 2002;347:1834-40.

24. de Denus S, Sanoski CA, Carlsson J, Opolski G, Spinler SA. Rate vs rhythm control in patients with atrial fibrillation: a meta-analysis. *Arch Intern Med* 2005;165:258-62.

25. Chatterjee S, Sardar P, Lichstein E, Mukherjee D, Aikat S. Pharmacologic rate versus rhythm-control strategies in atrial fibrillation: an updated comprehensive review and meta-analysis. *PACE* 2013;36:122-33.

26. Al-Khatib SM, Allen LaPointe NM, Chatterjee R, et al. Rate- and rhythm-control therapies in patients with atrial fibrillation: a systematic review. *Ann Intern Med* 2014;160:760-73.

27. Roy D, Talajic M, Nattel S, et al. Rhythm Control versus Rate Control for Atrial Fibrillation and Heart Failure. *N Engl J Med* 2008;358:2667-77.

28. Kong MH, Shaw LK, O'Connor C, Califf RM, Blazing MA, Al-Khatib SM. Is rhythm-control superior to rate-control in patients with atrial fibrillation and diastolic heart failure? *Ann Noninvasive Electrocardiol* 2010;15:209-17.

29. Corley SD, Epstein AE, DiMarco JP, et al. Relationships between sinus rhythm, treatment, and survival in the Atrial Fibrillation Follow-Up Investigation of Rhythm Management (AFFIRM) Study. *Circulation* 2004;109:1509-13.

30. Wazni O, Wilkoff B, Saliba W. Catheter Ablation for Atrial Fibrillation. *N Engl J Med* 2011;365:2296-304.

31. Jones DG, Haldar SK, Hussain W, et al. A randomized trial to assess catheter ablation versus rate control in the management of persistent atrial fibrillation in heart failure. *J Am Coll Cardiol* 2013;61:1894-903.

32. Kirchhof P, Ammentorp B, Darius H, et al. Management of atrial fibrillation in seven European countries after the publication of the 2010 ESC Guidelines on atrial

For peer review only

For peer review only - http://bmjopen.bmj.com/site/about/guidelines.xhtml

fibrillation: primary results of the PREvention oF thromboemolic events--European Registry in Atrial Fibrillation (PREFER in AF). *Europace* 2014;16:6-14.

33. Senoo K, Lip GY, Lane DA, Buller HR, Kotecha D. Residual risk of stroke and death in anticoagulated patients according to the type of atrial fibrillation: AMADEUS Trial. *Stroke* 2015;46:2523-8.

34. Van Gelder IC, Groenveld HF, Crijns HJGM, et al. Lenient versus Strict Rate Control in Patients with Atrial Fibrillation. *N Engl J Med* 2010;362:1363-73.

35. Groenveld HF, Crijns HJGM, Van den Berg MP, et al. The Effect of Rate Control on Quality of Life in Patients With Permanent Atrial Fibrillation: Data From the RACE II (Rate Control Efficacy in Permanent Atrial Fibrillation II) Study. *J Am Coll Cardiol* 2011;58:1795-803.

36. Groenveld HF, Tijssen JG, Crijns HJ, et al. Rate control efficacy in permanent atrial fibrillation: successful and failed strict rate control against a background of lenient rate control: data from RACE II (Rate Control Efficacy in Permanent Atrial Fibrillation). *J Am Coll Cardiol* 2013;61:741-8.

37. Van Gelder IC, Wyse DG, Chandler ML, et al. Does intensity of rate-control influence outcome in atrial fibrillation? An analysis of pooled data from the RACE and AFFIRM studies. *Europace* 2006;8:935-42.

38. Cooper HA, Bloomfield DA, Bush DE, et al. Relation between achieved heart rate and outcomes in patients with atrial fibrillation (from the Atrial Fibrillation Follow-up Investigation of Rhythm Management [AFFIRM] Study). *Am J Cardiol* 2004;93:1247-53.

39. Groenveld HF, Crijns HJ, Rienstra M, et al. Does intensity of rate control influence outcome in persistent atrial fibrillation? Data of the RACE study. *Am Heart J* 2009;158:785-91.

40. Kotecha D, Flather MD, Altman DG, et al. Heart rate, heart rhythm and prognostic benefits of beta-blockers in heart failure: individual patient-data meta-analysis. *J Am Coll Cardiol* 2017; 10.1016/j.jacc.2017.04.001.

41. Cullington D, Goode KM, Zhang J, Cleland JG, Clark AL. Is heart rate important for patients with heart failure in atrial fibrillation? *JACC Heart Fail* 2014;2:213-20.

For peer review only - http://bmjopen.bmj.com/site/about/guidelines.xhtml

42. Steg PG, Alam S, Chiang C-E, et al. Symptoms, functional status and quality of life in patients with controlled and uncontrolled atrial fibrillation: data from the RealiseAF cross-sectional international registry. *Heart* 2012;98:195-201.

43. Nabauer M, Gerth A, Limbourg T, et al. The Registry of the German Competence NETwork on Atrial Fibrillation: patient characteristics and initial management. *Europace* 2009;11:423-34.

44. Lip GY, Laroche C, Dan GA, et al. A prospective survey in European Society of Cardiology member countries of atrial fibrillation management: baseline results of EURObservational Research Programme Atrial Fibrillation (EORP-AF) Pilot General Registry. *Europace* 2014;16:308-19.

45. Kotecha D, Manzano L, Krum H, et al. Effect of age and sex on efficacy and tolerability of beta blockers in patients with heart failure with reduced ejection fraction: individual patient data meta-analysis. *BMJ* 2016;353:i1855.

46. The Digitalis Investigation Group. The effect of digoxin on mortality and morbidity in patients with heart failure. *N Engl J Med* 1997;336:525-33.

47. Koh KK, Kwon KS, Park HB, et al. Efficacy and safety of digoxin alone and in combination with low-dose diltiazem or betaxolol to control ventricular rate in chronic atrial fibrillation. *Am J Cardiol* 1995;75:88-90.

48. Lewis RV, McMurray J, McDevitt DG. Effects of atenolol, verapamil, and xamoterol on heart rate and exercise tolerance in digitalised patients with chronic atrial fibrillation. *J Cardiovasc Pharmacol* 1989;13:1-6.

49. Tsuneda T, Yamashita T, Fukunami M, et al. Rate control and quality of life in patients with permanent atrial fibrillation: the Quality of Life and Atrial Fibrillation (QOLAF) Study. *Circ J* 2006;70:965-70.

50. Ulimoen SR, Enger S, Carlson J, et al. Comparison of four single-drug regimens on ventricular rate and arrhythmia-related symptoms in patients with permanent atrial fibrillation. *Am J Cardiol* 2013;111:225-30.

51. Ulimoen SR, Enger S, Pripp AH, et al. Calcium channel blockers improve exercise capacity and reduce N-terminal Pro-B-type natriuretic peptide levels compared with beta-blockers in patients with permanent atrial fibrillation. *Eur Heart J* 2014;35:517-

For peer review only - http://bmjopen.bmj.com/site/about/guidelines.xhtml

24.

52. Lewis RV, Irvine N, McDevitt DG. Relationships between heart rate, exercise tolerance and cardiac output in atrial fibrillation: the effects of treatment with digoxin, verapamil and diltiazem. *Eur Heart J* 1988;9:777-81.

53. McMurray JJ, Adamopoulos S, Anker SD, et al. ESC Guidelines for the diagnosis and treatment of acute and chronic heart failure 2012: The Task Force for the Diagnosis and Treatment of Acute and Chronic Heart Failure 2012 of the European Society of Cardiology. Developed in collaboration with the Heart Failure Association (HFA) of the ESC. *Eur Heart J* 2012;33:1787-847.

54. Elkayam U. Calcium channel blockers in heart failure. *Cardiology* 1998;89 Suppl 1:38-46.

55. The effect of diltiazem on mortality and reinfarction after myocardial infarction. The Multicenter Diltiazem Postinfarction Trial Research Group. *N Engl J Med* 1988;319:385-92.

56. Goldstein RE, Boccuzzi SJ, Cruess D, Nattel S. Diltiazem increases late-onset congestive heart failure in postinfarction patients with early reduction in ejection fraction. *Circulation* 1991;83:52-60.

57. The Danish Study Group on Verapamil in Myocardial Infarction. Secondary prevention with verapamil after myocardial infarction. *Am J Cardiol* 1990;66:33-40.

58. Khand AU, Rankin AC, Martin W, Taylor J, Gemmell I, Cleland JG. Carvedilol alone or in combination with digoxin for the management of atrial fibrillation in patients with heart failure? *J Am Coll Cardiol* 2003;42:1944-51.

59. Partanen J, Heikkila J, Pellinen T, Nieminen MS. Effect of digoxin on the heart in normal subjects: influence of isometric exercise and autonomic blockade: a noninvasive study. *Br J Clin Pharmacol* 1988;25:331-40.

60. Dernellis JM, Panaretou MP. Effects of digoxin on left atrial function in heart failure. *Heart* 2003;89:1308-15.

61. Giunta A, Maione S, Arnese MR, et al. Effects of intravenous digoxin on pulmonary venous and transmitral flows in patients with chronic heart failure of different degrees. *Clin Cardiol* 1995;18:27-33.

For peer review only

62. Kotecha D. Magnesium for Atrial Fibrillation, Myth or Magic? *Circ Arrhythm Electrophysiol* 2016;9.

63. Thrall G, Lane D, Carroll D, Lip GYH. Quality of Life in Patients with Atrial Fibrillation: A Systematic Review. *Am J Med* 2006;119:448.e1-19.

64. Rienstra M, Lubitz SA, Mahida S, et al. Symptoms and Functional Status of Patients With Atrial Fibrillation: State of the Art and Future Research Opportunities. *Circulation* 2012;125:2933-43.

65. Pepine CJ. Effects of pharmacologic therapy on health-related quality of life in elderly patients with atrial fibrillation: a systematic review of randomized and nonrandomized trials. *Clin Med Insights Cardiol* 2013;7:1-20.

66. Hagens VE, Ranchor AV, Van Sonderen E, et al. Effect of rate or rhythm control on quality of life in persistent atrial fibrillation: Results from the Rate Control Versus Electrical Cardioversion (RACE) study. *J Am Coll Cardiol* 2004;43:241-47.

67. Grönefeld GC, Lilienthal J, Kuck K-H, Hohnloser SH. Impact of rate versus rhythm control on quality of life in patients with persistent atrial fibrillation: Results from a prospective randomized study. *Eur Heart J* 2003;24:1430-36.

68. Kotecha D, Ahmed A, Calvert M, Lencioni M, Terwee CB, Lane DA. Patient-reported outcomes for quality of life assessment in atrial fibrillation: A systematic review of measurement properties. *PLoS ONE* 2016;11:e0165790.

69. Kotecha D, Mohamed M, Shantsila E, Popescu BA, Steeds RP. Is echocardiography valid and reproducible in patients with atrial fibrillation? A systematic review. *Europace* 2017; 10.1093/europace/eux027.

70. Ziff OJ, Kotecha D. Digoxin: The good and the bad. *Trends Cardiovasc Med* 2016;26:585-95.

71. Dasgupta A. Impact of interferences including metabolite crossreactivity on therapeutic drug monitoring results. *Ther Drug Monit* 2012;34:496-506.

72. Chan AW, Tetzlaff JM, Gotzsche PC, et al. SPIRIT 2013 explanation and elaboration: guidance for protocols of clinical trials. *BMJ* 2013;346:e7586.

73. Calvert M, Kyte D, von Hildebrand M, King M, Moher D. Putting patients at the heart of

For peer review only

For peer review only - http://bmjopen.bmj.com/site/about/guidelines.xhtml

health-care research. *Lancet* 2015;385:1073-74.

74. Calvert M, Kyte D, Duffy H, et al. Patient-reported outcome (PRO) assessment in clinical trials: a systematic review of guidance for trial protocol writers. *PLoS One* 2014;9:e110216.

75. Kyte D, Duffy H, Fletcher B, et al. Systematic evaluation of the patient-reported outcome (PRO) content of clinical trial protocols. *PLoS One* 2014;9:e110229.

76. Ware JE, Gandek B. Overview of the SF-36 Health Survey and the International Quality of Life Assessment (IQOLA) Project. *J Clin Epidemiol* 1998;51:903-12.

77. Gandek B, Sinclair SJ, Kosinski M, Ware JE, Jr. Psychometric evaluation of the SF-36 health survey in Medicare managed care. *Health Care Financ Rev* 2004;25:5-25.

78. Herdman M, Gudex C, Lloyd A, et al. Development and preliminary testing of the new five-level version of EQ-5D (EQ-5D-5L). *Qual Life Res* 2011;20:1727-36.

79. Devlin NJ, Krabbe PF. The development of new research methods for the valuation of EQ-5D-5L. *Eur J Health Econ* 2013;14 Suppl 1:S1-3.

80. Janssen MF, Pickard AS, Golicki D, et al. Measurement properties of the EQ-5D-5L compared to the EQ-5D-3L across eight patient groups: a multi-country study. *Qual Life Res* 2013;22:1717-27.

81. Spertus J, Dorian P, Bubien R, et al. Development and validation of the Atrial Fibrillation Effect on QualiTy-of-Life (AFEQT) Questionnaire in patients with atrial fibrillation. *Circ Arrhythm Electrophysiol* 2011;4:15-25.

82. Dorian P, Burk C, Mullin CM, et al. Interpreting changes in quality of life in atrial fibrillation: How much change is meaningful? *Am Heart J* 2013;166:381-87.e8.

83. Wynn GJ, Todd DM, Webber M, et al. The European Heart Rhythm Association symptom classification for atrial fibrillation: validation and improvement through a simple modification. *Europace* 2014;16:965-72.

84. Carlsson J, Miketic S, Windeler J, et al. Randomized trial of rate-control versus rhythm-control in persistent atrial fibrillation: the Strategies of Treatment of Atrial Fibrillation (STAF) study. *J Am Coll Cardiol* 2003;41:1690-6.

For peer review only - http://bmjopen.bmj.com/site/about/guidelines.xhtml

85. Hohnloser SH, Kuck KH, Lilienthal J. Rhythm or rate control in atrial fibrillation--Pharmacological Intervention in Atrial Fibrillation (PIAF): a randomised trial. *Lancet* 2000;356:1789-94.

86. Opolski G, Torbicki A, Kosior DA, et al. Rate control vs rhythm control in patients with nonvalvular persistent atrial fibrillation: the results of the Polish How to Treat Chronic Atrial Fibrillation (HOT CAFE) Study. *Chest* 2004;126:476-86.

87. Vora A, Karnad D, Goyal V, et al. Control of heart rate versus rhythm in rheumatic atrial fibrillation: a randomized study. *J Cardiovasc Pharmacol Ther* 2004;9:65-73.

For peer review only

For peer review only - http://bmjopen.bmj.com/site/about/guidelines.xhtml

Table 1: The RATE-AF trial – Information for Patients

**About atrial fibrillation**
Atrial fibrillation is a common heart condition that leads to an irregular and often rapid heart rate. Atrial fibrillation causes 1 in 4 strokes, and patients have frequent hospital admissions and a higher risk of dying. In addition, atrial fibrillation makes many patients feel unwell, with reduced quality of life.
**What is the purpose of the trial?**
Atrial fibrillation usually requires medication to control heart rate, but we currently don't know which medication is better for patients. The aim of this study is to find out which of two treatments improves quality of life and the function of the heart, digoxin or bisoprolol (a beta-blocker).
**What will happen in the trial?**
The RATE-AF trial is designed to compare two approaches for control of heart rate, based on initial treatment with either digoxin or beta-blockers, medications which are commonly used by doctors. The main objective of the trial is to research the effects of treatment on quality of life in patients with atrial fibrillation. We will also test whether quality of life questionnaires respond to changes in symptoms experienced by patients, how we use ultrasound to look at the function of the heart, and develop new markers in the blood to personalise treatment.
**More information**
RATE-AF trial video: https://www.youtube.com/watch?v=4oxe8AcVo0E or search 'rateaf' in YouTube.<br>Patient information (British Heart Foundation): https://www.bhf.org.uk/heart-health/conditions/atrial-fibrillation.

For peer review only - http://bmjopen.bmj.com/site/about/guidelines.xhtml

Table 2: Outcomes and objectives of the RATE-AF trial

**Primary outcome:**
Comparison of two strategies for rate control on patient-reported quality of life, based on initial use of digoxin versus beta-blocker therapy, with a predefined focus on physical well-being using the SF-36 physical component summary at six months.
**Secondary outcomes:**
Patient-reported quality of life at six and twelve months, including SF-36 global and domain-specific scores, EQ-5D-5L summary index and visual analogue scale, and AFEQT overall score.
Echocardiographic left-ventricular function at 12 months, including LVEF and diastolic function (E/e' and composite of diastolic indices).
Functional assessment at 6 and 12 months, including six-minute walking distance and change in EHRA class.
Change in BNP levels at 6 months.
Change in heart rate from baseline and group comparison using 24-hour ambulatory ECG at end of uptitration.
**Feasibility assessment:**
Successful methods for recruitment across primary and secondary care.
Key issues that affect retention of participants, such as convenience, compliance and cross-over.
Drug discontinuation rate and adverse reactions leading to drug discontinuation.
Therapy-induced requirement for additional treatment (e.g. pacemaker implantation).
Population-specific standard deviations and proportions to enable sample size calculation for a future trial.
Assessment of unplanned hospital admissions and cardiovascular outcomes.
**Exploratory objectives:**
Assessment of the validity and reproducibility of echocardiographic measures in patients with AF.
Correlation of baseline measures, including quality of life questionnaires and unblinded baseline investigations such as quality of life, BNP, LVEF, E/e', EHRA class, intracellular biomarkers and heart rate.
Impact of therapy on intracellular sodium and calcium concentration and cardiotonic steroid levels as biomarkers of cellular response.
Impact of combination therapy on outcomes.
Change in cognitive function at twelve months.
Qualitative research of patient-reported quality of life using focus groups to explore patient acceptability, optimal delivery methods and responsiveness.
Correlation of serum digoxin concentration with change in quality of life and intracellular methods.
Cost-consequence economic analysis from an NHS healthcare perspective.

AF, Atrial Fibrillation; AFEQT, Atrial Fibrillation Effect on QualiTy-of-life questionnaire; BNP, B-type natriuretic peptide; ECG, electrocardiogram; EHRA, European Heart Rhythm Association functional class; EQ-5D-5L, EuroQol five dimensions five level questionnaire; LVEF, left ventricular ejection fraction; NHS, National Health Service; SF-36, Short Form (36) Health Survey.

For peer review only - http://bmjopen.bmj.com/site/about/guidelines.xhtml

Table 3: Patient-reported quality of life questionnaires used in RATE-AF

Questionnaire	Details	Advantages and disadvantages
SF-36<br>Short Form (36) Health Survey [76]	Generic instrument with 4-week recall period in eights domains (vitality, physical functioning, bodily pain, general health perceptions, physical role functioning, emotional role functioning, social role functioning and mental health).<br>11 subdivided questions, each recorded with a Likert scale.<br>Scoring: Each response is given a numerical value (0 to 100, with 100 representing the best level of functioning possible), which are averaged across each domain.	Extensively validated across a wide variety of conditions and the elderly.[77]<br>Not specific to AF and hence other comorbidities may dominate responses.<br>Requires a license fee.
EQ-5D-5L<br>EuroQol five dimensions five level questionnaire [78 79]	Generic instrument about today's health with a five-answer scale in five domains (mobility, self-care, usual activities, pain/discomfort and anxiety/depression).<br>Scoring: Each question is scored (1 to 5, with 1 representing the best health). The overall profile can be indexed to country specific value sets giving a continuous value.<br>Also includes a visual analogue scale denoting current health perception (0 to 100 scale, with 100 representing the best health the patient can imagine).	Simple questionnaire that is quick to complete and includes a visual scale.<br>Extensive utilisation, particularly for heath economic assessment, with improvement discrimination over prior versions.[80]<br>Not specific to AF and hence other comorbidities may dominate responses.
AFEQT<br><br>Atrial Fibrillation Effect on QualiTy-of-life questionnaire [81]	AF-specific quality of life instrument with 4-week recall period in domains relating to symptoms, daily activities and treatment.<br>20 questions (18 on health-related quality and life and 2 on treatment satisfaction), each recorded with a 7-point Likert scale.<br>Scoring: Responses to the 18 questions are summed and converted to a continuous score (0 to 100, with 100 corresponding to no patient concerns nor disability due to AF). Component domains are scored in a similar way.	Specific to the impact of AF on quality of life.<br>Better than other AF-specific tools in a systematic review of methodological/psychometric assessment.[68]<br>Limited validation as yet in comparison to generic tools[82 83], particularly for clinical responsiveness.<br>License fee may apply.

For peer review only

For peer review only - http://bmjopen.bmj.com/site/about/guidelines.xhtml

Figure legends

Figure 1: Evidenced-based summary for management of AF

Summary of evidence for main components of clinical management, highlighting paucity of robust data for key issues regarding rate control therapy. RCT, randomised controlled trial; LV, left-ventricular; NOAC, novel oral anticoagulants.

Figure 2: Hospitalisation in rate versus rhythm control trials

Meta-analysis of hospitalisation in the six largest rate versus rhythm control trials, excluding hospital visits for cardioversion procedures, where applicable. Studies are pooled with a random-effects model. Significant heterogeneity was identified, with an I^2 value of 66.8% (p=0.01). Grey boxes represent the comparative weight of the study.

STAF, Strategies of Treatment of Atrial Fibrillation study (cardioversion/AAD versus rate control in persistent AF)[84]; PIAF, Pharmacological Intervention in Atrial Fibrillation trial (amiodarone/cardioversion versus diltiazem in persistent AF)[85]; HOT CAFE, How to Treat Chronic Atrial Fibrillation study (cardioversion/AAD versus rate control in persistent AF)[86]; AF-CHF, Atrial Fibrillation and Congestive Heart Failure trial (cardioversion/AAD versus rate control in paroxysmal/persistent AF with LVEF ≤35%)[27]; CRAAFT, Control of Rate versus Rhythm in rheumatic Atrial Fibrillation Trial (cardioversion/amiodarone versus diltiazem in persistent AF due to rheumatic heart disease)[87]; AFFIRM, Atrial Fibrillation Follow-up Investigation of Rhythm Management study (AAD/cardioversion versus rate control in paroxysmal/persistent AF).[22]

Figure 3: RATE-AF trial schema

Trial flowchart, including major endpoints and inclusion/exclusion criteria.

For peer review only - http://bmjopen.bmj.com/site/about/guidelines.xhtml

Appendix: RATE-AF trial protocol

Please see attached file.

For peer review only

For peer review only - http://bmjopen.bmj.com/site/about/guidelines.xhtml

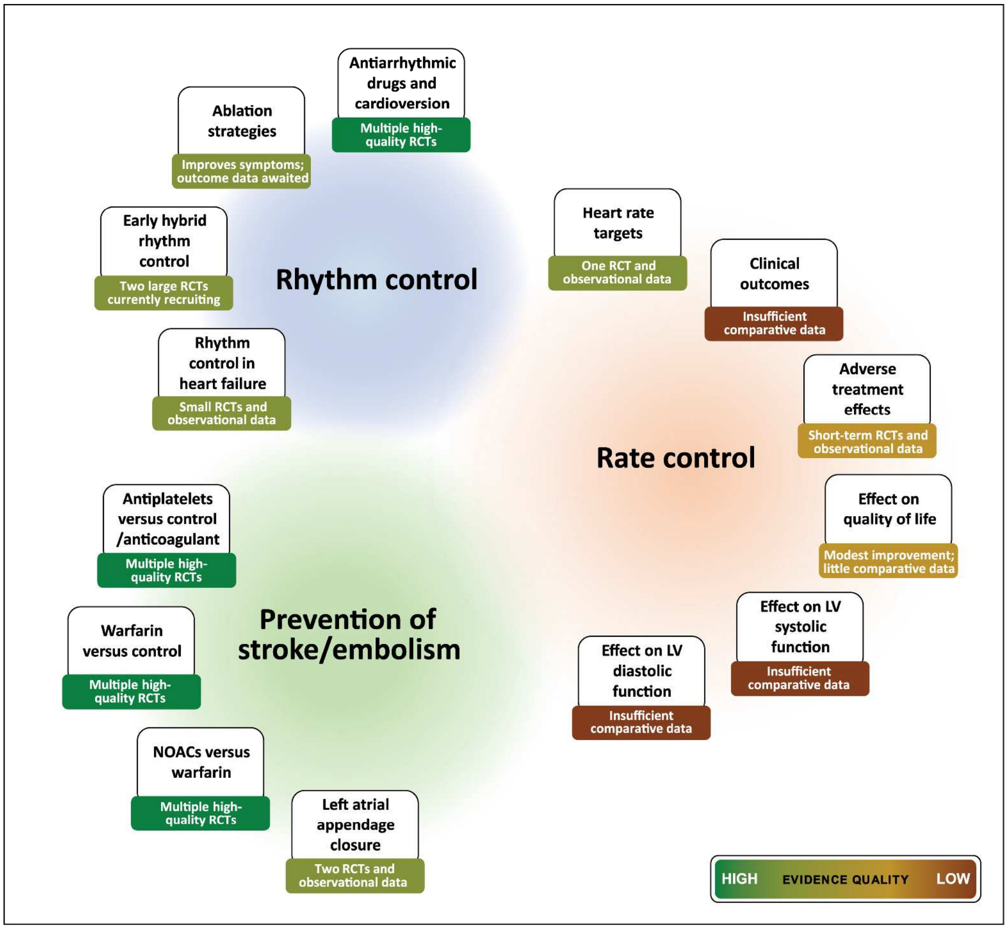

Figure 1: Evidenced-based summary for management of AF
Summary of evidence for main components of clinical management, highlighting paucity of robust data for key issues regarding rate control therapy. RCT, randomised controlled trial; LV, left-ventricular; NOAC, novel oral anticoagulants.

291x267mm (300 x 300 DPI)

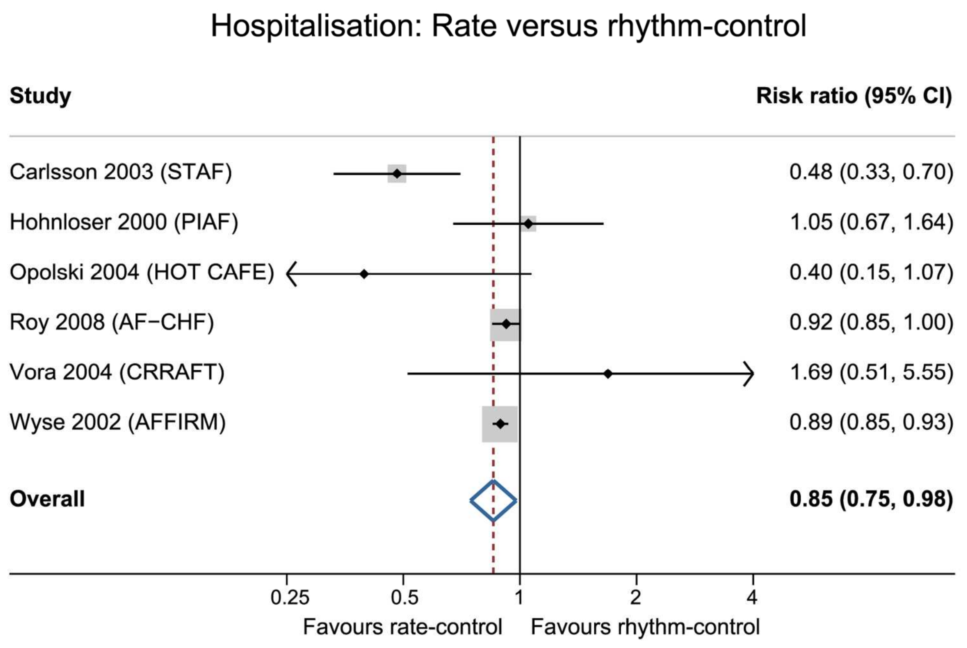

Figure 2: Hospitalisation in rate versus rhythm control trials
Meta-analysis of hospitalisation in the six largest rate versus rhythm control trials, excluding hospital visits for cardioversion procedures, where applicable. Studies are pooled with a random-effects model. Significant heterogeneity was identified, with an I2 value of 66.8% (p=0.01). Grey boxes represent the comparative weight of the study.
STAF, Strategies of Treatment of Atrial Fibrillation study (cardioversion/AAD versus rate control in persistent AF)81; PIAF, Pharmacological Intervention in Atrial Fibrillation trial (amiodarone/cardioversion versus diltiazem in persistent AF)82; HOT CAFE, How to Treat Chronic Atrial Fibrillation study (cardioversion/AAD versus rate control in persistent AF)83; AF-CHF, Atrial Fibrillation and Congestive Heart Failure trial (cardioversion/AAD versus rate control in paroxysmal/persistent AF with LVEF ≤35%)27; CRAAFT, Control of Rate versus Rhythm in rheumatic Atrial Fibrillation Trial (cardioversion/amiodarone versus diltiazem in persistent AF due to rheumatic heart disease)84; AFFIRM, Atrial Fibrillation Follow-up Investigation of Rhythm Management study (AAD/cardioversion versus rate control in paroxysmal/persistent AF).22

157x106mm (300 x 300 DPI)

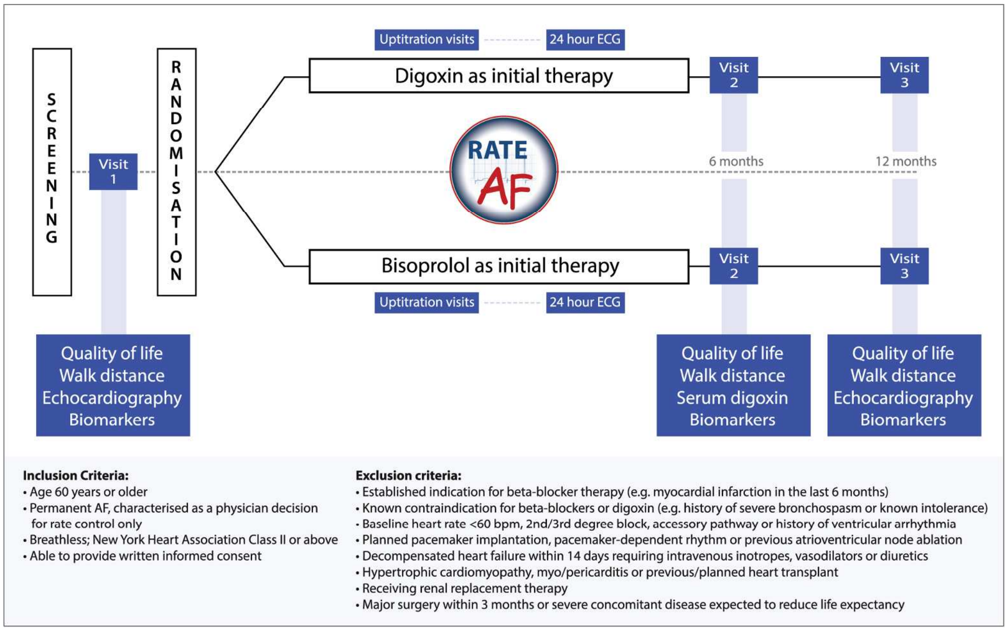

Figure 3: RATE-AF trial schema
Trial flowchart, including major endpoints and inclusion/exclusion criteria.

183x114mm (300 x 300 DPI)

Evaluating different rate control therapies in permanent atrial fibrillation: A prospective, randomised, open-label, blinded endpoint trial comparing digoxin and beta-blockers as initial rate control therapy

RAte control Therapy Evaluation in Atrial Fibrillation:

RATE-AF

RATE-AF TRIAL PROTOCOL

Version 1.0, 23rd March 2016

**Sponsor:**	**University of Birmingham**
**Chief Investigator:**	**Dr Dipak Kotecha**
**Coordinating Unit:**	**Birmingham Clinical Trials Unit**
**Funder:**	**National Institute for Health Research (NIHR) Career Development Fellowship**

**ISRCTN:**	**TBC**
**EudraCT No.:**	**2015-005043-13**
**REC Ref. No.:**	**TBC**

UNIVERSITY OF BIRMINGHAM

BIRMINGHAM CLINICAL TRIALS UNIT

NHS National Institute for Health Research

For peer review only

For peer review only - http://bmjopen.bmj.com/site/about/guidelines.xhtml

TRIAL COMMITTEES AND CONTACT DETAILS

Trial Management Group	
**Chief Investigator**<br>**Dr Dipak Kotecha**	**NIHR Career Development Fellow & Clinician Scientist**<br><br>Institute of Cardiovascular Sciences, University of Birmingham, The Medical School, Vincent Drive, Birmingham, B15 2TT, UK<br>Email: d.kotecha@bham.ac.uk<br>Telephone: 07974 115676
**Prof Paulus Kirchhof**	**Professor of Cardiovascular Medicine**<br><br>Institute of Cardiovascular Sciences, University of Birmingham, Institute of Biomedical Research, Vincent Drive, Birmingham B15 2TT, UK<br>Email: p.kirchhof@bham.ac.uk<br>Telephone: 0121 414 7042
**Dr Michael Griffith**	**Consultant Electrophysiologist**<br><br>University Hospitals Birmingham NHS Trust, Nuffield House, Queen Elizabeth Hospital, Birmingham, B15 2TH, UK<br>Email: michael.griffith@uhb.nhs.uk<br>Telephone: 0121 371 4038
**Prof Gregory Y H Lip**	**Professor of Cardiovascular Medicine & Director, Haemostasis Thrombosis & Vascular Biology Unit**<br><br>Institute of Cardiovascular Sciences, University of Birmingham, City Hospital, Birmingham, B18 7QH, UK<br>Email g.y.h.lip@bham.ac.uk<br>Telephone: 0121 5075080
**Prof Jonathan Townend**	**Professor of Cardiology**<br><br>University Hospitals Birmingham NHS Trust, Nuffield House, Queen Elizabeth Hospital, Birmingham, B15 2TH, UK<br>Email: john.townend@uhb.nhs.uk<br>Telephone: 0121 371 4623
**Dr Rick Steeds**	**Consultant Cardiologist and Head of Cardiac Imaging**<br><br>University Hospitals Birmingham NHS Trust, Nuffield House, Queen Elizabeth Hospital, Birmingham, B15 2TH, UK<br>Email: rick.steeds@uhb.nhs.uk<br>Telephone: 0121 371 6130
**Prof Melanie Calvert**	**Professor of Outcomes Methodology**<br><br>Institute of Applied Health Research, University of Birmingham, Birmingham, B15 2TT, UK<br>Email: m.calvert@bham.ac.uk<br>Telephone: 0121 414 8595
**Dr Susan Jowett**	**Senior Lecturer, Health Economics**<br><br>Institute of Applied Health Research, University of Birmingham, Birmingham, B15 2TT, UK<br>Email s.jowett@bham.ac.uk<br>Telephone: 0121 414 7898
**Dr Jonathan Mathers**	**Senior Lecturer, Qualitative and Mixed Methods Applied Health Research**<br><br>Institute of Applied Health Research, University of Birmingham, Birmingham, B15 2TT, UK<br>Email j.m.mathers@bham.ac.uk<br>Telephone: 0121 414 6024

For peer review only - http://bmjopen.bmj.com/site/about/guidelines.xhtml

For peer review only

Birmingham Clinical Trials Unit	
**Prof Jon Deeks**	**Professor of Biostatistics and Director, BCTU**<br><br>Birmingham Clinical Trials Unit, University of Birmingham, Birmingham, B15 2TT, UK<br>Email j.deeks@bham.ac.uk<br>Telephone: 0121 414 5328
**Dr Margaret Grant**	**Operations Manager**<br><br>Birmingham Clinical Trials Unit, University of Birmingham, Birmingham, B15 2TT, UK<br>Email m.r.grant@bham.ac.uk<br>Telephone: 0121 415 9106
**Gemma Slinn**	**Senior Trial Coordinator**<br><br>Birmingham Clinical Trials Unit, University of Birmingham, Birmingham, B15 2TT, UK<br>Email g.slinn@bham.ac.uk<br>Telephone: 0121 415 8445
**Samir Mehta**	**Statistician**<br><br>Birmingham Clinical Trials Unit, University of Birmingham, Birmingham, B15 2TT, UK<br>Email s.mehta.1@bham.ac.uk<br>Telephone: 0121 415 9117

Trial Oversight Committee

**Co-Chairs**	
**Dr Kazem Rahimi**	**Associate Professor of Cardiovascular Medicine, University of Oxford**<br>**Deputy Director, The George Institute for Global Health**<br><br>The George Institute for Global Health, University of Oxford, 34 Broad Street, Oxford OX1 3BD, UK<br>Email: kazem.rahimi@georgeinstitute.ox.ac.uk<br>Telephone: 01865 617 201
**Prof. John Camm**	**BHF Professor of Clinical Cardiology**<br><br>St George's University of London, Cranmer Terrace, London SW17 0RE, UK<br>Email: jcamm@sgul.ac.uk<br>Telephone: 0208 725 3414
**Patient Representative**	
**Mary Stanbury**	Lead PPI Representative<br>Email: dms27@btinternet.com
**On behalf of the Trial Management Group**	
**Dr Dipak Kotecha**	
**Prof. Jon Deeks**	For contact details, see Trial Management Group
**Prof. Paulus Kirchhof**	

For peer review only - http://bmjopen.bmj.com/site/about/guidelines.xhtml

RATE-AF Trial Office

For general protocol related queries and supply of trial materials:

Birmingham Clinical Trials Unit (BCTU), Institute of Applied Health Research, College of Medical & Dental Sciences, Public Health Building, University of Birmingham, Edgbaston, Birmingham B15 2TT

Telephone: 0121 415 8445
Fax: 0121 415 9135
Email: RATE-AF@trials.bham.ac.uk
Website: www.birmingham.ac.uk/RATE-AF

Randomisation

Telephone: 0800 953 0274

Website: https://www.birmingham.ac.uk/RATEAF

Safety Reporting

Fax SAE Forms to: 0121 415 9135 or 0121 415 9136

Protocol Development and Sign Off

Protocol Amendments

The following amendments and/ or administrative changes have been made to this protocol since the implementation of the first approved version

Amendment number	Date of amendment	Protocol version number	Type of amendment	Summary of amendment

For peer review only - http://bmjopen.bmj.com/site/about/guidelines.xhtml

Chief Investigator Signature Page

Trial Name: **RATE-AF**

Protocol Version Number: Version: __ __

Protocol Version Date: __ __ / __ __ __ / __ __ __ __

This protocol has been approved by:

CI Name: Dr Dipak Kotecha

Trial Role: Chief Investigator

Signature and date: ________________________ __ __ / __ __ __ / __ __ __ __

Sponsor Statement

Where the University of Birmingham takes on the sponsor role for protocol development oversight, the signing of the IRAS form by the Sponsor will serve as confirmation of the approval of this protocol.

For peer review only - http://bmjopen.bmj.com/site/about/guidelines.xhtml

Principal Investigator Signature Page

Principal Investigator:

I have read and agree to the protocol, as detailed in this document. I agree to adhere to the protocol as outlined and agree that any suggested changes to the protocol must be approved by the Trial Oversight Committee prior to seeking approval from the Research Ethics Committee and Regulatory Authority.

I am aware of my responsibilities as an Investigator under the guidelines of Good Clinical Practice (GCP), the Declaration of Helsinki, local regulations (as applicable) and the trial protocol and I agree to conduct the trial according to these guidelines and to appropriately direct and assist the staff under my control, who will be involved in the trial.

Trial Name: **RATE-AF**

Protocol Version Number: Version: __ __

Protocol Version Date: __ __ / __ __ __ / __ __ __ __

PI Name: <Enter>

Trial Role: Principal Investigator

Signature and date: ____________________________ __ __ / __ __ __ / __ __ __ __

The Principal Investigator should sign this page and return a copy to the RATE-AF Trial Office

For peer review only - http://bmjopen.bmj.com/site/about/guidelines.xhtml

Table of Contents

1 Trial Summary 13

1.1 Trial Schema 15

2 Introduction 16

2.1 Background 16

2.2 Epidemiology and Consequences of AF 16

2.3 Rhythm-Control in AF 17

2.4 Lack of Evidence to Guide Rate-Control Therapy 17

2.5 Patient Wellbeing 19

2.6 Rationale for the RATE-AF Trial 19

3 Trial Design and Objectives 20

3.1 Hypothesis 20

3.2 Primary objective 21

3.3 Secondary objectives 21

3.4 Feasibility objectives 21

3.5 Exploratory objectives 21

4 Selection of Participants 22

4.1 Inclusion Criteria 22

4.2 Exclusion Criteria 22

5 Informed Consent Process 23

6 Enrolment and Randomisation 24

6.1 Randomisation Procedures 25

7 Trial Treatment 26

7.1 Treatment 26

7.2 Treatment Supply and Storage 26

7.3 Dosing Schedule 27

7.4 Drug Interactions and Contraindications 27

7.5 Accountability Procedures and Labelling 29

7.6 Treatment Modification 29

7.7 Assessment of Compliance 30

8 Trial Procedures and Schedule of Assessments 30

8.1 Baseline Visit 30

For peer review only - http://bmjopen.bmj.com/site/about/guidelines.xhtml

8.2 Up-Titration Visits ... 31

8.3 Visit 2, Month 6 ... 31

8.4 Visit 3, Month 12 (Final Trial Assessment) ... 32

8.5 Investigator-blinded Endpoints ... 32

8.6 Long Term Follow-Up ... 32

8.7 Withdrawal ... 33

8.8 Trial Duration ... 33

9 Trial Procedures ... 35

9.1 Procedures Defined as Standard Clinical Care ... 35

9.2 Medical History ... 35

9.3 Medication History ... 35

9.4 Physical Examination ... 36

9.5 Patient Reported Outcomes ... 36

9.5.1 Choice of Outcomes and Qualitative Research ... 36

9.5.2 Data Collection for PROMs ... 37

9.5.3 Outcome Appraisal ... 38

9.6 Transthoracic Echocardiography ... 38

9.6.1 Reproducibility and Validity of Measurements ... 38

9.6.2 Systolic LV Function ... 38

9.6.3 Diastolic LV Function ... 39

9.6.4 Left Atrial Size and Function ... 40

9.6.5 Additional Echocardiography Parameters ... 40

9.7 Laboratory Evaluations ... 40

9.7.1 Laboratory Assays ... 41

9.7.2 Cellular Response to Rate Control ... 41

9.7.3 Stored Blood Samples ... 41

9.7.4 Specimen Preparation, Handling, Storage and Shipment ... 41

9.8 Economic Evaluation ... 41

10 Pharmacovigilance ... 43

10.1 Recording and Assessment of Adverse Events ... 43

10.2 Non-Serious Adverse Events/ Adverse Reactions ... 45

10.3 Serious Adverse Events ... 45

For peer review only - http://bmjopen.bmj.com/site/about/guidelines.xhtml

10.3.1 Expected SAEs NOT to be Reported on a SAE Form ... 45

10.4 SUSARs ... 45

10.5 Development Safety Update Reports ... 46

10.6 Annual Progress Reports ... 46

10.7 Pregnancy ... 46

10.8 Reporting Urgent Safety Measures ... 46

11 Quality Control and Quality Assurance ... 47

11.1 Site Set-Up and Initiation ... 47

11.2 Central Monitoring ... 47

11.3 Audit and Inspection ... 47

11.4 Notification of Serious Breaches ... 48

11.5 Data Handling and Analysis ... 48

11.6 End of Trial ... 49

11.7 Archiving ... 49

12 Statistical Considerations ... 50

12.1 Outcome measures ... 50

12.1.1 Primary Outcome ... 50

12.1.2 Secondary Outcomes ... 50

12.1.3 Feasibility Outcomes ... 50

12.2 Power Calculations ... 51

12.3 Statistical analysis ... 51

12.3.1 Primary outcome analysis ... 52

12.3.2 Feasibility and Secondary outcomes analysis ... 52

12.3.3 Missing data and sensitivity analyses ... 52

12.3.4 Interim analyses and Stopping rules ... 52

12.4 Final analysis ... 53

13 Ethics and Regulatory Requirements ... 53

14 Oversight Committees ... 53

14.1 Trial Management Group ... 53

14.2 Trial Oversight Committee ... 54

14.3 Protocol amendments ... 54

15 Finance ... 54

For peer review only - http://bmjopen.bmj.com/site/about/guidelines.xhtml

16 Confidentiality and Data Protection 54

17 Insurance and Indemnity 55

18 Dissemination and Publication 55

19 Statement of Compliance 56

20 References 57

Appendix A: Randomised treatment arm - Digoxin

Appendix B: Randomised treatment arm - Bisoprolol

For peer review only

For peer review only - http://bmjopen.bmj.com/site/about/guidelines.xhtml

List of Abbreviations

**ABPI**	Association of the British Pharmaceutical Industry
**AE**	Adverse Event
**AF**	Atrial Fibrillation
**BCTU**	Birmingham Clinical Trials Unit
**BNP**	B-type Natriuretic Peptide
**BPM**	Beats per Minute
**CCB**	Calcium Channel Blocker
**CI**	Chief Investigator
**CMR**	Cardiac Magnetic Resonance
**CRF**	Case Report Form
**CTA**	Clinical Trial Authorisation
**CTIMP**	Clinical Trial of Medicinal Product
**DIBD**	Developmental International Birth Date
**DMC**	Data Monitoring Committee
**DSUR**	Developmental Safety Update Report
**DT**	Deceleration Time
**ECG**	Electrocardiogram
**EHRA**	European Heart Rhythm Association
**EU**	European Union
**EudraCT No.**	European Union Drug Regulating Authorities Clinical Trials Number
**GCP**	Good Clinical Practice
**GFR**	Glomerular Filtration Rate
**GP**	General Practitioner
**HF**	Heart Failure
**HR**	Hazard Ratio
**IMP**	Investigational Medicinal Product
**INR**	International Normalised Ratio
**ISRCTN**	International Standard Randomised Controlled Trial Number
**IVRT**	Isovolumic Relaxation Time
**LA**	Left-Atrial
**LV**	Left-Ventricular
**LVEDD**	Left-Ventricle End-Diastolic Dimension
**LVEDV**	Left-Ventricle End-Diastolic Volume
**LVEF**	Left-Ventricular Ejection Fraction

For peer review only

For peer review only - http://bmjopen.bmj.com/site/about/guidelines.xhtml

**LVESD**	Left-Ventricle End-Systolic Dimension
**LVESV**	Left-Ventricle End-Systolic Volume
**LVSD**	Left-Ventricular Systolic Dysfunction
**MHRA**	Medicines and Healthcare Products Regulatory Agency
**MREC**	Main Research Ethics Committee
**NHS**	National Health Service
**NICE**	National Institute of Clinical Excellence
**NOAC**	Novel Oral Anticoagulants
**NYHA**	New York Health Association
**PEF**	Preserved Ejection Fraction
**PI**	Principal Investigator
**PIC**	Patient Identification Centre
**PIL**	Participant Information Leaflet
**PROBE**	Prospective Randomised Open Blinded End-point
**QALY**	Quality-Adjusted Life Year
**QoL**	Quality of Life
**R&D**	Research and Development
**RCT**	Randomised Controlled Trial
**REC**	Research Ethics Committee
**SAE**	Serious Adverse Event
**SAR**	Serious Adverse Reaction
**SD**	Standard Deviation
**SmPC**	Summary of Product Characteristics
**SUSAR**	Suspected Unexpected Serious Adverse Reaction
**TAPSE**	Tricuspid Annular Plane Systolic Excursion
**TDI**	Tissue Doppler Imaging
**TMF**	Trial Master File
**TMG**	Trial Management Group
**TSC**	Trial Steering Committee
**UHB**	University Hospitals Birmingham
**WTCRF**	NIHR Wellcome Trust Clinical Research Facility at Queen Elizabeth Hospital, Birmingham

For peer review only - http://bmjopen.bmj.com/site/about/guidelines.xhtml

1 Trial Summary

**Title**	**Evaluating different rate control therapies in permanent atrial fibrillation: A prospective, randomised, open-label, blinded endpoint trial comparing digoxin and beta-blockers as initial rate control therapy**<br><br>**RAte control Therapy Evaluation in Atrial Fibrillation: RATE-AF**
**Acronym**	**RATE-AF**
**Trial Design and Methods**	A prospective, randomised, open-label, blinded-endpoint (PROBE) trial design. The RATE-AF trial combines hypothesis testing (quality of life, cardiac function, exercise capacity and biomarkers), evaluation of measures (validity, reproducibility and correlation of outcomes) and a feasibility study for a future clinical event trial (assessing recruitment, retention and sample size).
**Trial Medications**	Digoxin 62.5 – 250 µg od<br>Bisoprolol 1.25 – 15 mg od
**Trial Outcomes**	**Primary Outcome:**
	Patient-reported quality of life (QoL): SF-36 physical component summary score at six months
	**Secondary Outcomes:**
	Patient-reported QoL:<br>• SF-36 global and domain-specific scores at 6 and 12 months<br>• EQ-5D-5L summary index and visual analogue scale at six and twelve months<br>• AFEQT overall score at six and twelve months
	Cardiac function:<br>• Echocardiographic LVEF at 12 months<br>• Diastolic function (E/e' and composite of diastolic indices) at 12 months
	Functional assessment:<br>• Six-minute walking distance at 6 and 12 months<br>• Change in European Heart Rhythm Association (EHRA) class at 6 and 12 months
	Biomarkers:<br>• Change in B-type natriuretic peptide (BNP) levels at 6 months
	Change in heart rate using 24-hour ambulatory ECG
	**Feasibility Outcomes:**
	Recruitment target of 3 patients per week across all participating centres.
	Compliance and reasons for non-compliance
	Number of withdrawals and losses to follow-up (with reasons)
	Drug discontinuation rate and adverse reactions requiring drug discontinuation.
	Number of patients needing therapy-induced requirement for additional treatment

For peer review only - http://bmjopen.bmj.com/site/about/guidelines.xhtml

	Population-specific standard deviations (SD) and proportions:<br>• SD of SF36 physical functioning score at 6 and 12 months<br>• SD of SF36 overall score at 6 and 12 months<br>• SD of AFEQT overall score at 6 and 12 months<br>• SD of LVEF and E/e' scores at 6 and 12 months<br>• Unplanned hospitalisation admissions rates
	Cardiovascular Events (particularly mortality, thromboembolic events, myocardial infarction and cardiovascular interventions)
**Trial Duration per Participant**	12 months of trial therapy
**Planned Trial Sites**	Multiple screening sites with single site recruitment
**Total Number of Participants**	160
**Main Inclusion/ Exclusion Criteria**	**Inclusion Criteria**
	Adult patients, aged 60 years or older
	Permanent AF, characterised (at time of randomisation) as a physician decision for rate-control with no plans for cardioversion, anti-arrhythmic medication, or ablation therapy
	Symptoms of breathlessness (New York Heart Association Class II or more)
	Able to provide written, informed consent
	**Exclusion Criteria**
	Established indication for beta-blocker therapy, e.g. myocardial infarction in the last 6 months
	Known contraindications for therapy with beta-blockers or digoxin, e.g. a history of severe bronchospasm that would preclude use of beta-blockers, or known intolerance to these medications
	Baseline heart rate <60 bpm
	History of second or third-degree heart block
	Supraventricular arrhythmias associated with accessory conducting pathways (e.g. Wolff-Parkinson-White syndrome) or a history of ventricular tachycardia or fibrillation
	Planned pacemaker implantation (including cardiac resynchronisation therapy), pacemaker-dependent rhythm or history of atrioventricular node ablation
	Decompensated heart failure (evidenced by need for intravenous inotropes, vasodilators or diuretics) within 14 days prior to randomisation
	A current diagnosis of obstructive hypertrophic cardiomyopathy, myocarditis or constrictive pericarditis
	Received or on waiting list for heart transplantation
	Receiving renal replacement therapy
	Major surgery, including thoracic or cardiac surgery, within 3 months of randomisation
	Severe, concomitant non-cardiovascular disease (including malignancy) that is expected to reduce life expectancy

For peer review only - http://bmjopen.bmj.com/site/about/guidelines.xhtml

1.1 Trial Schema

Figure 1

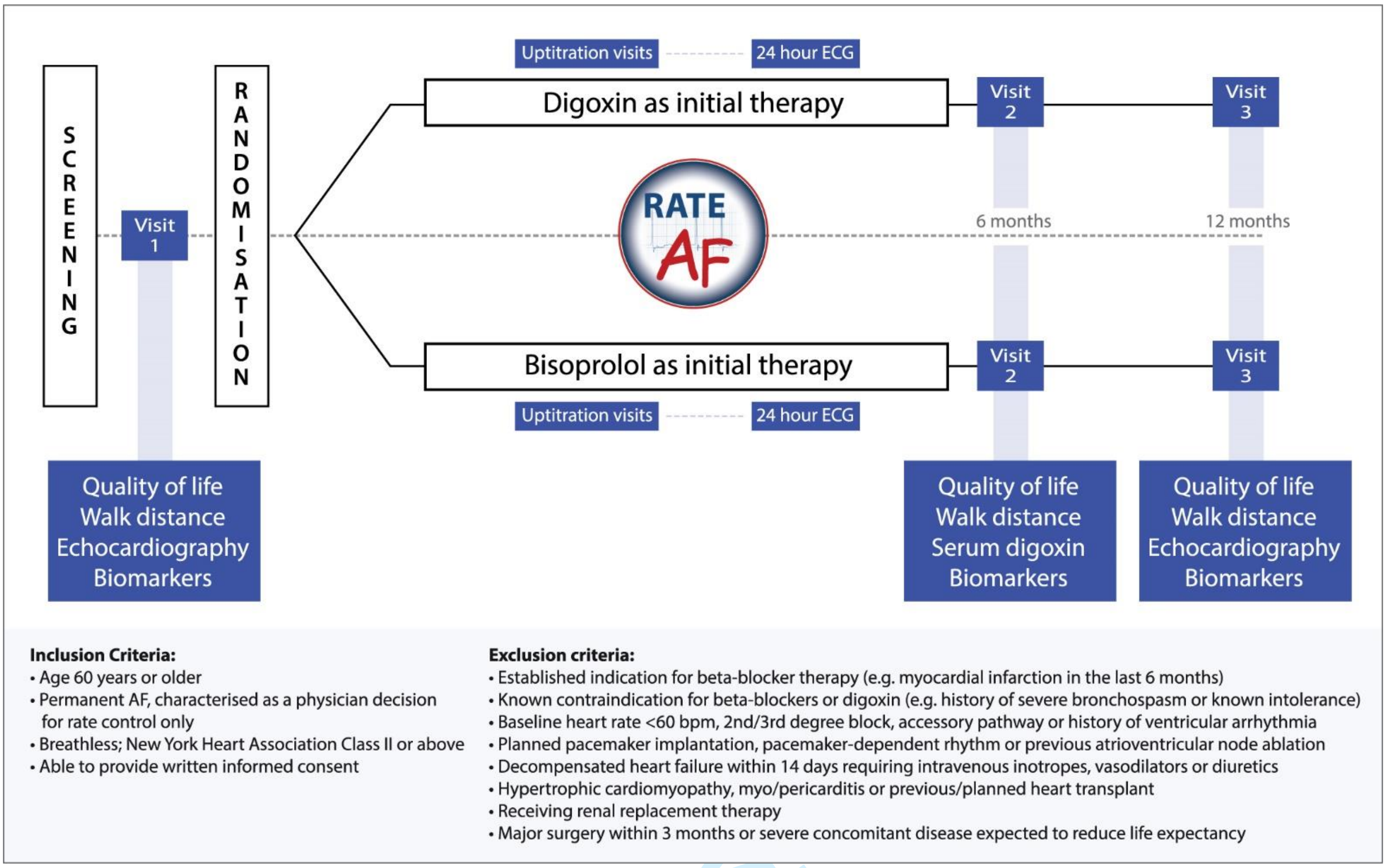

This protocol describes the **RATE-AF** trial only. The trial will be conducted in accordance with the protocol, Good Clinical Practice (GCP) and applicable regulatory requirements. Every care has been taken in the drafting of this protocol, but future amendments may be necessary, which will receive the required approvals prior to implementation.

For peer review only - http://bmjopen.bmj.com/site/about/guidelines.xhtml

2 Introduction

2.1 Background

Atrial fibrillation (AF) is an increasingly common cardiac condition that leads to a substantial burden on quality-of-life (QoL), an increased risk of cardiovascular events, hospitalisation and death, and significant healthcare costs for the NHS. In addition to anticoagulation and considerations for rhythm control therapy, most patients with AF are in need of pharmacological control of heart rate. This aspect of care has not received stringent investigation, with treatment guidelines based on small crossover studies and observational data rather than robust controlled trials.[1-3] Beta-blocker monotherapy remains the first-line option in the current NICE AF guidelines consultation document, with digoxin only for sedentary patients, although this recommendation is based on 'very low-quality evidence'.[4] The benefit of different rate-control therapies on symptoms and other intermediate outcomes (such as left-ventricular ejection fraction [LVEF] and diastolic function) are unknown, as are their effects on clinical events such as hospitalisation. This situation is unacceptable in light of the potential benefits and risk of different rate-control options in AF. It also limits our ability to personalise treatment according to patient characteristics.

The RAte control Therapy Evaluation in Atrial Fibrillation (**RATE-AF**) trial is informed by a number of in-depth systematic reviews of management and clinical outcomes in AF patients.[5-11] Taken together, this information provides a sound basis to plan a major randomised controlled trial (RCT).[12, 13] However as trials of rate-control in AF have typically been small or uncontrolled, further information is needed before designing a trial that can assess clinical outcomes. The **RATE-AF** trial will allow us to define appropriate primary and secondary outcome measures and their standard deviation in a contemporary population of patients with permanent AF. This information will allow us to estimate sample size, determination of recruitment, retention and adherence policies, and to ascertain the best methods of obtaining adverse event data and reliable economic costs for a larger trial assessing cardiovascular outcomes and hospitalisation. The **RATE-AF** trial will also be the largest RCT of its kind, allowing us to compare the effect of beta-blockers and digoxin on QoL as initial rate-control therapy in patients with permanent AF. The long-term aim of the research is to answer key questions about how to initiate therapy, stratified by relevant patient characteristics such as systolic and diastolic cardiac function, baseline symptoms and concurrent medication. The research will also define the patho-physiological mechanisms underlying AF-related symptoms, left-ventricular function and their association with adverse clinical outcomes, and to identify clinical markers for the response to different rate control therapy.

2.2 Epidemiology and Consequences of AF

AF is a common condition that is associated with increased rates of mortality and serious morbidity, including stroke, worsening of heart failure, sudden death, and reduced QoL.[1] The prevalence of AF increases with age, ranging from 0.7% in those aged 55–59 years to 17.8% in those aged above 85.[14] A doubling of both incidence and prevalence of AF is predicted in the next 20 years.[15]

For peer review only - http://bmjopen.bmj.com/site/about/guidelines.xhtml

Patients with AF are twice as likely to be hospitalised as propensity score-matched controls, with direct medical costs estimated to be 73% higher.[16] Further, AF is an independent predictor of all-cause mortality, with a two-fold adjusted increase in death.[17, 18] While most strokes in AF can be prevented by oral anticoagulation, AF patients still have high cardiovascular death rates due to sudden death or progressive heart failure.[19, 20] Patients with AF also have significantly poorer QoL[21], experiencing a variety of symptoms including lethargy, palpitations, dyspnoea, sleeping difficulties and psychosocial distress.[22, 23] In the context of patients diagnosed with heart failure, the presence of AF leads to higher rates of death and hospitalisation, independent of other risk variables or which condition comes first.[24, 25] From observational data, 40% of AF patients will be diagnosed with heart failure and vice-versa[16], representing a large and growing unmet clinical need for healthcare improvement.

2.3 Rhythm-Control in AF

Numerous large RCTs comparing rhythm-control (using arrhythmic drugs and/or cardioversion) versus rate-control have identified no significant difference in clinical outcomes in patients with persistent AF.[26-30] In a number of studies, hospitalisation rates were actually higher in those randomised to rhythm-control.[26, 29, 30] Similar findings have been shown in AF patients with heart failure[31, 32], both in those with impaired and preserved ejection fraction.[33-35] Although AF ablation is becoming increasingly popular to restore sinus rhythm, it remains a highly invasive method to improve AF-related symptoms.[36, 37] At present, European and NICE treatment guidelines recommend ablation only in symptomatic paroxysmal AF, or as a treatment option in symptomatic persistent AF that is refractory to other therapy.[3] Further trials are currently underway to determine the clinical value of prompt rhythm-control, including the Early treatment of Atrial fibrillation for Stroke prevention Trial (EAST).[38] In light of the high recurrence rate of AF (even in patients receiving intensive rhythm-control therapy), rate-control is an important part of AF management in almost all patients. Unfortunately, rate-control therapy has much less evidence underpinning its use.

2.4 Lack of Evidence to Guide Rate-Control Therapy

Rate-control in AF can be achieved with beta-blockers, non-dihydropyridine calcium-channel blockers (CCB), digoxin and their combinations. Unfortunately, little data exists to assist clinicians in choosing appropriate first-line and subsequent therapy. Current patterns of medication usage vary considerably (between and within countries). For example, in a worldwide registry, digoxin was prescribed in 2877 of 10,523 patients (27.3%), compared to 1599 of 3141 (50.9%) of patients in the German Competence NETwork on Atrial Fibrillation (AFNET).[39, 40]

Current European guidelines suggest “the choice of medication should be individualised and the dose modulated to avoid bradycardia”. This recommendation (Class 1, Level B) is based on a systematic review of trials addressing rate-control between 1983 and 1997.[41] Most of the studies included less than 50 participants (with several less than 10). The majority were low quality studies, as assessed by the risk of bias or confounding, and follow-up was typically in the order of

For peer review only - http://bmjopen.bmj.com/site/about/guidelines.xhtml

hours, days or weeks. Whilst this may be sufficient to assess an acute effect on heart-rate, it provides limited data on the longer-term effects of different treatments or the frequency of adverse reactions.

Beta-blockers are often preferred over other agents due to the prognostic benefit seen in patients with heart failure who are in sinus rhythm. However, in patients with heart failure, reduced LVEF and concomitant AF, we have shown that beta-blockers do not reduce mortality (hazard ratio 0·97, 95% CI 0.83-1.14; p=0.73) or cardiovascular hospital admissions (hazard ratio 0·91; 95% CI 0.79-1.04; p=0.15).[5] This distinctly contrasts with the significant benefit seen in patients with sinus rhythm and highlights the need for further comparative RCTs specifically in patients with AF.

The most highly cited trial comparing beta-blockers and digoxin for rate-control in chronic AF was an open-label two-week crossover study of 5 drug regimes in 12 patients.[42] Peak heart-rate after exercise was significantly higher in those taking digoxin compared to beta-blockers but there were no differences in exercise duration. In a trial of 42 patients, rate-control was improved with combination beta-blocker/digoxin therapy compared to digoxin alone, however there were similarly no differences in exercise capacity.[43] Systematic review of other small randomised studies identify no difference in exercise tolerance with beta-blockers, despite a lowering of heart-rate.[44] From observational data, such as the Atrial Fibrillation Follow-up Investigation of Rhythm Management (AFFIRM) study, more cardiac and non-cardiac adverse effects have been noted with beta-blockers than digoxin (n=67 vs. n=38).[28] In a 3-week crossover study of 60 participants, 10% withdrew during beta-blocker therapy due to adverse events.[45] Those in the beta-blocker group had a reduction in exercise capacity on cardio-pulmonary testing and a significant increase in B-type natriuretic peptide (BNP, a marker of ventricular strain) compared to patients treated with calcium-channel blockers.[46]

Only a single RCT has been published comparing digoxin and beta-blockers in patients with AF and heart failure (mean LVEF 24%, n=47).[47] Although there was a marginally-significant improvement in LVEF with carvedilol/digoxin versus placebo/digoxin, blinded withdrawal of digoxin then led to a deterioration in LVEF, accompanied by an increase in BNP. There was no difference in the number of heart-rate pauses >3 seconds or in daytime/exercise heart-rate comparing the two therapies alone.

Digoxin itself has been associated with an increased mortality in observational cohorts of AF patients[48], however careful adjustment of baseline differences reject a true excess in adverse outcomes.[49-51] In a detailed systematic review of all studies published on digoxin, we identified that confounding was the main reason that digoxin was associated with increased mortality in observational studies, and confirmed a neutral association in RCTs (risk ratio 0.99, 95% CI 0.93 to 1.05).[6] Lower rates of hospitalisation have been noted with digoxin therapy, independent of the type of heart failure[52], however the lack of randomised data versus placebo (despite widespread clinical use) makes true comparison difficult. Small RCTs comparing CCB with digoxin have been inconsistent; two have identified lower heart-rates with CCB but no significant difference in exercise capacity[42, 43], one demonstrated higher post-exercise cardiac output after

For peer review only - http://bmjopen.bmj.com/site/about/guidelines.xhtml

digoxin[53] and another showed improved exercise duration and QoL with CCB.[54] These results highlight the need for randomised data with appropriately-defined outcomes to accurately identify the benefits and risks of common therapies in patients with AF.

An example where RCT data have impacted on clinical practice is the Rate Control Efficacy in Permanent Atrial Fibrillation (RACE II) trial. This study challenged conventional wisdom that stricter control of heart-rate would allow time for diastolic filling and improve haemodynamics. In summary, 614 patients with permanent AF were randomised to strict or lenient rate-control and followed for 2-3 years.[55] There was no significant difference in the cumulative incidence of the composite primary outcome; 14.9% in the strict-control arm and 12.9% in the lenient-control group. There were also no differences in symptoms, New York Heart Association (NYHA) class or hospitalisations[55, 56], no interaction with baseline heart failure[57], and those participants achieving strict rate-control required more clinic visits and higher doses of medical therapy.[58] Current guidelines therefore suggest that lenient rate-control is acceptable, except for patients with adverse symptoms or clinical deterioration.[1] Whilst this study provides important data on the intensity of rate-control in AF, the more clinically-relevant questions of how to initiate therapy and the choice of optimal agents for individual patients remain unanswered.

2.5 Patient Wellbeing

Patient-reported outcomes are any report of a patient's health status (for example QoL) that is derived directly from the patient, without interpretation by a clinician.[59] There is limited data on the effect of pharmacological rate-control therapy on QoL and no comparative data assessing the benefit of different strategies.[22, 60] Rate-control has been associated with improved QoL scores in trials assessing rate versus rhythm-control.[61, 62] In the PIAF study, over 50% of participants randomised to calcium-channel blockers reported an improvement in health with significant benefits in the physical aspects of the SF-36.[63] A number of smaller studies have shown inconsistent effects on QoL in AF, although the data is limited by inclusion of patients with paroxysmal AF, a focus on heart rate and the use of a variety of QoL tools.

Current QoL questionnaires can be divided into disease-specific evaluations or generic health assessments (such as the Short Form Health Survey SF-36[64] or the EuroQol EQ-5D[65, 66]). However there is a distinct lack of knowledge regarding the mechanisms that underpin AF-related symptoms, the responsiveness of QoL questionnaires and their validity.[60] The Atrial Fibrillation Effect on QualiTy-of-life (AFEQT) questionnaire was designed to address these disparities by using more robust methods.[67] Although there is limited clinical application to-date, AFEQT has demonstrated sensitivity to clinical change.[68] An important objective of the research is to ascertain appropriate and responsive QoL tools for this population, as well as determine the acceptability and delivery of the questionnaires to patients.

2.6 Rationale for the RATE-AF Trial

Rate-control is an integral part of management in all AF patients but hardly any controlled trial evidence exists to guide the choice of agents. We have shown that neither beta-blockers nor

For peer review only - http://bmjopen.bmj.com/site/about/guidelines.xhtml

digoxin has an impact on mortality in AF patients, even with concomitant heart failure, which highlights the need to determine treatment effects on quality of life and cardiac function.

3 Trial Design and Objectives

RATE-AF is Prospective, Randomised Open-label Blinded Endpoint (PROBE) clinical trial comparing the use of digoxin and beta-blockers as initial rate control therapy.

In this section, we discuss the trial design and study objectives. Detailed outcome measures are listed in **Section 12**.

For peer review only

Justification for a PROBE rather than a Double Blind Trial Design

Although a double blind design would be the most robust trial design with respect to bias, it would not be ethical to do so in this scenario as clinicians would feel the need to add therapy according to heart rate. In addition, the RATE-AF Trial aims to test a strategy of initial care. PROBE trial design maintains the benefits associated with a strict randomisation procedure, while the blinded end points help to eliminate bias.

The trial design aims for a pragmatic 'all-comers' approach, applicable to those seen in clinical practice to allow transfer of the findings to routine clinical management of patients with permanent AF.

Assessment and Management of Risk

This trial is categorised by the Medicines and Healthcare products Regulatory Agency (MHRA) as:

Type A = No higher than the risk of standard medical care

The assessment and management of risk is detailed in the separate **RATE-AF** Risk Assessment document. An on-going evaluation of risk will continue throughout the recruitment period.

3.1 Hypothesis

Null Hypothesis for primary outcome:

No difference in patient-reported quality of life (measured using the physical functioning domain of the SF36 questionnaire) when comparing a strategy of digoxin versus beta-blocker therapy for initial rate control in patients with permanent AF.

Alternative Hypothesis:

Use of digoxin or beta-blocker therapy as initial rate control in patients with permanent AF is superior based on patient reported quality of life (measured using the physical functioning domain of the SF36 questionnaire).

For peer review only - http://bmjopen.bmj.com/site/about/guidelines.xhtml

3.2 Primary objective

- Patient-reported quality of life (QoL), with a predefined focus on physical well-being using the SF-36 physical component summary at six months.

3.3 Secondary objectives

- Generic and AF-specific patient-reported QoL using the SF-36 global and domain-specific scores, the AFEQT overall score and the EQ-5D-5L summary index and visual analogue scale at six and twelve months.
- Echocardiographic left-ventricular ejection fraction (LVEF) and diastolic function (E/e' and composite of diastolic indices) at twelve months.
- Functional assessment, including 6-minute walking distance achieved, change in European Heart Rhythm Association (EHRA) class and cognitive function at six and twelve months.
- Change in B-type natriuretic peptide (BNP) levels as a surrogate for total cardiac strain at six months.
- Change in heart rate from baseline and group comparison using 24-hour ambulatory ECG.

3.4 Feasibility objectives

- Successful methods for recruitment
- Key issues that affect retention of participants, such as convenience, compliance and cross-over (target of 85% study completion rate).
- Drug discontinuation rate and adverse reactions leading to drug discontinuation.
- Therapy-induced requirement for additional treatment (e.g. pacemaker implantation).
- Population-specific standard deviations and proportions to enable sample size calculation for a future trial.
- Assessment of cardiovascular outcomes including a composite of adverse clinical events (mortality, thromboembolic events, myocardial infarction and cardiovascular interventions).

3.5 Exploratory objectives

- Correlation of baseline measures, including QoL questionnaires and unblinded baseline investigations such as QoL, BNP, LVEF, E/e', EHRA, intracellular methods and heart rate.
- Impact of therapy on intracellular sodium and calcium concentration and cardiotonic steroid levels as biomarkers of cellular response at six and twelve months.

For peer review only - http://bmjopen.bmj.com/site/about/guidelines.xhtml

- Impact of combination therapy on outcomes, including comparison of bisoprolol/non-dihydropyridine calcium channel blocker (CCB) vs. bisoprolol/digoxin vs. digoxin/CCB vs. single therapies.
- Change in cognitive function at twelve months
- Qualitative research of patient-reported QoL using focus groups to explore patient acceptability, optimal delivery methods and responsiveness.
- Assessment of the validity and reproducibility of echocardiographic measures in patients with AF.
- Correlation of serum digoxin concentration with change in QoL and intracellular methods.
- Cost-consequence economic analysis from an NHS perspective.

4 Selection of Participants

Participants who potentially fulfil the inclusion criteria for this trial must have their eligibility confirmed by medically qualified personnel with access to and a full understanding of the potential participant's medical history. If eligibility has been assessed and documented by medically qualified personnel, then the process of obtaining informed consent may be delegated as appropriate and as documented on the **RATE-AF** Delegation and Signature Log.

4.1 Inclusion Criteria

- Adult patients aged 60 years or older
- Permanent AF, characterised (at time of randomisation) as a physician decision for rate-control with no plans for cardioversion, anti-arrhythmic medication, or ablation therapy
- Symptoms of breathlessness (New York Heart Association Class II or more)
- Able to provide written informed consent

4.2 Exclusion Criteria

- Established clinical indication for beta-blocker therapy, e.g. myocardial infarction in the last 6 months
- Known contraindications for therapy with beta-blockers or digoxin, e.g. a history of severe bronchospasm that would preclude use of beta-blockers, or known intolerance to these medications
- Baseline heart rate <60 bpm
- History of second or third-degree heart block
- Supraventricular arrhythmias associated with accessory conducting pathways (e.g. Wolff-Parkinson-White syndrome) or a history of ventricular tachycardia or fibrillation

For peer review only - http://bmjopen.bmj.com/site/about/guidelines.xhtml

- Planned pacemaker implantation (including cardiac resynchronisation therapy), pacemaker-dependent rhythm or history of atrioventricular node ablation
- Decompensated heart failure (evidenced by need for intravenous inotropes, vasodilators or diuretics) within 14 days prior to randomisation
- A current diagnosis of obstructive hypertrophic cardiomyopathy, myocarditis or constrictive pericarditis
- Received or on waiting list for heart transplantation
- Receiving renal replacement therapy
- Major surgery, including thoracic or cardiac surgery, within 3 months of randomisation
- Severe, concomitant non-cardiovascular disease (including malignancy) that is expected to reduce life expectancy

5 Informed Consent Process

It will be the responsibility of the Investigator to obtain written informed consent for each participant prior to performing any trial related procedure. If local practice allows, this responsibility may be delegated by the Principal Investigator, to a Research Nurse as captured on the Site Signature and Delegation Log. A Participant Information Leaflet (PIL) will be provided to facilitate this process. Investigators or delegate(s) will ensure that they adequately explain the aim, trial treatment, anticipated benefits and potential hazards of taking part in the trial to the participant. They will also stress that participation is voluntary and that the participant is free to refuse to take part and may withdraw from the trial at any time. The participant will be given adequate time to read the PIL and to discuss their participation with others outside of the site research team. The participant will be given the opportunity to ask questions.

If the participant expresses an interest in participating in the trial they will be asked to sign and date the latest version of the Informed Consent Form (ICF). The participant must give explicit consent for the regulatory authorities, members of the research team and representatives of the sponsor to be given direct access to the participant's medical records.

The Investigator or delegate(s) will then sign and date the form. A copy of the ICF will be given to the participant, a copy will be filed in the medical notes, and the original placed in the Investigator Site File (ISF). Once the participant is entered into the trial, the participant's unique trial identification number will be entered on the ICF maintained in the ISF. As part of the consent process, the participant will be asked to give explicit consent to their trial-related information being sent to the Trials Office at the University of Birmingham.

This trial will include **optional consent** to allow linkage to patient data available in NHS routine clinical datasets, including primary care data (e.g. Clinical Practice Research Datalink; CPRD, The Health Improvement Network; THIN, QResearch), secondary care data (Hospital Episode Statistics; HES) and mortality data from the Office of National Statistics (ONS) through The Health and Social Care Information Centre and other central UK NHS bodies. The consent will

For peer review only - http://bmjopen.bmj.com/site/about/guidelines.xhtml

also allow access to other new central UK NHS databases that will appear in the future. This will allow us to double check the main outcomes against routine data sources, and extend the follow-up of patients in the trial and collect long-term outcome and health resource usage data without needing further contact with the trial participants. This is important as it will link a trial of treatments that may become a clinical standard of care to long-term outcomes that are routinely collected in clinical data but which may be collected during the follow-up period of the trial.

Details of the informed consent discussions will be recorded in the participant's medical notes. This will include date of discussion, the name of the trial, summary of discussion, version number of the PIL given to participant and version number of ICF signed and date consent received. Where consent is obtained on the same day that the trial related assessments are due to start, a note will be made in the medical notes as to what time the consent was obtained and what time the procedures started.

At each visit the participant's willingness to continue in the trial will be ascertained and documented in the medical notes. Throughout the trial the participant will have the opportunity to ask questions about the trial. Any new information that may be relevant to the participant's continued participation will be provided. Where new information becomes available which may affect the participants' decision to continue, participants will be given time to consider and if happy to continue will be re-consented. Re-consent will be documented in the medical notes. The participant's right to withdraw from the trial will remain.

Electronic copies of the PIL and ICF will be available from the Trials Office and will be presented on the headed paper of the local institution. Details of all participants approached about the trial will be recorded on the Participant Screening/Enrolment Log and with the participant's prior consent, their General Practitioner (GP) will also be informed that they are taking part in the trial.

6 Enrolment and Randomisation

A flowchart of the recruitment process is shown in the Trial Schema (**Figure 1**) together with the schedule of investigation. **Section 9** gives more detailed information of trial procedures and assessments.

In the majority, potentially eligible participants will be identified by their Cardiologist, usually following referral from their General Practitioner (GP), and provided with an ethically-approved patient information leaflet (PIL). The patient will then be invited to attend a baseline visit at the NIHR Wellcome Trust Clinical Research Facility (WTCRF) at Queen Elizabeth Hospital, Birmingham. Potentially eligible participants may also be identified from inpatient referrals; these patients will be provided with a PIL and invited to attend a baseline visit following the same procedure.

GP Practices in the Birmingham area may be asked to refer patients that present with AF, but are not on medication, to the RATE-AF Research Team at University Hospitals Birmingham (UHB). These patients will be given a one-page, ethics committee-approved trial summary and asked to

For peer review only - http://bmjopen.bmj.com/site/about/guidelines.xhtml

sign a contact details form to confirm that they are happy to be contacted by a member of the Research Team to arrange an appointment.

Prior to patients undertaking any trial-related procedures, informed consent will be obtained.

Details of all patients approached about the trial should be recorded on the **RATE-AF** Screening & Enrolment Log. This Log should be maintained within the Investigator Site File.

6.1 Randomisation Procedures

After all eligibility criteria have been confirmed and informed consent has been received, the participants can be randomised into the **RATE-AF** trial.

Participants will be randomised in a 1:1 ratio to either **Digoxin 62.5 – 250 µg od or Bisoprolol 1.25 – 15 mg od**. The time between randomisation and commencement of trial therapy should be minimised (ideally <24 hours). Randomisation will be provided by a computer generated programme at the Birmingham Clinical Trials Unit (BCTU), using a minimisation algorithm to ensure balance between the arms with regard to important clinical variables, stratifying for baseline EHRA (class 1/2a and 2b/3/4) and gender.

Telephone and Online Randomisation

Participants can be randomised into the trial via a secure 24 hour internet based randomisation service (https://www.trials.bham.ac.uk/RATEAF) or by a telephone call to the BCTU (telephone number **0800 953 0274**). Telephone randomisations are available Monday-Friday, 09:00-17:00. For the secure internet randomisation, each site and each randomiser will be provided with a unique log-in username and password in order to access the online system. Online randomisation is available 24 hours a day, 7 days a week, apart from short periods of scheduled maintenance and occasional network problems.

Randomisation Forms will be provided to investigators and should be completed and used to collate the necessary information prior to randomisation. Once all eligibility criteria have been provided and confirmed, a Trial Number and treatment allocation be given and relevant parties notified, including the participant's GP.

Back-up Randomisation

If the internet based randomisation service is unavailable for an extended period of time, a back-up paper randomisation will also be available at the BCTU. The randomisation list will be produced using a random length block design. In this instance, investigators should ring the BCTU randomisation service (telephone number **0800 953 0274**).

For peer review only - http://bmjopen.bmj.com/site/about/guidelines.xhtml

7 Trial Treatment

7.1 Treatment

The Investigational Medicinal Products (IMPs) for this trial are Digoxin and Bisoprolol.

At randomisation, participants will be allocated to open-label treatment with either Digoxin 62.5 – 250 µg od or Bisoprolol 1.25 – 15 mg od.

Digoxin

Digoxin is a cardiac glycoside derived from the foxglove plant. The cardiac effects of digoxin therapy are summarised by:

- Positive inotropic effects: increased intracellular calcium due to direct inhibition of sodium-potassium adenosine triphosphatase (Na/K-ATPase)
- Negative chronotropic effects: decreased conduction velocity through the atrioventricular node, an increase in the effective refractory period and an increase in vagal activity leading to sinus node depression.

Clinically, digoxin is commonly prescribed in two conditions, heart failure and AF.

Bisoprolol

Bisoprolol fumarate is a highly beta-1 selective adrenoreceptor blocker first approved by the U.S. Food and Drug Administration in 1992. The cardiac effects of bisoprolol therapy are summarised by:

- Negative chronotropic effects: a reduction in resting and exercise heart rate due to prevention of norepinephrine and epinephrine from binding to the beta-receptor in cardiac conduction tissue.
- Negative (mild) inotropic effects: an initial fall in resting and exercise cardiac output with little observed change in stroke volume and only a small increase in right atrial pressure or pulmonary capillary wedge pressure.

Clinically, bisoprolol is commonly prescribed in a range of cardiology conditions, including post-myocardial infarction, heart failure and in patients with atrial tachyarrhythmia, including AF.

7.2 Treatment Supply and Storage

Due to the participant population and the fact that the trial closely aligns with standard care, trial medication may be dispensed from routine standard stock by both the pharmacy at the research site and community pharmacies local to the participant. Both treatments are used as per normal clinical practice therefore there is no additional requirement, above that of local policy, to monitor temperature during storage.

For peer review only - http://bmjopen.bmj.com/site/about/guidelines.xhtml

Digoxin

Digoxin is available as an oral tablet in doses of 62.5, 125 and 250 µg or as an elixir (50 µg/mL). It is packaged in 28 or 500 tablet packs under the generic title digoxin and trade label Lanoxin.[69] Digoxin should be stored according to local policy.

Bisoprolol

Bisoprolol is available as an oral tablet in doses of 1.25, 2.5, 3.75, 5.0, 7.5 and 10 mg. It is packaged as 28 tablets under the generic title bisoprolol fumarate and trade labels Cardicor and Emcor.[69] Bisoprolol should be stored according to local policy.

7.3 Dosing Schedule

Digoxin

An advice sheet for the investigator is presented in **Appendix A.**

Trial maintenance doses will initially be 62.5 or 125 µg orally (at the clinician's discretion, taking into account age and renal function), with planned up-titration to 125/250 µg. The maximum trial dose will be 250 µg daily.

A single loading dose of four tablets (250 or 500 µg according to target maintenance dose) will be prescribed in digoxin-naïve participants. The clinician is permitted to omit the loading dose or prescribe a second, where necessary.

Unblinded serum digoxin concentrations will be assessed at visits 2 and 3, with results reported back to the relevant clinician(s). This process will assist in monitoring compliance, adjusting dosage in cases of low serum levels and avoiding toxicity.

Bisoprolol

An advice sheet for the investigator is presented in **Appendix B**

Trial starting doses will be 1.25 or 2.5 or 5 mg (at the clinician's discretion), with planned up-titration to 10 mg in increments of 1.25 or 2.5 mg. The maximum trial dose will be 15 mg daily. No loading dose is required.

Plasma concentrations have not shown to be associated with toxicity and are not part of standard clinical practice.

7.4 Drug Interactions and Contraindications

Digoxin

Following oral administration of digoxin, approximately 60–85% of the dose is usually absorbed, mainly from the small intestine. The onset of action is 0.5-2 hours and maximal effects occur in

For peer review only - http://bmjopen.bmj.com/site/about/guidelines.xhtml

2-6 hours. Digoxin has a large volume of distribution and approximately 20-30% of digoxin in blood is bound to plasma proteins. Metabolism is minimal but variable, with the majority of drug excreted unchanged in the urine by glomerular filtration and tubular secretion. With normal renal function, the elimination half-life is 34-44 hours which is prolonged in patients with renal failure by two to threefold. Dose adjustment is unnecessary in patients with hepatic impairment. Therapeutic plasma concentrations of digoxin have been described as 0.5-2.0 ng/mL.[70] In digoxin-naïve patients with normal renal function, approximately seven days are required to reach steady-state therapeutic concentrations if a loading dose is omitted. As such, the majority of clinicians prescribe one or two loading doses, totalling 500 to 1000 µg over 24 hours.

Caution is recommended in patients with electrolyte disturbance (due to increased risk of toxicity) and reduced doses are recommended in patients with renal impairment. There are no concerns in pregnancy or with breast-feeding, although dose adjustment may be required.

Contraindications for digoxin therapy include heart block, accessory pathway supraventricular tachycardia and a current diagnosis of obstructive hypertrophic cardiomyopathy, myocarditis or constrictive pericarditis.

Digoxin has been associated with a number of adverse effects, although data from randomised trials show little difference in comparison to placebo, apart from cases of toxicity (2% versus 0.9% respectively in the DIG trial of patients with HF)[71]. The most common side effects are gastrointestinal upset, dizziness, blurred vision, headache and rash. In toxic states (serum levels >2 ng/mL), digoxin is pro-arrhythmic and can aggravate heart failure, particularly with co-existent hypokalaemia. In cases of overdose, repeated early doses of activated charcoal may be given to reduce absorption and in severe toxicity, digoxin-specific antibody fragments are available as an intravenous infusion.

In rigorous assessment, drug interactions with digoxin have proved inconsistent.[72] Serum digoxin concentrations are increased by amiodarone, dronedarone, propafenone and quinidine but increased bioavailability with CCB and certain antibiotics (such as erythromycin and tetracycline) only occur in selected patients. The risk of toxicity increases with drugs that cause electrolyte disturbances, such as thiazide and loop diuretics.

Bisoprolol

Following oral administration of digoxin, the absolute bioavailability is approximately 80%, first pass metabolism of 20% and 30% protein binding. Peak plasma concentrations occur within 2-4 hours, the elimination half-life is 9-12 hours and steady state is attained within 5 days. Elimination occurs equally by renal and non-renal pathways with about 50% of the dose remaining unchanged in the urine.

Caution is recommended in patients with first-degree heart block, portal hypertension, diabetes, a history of obstructive airways disease, myasthenia gravis, a history of hypersensitivity and psoriasis, although many cardiologists use beta-blockers frequently in these groups with appropriate supervision. In pregnancy, beta-blockers may cause intra-uterine growth restriction,

For peer review only - http://bmjopen.bmj.com/site/about/guidelines.xhtml

neonatal hypoglycaemia, and bradycardia (although as above, these agents are frequently used in pregnancy). There is a theoretical risk of toxicity in breast feeding, although the amount present in milk is likely too small to affect infants. Abrupt withdrawal should be avoided, especially in cases of ischaemic heart disease. Up-titration should be more cautious in patients with renal or hepatic impairment.

Contraindications for bisoprolol therapy include cardiogenic shock, overt cardiac failure, second or third degree heart block, marked sinus bradycardia and severe peripheral arterial disease.

Bisoprolol has been associated with a wide variety of adverse effects although data from RCTs suggest similar discontinuation rates compare to placebo.[5, 73] The most common adverse symptoms are lethargy, headache, peripheral oedema, upper respiratory tract symptoms, gastrointestinal upset and dizziness. In cases of overdose, bradycardia, hypotension, congestive heart failure, bronchospasm and hypoglycaemia may be expected, with treatment directed to supportive methods and atropine, fluids, glucagon or diuretics as required.

Pharmacokinetic interactions with beta-blockers have not shown to be clinically significant. Drugs that reduce absorption include aluminium salts and cholestyramine, whilst metabolism can be increased by barbiturates and rifampicin and decreased with cimetidine, erythromycin, fluvoxamine, and hydralazine.

7.5 Accountability Procedures and Labelling

Through the risk-adapted approach, a full risk assessment of the **RATE-AF** trial has been conducted including the drug accountability requirements. The IMPs will be used within their authorisations, prescribed on an NHS prescription and dispensed by pharmacy from standard stock. The risk assessment has determined that a normal dispensing label is appropriate and an additional clinical trial label is not necessary (as covered by Regulation 46 (2) of SI 2004/1031). Drug accountability will be according to standard practice for NHS prescriptions. Details of how compliance will be assessed can be found in **Section 7.7**.

7.6 Treatment Modification

Patients that withdraw from medication for any reason will do so under strict clinical supervision.

The trial is designed to assess the impact of **initial** impact of rate control therapy; it is expected that treatments will modify during the trial period (in particular, the addition of therapy to attain heart rate targets). Patients will not be withdrawn from the trial if they commence therapy from the other arm of the trial due to any absolute or relative clinical indications (for example, patients in the digoxin arm starting beta-blockers due to incident myocardial infarction, or heart failure with reduced LVEF).

For peer review only - http://bmjopen.bmj.com/site/about/guidelines.xhtml

7.7 Assessment of Compliance

We will ask participants about compliance with their trial medication at each follow-up visit and this will be documented in the CRFs. It may also be clinically evident from the heart rate check, performed as part of all visits, whether or not the patient has been compliant with their trial mediation.

In addition, patients that are randomised to the digoxin arm will have a serum digoxin sample taken as part of Visit 2 (month 6) and Visit 3 (month 12) follow-ups. The results will indicate whether the patient has been compliant with their trial medication.

8 Trial Procedures and Schedule of Assessments

8.1 Baseline Visit

The baseline visit will occur as soon as possible after screening and will involve the following procedures (see **Section 9** for procedure details):

- Verify inclusion/exclusion criteria.
- Obtain written informed consent from the potential participant.
- Randomise the patient via telephone or the secure web-based system as outlined in **Section 6**
- Administer QoL and functional capacity questionnaires.
- Review recent blood results (within 6 months of Baseline Visit)
    - Assessing renal function to aid in dose assignment and serum potassium level as part of standard clinical care.
- Document the use of oral anticoagulation and arrange appropriate prescription for patients not on therapy according to clinical guidelines. If the participant is already receiving vitamin-K antagonists (VKA), recent INR results will be documented.
- Record results of physical examinations.
- Collect blood samples for baseline blood tests and biomarker analysis.
- Complete case report form (CRF)
- Perform a 12-lead electrocardiogram.
- Supervise a 6-minute walk test, recording distance walked and peak heart rate.
- Arrange the baseline echocardiogram; images will be delivered to the echocardiographic core laboratory for blinded reporting.
- Discuss the randomised allocation with the participant including schedule for drug therapy and up-titration.

For peer review only - http://bmjopen.bmj.com/site/about/guidelines.xhtml

Participants will be followed up by telephone call two weeks after the Baseline Visit to ensure they have commenced trial medication.

8.2 Up-Titration Visits

For the majority of participants, two up-titration visits will be planned to supervise the appropriate use of medications as per the up-titration schedule (see **Appendices A and B**). Additional up-titration visits, as required, are acceptable in order to attain a heart rate at rest of ≤100bpm.

Up-titration visits will involve the following procedures:

- Record adverse events as reported by the participant or observed by the investigator.
- Review of medications and plan for trial drug up-titration
- Assessment of compliance
- Symptom-directed clinical examination
- Vital signs, including heart rate and blood pressure
- Administer QoL and functional capacity questionnaires (last uptitration visit only).
- Organise a 24-hour ambulatory ECG once up-titration completed (results to be forwarded to the clinician).

8.3 Visit 2, Month 6

Visit 2 will occur at an interval of six months (± four weeks) after the Baseline Visit and involve the following procedures:

- Administer QoL and functional capacity questionnaires.
- Record adverse events as reported by the participant or observed by the investigator.
- Confirm current rate control therapy (including dosage) and check concomitant medications.
- Assessment of compliance.
- Collect blood samples for biomarker analysis.
- Collect blood sample for serum digoxin concentration, potassium and creatinine as part of standard clinical care.
- Record time in therapeutic range for patients on anticoagulation with vitamin-K antagonists and compliance in patients receiving non-VKA oral anticoagulants.
- Obtain a twelve lead ECG.
- Record the results of physical examinations and vital signs.
- Supervise a 6-minute walk test, recording distance walked and peak heart rate.
- Complete other CRF requirements.
- If an echocardiogram has been performed for clinical reasons since the previous visit, images will be retrieved and sent to the core echocardiographic laboratory.

For peer review only - http://bmjopen.bmj.com/site/about/guidelines.xhtml

- Confirm appointment date for Visit 3.

8.4 Visit 3, Month 12 (Final Trial Assessment)

Visit 3 will occur at an interval of 12 months (± four weeks) after the Baseline Visit and involve the following procedures:

- Administer QoL and functional capacity questionnaires.
- Record adverse events as reported by the participant or observed by the investigator.
- Confirm current rate control therapy (including dosage) and check concomitant medications.
- Assessment of compliance.
- Transthoracic echocardiography (as per **Section 9.6**), with images delivered to the echocardiographic core laboratory for blinded reporting.
- Collect blood sample for serum digoxin concentration, potassium and creatinine as part of standard clinical care.
- Record time in therapeutic range for patients on anticoagulation with vitamin-K antagonists and compliance in patients receiving non-VKA oral anticoagulants.
- Obtain a twelve lead ECG.
- Record the results of physical examinations and vital signs.
- Supervise a 6-minute walk test, recording distance walked and peak heart rate.
- Complete other CRF requirements.
- If an echocardiogram has been performed for clinical reasons since the previous visit, images will be retrieved and sent to the core echocardiographic laboratory.
- Complete the end of trial standardised letter to the GP and clinician explaining that the participant has reached the end of the trial protocol and is no longer bound by their allocated medication strategy. Advise that all participants are invited for continued follow up and long term clinical outcome assessment.
- Provide final instructions to participant (e.g. follow-up of ongoing adverse events).

8.5 Investigator-blinded Endpoints

Investigator-blinded endpoints (PROMs, echocardiography and biomarkers) will be assessed by the core laboratory, identified only by the trial number. Ambulatory ECG and serum digoxin level will remain unblinded and results delivered to the responsible clinician.

8.6 Long Term Follow-Up

In patients who have agreed to NHS data linkage, a follow-up CRF will be completed. The CRF will capture items that include, but are not limited to death, hospital admissions and

For peer review only - http://bmjopen.bmj.com/site/about/guidelines.xhtml

cardiovascular events. The planned interval for outcome assessment is 2 and 5 years post-enrolment.

8.7 Withdrawal

Participants may withdraw at any time during the main **RATE-AF** trial if they choose not to continue or if their clinical team feel that continued participation in the trial is inappropriate.

An investigator may deem it necessary to withdraw a participant from the trial if:

1) Any clinical adverse event, laboratory abnormality, or other medical condition or situation occurs such that continued participation in the trial would not be in the best interest of the participant.
2) The participant meets an exclusion criterion (either newly developed or not previously recognised) that precludes further trial participation.

Full details of the reason(s) for withdrawal should be recorded on the Case Report Forms (CRFs) if healthcare professional-initiated, otherwise a simple statement reflecting patient preference will suffice.

Clear distinction will be made between withdrawals from trial treatments whilst allowing further follow-up, and any participants who refuse any follow-up. If a participant explicitly withdraws consent to any further data recording, then this decision will be respected. All communications surrounding the withdrawal will be noted in the participant's records and no further data will be collected for the participant.

In the case of missing echocardiographic outcome data due to withdrawal (but with consent for ongoing follow-up) or death, results of recent clinical echocardiography will be retrieved. The participant's permission to obtain such data will be obtained and documented during the consent process. As with all trial echocardiograms, the scan will be reported by the core echocardiographic laboratory. With respect to patient-reported outcomes, QoL questionnaires will be mailed to participants who withdraw from trial treatment but consent to ongoing follow up. Those patients where adverse symptoms were related to withdrawal will be invited to a focus group for further discussion.

8.8 Trial Duration

Patients will be on trial medication for 12 months and will be followed-up, during this period according to the protocol. At the end of the 12 months, the participants may, as determined by their clinician, continue on medication but it will not be considered part of the trial intervention. The trial will cease when the 12-month follow-up has been completed for the last participant recruited.

For peer review only - http://bmjopen.bmj.com/site/about/guidelines.xhtml

Table 1: Schedule of Assessments

Procedures		Baseline Visit	Up-titration Visits (Day 14 to 60)	Visit 2, Month 6 (± 4 weeks)	Visit 3, Month 12 (± 4 weeks)
Assessment of eligibility criteria		X			
Informed consent taken		X			
Review of medical history		X			
Review of medications		X	X	X	X
Physical exam	Complete	X			
	Symptom-directed		X	X	X
	Vital signs	X	X	X	X
Quality of life assessment		X	(X)	X	X
Functional and cognitive assessment		X		X	X
Transthoracic echocardiogram		X			X
12-lead electrocardiogram		X		X	X
6-minute walk test		X		X	X
24-hour ambulatory ECG			X	(X)	
Clinical labs	Chemistry	X		X	X
	Haematology	X		X	X
	Serum digoxin			(X)	(X)
Trial labs	BNP	X		X	
	Stored sample	X		X	
Assessment of compliance			X	X	X
Assessment of adverse events			X	X	X

Parentheses denote where a procedure is dependent on the stage of participants within the trial.

For peer review only - http://bmjopen.bmj.com/site/about/guidelines.xhtml

9 Trial Procedures

9.1 Procedures Defined as Standard Clinical Care

The following assessments are considered part of the standard clinical care of AF patients receiving heart rate control therapy and will occur at all trial visits:

- Blood tests for haemoglobin, serum creatinine, potassium and serum digoxin concentration; these will be obtained by the research nurse and submitted to the site-specific hospital laboratory as per local guidelines and SOPs, ensuring that all specimens are accurately labelled and handled appropriately. In the case of results requiring urgent action, local policies will be followed which may include the participant visiting their GP, local hospital or investigator. In all cases, appropriate trial documentation will be completed.
- A 12-lead ECG; these will be completed by appropriately trained local staff.

9.2 Medical History

Medical history will be obtained by interview and from medical records (physical and electronic) at the Baseline Visit comprising:

- Cardiovascular history, including prior ischaemic coronary disease, interventions and surgery, history of hypertension, heart failure or hyperlipidaemia, stroke or transient ischaemic attack, pulmonary embolus/deep vein thrombosis and peripheral vascular disease.
- AF history, including year of diagnosis, previous cardioversions, previous ablation therapy and anti-arrhythmic drug history.
- Pacemaker history, including date and reason for implantation, type of device (single-chamber, dual-chamber, biventricular, implanted defibrillator) and dependency.
- Non-cardiac history, including diabetes mellitus, airways disease (asthma/COPD), renal impairment, bleeding history and other major co-morbidities.
- Social and demographic history, including smoking status (current/ex/never), race (Caucasian/Indian subcontinent/Asian/African/other) and physical activity using the International Physical Activity Questionnaire (short form).

9.3 Medication History

Medications history will be assessed according to the categories below and include current dosage. Except for anticoagulation, antiarrhythmic and rate control therapies, only current medications will be included.

- Anticoagulation therapy (vitamin-K antagonists and novel agents), including past use, INR results and time in therapeutic range.
- Antiarrhythmic therapy, including past use.

For peer review only - http://bmjopen.bmj.com/site/about/guidelines.xhtml

- Rate control therapy (beta-blockers, digoxin, CCB), including past use.
- Antiplatelet therapy.
- Angiotensin-converting enzyme inhibitors or angiotensin receptor blockers.
- Aldosterone antagonists.
- Diuretics (loop, thiazide, potassium-sparing, others).
- Nitrates.
- Other anti-hypertensive/anti-anginal therapy.
- Statins.
- Other lipid-lowering medication.
- Diabetic medication and insulin.
- Asthma/COPD medication (including inhalers).
- Non-steroidal anti-inflammatory agents.

9.4 Physical Examination

Physical and vital signs will be assessed at all up-titration and trial visits. In most cases, a targeted physical examination will be performed, comprising of cardiovascular elements as summarised below:

- Heart rate (manual palpation at radial artery and apex).
- Heart sounds.
- Lung auscultation.
- Assessment of jugular venous pressure and/or peripheral oedema.
- Other focused examinations according to symptoms and complaints.
- Blood pressure (two measurements at the right brachial in a seated position preferred, using a validated oscillometric device).
- Height (Baseline Visit only), weight (all listed visits) and waist circumference (Baseline Visit; defined as the narrowest point between ribs and hips when viewed from the front after exhaling to the nearest centimetre).

9.5 Patient Reported Outcomes

9.5.1 Choice of Outcomes and Qualitative Research

A systematic review (according to and in collaboration with the COnsensus-based Standards for the selection of health Measurement Instruments, COSMIN[74]) is underway to evaluate PROMs in AF, with a focus on psychometric properties including internal consistency, reliability, and measurement error. Additional assessment and practical evaluation of PROMs will follow published guidance[75, 76], complementing qualitative research using patient focus groups, surveys

For peer review only - http://bmjopen.bmj.com/site/about/guidelines.xhtml

and directed interviews guided by the PROMs and qualitative research centres at the University of Birmingham.[77]

Instruments for assessment will be selected on the basis of overall validity, preferably in this patient population but including other groups where data are limited. Patient focus groups will allow exploration of patient perspectives on appropriate instruments that adequately reflect the experience of living with AF.[78] They will also allow comparison of QoL questionnaires that adequately summarise patient-prioritised components of their health and well-being. Additional focus groups and individual interviews will occur at interim and final follow-up during the trial. These aim to understand the patient experience of trial participation and processes, including the ease of completion of QoL questionnaires, relevance, reasons for non-completion and other feasibility issues that emerge during the trial e.g. non-compliance and recruitment, with reference to core outcome sets for this population.[79] A patient and public involvement (PPI) panel will contribute to all stages in the focus group process.[80]

This protocol was developed in accordance with the Standard Protocol Items for Randomized Trials [SPIRIT] statement[81], and the latest PROM-specific guidance from the International Society for Quality of Life Research (ISOQOL) Best Practice taskforce.[77, 82, 83]

9.5.2 Data Collection for PROMs

PROMs will be assessed at all main visits (Baseline, 2 and 3) and at the participants final up-titration visit (if applicable). The QoL tools used will be EQ-5D-5L, SF-36 and AFEQT. To avoid introducing co-intervention bias, all QoL data will be kept confidential and will not be used to inform clinical care.[84] Patients will be advised of this in the patient information sheet. PROMs will be collected at the start of each visit, before other trial procedures. In cases where the visit coincides with a clinician review, questionnaires should be completed in advance. The feasibility of using an online data collection tool will be explored, administered by trained research nurses and according to good-practice guidelines.[85] We will use this trial to perform an initial small-group assessment of electronic PROMs-equivalence to inform a future clinical event trial.

Qualitative research will be performed using a focus group of 10 volunteer patients enrolled at the start of the trial (5 in each randomised group). The focus group will meet after up-titration and then at 6 and 12 months. Detailed methods will be established before the first meeting, in collaboration with the University of Birmingham Qualitative Research Group.

All staff will receive training in QoL collection, with specific guidance on reducing introduced bias, minimising missing data and coaching participants to use the QoL software. Levels of missing PROMs data will be monitored. The site personnel responsible for collection of patient reported outcomes will be the Research Nurse under the supervision of the Principal Investigator.

For peer review only - http://bmjopen.bmj.com/site/about/guidelines.xhtml

9.5.3 Outcome Appraisal

Each QoL tool will be scored according to their published requirements (www.euroqol.org; www.sf-36.org; www.afeqt.org), using total and sub-category scores where appropriate.

To avoid dilution of effect over time, the primary analysis will be at six months (adjusting for baseline QOL and stratification variables). We have predefined a focus on physical well-being, which we hypothesize are where the greatest treatment effects will be observed, but will explore all aspects of QoL. Exploratory analysis of medication effects over the 12-month period will also be analysed and remain clinically important, as little data currently exists on the longer-term profile of QoL in AF.

Qualitative research outcomes will focus on the clinical responsiveness of the QoL instruments. The findings of the COSMIN systematic report will determine these outcomes and their relevant appraisal.

The RATE-AF trial will allow us to gain an initial understanding and framework of the patient experience of AF. We aim to begin the process of determining appropriate and responsive PROMs for AF patients and the optimum methods for delivery into a subsequent large-scale clinical trial.

9.6 Transthoracic Echocardiography

Echocardiography will be performed at Visits 1 and 3 and focus on systolic left-ventricular (LV) function, diastolic function and left-atrial assessment. Images will be obtained by an accredited echocardiographer. All trial echocardiograms will be labelled with the Trial Number and pseudoanonymised patient data, with specific instruction that the echocardiographer will remain blinded to the treatment assignment. All images will be archived to the core echocardiographic laboratory, with a copy retained in the site file.

9.6.1 Reproducibility and Validity of Measurements

Inter-observer and intra-observer variability in measurement will be assessed by comparing results of the stated methods discussed below across the cardiac cycle. To evaluate the minimum number of repeat measurements required that maintains clinical utility, reproducibility of single measurements will be compared to averages of 3/5/10 beats. This will also include the reliability of using an 'index beat' with a cycle length equivalent to a heart rate of 70-80 beats per minute, or with similar preceding and pre-preceding RR intervals.

9.6.2 Systolic LV Function

Systolic LV function will be determined by the following methods:

- Two-dimensional biplane Simpson's method utilising the simultaneous multi-planar approach to obtain LVEF in a single heartbeat (four and two-chamber views). In each

For peer review only - http://bmjopen.bmj.com/site/about/guidelines.xhtml

view, LV end-diastolic and end-systolic volumes (LVEDV, LVESV) are computed, with LVEF calculated as (LVEDV – LVESV) / LVEDV. Two-dimensional echocardiography has excellent spatial resolution but is limited by potential foreshortening of the ventricular apex and drop-out of the endocardial border.

- Standard Simpson's biplane method with four and two-chamber volumes obtained from separate heartbeats. This is the conventional method in current clinical use but is limited by varying RR intervals in AF.
- Fractional shortening on M-mode along the minor-axis of the left-ventricle (parasternal long-axis), calculated by the formula: (LV internal dimension in diastole - LV internal dimension in systole) / LV internal dimension in diastole. M-mode measurements are reproducible and easy to perform with excellent temporal resolution, but are limited in cases of regional wall motion abnormalities and in patients where the true minor-axis is difficult to visualise.
- Both automated endocardial tracking and speckle-tracking analysis will be utilised (where available) by the echocardiographic core laboratory. Multiple planes will be obtained (four-chamber, two-chamber and short-axis mid-ventricle views). These methods have the advantage of reducing operator time and are angle-independent, but rely on good ultrasound windowing. Global longitudinal systolic strain using 2D speckle-tracking has recently been proposed as an important marker for adverse cardiovascular outcomes in AF.[86]
- Three-dimensional full-volume analyses of LV function, with single-beat analysis where feasible. This method has the advantage of not relying on geometric assumptions and allows the acquisition of full volume data within a single heartbeat. It correlates well with gold standard methods such as cardiac magnetic resonance imaging, but relies on adequate ultrasound windowing.
- Peak S-wave on tissue Doppler imaging (TDI) of the mitral valve annular sub-endocardium. This method has good correlation with LVEF across a wide range of function and is obtainable in patients with poor acoustic windows, but is limited in cases of regional wall motion abnormality.

Where poor quality acoustic windows limit accurate assessment of LV function, use of an intravenous contrast agent is recommended in participants without known allergy.

9.6.3 Diastolic LV Function

Diastolic LV function will be determined using the following methods (in all cases repeated over 3-5 cardiac cycles):

- Mitral inflow pulse-wave Doppler peak E velocity and deceleration time (DT).
- Mitral annular TDI to calculate septal E', lateral E' and the individual and averaged E/E' ratios.
- LV outflow tract pulse-wave Doppler to calculate isovolumic relaxation time (IVRT).

For peer review only - http://bmjopen.bmj.com/site/about/guidelines.xhtml

- Pulmonary vein pulse-wave Doppler to calculate peak systolic (where present) and diastolic velocities, ratio of peak velocities and DT of diastolic PV flow.
- Colour M-mode Doppler assessment of mitral flow propagation velocity (Vp) and ratio of E/Vp.

Overall diastolic function will be categorised according to the British Society of Echocardiography guidelines into normal function or mild/moderate/severe dysfunction based on a combination of the above variables. Individual parameters will also be categorised using cut-points identified from published studies.[87]

9.6.4 Left Atrial Size and Function

Left atrial (LA) size will be measured in the anteroposterior (parasternal long-axis), transverse and longitudinal dimensions (apical 4-chamber). LA volumes will be calculated using the biplane area-length method: (0.85 x 4-chamber LA area x 2-chamber LA area) / LA length. The length is measured from the middle of the plane of the mitral annulus to the superior aspect of the LA (shortest of 4- and 2-chamber measurements). LA volumes will be indexed for body surface area.

Where suitable datasets are obtained, 3D LA volumetric analysis and assessment of LA function and strain will also be performed.

9.6.5 Additional Echocardiography Parameters

The following parameters will be obtained in all participants:

- Tricuspid annular plane systolic excursion (TAPSE) for estimation of right ventricular function using pulse-wave Doppler.
- Where applicable, mitral regurgitation dP/dt.

9.7 Laboratory Evaluations

The use of biomarkers than can affect treatment decisions in AF is at an early stage of development.[88] The RATE-AF trial will allow us to collect and store blood samples on patients at baseline and follow-up, providing a unique biobank of AF patients receiving rate-control. In collaboration with the Human Biomaterials Resource Centre (HBRC) at the University of Birmingham, we will also isolate DNA for future work on predictors of response, including known polymorphisms of rate-responsiveness.[89]

Laboratories at each clinical site will process the standard laboratory investigations required as part of standard clinical care (see **Section 9.1**). Trial laboratory evaluations will be performed at the core laboratory and processed according to the guidelines in **Sections 9.7.1**, **9.7.2** and **9.7.3**.

For peer review only - http://bmjopen.bmj.com/site/about/guidelines.xhtml

9.7.1 Laboratory Assays

NT-pro B-type natriuretic peptide will be analysed using a Sandwich immunoassay using monoclonal ruthenium labelled antibody and Roche Cobas 8000 e602. The total coefficient of variation for repeatability with this assay is <2% with an estimated volume of 250 microlitres required for each test and measurement range of 5‑35000 pg/mL (0.6‑4130 pmol/L).

9.7.2 Cellular Response to Rate Control

The effect of baseline and follow-up serum on intracellular sodium/calcium, force of contraction and activation of ERK1/2-dependent cascades will be examined in human induced pluripotent stem cell-derived cardiomyocytes, using well-established integrated fluorescence/contractility photometry and western blotting techniques.[90, 91] DigiFAB[92], will be used to determine whether changes are dependent on endogenous cardiotonic steroids, which can modulate intracellular ion concentration in cardiomyocytes[93, 94], and potentially contribute to treatment discontinuation (or the development of toxicity).[95] The concentration of serum cardiotonic steroids will be determined using liquid chromatography–tandem mass spectrometry. Individual change in cardiotonic steroids and intracellular sodium/calcium will be correlated with the change in heart rate, LVEF, B-type natriuretic peptide and quality of life. In addition, we will identify patterns in patients withdrawing from treatment or experiencing adverse reactions.

9.7.3 Stored Blood Samples

Blood samples will be stored at HBRC for future biomarker and genetic analysis, with participants providing explicit consent for this process. Any future use of these samples will be undertaken with ethical approval.

9.7.4 Specimen Preparation, Handling, Storage and Shipment

Specimens will be handled according to local standard operating procedures consisting of the time requirements for processing, required temperatures, aliquots of specimens, where they will be stored, how they will be labelled, the process for remnant samples/disposal and appropriate instructions for transportation.

9.8 Economic Evaluation

The RATE-AF trial will allow determination of the most appropriate data collection methods and ease of acquiring resource use and cost data for a subsequent outcomes trial. Specifically, how data is obtained from secondary care records, patient-reported resource use and the feasibility of obtaining primary care records. A preliminary economic evaluation from an NHS perspective will be performed to estimate costs over the 12-month period. The patient-level cost-analysis will determine all AF-related costs, with respect to trial interventions and secondary-care resource use (including adverse events) in the two arms of the trial. We will collect both cost and outcome data and present them in a cost-consequence analysis. Costing for this trial suggests that simplifying medication alone could result in a saving of £5900 over each 12-month period.

For peer review only - http://bmjopen.bmj.com/site/about/guidelines.xhtml

Considering the high and increasing prevalence of AF, this could result in a substantial NHS cost savings, particularly if adverse reactions are also reduced. The aim of this objective within the trial is to prepare the groundwork for a future cost-per-quality adjusted life year (QALY) analysis of rate-control in AF.

Costs of care will be derived from patient level resource-use data, focusing on secondary care costs, and including adverse effects, such as pacemaker implantation. Other major drivers of cost are hospitalisation (including visits to Accident & Emergency), unplanned outpatient visits and outpatient tests such as echocardiography or ambulatory ECG. The cost analysis will also consider therapy costs, both trial drug and additional treatments. Unit costs will be obtained from standard sources including NHS Reference Costs, Unit Costs of Health and Social Care[96] and health care providers. Total per-patient health care costs will initially be calculated thus allowing the estimation of mean costs per trial arm over 12 months follow-up. Responses to the EQ-5D-5L questionnaire at baseline, visit 2 (6 months) and visit 3 (12 months) will be used to plan a future QALY analysis.

Key feasibility elements are:

- Determining the best methods for obtaining hospitalisation data, including where participants have been hospitalised outside of research site
- Whether robust primary care costs can be estimated and the method(s) for acquiring this type of data
- How key cost drivers can be incorporated into data collection for any future trial

For peer review only

For peer review only - http://bmjopen.bmj.com/site/about/guidelines.xhtml

10 Pharmacovigilance

Definitions of different types of AE are listed in **Table 2**. The Investigator should assess the seriousness and causality (relatedness) of AEs experienced by the participant (this should be documented in the source data). For further information please refer to **Section 10.1**.

Table 2: Standard AE Definitions

Term	Definition
Adverse Event (AE)	Any untoward medical occurrence in a patient or clinical trial participant administered a medicinal product and which does not necessarily have a causal relationship with this treatment
Adverse Reaction (AR)	Any untoward and unintended response in a participant to an investigational medicinal product which is related to any dose administered to that participant
Serious adverse event (SAE), serious adverse reaction (SAR) or unexpected serious adverse reaction	Any adverse event, adverse reaction or unexpected adverse reaction, respectively, that:<br>• results in death;<br>• is life-threatening;<br>• requires hospitalisation or prolongation of existing hospitalisation;<br>• results in persistent or significant disability or incapacity; or<br>• consists of a congenital anomaly or birth defect
Unexpected Adverse Reaction	An adverse reaction the nature and severity of which is not consistent with the information about the medicinal product in question set out:<br>(a) in the case of a product with a marketing authorisation, in the summary of product characteristics for that product;<br>(b) in the case of any other investigational medicinal product, in the investigator's brochure relating to the trial in question.
SUSAR	Suspected Unexpected Serious Adverse Reaction

10.1 Recording and Assessment of Adverse Events

All adverse events will be reportable to the **RATE-AF** Trial Office up to 30 days post last IMP administration. Any SUSAR related to the IMP should to be reported irrespective of how long after IMP administration the reaction has occurred.

Adverse events will be recorded in the medical records and CRFs. Most AE/ARs that occur in this trial, whether they are serious or not, will be 'expected' treatment-related toxicities due to the drugs used in this trial.

For peer review only - http://bmjopen.bmj.com/site/about/guidelines.xhtml

Refer to **Table 3** for definition of expectedness.

Table 3: Expectedness

Category	Definition
**Expected**	An adverse event that is classed in nature as serious and which is consistent with the information about the IMP listed in the Investigator Brochure (or SmPC if Licensed IMP) or clearly defined in this protocol
**Unexpected**	An adverse event that is classed in nature as serious and which is not consistent with the information about the IMP listed in the Investigator Brochure (or SmPC if Licensed IMP)

Adverse events will be recorded with clinical symptoms and accompanied with a simple, brief description of the event, including dates as appropriate.

The assessment of relationship of adverse events to the administration of IMP is a clinical decision based on all available information at the time. The following categories as outlined in **Table 4** will be used to define the causality of the adverse event.

Table 4: Categorisation of Causality

Category	Definition
**Definitely**	There is clear evidence to suggest a causal relationship, and other possible contributing factors can be ruled out
**Probably**	There is evidence to suggest a causal relationship, and the influence of other factors is unlikely
**Possibly**	There is some evidence to suggest a causal relationship (e.g. the event occurred within a reasonable time after administration of the trial medication). However, the influence of other factors may have contributed to the event (e.g. the patient's clinical condition, other concomitant events)
**Unlikely**	There is little evidence to suggest there is a causal relationship (e.g. the event did not occur within a reasonable time after administration of the trial medication). There is another reasonable explanation for the event (e.g. the patient's clinical condition, other concomitant treatments)
**Not related**	There is no evidence of any causal relationship

The relevant SmPC for Digoxin and Bisoprolol (which will be dependent on which generic is being used according to local practice at each site) should be filed in the site file by the local research team.

For peer review only - http://bmjopen.bmj.com/site/about/guidelines.xhtml

The **RATE-AF** Trial protocol and the reference safety information will be used to assess disease related and/or expected events related to the trial treatment.

10.2 Non-Serious Adverse Events/ Adverse Reactions

Refer to Table 2 for definitions

Common adverse reactions (see Section 7.4) will be recorded on the relevant CRF and sent to the RATE-AF Trial Office.

10.3 Serious Adverse Events

Refer to Table 2 for definitions

All Serious Adverse Events (SAEs), that are not excluded from expedited reporting will be recorded in the hospital notes and should be reported to the **RATE-AF** Trial Office on a SAE Form. The completed form should be **faxed to the RATE-AF Trial Office on 0121 415 9135 or 0121 415 9136**, as soon as possible and ideally within one working day of becoming aware of the event. The site Investigator should be able to respond to any related queries raised by the **RATE-AF** Trial Office as soon as possible.

10.3.1 Expected SAEs NOT to be Reported on a SAE Form

Expected SAEs are those listed in the current SmPC for the trial IMPs and can be excluded from the expedited reporting outlined in **Section 10.1**, for example if they are expected to occur on a regular basis and offer no further new information to the safety profile. These events should continue to be recorded in the source data and relevant CRFs.

In addition, events **NOT** considered to be SAEs are hospitalisations for:

- Routine treatment or monitoring of the studied indication, not associated with any deterioration in condition
- Treatment, which was elective or pre-planned, for a pre-existing condition that is unrelated to the indication under trial, and has not worsened

Note: Death from any cause should be reported on an SAE Form and returned to the **RATE-AF** Trial Office.

10.4 SUSARs

Refer to Table 2 for definitions

SAEs classed by as both suspected to be related to the trial IMP and unexpected are categorised as SUSARs, and are always subject to expedited reporting. An SAE Form should be completed,

For peer review only - http://bmjopen.bmj.com/site/about/guidelines.xhtml

and faxed to the RATE-AF Trial Office within 24 hours of the research staff at site becoming aware of the event. The local investigator will provide the causality assessment.

The Chief Investigator (or nominated individual) will undertake urgent review of all such SAEs and may request further information immediately from the clinical team at site. The Chief Investigator will not overrule the causality or seriousness assessment given by the local investigator but may add additional comment on these. The Chief Investigator will provide the determination of expectedness according to the reference safety information.

SUSARs will be notified to the MHRA and REC by the RATE-AF Trial Office. SUSARs that are fatal or life-threatening will be notified to the MHRA and REC within 7 days after the **RATE-AF** Trial Office has been notified. Other SUSARs will be reported to the REC and MHRA within 15 days after the Trial Office has been notified.

10.5 Development Safety Update Reports

The **RATE-AF** Trial Office will provide the MHRA with Development Safety Update Reports (DSURs). The reports will be submitted within 60 days of the Developmental International Birth Date (DIBD) of the trial each year until the trial is declared ended.

10.6 Annual Progress Reports

An annual progress report will be submitted to the REC within 30 days of the anniversary date on which the favourable opinion was given, and annually until the trial is declared ended.

10.7 Pregnancy

Due to the age of participants that will be randomised into the **RATE-AF** Trial (≥ 60 years), it is highly improbable that female participants will be pregnant at the time of randomisation, or become pregnant during the trial. Any pregnancies will be followed up for outcome; any outcome meeting the definition of an SAE will be reported to the **RATE-AF** Trial Office on the relevant CRF.

10.8 Reporting Urgent Safety Measures

If any urgent safety measures are taken the Principal Investigator/BCTU/Sponsor shall immediately and in any event no later than 3 days from the date the measures are taken, give written notice to the MHRA and the REC of the measures taken and the circumstances giving rise to those measures.

For peer review only - http://bmjopen.bmj.com/site/about/guidelines.xhtml

11 Quality Control and Quality Assurance

11.1 Site Set-Up and Initiation

All participating Principal Investigators will be asked to sign the necessary agreements and supply a current CV to the Trials Office. All members of the site research team will also be required to sign a site signature and delegation log. Prior to commencing recruitment all sites will undergo a process of initiation and will have completed GCP training. Key members of the site research team will be required to attend either a meeting or a teleconference covering aspects of the trial design, protocol procedures, Adverse Event reporting, collection and reporting of data and record keeping. Sites will be provided with an Investigator Site File containing essential documentation, instructions, and other documentation required for the conduct of the trial. The Trials Office must be informed immediately of any change in the site research team.

11.2 Central Monitoring

Monitoring of this trial will be to ensure compliance with Good Clinical Practice. A risk proportionate approach to the initiation, management and monitoring of the trial will be adopted (as per the MRC/DH/MHRA Joint Project: Risk-adapted Approaches to the Management of Clinical Trials of Investigational Medicinal Products) and outlined in the trial-specific risk assessment.

The Trials Office will be in regular contact with the site research team to check on progress and address any queries that they may have. The Trials Office will check incoming CRFs for compliance with the protocol, data consistency, missing data and timing. Sites will be asked for missing data or clarification of inconsistencies or discrepancies. Sites will be requested to send in copies of signed Informed Consent Forms and other documentation for in-house review for all participants providing explicit consent.

Additional on-site monitoring visits may be triggered, for example by poor CRF return, poor data quality, low SAE reporting rates, excessive number of participant withdrawals or deviations. This will be detailed in the monitoring plan. If a monitoring visit is required the Trials Office will contact the site to arrange a date for the proposed visit and will provide the site with written confirmation. Investigators will allow the **RATE-AF** trial staff access to source documents as requested.

11.3 Audit and Inspection

The Principal Investigator will permit trial-related monitoring, quality checks, audits, ethical reviews, and regulatory inspection(s) at their site, providing direct access to source data/documents. The Principal Investigator will comply with these visits and any required follow up. Sites are also requested to notify the Trials Office of any MHRA inspections.

For peer review only - http://bmjopen.bmj.com/site/about/guidelines.xhtml

11.4 Notification of Serious Breaches

In accordance with Regulation 29A of the Medicines for Human Use (Clinical Trials) Regulations 2004 and its amendments the Sponsor of the trial is responsible for notifying the licensing authority in writing of any serious breach of the conditions and principles of GCP in connection with that trial or the protocol relating to that trial, within 7 days of becoming aware of that breach.

For the purposes of this regulation, a “serious breach” is a breach which is likely to effect to a significant degree the safety or physical or mental integrity of the participants of the trial; or the scientific value of the trial. Sites are therefore requested to notify the Trials office of any suspected trial-related serious breach of GCP and/or the trial protocol. Where the Trials Office is investigating whether or not a serious breach has occurred sites are also requested to cooperate with the Trials Office in providing sufficient information to report the breach to the MHRA where required and in undertaking any corrective and/or preventive action. Sites may be suspended from further recruitment in the event of serious and persistent non-compliance with the protocol and/or GCP, and/or poor recruitment. Any major problems identified during monitoring may be reported to Trial Management Group and Trial Oversight Committee, the REC and the relevant regulatory bodies. This includes reporting serious breaches of GCP and/or the trial protocol to the REC and MHRA. A copy is sent to the University of Birmingham Clinical Research Compliance Team at the time of reporting to the REC and/or relevant regulatory bodies.

11.5 Data Handling and Analysis

Paper CRFs must be completed, signed/ dated and either entered directly online or returned to the **RATE-AF** Trial Office by the PI or an authorised member of the site research team (as delegated on the **RATE-AF** Trial Signature and Delegation Log) within the timeframe listed in **Table 5**. Copies of all completed CRFs should be filed in the site file. Entries on paper CRFs should be made in ballpoint pen, in black ink, and must be legible. Any errors should be crossed out with a single stroke, the correction inserted and the change initialled and dated. If it is not obvious why a change has been made, an explanation should be written next to the change.

CRFs can be entered online at http://www.bctu.bham.ac.uk/RATEAF. Authorised staff at sites will require an individual secure login username and password to access this online data entry system.

Data reported on each CRF should be consistent with the source data or the discrepancies should be explained. If information is not known, this must be clearly indicated on the CRF. All missing and ambiguous data will be queried. All sections are to be completed.

CRF versions may be updated by the **RATE-AF** Trial Office, as appropriate, throughout the duration of the trial. Whilst this will not constitute a protocol amendment, new versions of the CRFs must be implemented by participating sites immediately on receipt.

For peer review only - http://bmjopen.bmj.com/site/about/guidelines.xhtml

It will be the responsibility of the Principal Investigator to ensure the accuracy of all data entered in the CRFs. The **RATE-AF** Trial Signature & Delegation Log will identify all those personnel with responsibilities for data collection.

Access to data, including the final trial dataset, will be limited to members of the Research Team.

The investigator(s)/institution(s) will permit trial-related monitoring, audits, REC review, and regulatory inspection(s), providing direct access to source data/documents. Trial participants are informed of this during the informed consent discussion and will consent to provide access to their medical notes.

Table 5: Data Collection Forms

Form Name	Schedule for submission
**Randomisation Form**	Collected at randomisation
**Baseline and Follow-Up CRFs**	As soon as possible after each follow-up assessment time point
**Serious Adverse Event Form**	Faxed within 24hrs of research staff at site becoming aware of event

11.6 End of Trial

The end of trial will be 30 days after the last data capture. This will allow sufficient time for the completion of protocol procedures, data collection and data input. The Trials Office will notify the MHRA and REC that the trial has ended within 90 days of the end of trial. Where the trial has terminated early, the Trials Office will inform the MHRA and REC within 15 days of the end of trial. The Trials Office will provide them with a summary of the clinical trial report within 12 months of the end of trial.

A copy of the end of trial notification as well as the summary report is also sent to the University of Birmingham Research Governance Team at the time of sending these are sent to the MHRA and REC.

11.7 Archiving

Archiving will be authorised by the BCTU on behalf of the Sponsor following submission of the end of trial report.

For peer review only - http://bmjopen.bmj.com/site/about/guidelines.xhtml

Principal Investigators are responsible for the secure archiving of essential trial documents (for their site) as per their NHS Trust policy. All essential documents will be archived for a minimum of 25 years after completion of trial.

Destruction of essential documents will require authorisation from the BCTU on behalf of the Sponsor.

12 Statistical Considerations

12.1 Outcome measures

12.1.1 Primary Outcome

Patient-reported quality of life (QoL) - SF-36 physical component summary score at six months

12.1.2 Secondary Outcomes

Patient-reported QoL:

- SF-36 global and domain-specific scores at 6 and 12 months
- EQ-5D-5L summary index and visual analogue scale at six and twelve months
- AFEQT overall score at six and twelve months

Cardiac function:

- Echocardiographic LVEF at 12 months
- Diastolic function (E/e' and composite of diastolic indices) at 12 months
- Functional assessment:
- Six-minute walking distance at 6 and 12 months
- Change in European Heart Rhythm Association (EHRA) class at 6 and 12 months

Biomarkers:

- Change in B-type natriuretic peptide (BNP) levels at 6 months
- Change in heart rate using 24-hour ambulatory ECG

12.1.3 Feasibility Outcomes

- Recruitment target of 3 patients per week across all participating centres.
- Compliance and reasons for non-compliance
- Number of withdrawals and losses to follow-up (with reasons)
- Drug discontinuation rate and adverse reactions requiring drug discontinuation.
- Number of patients needing therapy-induced requirement for additional treatment
- Population-specific standard deviations (SD) and proportions

For peer review only - http://bmjopen.bmj.com/site/about/guidelines.xhtml

- *SD of SF36 physical functioning score at 6 and 12 months*
- *SD of SF36 overall score at 6 and 12 months*
- *SD of AFEQT overall score at 6 and 12 months*
- *SD of LVEF and E/e' score at 6 and 12 months*
- *Unplanned hospitalisation admissions rates*

- Cardiovascular Events (particularly mortality, thromboembolic events, myocardial infarction and cardiovascular interventions)

The final analyses will follow a pre-specified analysis plan, drafted in conjunction with the Birmingham Clinical Trials Unit and submitted to the steering committee at the penultimate meeting. We intend to perform a primary intention-to-treat analysis, in addition to a per-protocol analysis.

Any additional (exploratory) analyses will be explicitly labelled as such in any subsequent manuscript.

12.2 Power Calculations

Randomising 144 patients we can assume an 85% power to detect an effect size of half a standard deviation in a continuous outcome measure of QoL (two-sided alpha of 0.05). A sample size of 160 patients would account for an estimated 10% loss to follow-up (including withdrawal and death prior to 12-month assessment). There is some evidence from existing research to support the notion that the treatment effect could be this large. The mean SF-36 role-physical score from the rate-control arm of the RACE study was 47, with a 17% improvement with rate-control over time.[62] In another study, CCB resulted in 22% improvement in a proprietary symptom-checklist, compared to a non-significant 8% change in those assigned to beta-blockers (SD 10-points in both groups). These values are also consistent with a 17% improvement in SF-36 scores in a third trial, PIAF.[63] Thus whilst it is possible that this trial may provide a clear indication of effect, it is accepted that the trial will be underpowered to detect smaller differences, reinforcing the requirement for a larger definitive trial which would also be powered to assess impact on clinical event rates.

12.3 Statistical analysis

A separate Statistical Analysis Plan for the RATE-AF trial provides a detailed description of the planned statistical analyses. A brief outline of these analyses is given below.

The primary comparison groups will be composed of those who are randomised to digoxin group and those randomised to the beta-blockers group. All analyses will be based on the intention to treat principle, with all patients analysed in the arms to which they were allocated irrespective of compliance with the randomised allocated treatment, and all patients will be included in the analyses. We will, as a sensitivity analysis, conduct per-protocol analyses, where appropriate.

For peer review only - http://bmjopen.bmj.com/site/about/guidelines.xhtml

For all analyses, a p-value <0.05 will be considered statistically significant.

12.3.1 Primary outcome analysis

The primary outcome for this trial is the continuous SF36 physical functioning domain score at 6 months. This outcome will be analysed using an ANCOVA model adjusting for treatment arm, baseline score and all minimisation variables. Results will be presented as difference in means and 95% confidence intervals.

12.3.2 Feasibility and Secondary outcomes analysis

The feasibility and secondary outcomes for the trial comprise of a combination of both continuous and categorical (dichotomous) data items.

Categorical endpoints:

For outcomes which are categorical (dichotomous) in nature, the proportion of participants and percentages will be analysed between arms.

Logistic/Log-binomial regression models will be fitted (where appropriate) to adjust for treatment arm, baseline scores and all minimisation variables.

Results will be presented as odds ratios/relative risks and 95% confidence intervals.

Continuous endpoints:

Any outcomes that are continuous in nature will be analysed in the same way as the primary outcome.

12.3.3 Missing data and sensitivity analyses

Primary analysis will concentrate on available data only, with no attempt made to impute missing data. Where appropriate, sensitivity analyses will be carried out to examine the possible impact of missing data on the results (full details will be discussed within the Statistical Analysis Plan).

12.3.4 Interim analyses and Stopping rules

Analysis of the data with respect to efficacy and safety will be performed at 12 months and sent to Data Monitoring Committee (DMC); see **Section 16**. The DMC will outline and agree the stopping rules for the trial which will be documented in the DMC charter. It is likely that the Haybittle-Peto boundary will be used. This states that if an interim analysis shows a probability of less than 0.001 that the treatments are different, then the trial should be stopped early. This will be used alongside data on important secondary endpoints and all other relevant evidence. A DMC report and charter outlining the terms of reference (including information on stopping rules) will be agreed with the DMEC.

For peer review only - http://bmjopen.bmj.com/site/about/guidelines.xhtml

12.4 Final analysis

The final analysis for the RATE-AF trial will occur once the last randomised participant completes their 12-month follow-up.

13 Ethics and Regulatory Requirements

The trial will be performed in accordance with the recommendations guiding physicians in biomedical research involving human participants, adopted by the 18th World Medical Association General Assembly, Helsinki, Finland, June 1964, amended at the 48th World Medical Association General Assembly, Somerset West, Republic of South Africa, October 1996 (website: http://www.wma.net/en/30publications/10policies/b3/index.html).

The trial will be conducted in accordance with the Research Governance Framework for Health and Social Care, the applicable UK Statutory Instruments, (which include the Medicines for Human Use Clinical Trials 2004 and subsequent amendments and the Data Protection Act 1998 and Human Tissue Act 2008, EU Clinical Trials Directive and amendment Regulations as appropriate) and Guidelines for Good Clinical Practice (GCP). This trial will be carried out under a Clinical Trial Authorisation in accordance with the Medicines for Human Use Clinical Trials regulations. The protocol will be submitted to and approved by the REC prior to circulation.

Before any participants are enrolled into the trial, the Principal Investigator at each site is required to obtain local R&D approval. Sites will not be permitted to enrol participants until written confirmation of R&D approval is received by the Principal Investigator.

It is the responsibility of the Principal Investigator to ensure that all subsequent amendments gain the necessary local approval. This does not affect the individual clinicians' responsibility to take immediate action if thought necessary to protect the health and interest of individual participants.

Within 90 days after the end of the trial, the Chief Investigator/Sponsor will ensure that the REC and the MHRA are notified that the trial has finished. If the trial is terminated prematurely, those reports will be made within 15 days after the end of the trial. The Chief Investigator will provide the Sponsor with a summary report of the clinical trial, which will then be submitted to the MHRA and REC within one year after the end of the trial.

14 Oversight Committees

14.1 Trial Management Group

The Trial Management Group (TMG) will comprise the CI, other lead investigators (clinical and non-clinical) and members of the BCTU. The TMG will be responsible for the day-to-day running and management of **RATE-AF**. The TMG will convene at regular intervals.

For peer review only - http://bmjopen.bmj.com/site/about/guidelines.xhtml

14.2 Trial Oversight Committee

A joint oversight committee comprising a Trial Steering Committee (TSC) and Data Monitoring Committee (DMC) will be engaged for this trial.

The role of the TSC is to provide the overall supervision of the trial. The TSC will monitor trial progress and conduct and advice on scientific credibility. The TSC will consider and act, as appropriate, upon the recommendations of the Data Monitoring Committee. Further details of the remit and role of the TSC are available in the TSC Charter.

An independent DMC will be established to oversee the safety of participants in the trial. The DMC will meet prior to the trial opening to enrolment, and then meet at least annually, or as per a timetable agreed by the DMC prior to trial commencement. Data analyses will be supplied in confidence to the DMC, which will be asked to give advice on whether the accumulated data from the trial, together with the results from other relevant research, justifies the continuing recruitment of further participants. The DMC will operate in accordance with the trial specific charter.

14.3 Protocol amendments

Where important protocol modifications are required as a result of oversight (for example, changes to eligibility criteria, outcomes or analyses), this information will be communicated to relevant parties, such as investigators, the REC, trial registries and regulators.

15 Finance

The **RATE-AF** Trial is funded through a Career Development Fellowship awarded to the Chief Investigator by the National Institute for Health Research (NIHR).

16 Confidentiality and Data Protection

Personal data recorded on all documents will be regarded as strictly confidential and will be handled and stored in accordance with the Data Protection Act 1998.

Participants will be identified using their unique trial identification number, date of birth and hospital number on the CRFs. and correspondence between the Trials Office and the participating site. Participants will give their explicit consent for the movement of their consent form, giving permission for the Trials Office to be sent a copy. This will be used to perform in-house monitoring of the consent process.

The Investigator must maintain documents not for submission to the Trials Office (e.g. Participant Identification Logs) in strict confidence. In the case of specific issues and/or queries from the

For peer review only - http://bmjopen.bmj.com/site/about/guidelines.xhtml

regulatory authorities, it will be necessary to have access to the complete trial records, provided that participant confidentiality is protected.

The Trials Office will maintain the confidentiality of all participants' data and will not disclose information by which participants may be identified to any third party other than those directly involved in the treatment of the participant and organisations for which the participant has given explicit consent for data transfer (e.g. competent authority, sponsor). Representatives of the **RATE-AF** Trials Office and sponsor may be required to have access to participant's notes for quality assurance purposes but participants should be reassured that their confidentiality will be respected at all times.

17 Insurance and Indemnity

The University of Birmingham has in place Clinical Trials indemnity coverage for this trial which provides cover to the University for harm which comes about through the University's, or its staff's, negligence in relation to the design or management of the trial and may alternatively, and at the University's discretion provide cover for non-negligent harm to participants.

With respect to the conduct of the trial at the Clinical Site and other clinical care of the patient, responsibility for the care of the patients remains with the NHS organisation responsible for the Clinical Site and is therefore indemnified through the NHS Litigation Authority.

The University of Birmingham is independent of any pharmaceutical company, and as such it is not covered by the Association of the British Pharmaceutical Industry (ABPI) guidelines for participant compensation.

18 Dissemination and Publication

Regular newsletters will keep collaborators informed of trial progress and regular meetings will be held to report the progress of the trial and to address any problems encountered in the conduct of the trial. The CI will coordinate dissemination of data from this trial. All publications and presentations, including abstracts, relating to the main trial will be authorised by the **RATE-AF** TMG. The results of the analysis will be published in the name of the **RATE-AF** Collaborative Group in a peer reviewed journal (provided that this does not conflict with the journal's policy).

Named authors must satisfy the International Committee of Medical Journal Editors (ICMJE) criteria for authorship (contribute to drafting of the article or revision for important intellectual content), provide timely approval of the final version to be published and supply detailed statements on any potential conflict of interest or financial relationship (http://www.icmje.org/). Members of the group who do not fulfil ICMJE criteria for authorship will be listed in the article appendix. Trial participants will be sent a lay summary of the final results of the trial, which will contain a reference to the full paper.

For peer review only - http://bmjopen.bmj.com/site/about/guidelines.xhtml

19 Statement of Compliance

The **RATE-AF** trial will be conducted in compliance with the approved protocol, EU GCP and the applicable regulatory requirements.

For peer review only

For peer review only - http://bmjopen.bmj.com/site/about/guidelines.xhtml

20 References

1. Camm AJ, Kirchhof P, Lip GY, Schotten U, Savelieva I, Ernst S, Van Gelder IC, Al-Attar N, Hindricks G, Prendergast B, Heidbuchel H, Alfieri O, Angelini A, Atar D, Colonna P, De Caterina R, De Sutter J, Goette A, Gorenek B, Heldal M, Hohloser SH, Kolh P, Le Heuzey JY, Ponikowski P, Rutten FH. Guidelines for the management of atrial fibrillation: the Task Force for the Management of Atrial Fibrillation of the European Society of Cardiology (ESC). *Eur Heart J*. 2010;31:2369-2429

2. Anderson JL, Halperin JL, Albert NM, Bozkurt B, Brindis RG, Curtis LH, DeMets D, Guyton RA, Hochman JS, Kovacs RJ, Ohman EM, Pressler SJ, Sellke FW, Shen W-K. Management of patients with atrial fibrillation (Compilation of 2006 ACCF/AHA/ESC and 2011 ACCF/AHA/HRS recommendations). *Circulation*. 2013;127:1916-1926

3. Camm AJ, Lip GY, De Caterina R, Savelieva I, Atar D, Hohnloser SH, Hindricks G, Kirchhof P, E. S. C. Committee for Practice Guidelines. 2012 focused update of the ESC Guidelines for the management of atrial fibrillation: an update of the 2010 ESC Guidelines for the management of atrial fibrillation. Developed with the special contribution of the European Heart Rhythm Association. *Eur Heart J*. 2012;33:2719-2747

4. National Institute for Health and Care Excellence. Atrial fibrillation: the management of atrial fibrillation. *NICE clinical guideline 180*. 2014; Accessed 15/09/2014; http://www.nice.org.uk/guidance/cg180/

5. Kotecha D, Holmes J, Krum H, Altman DG, Manzano L, Cleland JG, Lip GY, Coats AJ, Andersson B, Kirchhof P, von Lueder TG, Wedel H, Rosano G, Shibata MC, Rigby A, Flather MD, Beta-Blockers in Heart Failure Collaborative Group. Efficacy of beta blockers in patients with heart failure plus atrial fibrillation: an individual-patient data meta-analysis. *Lancet*. 2014;384:2235-2243

6. Ziff OJ, Lane DA, Samra M, Griffith M, Kirchhof P, Lip GY, Steeds RP, Townend J, Kotecha D. Safety and efficacy of digoxin: systematic review and meta-analysis of observational and controlled trial data. *BMJ*. 2015;351:h4451

7. Kotecha D, Banerjee A, Lip GY. Increased stroke risk in atrial fibrillation patients with heart failure: does ejection fraction matter? *Stroke*. 2015;46:608-609

8. Kotecha D, Kirchhof P. Rate and rhythm control have comparable effects on mortality and stroke in atrial fibrillation but better data are needed. *Evid Based Med*. 2014;19:222-223

9. Kotecha D, Piccini JP. Atrial fibrillation in heart failure: what should we do? *Eur Heart J*. 2015;36:3250-3257

10. Senoo K, Lip GY, Lane DA, Buller HR, Kotecha D. Residual risk of stroke and death in anticoagulated patients according to the type of atrial fibrillation: AMADEUS Trial. *Stroke*. 2015;46:2523-2528

11. Kotecha D, Chudasama R, Lane DA, Kirchhof P, Lip GY. Atrial fibrillation and heart failure due to reduced versus preserved ejection fraction: A systematic review and meta-analysis of death and adverse outcomes. *Int J Cardiol*. 2016;203:660-666

12. Arain M, Campbell MJ, Cooper CL, Lancaster GA. What is a pilot or feasibility study? A review of current practice and editorial policy. *BMC Med Res Methodol*. 2010;10:67

13. Thabane L, Ma J, Chu R, Cheng J, Ismaila A, Rios LP, Robson R, Thabane M, Giangregorio L, Goldsmith CH. A tutorial on pilot studies: the what, why and how. *BMC Med Res Methodol*. 2010;10:1

14. Heeringa J, van der Kuip DAM, Hofman A, Kors JA, van Herpen G, Stricker BHC, Stijnen T, Lip GYH, Witteman JCM. Prevalence, incidence and lifetime risk of atrial fibrillation: the Rotterdam study. *Eur Heart J*. 2006;27:949-953

For peer review only - http://bmjopen.bmj.com/site/about/guidelines.xhtml

15. Colilla S, Crow A, Petkun W, Singer DE, Simon T, Liu X. Estimates of Current and Future Incidence and Prevalence of Atrial Fibrillation in the U.S. Adult Population. *Am J Cardiol*. 2013;112:1142-1147

16. Kim MH, Johnston SS, Chu BC, Dalal MR, Schulman KL. Estimation of total incremental health care costs in patients with atrial fibrillation in the United States. *Circ Cardiovasc Qual Outcomes*. 2011;4:313-320

17. Benjamin EJ, Wolf PA, D'Agostino RB, Silbershatz H, Kannel WB, Levy D. Impact of Atrial Fibrillation on the Risk of Death: The Framingham Heart Study. *Circulation*. 1998;98:946-952

18. Stewart S, Hart CL, Hole DJ, McMurray JJV. A population-based study of the long-term risks associated with atrial fibrillation: 20-year follow-up of the Renfrew/Paisley study. *Am J Med*. 2002;113:359-364

19. Marijon E, Le Heuzey JY, Connolly S, Yang S, Pogue J, Brueckmann M, Eikelboom J, Themeles E, Ezekowitz M, Wallentin L, Yusuf S. Causes of death and influencing factors in patients with atrial fibrillation: a competing-risk analysis from the randomized evaluation of long-term anticoagulant therapy study. *Circulation*. 2013;128:2192-2201

20. Chen LY, Sotoodehnia N, Buzkova P, Lopez FL, Yee LM, Heckbert SR, Prineas R, Soliman EZ, Adabag S, Konety S, Folsom AR, Siscovick D, Alonso A. Atrial fibrillation and the risk of sudden cardiac death: the atherosclerosis risk in communities study and cardiovascular health study. *JAMA Intern Med*. 2013;173:29-35

21. Dorian P, Jung W, Newman D, Paquette M, Wood K, Ayers GM, Camm J, Akhtar M, Luderitz B. The impairment of health-related quality of life in patients with intermittent atrial fibrillation: implications for the assessment of investigational therapy. *J Am Coll Cardiol*. 2000;36:1303-1309

22. Thrall G, Lane D, Carroll D, Lip GYH. Quality of Life in Patients with Atrial Fibrillation: A Systematic Review. *Am J Med*. 2006;119:448.e441-419

23. Sears SF, Serber ER, Alvarez LG, Schwartzman DS, Hoyt RH, Ujhelyi MR. Understanding Atrial Symptom Reports: Objective versus Subjective Predictors. *Pacing Clin Electrophysiol*. 2005;28:801-807

24. Dries D, Exner D, Gersh B, Domanski M, Waclawiw M, Stevenson L. Atrial fibrillation is associated with an increased risk for mortality and heart failure progression in patients with asymptomatic and symptomatic left ventricular systolic dysfunction: a retrospective analysis of the SOLVD trials. *J Am Coll Cardiol*. 1998;32:695-703

25. Wang TJ, Larson MG, Levy D, Vasan RS, Leip EP, Wolf PA, D'Agostino RB, Murabito JM, Kannel WB, Benjamin EJ. Temporal Relations of Atrial Fibrillation and Congestive Heart Failure and Their Joint Influence on Mortality: The Framingham Heart Study. *Circulation*. 2003;107:2920-2925

26. The Atrial Fibrillation Follow-up Investigation of Rhythm Management (AFFIRM) Investigators. A Comparison of Rate Control and Rhythm Control in Patients with Atrial Fibrillation. *N Engl J Med*. 2002;347:1825-1833

27. Van Gelder IC, Hagens VE, Bosker HA, Kingma JH, Kamp O, Kingma T, Said SA, Darmanata JI, Timmermans AJM, Tijssen JGP, Crijns HJGM. A Comparison of Rate Control and Rhythm Control in Patients with Recurrent Persistent Atrial Fibrillation. *N Engl J Med*. 2002;347:1834-1840

28. Olshansky B, Rosenfeld LE, Warner AL, Solomon AJ, O'Neill G, Sharma A, Platia E, Feld GK, Akiyama T, Brodsky MA, Greene HL. The Atrial Fibrillation Follow-up Investigation of Rhythm Management (AFFIRM) study: approaches to control rate in atrial fibrillation. *J Am Coll Cardiol*. 2004;43:1201-1208

29. Hohnloser SH, Kuck KH, Lilienthal J. Rhythm or rate control in atrial fibrillation--Pharmacological Intervention in Atrial Fibrillation (PIAF): a randomised trial. *Lancet*. 2000;356:1789-1794

For peer review only - http://bmjopen.bmj.com/site/about/guidelines.xhtml

30. Carlsson J, Miketic S, Windeler J, Cuneo A, Haun S, Micus S, Walter S, Tebbe U, Investigators S. Randomized trial of rate-control versus rhythm-control in persistent atrial fibrillation: the Strategies of Treatment of Atrial Fibrillation (STAF) study. *J Am Coll Cardiol*. 2003;41:1690-1696

31. de Denus S, Sanoski CA, Carlsson J, Opolski G, Spinler SA. Rate vs rhythm control in patients with atrial fibrillation: a meta-analysis. *Arch Intern Med*. 2005;165:258-262

32. Chatterjee S, Sardar P, Lichstein E, Mukherjee D, Aikat S. Pharmacologic rate versus rhythm-control strategies in atrial fibrillation: an updated comprehensive review and meta-analysis. *PACE*. 2013;36:122-133

33. Shelton RJ, Clark AL, Goode K, Rigby AS, Houghton T, Kaye GC, Cleland JG. A randomised, controlled study of rate versus rhythm control in patients with chronic atrial fibrillation and heart failure: (CAFE-II Study). *Heart*. 2009;95:924-930

34. Roy D, Talajic M, Nattel S, Wyse DG, Dorian P, Lee KL, Bourassa MG, Arnold JMO, Buxton AE, Camm AJ, Connolly SJ, Dubuc M, Ducharme A, Guerra PG, Hohnloser SH, Lambert J, Le Heuzey J-Y, O'Hara G, Pedersen OD, Rouleau J-L, Singh BN, Stevenson LW, Stevenson WG, Thibault B, Waldo AL. Rhythm Control versus Rate Control for Atrial Fibrillation and Heart Failure. *N Engl J Med*. 2008;358:2667-2677

35. Kong MH, Shaw LK, O'Connor C, Califf RM, Blazing MA, Al-Khatib SM. Is rhythm-control superior to rate-control in patients with atrial fibrillation and diastolic heart failure? *Ann Noninvasive Electrocardiol*. 2010;15:209-217

36. Wazni O, Wilkoff B, Saliba W. Catheter Ablation for Atrial Fibrillation. *N Engl J Med*. 2011;365:2296-2304

37. Jones DG, Haldar SK, Hussain W, Sharma R, Francis DP, Rahman-Haley SL, McDonagh TA, Underwood SR, Markides V, Wong T. A randomized trial to assess catheter ablation versus rate control in the management of persistent atrial fibrillation in heart failure. *J Am Coll Cardiol*. 2013;61:1894-1903

38. Kirchhof P, Breithardt G, Camm AJ, Crijns HJ, Kuck KH, Vardas P, Wegscheider K. Improving outcomes in patients with atrial fibrillation: rationale and design of the Early treatment of Atrial fibrillation for Stroke prevention Trial. *Am Heart J*. 2013;166:442-448

39. Steg PG, Alam S, Chiang C-E, Gamra H, Goethals M, Inoue H, Krapf L, Lewalter T, Merioua I, Murin J, Naditch-Brûlé L, Ponikowski P, Rosenqvist M, Silva-Cardoso J, Zharinov O, Brette S, Neill JO. Symptoms, functional status and quality of life in patients with controlled and uncontrolled atrial fibrillation: data from the RealiseAF cross-sectional international registry. *Heart*. 2012;98:195-201

40. Nabauer M, Gerth A, Limbourg T, Schneider S, Oeff M, Kirchhof P, Goette A, Lewalter T, Ravens U, Meinertz T, Breithardt G, Steinbeck G. The Registry of the German Competence NETwork on Atrial Fibrillation: patient characteristics and initial management. *Europace*. 2009;11:423-434

41. Segal JB, McNamara RL, Miller MR, Kim N, Goodman SN, Powe NR, Robinson K, Yu D, Bass EB. The evidence regarding the drugs used for ventricular rate control. *J Fam Practice*. 2000;49:47-59

42. Farshi R, Kistner D, Sarma JSM, Longmate JA, Singh BN. Ventricular rate control in chronic atrial fibrillation during daily activity and programmed exercise: a crossover open-label study of five drug regimens. *J Am Coll Cardiol*. 1999;33:304-310

43. Koh KK, Kwon KS, Park HB, Baik SH, Park SJ, Lee KH, Kim EJ, Kim SH, Cho SK, Kim SS. Efficacy and safety of digoxin alone and in combination with low-dose diltiazem or betaxolol to control ventricular rate in chronic atrial fibrillation. *Am J Cardiol*. 1995;75:88-90

44. Nikolaidou T, Channer KS. Chronic atrial fibrillation: a systematic review of medical heart rate control management. *Postgrad Med J*. 2009;85:303-312

For peer review only - http://bmjopen.bmj.com/site/about/guidelines.xhtml

45. Ulimoen SR, Enger S, Carlson J, Platonov PG, Pripp AH, Abdelnoor M, Arnesen H, Gjesdal K, Tveit A. Comparison of four single-drug regimens on ventricular rate and arrhythmia-related symptoms in patients with permanent atrial fibrillation. *Am J Cardiol*. 2013;111:225-230

46. Ulimoen SR, Enger S, Pripp AH, Abdelnoor M, Arnesen H, Gjesdal K, Tveit A. Calcium channel blockers improve exercise capacity and reduce N-terminal Pro-B-type natriuretic peptide levels compared with beta-blockers in patients with permanent atrial fibrillation. *Eur Heart J*. 2014;35:517-524

47. Khand AU, Rankin AC, Martin W, Taylor J, Gemmell I, Cleland JG. Carvedilol alone or in combination with digoxin for the management of atrial fibrillation in patients with heart failure? *J Am Coll Cardiol*. 2003;42:1944-1951

48. Vamos M, Erath JW, Hohnloser SH. Digoxin-associated mortality: a systematic review and meta-analysis of the literature. *Eur Heart J*. 2015; In press: 10.1093/eurheartj/ehv143

49. Gheorghiade M, Fonarow GC, van Veldhuisen DJ, Cleland JG, Butler J, Epstein AE, Patel K, Aban IB, Aronow WS, Anker SD, Ahmed A. Lack of evidence of increased mortality among patients with atrial fibrillation taking digoxin: findings from post hoc propensity-matched analysis of the AFFIRM trial. *Eur Heart J*. 2013;34:1489-1497

50. Friberg L, Hammar N, Rosenqvist M. Digoxin in atrial fibrillation: report from the Stockholm Cohort study of Atrial Fibrillation (SCAF). *Heart*. 2010;96:275-280

51. Flory JH, Ky B, Haynes K, S MB, Munson J, Rowan C, Strom BL, Hennessy S. Observational cohort study of the safety of digoxin use in women with heart failure. *BMJ Open*. 2012;2:e000888

52. Andrey JL, Romero S, Garcia-Egido A, Escobar MA, Corzo R, Garcia-Dominguez G, Lechuga V, Gomez F. Mortality and morbidity of heart failure treated with digoxin. A propensity-matched study. *Int J Clin Pract*. 2011;65:1250-1258

53. Lewis RV, Irvine N, McDevitt DG. Relationships between heart rate, exercise tolerance and cardiac output in atrial fibrillation: the effects of treatment with digoxin, verapamil and diltiazem. *Eur Heart J*. 1988;9:777-781

54. Tsuneda T, Yamashita T, Fukunami M, Kumagai K, Niwano S, Okumura K, Inoue H. Rate control and quality of life in patients with permanent atrial fibrillation: the Quality of Life and Atrial Fibrillation (QOLAF) Study. *Circ J*. 2006;70:965-970

55. Van Gelder IC, Groenveld HF, Crijns HJGM, Tuininga YS, Tijssen JGP, Alings AM, Hillege HL, Bergsma-Kadijk JA, Cornel JH, Kamp O, Tukkie R, Bosker HA, Van Veldhuisen DJ, Van den Berg MP. Lenient versus Strict Rate Control in Patients with Atrial Fibrillation. *N Engl J Med*. 2010;362:1363-1373

56. Groenveld HF, Crijns HJGM, Van den Berg MP, Van Sonderen E, Alings AM, Tijssen JGP, Hillege HL, Tuininga YS, Van Veldhuisen DJ, Ranchor AV, Van Gelder IC. The Effect of Rate Control on Quality of Life in Patients With Permanent Atrial Fibrillation: Data From the RACE II (Rate Control Efficacy in Permanent Atrial Fibrillation II) Study. *J Am Coll Cardiol*. 2011;58:1795-1803

57. Mulder BA, Van Veldhuisen DJ, Crijns HJGM, Tijssen JGP, Hillege HL, Alings M, Rienstra M, Groenveld HF, Van den Berg MP, Van Gelder IC. Lenient vs. strict rate control in patients with atrial fibrillation and heart failure: a post-hoc analysis of the RACE II study. *Eur J Heart Fail*. 2013;15:1311-1318

58. Groenveld HF, Tijssen JG, Crijns HJ, Van den Berg MP, Hillege HL, Alings M, Van Veldhuisen DJ, Van Gelder IC. Rate control efficacy in permanent atrial fibrillation: successful and failed strict rate control against a background of lenient rate control: data from RACE II (Rate Control Efficacy in Permanent Atrial Fibrillation). *J Am Coll Cardiol*. 2013;61:741-748

59. US Department of Health and Human Services Food and Drug Administration. Guidance for industry: patient-reported outcome measures: use in medical product development to support

For peer review only - http://bmjopen.bmj.com/site/about/guidelines.xhtml

labeling claims. www.fda.gov/downloads/Drugs/GuidanceComplianceRegulatoryInformation/Guidances/UCM193282.pdf. 2009

60. Rienstra M, Lubitz SA, Mahida S, Magnani JW, Fontes JD, Sinner MF, Van Gelder IC, Ellinor PT, Benjamin EJ. Symptoms and Functional Status of Patients With Atrial Fibrillation: State of the Art and Future Research Opportunities. *Circulation*. 2012;125:2933-2943

61. Pepine CJ. Effects of pharmacologic therapy on health-related quality of life in elderly patients with atrial fibrillation: a systematic review of randomized and nonrandomized trials. *Clin Med Insights Cardiol*. 2013;7:1-20

62. Hagens VE, Ranchor AV, Van Sonderen E, Bosker HA, Kamp O, Tijssen JGP, Kingma JH, Crijns HJGM, Van Gelder IC. Effect of rate or rhythm control on quality of life in persistent atrial fibrillation: Results from the Rate Control Versus Electrical Cardioversion (RACE) study. *J Am Coll Cardiol*. 2004;43:241-247

63. Grönefeld GC, Lilienthal J, Kuck K-H, Hohnloser SH. Impact of rate versus rhythm control on quality of life in patients with persistent atrial fibrillation: Results from a prospective randomized study. *Eur Heart J*. 2003;24:1430-1436

64. Ware Jr JE, Gandek B. Overview of the SF-36 Health Survey and the International Quality of Life Assessment (IQOLA) Project. *J Clin Epidemiol*. 1998;51:903-912

65. Herdman M, Gudex C, Lloyd A, Janssen M, Kind P, Parkin D, Bonsel G, Badia X. Development and preliminary testing of the new five-level version of EQ-5D (EQ-5D-5L). *Qual Life Res*. 2011;20:1727-1736

66. Devlin NJ, Krabbe PF. The development of new research methods for the valuation of EQ-5D-5L. *Eur J Health Econ*. 2013;14 Suppl 1:S1-3

67. Spertus J, Dorian P, Bubien R, Lewis S, Godejohn D, Reynolds MR, Lakkireddy DR, Wimmer AP, Bhandari A, Burk C. Development and validation of the Atrial Fibrillation Effect on QualiTy-of-Life (AFEQT) Questionnaire in patients with atrial fibrillation. *Circ Arrhythm Electrophysiol*. 2011;4:15-25

68. Dorian P, Burk C, Mullin CM, Bubien R, Godejohn D, Reynolds MR, Lakkireddy DR, Wimmer AP, Bhandari A, Spertus J. Interpreting changes in quality of life in atrial fibrillation: How much change is meaningful? *Am Heart J*. 2013;166:381-387.e388

69. Joint Formulary Committee. *British National Formulary*. London: BMJ Group and Pharmaceutical Press; 2013.

70. American Hospital Formulary Service. *Drug Information*. Bethesda: American Society of Health-System Pharmacists; 2013.

71. The Digitalis Investigation Group. The effect of digoxin on mortality and morbidity in patients with heart failure. *N Engl J Med*. 1997;336:525-533

72. Magnani B, Malini PL. Cardiac glycosides. Drug interactions of clinical significance. *Drug Safety*. 1995;12:97-109

73. Ko DT, Hebert PR, Coffey CS, Sedrakyan A, Curtis JP, Krumholz HM. Beta-blocker therapy and symptoms of depression, fatigue, and sexual dysfunction. *JAMA*. 2002;288:351-357

74. Terwee C, Mokkink L, Knol D, Ostelo RJG, Bouter L, Vet HW. Rating the methodological quality in systematic reviews of studies on measurement properties: a scoring system for the COSMIN checklist. *Qual Life Res*. 2012;21:651-657

75. Streiner DL, Norman GR. *Health measurement scales : a practical guide to their development and use*. Oxford ; New York: Oxford University Press; 2008.

For peer review only - http://bmjopen.bmj.com/site/about/guidelines.xhtml

76. Staniszewska S, Haywood KL, Brett J, Tutton L. Patient and public involvement in patient-reported outcome measures: evolution not revolution. *Patient*. 2012;5:79-87

77. Calvert M, Kyte D, Duffy H, Gheorghe A, Mercieca-Bebber R, Ives J, Draper H, Brundage M, Blazeby J, King M. Patient-reported outcome (PRO) assessment in clinical trials: a systematic review of guidance for trial protocol writers. *PLoS One*. 2014;9:e110216

78. McCabe PJ, Schumacher K, Barnason SA. Living with atrial fibrillation: a qualitative study. *J Cardiovasc Nurs*. 2011;26:336-344

79. Kirchhof P, Auricchio A, Bax J, Crijns H, Camm J, Diener H-C, Goette A, Hindricks G, Hohnloser S, Kappenberger L, Kuck K-H, Lip GYH, Olsson B, Meinertz T, Priori S, Ravens U, Steinbeck G, Svernhage E, Tijssen J, Vincent A, Breithardt G. Outcome parameters for trials in atrial fibrillation: Recommendations from a consensus conference organized by the German Atrial Fibrillation Competence NETwork (AFNET) and the European Heart Rhythm Association (EHRA). *Eur Heart J*. 2007;28:2803-2817

80. Haywood K, Brett J, Salek S, Marlett N, Penman C, Shklarov S, Norris C, Santana MJ, Staniszewska S. Patient and public engagement in health-related quality of life and patient-reported outcomes research: what is important and why should we care? Findings from the first ISOQOL patient engagement symposium. *Qual Life Res*. 2014: Epub ahead of print; PMID 25194573

81. Chan AW, Tetzlaff JM, Gotzsche PC, Altman DG, Mann H, Berlin JA, Dickersin K, Hrobjartsson A, Schulz KF, Parulekar WR, Krleza-Jeric K, Laupacis A, Moher D. SPIRIT 2013 explanation and elaboration: guidance for protocols of clinical trials. *BMJ*. 2013;346:e7586

82. Calvert M, Kyte D, von Hildebrand M, King M, Moher D. Putting patients at the heart of health-care research. *Lancet*. 2015;385:1073-1074

83. Kyte D, Duffy H, Fletcher B, Gheorghe A, Mercieca-Bebber R, King M, Draper H, Ives J, Brundage M, Blazeby J, Calvert M. Systematic evaluation of the patient-reported outcome (PRO) content of clinical trial protocols. *PLoS One*. 2014;9:e110229

84. Kyte D, Draper H, Calvert M. Patient-reported outcome alerts: ethical and logistical considerations in clinical trials. *JAMA*. 2013;310:1229-1230

85. Zbrozek A, Hebert J, Gogates G, Thorell R, Dell C, Molsen E, Craig G, Grice K, Kern S, Hines S. Validation of electronic systems to collect patient-reported outcome (PRO) data-recommendations for clinical trial teams: report of the ISPOR ePRO systems validation good research practices task force. *Value Health*. 2013;16:480-489

86. Su H-M, Lin T-H, Hsu P-C, Lee W-H, Chu C-Y, Lee C-S, Voon W-C, Lai W-T, Sheu S-H. Global left ventricular longitudinal systolic strain as a major predictor of cardiovascular events in patients with atrial fibrillation. *Heart*. 2013;99:1588-1596

87. Al-Omari MA, Finstuen J, Appleton CP, Barnes ME, Tsang TSM. Echocardiographic assessment of left ventricular diastolic function and filling pressure in atrial fibrillation. *Am J Cardiol*. 2008;101:1759-1765

88. Hijazi Z, Oldgren J, Siegbahn A, Granger CB, Wallentin L. Biomarkers in atrial fibrillation: a clinical review. *Eur Heart J*. 2013;34:1475-1480

89. Parvez B, Chopra N, Rowan S, Vaglio JC, Muhammad R, Roden DM, Darbar D. A common beta1-adrenergic receptor polymorphism predicts favorable response to rate-control therapy in atrial fibrillation. *J Am Coll Cardiol*. 2012;59:49-56

90. Mahmmoud YA, Shattock M, Cornelius F, Pavlovic D. Inhibition of K+ transport through Na+, K+-ATPase by capsazepine: role of membrane span 10 of the alpha-subunit in the modulation of ion gating. *PLoS One*. 2014;9:e96909

For peer review only - http://bmjopen.bmj.com/site/about/guidelines.xhtml

91. Pavlovic D, Hall AR, Kennington EJ, Aughton K, Boguslavskyi A, Fuller W, Despa S, Bers DM, Shattock MJ. Nitric oxide regulates cardiac intracellular Na(+) and Ca(2)(+) by modulating Na/K ATPase via PKCepsilon and phospholemman-dependent mechanism. *J Mol Cell Cardiol*. 2013;61:164-171

92. Pullen MA, Brooks DP, Edwards RM. Characterization of the neutralizing activity of digoxin-specific Fab toward ouabain-like steroids. *J Pharmacol Exp Ther*. 2004;310:319-325

93. Manunta P, Messaggio E, Casamassima N, Gatti G, Carpini SD, Zagato L, Hamlyn JM. Endogenous ouabain in renal Na(+) handling and related diseases. *Biochim Biophys Acta*. 2010;1802:1214-1218

94. Pavlovic D. The role of cardiotonic steroids in the pathogenesis of cardiomyopathy in chronic kidney disease. *Nephron Clin Pract*. 2014;128:11-21

95. Song H, Karashima E, Hamlyn JM, Blaustein MP. Ouabain-digoxin antagonism in rat arteries and neurones. *J Physiol*. 2014;592:941-969

96. Curtis L. Unit Costs of Health & Social Care 2012. *Personal Social Services Research Unit*. 2012:http://www.pssru.ac.uk/archive/pdf/uc/uc2012/full-with-covers.pdf

97. Jerosch-Herold M. Quantification of myocardial perfusion by cardiovascular magnetic resonance. *Journal of Cardiovascular Magnetic Resonance*. 2010;12:57

For peer review only - http://bmjopen.bmj.com/site/about/guidelines.xhtml

APPENDIX A – Dosing Schedule (Digoxin)

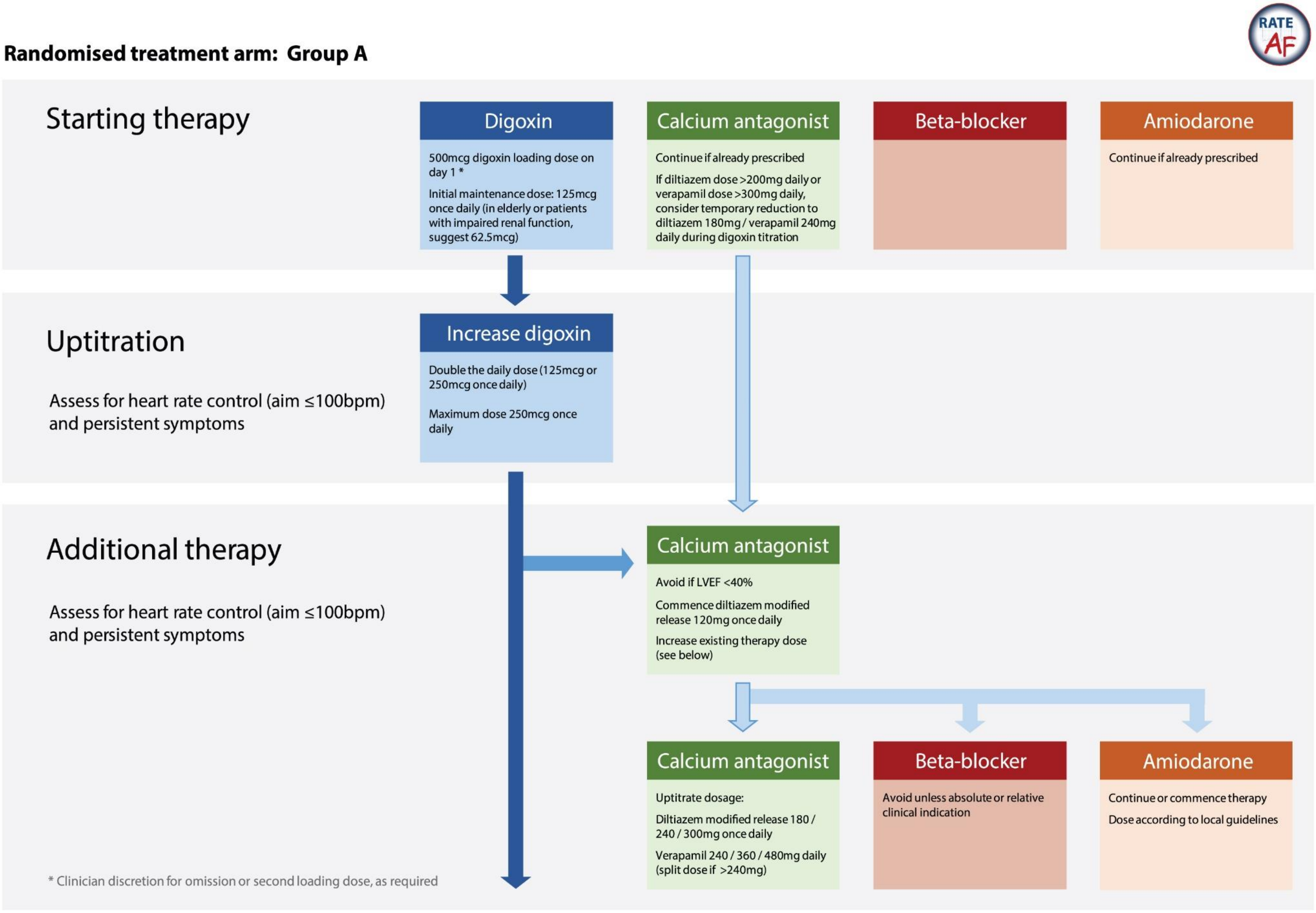

For peer review only - http://bmjopen.bmj.com/site/about/guidelines.xhtml

APPENDIX B – Dosing Schedule (Bisoprolol)

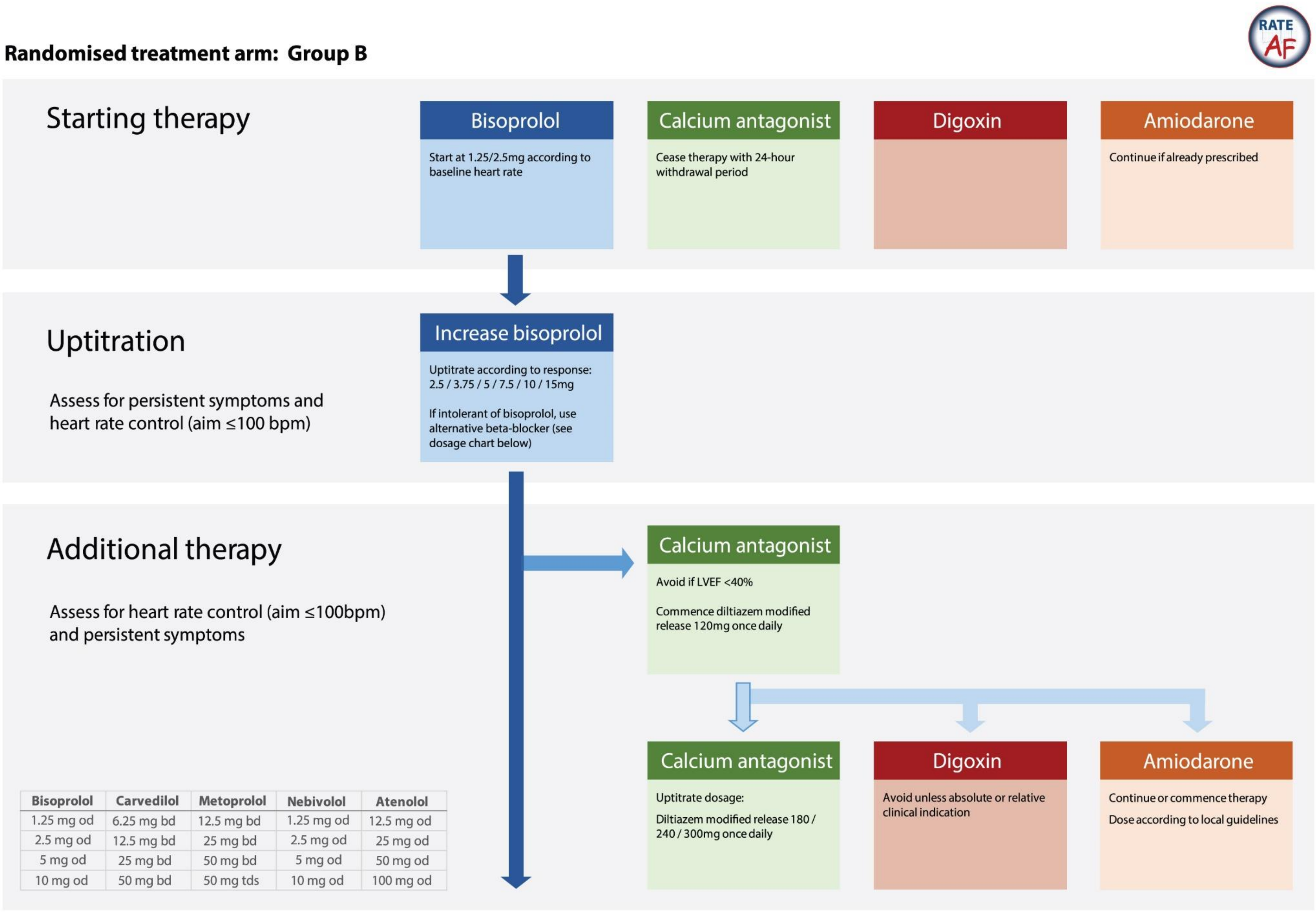

Bisoprolol	Carvedilol	Metoprolol	Nebivolol	Atenolol
1.25 mg od	6.25 mg bd	12.5 mg bd	1.25 mg od	12.5 mg od
2.5 mg od	12.5 mg bd	25 mg bd	2.5 mg od	25 mg od
5 mg od	25 mg bd	50 mg bd	5 mg od	50 mg od
10 mg od	50 mg bd	50 mg tds	10 mg od	100 mg od

For peer review only - http://bmjopen.bmj.com/site/about/guidelines.xhtml

SPIRIT

STANDARD PROTOCOL ITEMS: RECOMMENDATIONS FOR INTERVENTIONAL TRIALS

SPIRIT 2013 Checklist: Recommended items to address in a clinical trial protocol and related documents*

Section/item	Item No	Description	Addressed on page number
**Administrative information**			
Title	1	Descriptive title identifying the study design, population, interventions, and, if applicable, trial acronym	______________
Trial registration	2a	Trial identifier and registry name. If not yet registered, name of intended registry	______________
	2b	All items from the World Health Organization Trial Registration Data Set	______________
Protocol version	3	Date and version identifier	______________
Funding	4	Sources and types of financial, material, and other support	______________
Roles and responsibilities	5a	Names, affiliations, and roles of protocol contributors	______________
	5b	Name and contact information for the trial sponsor	______________
	5c	Role of study sponsor and funders, if any, in study design; collection, management, analysis, and interpretation of data; writing of the report; and the decision to submit the report for publication, including whether they will have ultimate authority over any of these activities	______________
	5d	Composition, roles, and responsibilities of the coordinating centre, steering committee, endpoint adjudication committee, data management team, and other individuals or groups overseeing the trial, if applicable (see Item 21a for data monitoring committee)	______________

For peer review only

Introduction

Background and rationale	6a	Description of research question and justification for undertaking the trial, including summary of relevant studies (published and unpublished) examining benefits and harms for each intervention	______________
	6b	Explanation for choice of comparators	______________
Objectives	7	Specific objectives or hypotheses	______________
Trial design	8	Description of trial design including type of trial (eg, parallel group, crossover, factorial, single group), allocation ratio, and framework (eg, superiority, equivalence, noninferiority, exploratory)	______________

Methods: Participants, interventions, and outcomes

Study setting	9	Description of study settings (eg, community clinic, academic hospital) and list of countries where data will be collected. Reference to where list of study sites can be obtained	______________
Eligibility criteria	10	Inclusion and exclusion criteria for participants. If applicable, eligibility criteria for study centres and individuals who will perform the interventions (eg, surgeons, psychotherapists)	______________
Interventions	11a	Interventions for each group with sufficient detail to allow replication, including how and when they will be administered	______________
	11b	Criteria for discontinuing or modifying allocated interventions for a given trial participant (eg, drug dose change in response to harms, participant request, or improving/worsening disease)	______________
	11c	Strategies to improve adherence to intervention protocols, and any procedures for monitoring adherence (eg, drug tablet return, laboratory tests)	______________
	11d	Relevant concomitant care and interventions that are permitted or prohibited during the trial	______________
Outcomes	12	Primary, secondary, and other outcomes, including the specific measurement variable (eg, systolic blood pressure), analysis metric (eg, change from baseline, final value, time to event), method of aggregation (eg, median, proportion), and time point for each outcome. Explanation of the clinical relevance of chosen efficacy and harm outcomes is strongly recommended	______________
Participant timeline	13	Time schedule of enrolment, interventions (including any run-ins and washouts), assessments, and visits for participants. A schematic diagram is highly recommended (see Figure)	______________

For peer review only - http://bmjopen.bmj.com/site/about/guidelines.xhtml

Sample size	14	Estimated number of participants needed to achieve study objectives and how it was determined, including clinical and statistical assumptions supporting any sample size calculations	______________
Recruitment	15	Strategies for achieving adequate participant enrolment to reach target sample size	______________

Methods: Assignment of interventions (for controlled trials)

Allocation:

Sequence generation	16a	Method of generating the allocation sequence (eg, computer-generated random numbers), and list of any factors for stratification. To reduce predictability of a random sequence, details of any planned restriction (eg, blocking) should be provided in a separate document that is unavailable to those who enrol participants or assign interventions	______________
Allocation concealment mechanism	16b	Mechanism of implementing the allocation sequence (eg, central telephone; sequentially numbered, opaque, sealed envelopes), describing any steps to conceal the sequence until interventions are assigned	______________
Implementation	16c	Who will generate the allocation sequence, who will enrol participants, and who will assign participants to interventions	______________
Blinding (masking)	17a	Who will be blinded after assignment to interventions (eg, trial participants, care providers, outcome assessors, data analysts), and how	______________
	17b	If blinded, circumstances under which unblinding is permissible, and procedure for revealing a participant's allocated intervention during the trial	______________

Methods: Data collection, management, and analysis

Data collection methods	18a	Plans for assessment and collection of outcome, baseline, and other trial data, including any related processes to promote data quality (eg, duplicate measurements, training of assessors) and a description of study instruments (eg, questionnaires, laboratory tests) along with their reliability and validity, if known. Reference to where data collection forms can be found, if not in the protocol	______________
	18b	Plans to promote participant retention and complete follow-up, including list of any outcome data to be collected for participants who discontinue or deviate from intervention protocols	______________

For peer review only - http://bmjopen.bmj.com/site/about/guidelines.xhtml

Data management	19	Plans for data entry, coding, security, and storage, including any related processes to promote data quality (eg, double data entry; range checks for data values). Reference to where details of data management procedures can be found, if not in the protocol	______________
Statistical methods	20a	Statistical methods for analysing primary and secondary outcomes. Reference to where other details of the statistical analysis plan can be found, if not in the protocol	______________
	20b	Methods for any additional analyses (eg, subgroup and adjusted analyses)	______________
	20c	Definition of analysis population relating to protocol non-adherence (eg, as randomised analysis), and any statistical methods to handle missing data (eg, multiple imputation)	______________
**Methods: Monitoring**			
Data monitoring	21a	Composition of data monitoring committee (DMC); summary of its role and reporting structure; statement of whether it is independent from the sponsor and competing interests; and reference to where further details about its charter can be found, if not in the protocol. Alternatively, an explanation of why a DMC is not needed	______________
	21b	Description of any interim analyses and stopping guidelines, including who will have access to these interim results and make the final decision to terminate the trial	______________
Harms	22	Plans for collecting, assessing, reporting, and managing solicited and spontaneously reported adverse events and other unintended effects of trial interventions or trial conduct	______________
Auditing	23	Frequency and procedures for auditing trial conduct, if any, and whether the process will be independent from investigators and the sponsor	______________
**Ethics and dissemination**			
Research ethics approval	24	Plans for seeking research ethics committee/institutional review board (REC/IRB) approval	______________
Protocol amendments	25	Plans for communicating important protocol modifications (eg, changes to eligibility criteria, outcomes, analyses) to relevant parties (eg, investigators, REC/IRBs, trial participants, trial registries, journals, regulators)	______________

For peer review only - http://bmjopen.bmj.com/site/about/guidelines.xhtml

Consent or assent	26a	Who will obtain informed consent or assent from potential trial participants or authorised surrogates, and how (see Item 32)	________
	26b	Additional consent provisions for collection and use of participant data and biological specimens in ancillary studies, if applicable	________
Confidentiality	27	How personal information about potential and enrolled participants will be collected, shared, and maintained in order to protect confidentiality before, during, and after the trial	________
Declaration of interests	28	Financial and other competing interests for principal investigators for the overall trial and each study site	________
Access to data	29	Statement of who will have access to the final trial dataset, and disclosure of contractual agreements that limit such access for investigators	________
Ancillary and post-trial care	30	Provisions, if any, for ancillary and post-trial care, and for compensation to those who suffer harm from trial participation	________
Dissemination policy	31a	Plans for investigators and sponsor to communicate trial results to participants, healthcare professionals, the public, and other relevant groups (eg, via publication, reporting in results databases, or other data sharing arrangements), including any publication restrictions	________
	31b	Authorship eligibility guidelines and any intended use of professional writers	________
	31c	Plans, if any, for granting public access to the full protocol, participant-level dataset, and statistical code	________
**Appendices**			
Informed consent materials	32	Model consent form and other related documentation given to participants and authorised surrogates	________
Biological specimens	33	Plans for collection, laboratory evaluation, and storage of biological specimens for genetic or molecular analysis in the current trial and for future use in ancillary studies, if applicable	________

*It is strongly recommended that this checklist be read in conjunction with the SPIRIT 2013 Explanation & Elaboration for important clarification on the items. Amendments to the protocol should be tracked and dated. The SPIRIT checklist is copyrighted by the SPIRIT Group under the Creative Commons "Attribution-NonCommercial-NoDerivs 3.0 Unported" license.

For peer review only
